# [3-N_2_-*o*-C_2_B_10_H_11_][BF_4_]: a useful synthon for multiple cage boron functionalizations of *o*-carborane†
†Electronic supplementary information (ESI) available: Experimental details, complete characterization data, and X-ray data in CIF format for **4** and **6**. CCDC 1473011 and 1473012. For ESI and crystallographic data in CIF or other electronic format see DOI: 10.1039/c6sc01566b


**DOI:** 10.1039/c6sc01566b

**Published:** 2016-06-08

**Authors:** Da Zhao, Zuowei Xie

**Affiliations:** a Department of Chemistry and State Key Laboratory of Synthetic Chemistry , The Chinese University of Hong Kong , Shatin, New Territories , Hong Kong , China . Email: zxie@cuhk.edu.hk

## Abstract

A simple and efficient method for selective cage B(3) multiple functionalization of *o*-carborane is described. Reaction of [3-N_2_-*o*-C_2_B_10_H_11_][BF_4_] with various kinds of nucleophiles gave a very broad spectrum of cage B(3)-substituted *o*-carborane derivatives, 3-X-*o*-C_2_B_10_H_11_ (X = OH, SCN, NH_2_, NO_2_, N_3_, CF_3_, PO(C_6_H_5_)_2_, *etc*). This reaction may serve as another efficient [^18^F]-radiolabeling method of carborane clusters for positron emission tomography applications.

## Abstract 2

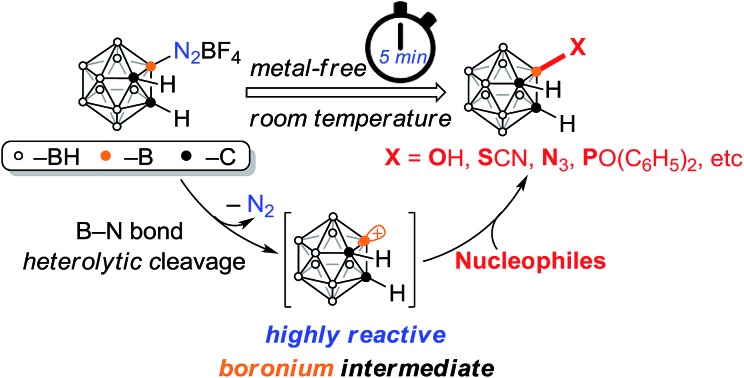
Reaction of [3-N_2_-*o*-C_2_B_10_H_11_][BF_4_] with various kinds of nucleophiles gives a very broad spectrum of cage B(3)-substituted *o*-carborane derivatives, 3-X-*o*-C_2_B_10_H_11_ (X = OH, SCN, NH_2_, NO_2_, N_3_, CF_3_, PO(C_6_H_5_)_2_, *etc.*), and serves as a simple and efficient method for multiple functionalization of *o*-carborane.

## Introduction

Carboranes, 3-dimensional relatives of benzenes, are a class of boron hydride clusters in which one or more BH vertices are replaced by CH units.^1^ Carboranes and organic molecules display different electronic, physical, chemical and geometrical properties, which highlights the feasibility or necessity to produce hybrid molecules incorporating both of these two types of fragments.2a,b Indeed, functional carboranes are now finding a broad range of applications encompassing organic synthesis, polymers, catalysis, metal–organic frameworks, electronic devices and more.2c–r As a result, considerable attention has been directed towards the functionalization of carborane molecules.^3^ In contrast to the relatively well-studied methods for cage carbon functionalization of carboranes,^1^,^4^ selective cage boron functionalization of carboranes still represents a challenging task and developing new methodologies for selective boron derivatization is eagerly desired.^5^,^6^

Diazonium compounds (R-N_2_^+^X^–^) constitute an important group of intermediates that have found wide applications in organic synthesis.^7^ Many prominent named reactions associated with aryl diazonium salts have been developed since their first discovery in 1858.^8^ In sharp contrast, diazonium derivatives of carboranes are little known.^9^ It has been documented that *o*-carboranyl diazonium salts are non-isolable, can only be prepared *in situ* and undergo substitution reactions with the reaction solvent, usually inorganic acids, in the presence of copper salts.^10^ Recently, we have reported the synthesis of a stable and isolable *o*-carboranyl diazonium salt, [3-N_2_-*o*-C_2_B_10_H_11_][BF_4_].11*a* It serves as an ideal precursor for the generation of 1,3-dehydro-*o*-carborane^11^ and boron-centered carboranyl radicals.^12^

On the other hand, it has been reported that B(9)-carboranyl iodonium salt can react with nucleophiles.^13^ Very recently, a similar approach for the functionalization of *closo*-borates *via* nucleophilic substitution reactions of the corresponding iodonium zwitterions has been developed.^14^ However, in these cases, only limited nucleophiles are tolerated and the chemoselectivity of the reaction is highly dependent on the nature of the nucleophiles or the reaction conditions.13c,^14^ As the most widely investigated among the carborane family, general and versatile methods for selected cage boron functionalization of *o*-carboranes still remain very limited.^5^ Previously, our group has reported that utilizing 3-diazonium-*o*-carborane tetrafluoroborate as the starting material, selective B(3)-arylation of *o*-carborane can be achieved *via* the aromatic ene reaction of 1,3-dehydro-*o*-carborane or a visible-light mediated B–C(sp^2^) coupling of a carboranyl boron-centered radical. However, the substrate scope is only limited to arenes.11a,^12^

**Scheme 1:**
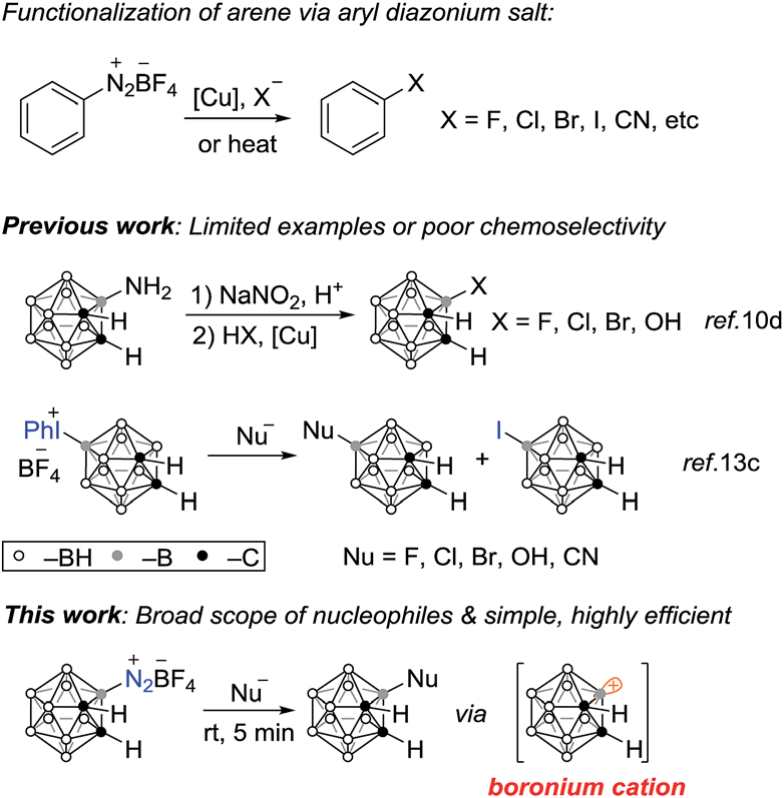
Functionalization of arene and *o*-carborane *via* diazonium salt.

Considering that dinitrogen is an excellent leaving group, carboranyl diazonium salt may easily undergo a substitution reaction in the presence of a nucleophile. Moreover, compared to aryl diazonium salts, carboranyl diazonium salt may exhibit higher reactivity due to the electron deficient nature of the boron atom and lack of conjugation between the carborane cage and the diazonium group. Herein, we report a proof-of-concept study demonstrating that carboranyl diazonium salt can serve as a powerful synthon for selective cage boron functionalization of *o*-carboranes (Scheme 1).

## Results and discussion

3-Diazonium-*o*-carborane tetrafluoroborate ([3-N_2_-*o*-C_2_B_10_H_11_][BF_4_]; **1**) was prepared in 77% isolated yield, by treatment of 3-amino-*o*-carborane with 1.5 equivalents of nitrosonium tetrafluoroborate.11*a* It is noted that the stability of **1** is dependent upon the counterions used and BF_4_^–^ offers the highest thermal stability of the salt among the anions examined, such as PF_6_^–^ and Cl^–^. A 1.0 g batch of carboranyl diazonium salt **1** stored at –5 °C showed no signs of decomposition over four months.

With this stable precursor in hand, we found that precursor **1** reacted rapidly with various nucleophiles (**2**) in acetonitrile, providing the corresponding B(3)-substituted *o*-carboranes in good to excellent yields (Table 1). Treatment of **1** with 1 equivalent of strong (charged) nucleophiles, such as halide ions, gave the corresponding halogenated carboranes in excellent yields in <5 min (Table 1, entry 1).

**Table 1:** Reaction of nucleophiles with precursor **1**^*a*^

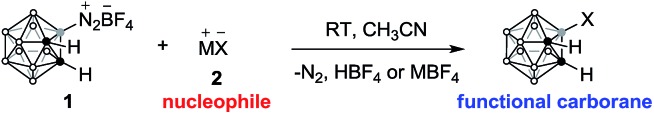
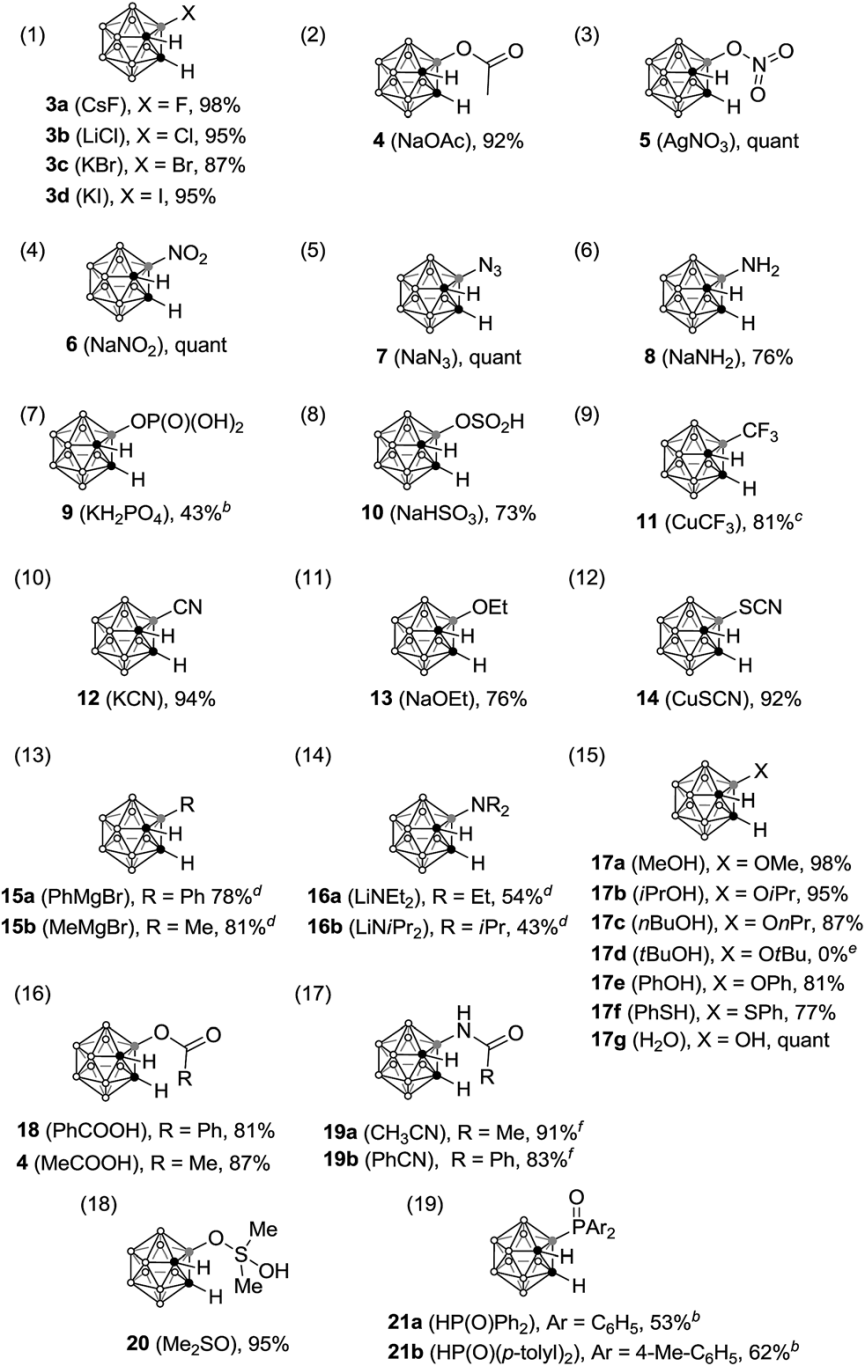

^*a*^Reaction conditions: precursor **1** (0.1 mmol) was treated with nucleophile **2** (0.1 mmol for inorganic salt and phosphine oxide; 1.0 mmol for alcohol, acid and ketone; 0.4 mmol for Grignard reagent and lithium amide; nitriles were utilized as solvent) in CH_3_CN solution for 5 min; yields of isolated products are given.

^*b*^Deboronation occurred during purification.

^*c*^CuCF_3_ was prepared *in situ* from TMSCF_3_, CuSCN and Cs_2_CO_3_ in acetonitrile.^16^

^*d*^–78 °C, THF, 15 min.

^*e*^The only product was 3-F-C_2_B_10_H_11_.

^*f*^50 °C, 6 h.

A large variety of nucleophiles, including inorganic salts, water, alcohols, acids, organometallic reagents, ketones, nitriles and phosphine oxides are compatible with this reaction, resulting in the formation of B–C, B–N, B–P, B–O, B–S and B–X (X = F, Cl, Br, I) bonds. More importantly, various functional groups that were previously unable to be introduced into the carborane unit can now be installed in a very simple and efficient manner. For instance, common functional groups can be easily installed on the *o*-carborane cage boron position using simple inorganic salts in 5 min, affording the corresponding B(3)-functionalized *o*-carboranes **3–14** (Table 1, entries 1–12). Reaction of precursor **1** with Grignard reagents or lithium amides also gave the B(3)-substituted *o*-carboranes in moderate to good yields (Table 1, entries 13 and 14).

Weak nucleophiles also work well in this reaction. For instance, in the presence of 10 equivalents of alcohols or water, B(3)-oxygenated carboranes **17** were produced in 81–98% yield (Table 1, entry 15). However, no desired product was observed for *tert*-butyl alcohol, probably due to the steric hindrance imposed by the *tert*-butyl group. Instead, 3-F-*o*-carborane **3a**, generated *via* decomposition of precursor **1**, was the only isolated product. Compared to other neutral nucleophiles, the reaction of nitriles is slower even at elevated temperature (Table 1, entry 17).^15^ The reactivity of precursor **1** towards nucleophiles containing P=O and S=O double bonds was also examined. For example, the reaction of dimethyl sulfoxide furnished compound **20** after hydrolysis (Table 1, entry 18). Although ^31^P and ^11^B NMR spectra indicated high conversions, reactions with phosphine oxide nucleophiles resulted in lower yields due to the deboronation of the product during the purification process (Table 1, entry 19).^17^ Notably, this metal-free approach provides a rare example of B-carboranyl phosphines.^18^ The rich chemistry of the carboranyl diazonium salt towards various nucleophiles suggests that it can serve as a very promising synthon for selective cage boron functionalization of *o*-carboranes. It is noteworthy that the reaction also works well when performed on a 0.5 mmol scale.^17^

All new compounds were fully characterized by ^1^H, ^13^C, and ^11^B NMR spectroscopy as well as HRMS spectrometry.

The molecular structures of compounds **4** and **6** were further confirmed by single-crystal X-ray analyses.^17^

**Scheme 2:**
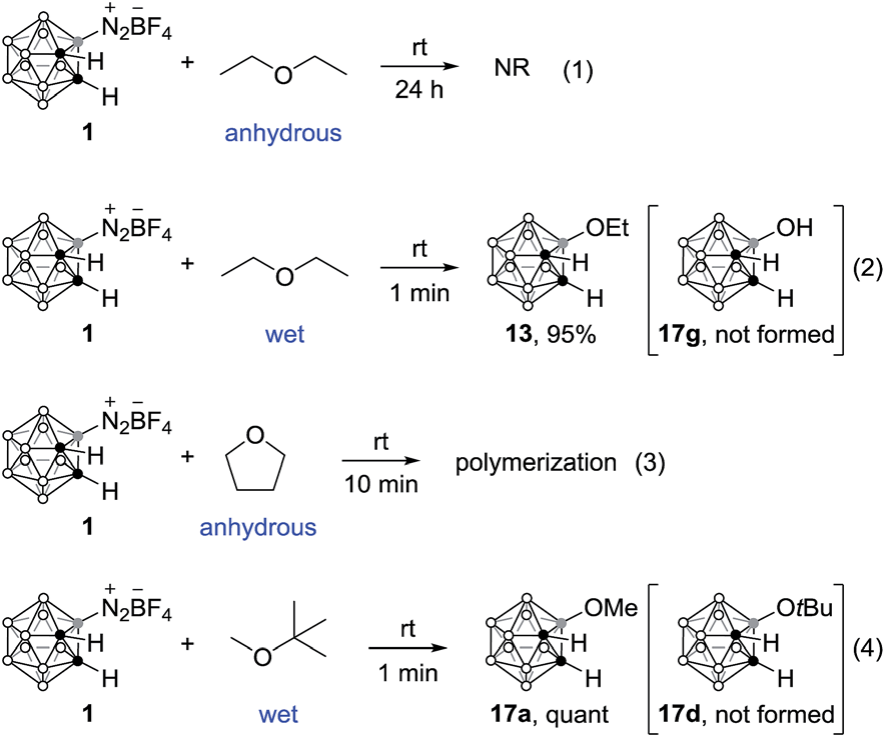
Reaction of precursor **1** with ethers.

Interestingly, precursor **1** did not react with anhydrous ether (Scheme 2, eqn (1)); however, it reacted rapidly with wet ethereal solvents. For instance, upon treatment with wet diethyl ether, compound **13**, resulting from the C–O bond cleavage of ether, was isolated in 95% yield (Scheme 2, eqn (2)). When treated with anhydrous THF, polymerization occurred, leading to gel formation, which suggests the intermediacy of cationic species (Scheme 2, eqn (3)).^17^ When *tert*-butyl methyl ether was examined under the same reaction conditions, compound **17a**, bearing a methoxy substituent at B(3) position, was formed quantitatively (Scheme 2, eqn (4)), which may shed some light on the reaction mechanism (*vide infra*).

**Scheme 3:**
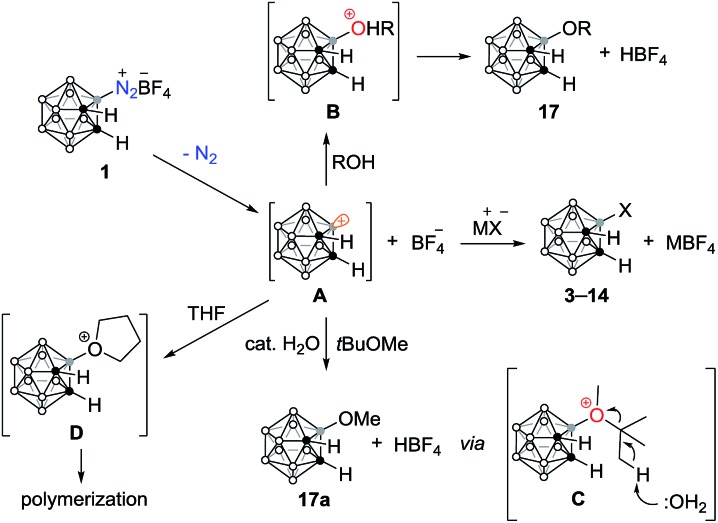
Proposed reaction pathways.

The nucleophilic reaction of the carboranyl diazonium salt was expected to proceed through an S_N_1 type of mechanism (Scheme 3).^9^,^19^ Although precursor **1** is stable in solution, it can undergo nucleophile-induced heterolytic B–N bond cleavage, producing a boronium intermediate **A**.^14^ Similar to the reaction of the dinitrogen derivatives of *closo*-borates, the rate-determining step is the B–N bond cleavage.^9^ The resultant reactive boronium intermediate can be trapped by various nucleophiles. For instance, when charged nucleophiles such as inorganic salts were employed as nucleophiles, the corresponding substituted compounds **3–14** were formed in very high yields within 5 min. If the nucleophiles are strong bases, the addition products **15–17** might also be produced *via* the intermediacy of 1,3-dehydro-*o*-carborane intermediates.^11^ Addition of neutral nucleophiles to the boronium intermediate **A**, alcohols for example, generates an oxonium ion **B**, which is further deprotonated by the BF_4_^–^ anion to afford **17**. For weakly nucleophilic ethers, such as *tert*-butyl methyl ether, no reaction occurs under anhydrous conditions. However, in the presence of a catalytic amount of water, the oxygenated products, such as **17a**, were produced within 5 min. This reaction probably proceeds through a sequence of C–H bond cleavage/isobutylene elimination in intermediate **C**, which is generated by the nucleophilic addition of the ether to the naked boron vertex of intermediate **A**.^20^ The role of the catalytic amount of water is to facilitate the isobutylene elimination that was detected by GC-MS analyses. The formation of HBF_4_ was also confirmed by ^11^B and ^19^F NMR spectra.^17^

The present strategy provides a straightforward and practical access to cage boron functionalized *o*-carboranes. It has been documented that ^18^F-labelled (*t*_1/2_ = 109.8 min) carboranes are promising radiotracers in Positron Emission Tomography (PET). Previously, ^18^[F]-fluorination of *o*-carborane was achieved by nucleophilic substitution of a B(9)-carboranyl iodonium bromide.^21^ However, the overall synthesis time of 20 min limits its possible application, probably due to the low reactivity of the carboranyl iodonium bromide. As a proof of concept, we opted to improve the efficiency of the fluorination process by using precursor **1** as the starting material. Under similar reaction conditions to those reported in the literature,^17^ the fluorinated product **3a** was formed quantitatively within 1 min and it can be easily purified (eqn (5)).
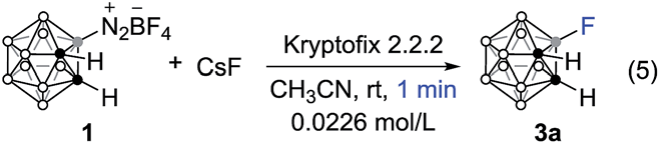


## Conclusions

A practical method for selective cage boron functionalization of *o*-carborane has been developed. By utilizing B-carboranyl diazonium salt as a synthon, a large class of *o*-carborane derivatives bearing previously inaccessible functional groups can now be efficiently prepared, which may find applications in materials sciences.

This work demonstrates that B-carboranyl diazonium salt can serve not only as a source of boron-centered radicals^12^ or 1,3-dehydro-*o*-carborane,^11^ but also as a source of boronium cations in the presence of nucleophiles.^9^,^20^ These intermediates serve different purposes and are complementary to each other, building up a useful toolbox for cage boron functionalization of *o*-carboranes.

Compared to aryl diazonium salts, the exceptionally high reactivity of B-carboranyl diazonium salt may be due to the lack of conjugation between the carborane cage and the diazonium group. Such a method may find useful applications in the efficient and fast synthesis of ^18^F-labelled *o*-carborane derivatives for medical applications.^21^

## Supplementary Material

Supplementary information

Crystal structure data
