# Supplementary material for: [3-N2-o-C2B10H11][BF4]: a useful synthon for multiple cage boron functionalizations of o-carborane
Source: Chem Sci. 2016 Jun 8;7(9):5635–9. doi: 10.1039/c6sc01566b (PMC6022115; doi:10.1039/c6sc01566b)
Supplement: Supplementary file 1 [file SC-007-C6SC01566B-s001.pdf]

Electronic Supplementary Material (ESI) for Chemical Science.
This journal is © The Royal Society of Chemistry 2016

Electronic Supplementary Information

[3-N_2-*o*-$C_2B_{10}H_{11}$][BF_4]: A Useful Synthon for Multiple Cage Boron Functionalization of *o*-Carborane

*Da Zhao and Zuowei Xie**

Department of Chemistry and State Key Laboratory of Synthetic Chemistry, The Chinese University of Hong Kong, Shatin, New Territories, Hong Kong, China

Table of Contents

General Information	S2
Synthesis of starting materials	S2
General procedure for the reaction of carboranyl diazonium salt **1** with representative nucleophiles	S4
General procedure for the reaction of carboranyl diazonium salt **1** with other nucleophiles	S6
A 0.5 mmol scale reaction of carboranyl diazonium salt **1** with CsF	S12
Reaction of carboranyl diazonium salt **1** with ethers	S13
Formation of HBF_4 in the reaction of carboranyl diazonium salt **1** with methanol	S15
Proof-of-concept synthesis of 3-fluoro-*o*-carborane under reported reaction conditions	S16
References	S17
NMR Spectra	S18

General Information.

All reactions were carried out under an atmosphere of dry argon with the rigid exclusion of air and moisture in a glovebox unless otherwise specified. ^{1}H and ^{13}C NMR spectra were recorded on a Bruker DPX 400 spectrometer at 400 and 100 MHz, respectively. ^{11}B NMR spectra were recorded on a Bruker DPX 300 spectrometer at 96 MHz or a Varian Inova 400 spectrometer at 128 MHz. ^{31}P NMR spectra were recorded on a Bruker DPX 300 spectrometer at 162 MHz. All signals were reported in ppm with reference to the residual solvent resonances of the deuterated solvents for proton and carbon chemical shifts, to external $BF_3 \cdot OEt_2$ (0.00 ppm) for boron chemical shifts, to external 85% H_3PO_4 (0.00 ppm) for phosphorus chemical shifts, and to external $CFCl_3$ (0.00 ppm) for fluorine chemical shifts. The data were reported as follows: chemical shift, multiplicity (s = singlet, d = doublet, t = triplet, q = quadruplet, m = multiplet or unresolved, br = broad), coupling constant(s) in Hz, integration, and assignment. Mass spectra were obtained on a Thermo Finnigan MAT 95 XL spectrometer or Waters Micromass GCT Premier. The thermal property was investigated with TA Instruments (model Q1000) at a heating rate of 5 ^{o}C/min (Purge flow: 50mL/min under N_2). All organic solvents were freshly distilled from Na-K alloy or CaH_2 immediately prior to use. 3-Diazonium-*o*-carborane tetrafluoroborate was prepared according to literature method.[1] All other chemicals were purchased from either Aldrich or Acros Chemical Co. and used as received unless otherwise specified.

Synthesis of starting materials

Preparation of 3-amino-*o*-carborane. A 250 mL three-neck round-bottom flask was charged with *o*-carborane (*o*-$C_2B_{10}H_{12}$, 5.0 g, 34.7 mmol) and a stir bar under argon atmosphere. Liquid ammonia (150 mL) was collected in the same flask using a dry ice-acetone ammonia condenser. At -78 ^{o}C, finely cut sodium metal (1.68 g, 72.5 mmol) was added slowly over 30 min through a side arm using a protective blast shield. At the end of the addition of the sodium metal, the ammonia solution turned into a deep blue due to excess sodium metal. The reaction mixture was then allowed to stir at -40 ^{o}C for 1.5 h (do not

lower the temperature to -50 ^{o}C or below. Otherwise, the *nido*-$C_2B_{10}H_{12}$ reacts very slowly with liquid ammonia). Then the reaction mixture was cooled to -78 ^{o}C and potassium permanganate (12.0 g, 72.5 mmol) was slowly added over 30 min through the side arm using a protective blast shield. The reaction mixture was allowed to stir for another 2 h at -78 ^{o}C. *tert*-Butyl alcohol (10 mL) was then slowly added with caution at -78 ^{o}C, followed by 50% aqueous *tert*-butyl alcohol (20 mL) to destroy excess sodium in the reaction mixture. After that, ethyl acetate (30 mL) and water (30 mL) were slowly added in sequence. The reaction was allowed to warm up slowly to room temperature, during which ammonia was evaporated. The reaction mixture was filtered through a Buchner funnel to remove MnO_2 and washed with ethyl acetate (2 x 30 mL). The organic layers were separated and dried over sodium sulfate. After removal of solvents in vacuo, the residue was subjected to flash column chromatography on silica gel (230-400 mesh) using *n*-hexane/EtOAc (50/1 to 10/1 in V/V) as eluent to give 3-amino-*o*-carborane as a white solid (4.8 g, 88%). (**CAUTION:** This reaction is potentially explosive and should be carried out with protection).

Preparation of 3-diazonium-*o*-carborane tetrafluoroborate (1). To an anhydrous acetonitrile solution (1.5 mL) of 3-amino-*o*-carborane (318.0 mg, 2.0 mmol) was added $NOBF_4$ (323.5 mg, 3.0 mmol) at -30 ^{o}C and the reaction mixture was stirred at -15 ^{o}C for 1.5 h. Dry ether (10 mL) was then added at -30 ^{o}C to the reaction mixture, from which **1** was precipitated out. Solvent was transferred via a cannula and the remaining solid was further washed by dry ether (2 mL × 2). After removal of ether via a cannula, the crystalline solid was dried under vacuum to give **1** as a white solid (402.5 mg, 78%). (**CAUTION:** Although spontaneous detonate or explosion has not been observed in our laboratory upon synthesizing or handling the dry diazonium salt **1**, we recommend to handle this compound with care). Compound **1** displayed DSC exotherm at ~104.9 °C as depicted in Figure S1. IR (KBr, cm^{-1}): 3067 (cage CH), 2645, 2617, 2592 (cage BH), 2357 (N_2).

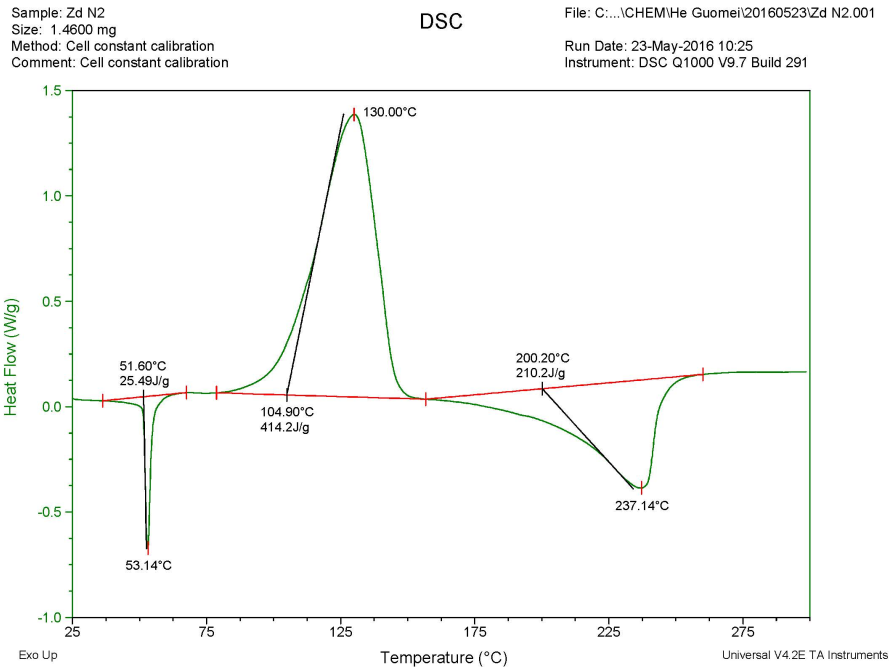

Figure S1. Thermal property of 3-diazonium salt **1** tested by differential scanning calorimeter (TA Instruments, model Q1000); Headspace gas: nitrogen (50 mL/min), temperature range: 25 to 800 ^{o}C, temperature rate: 5 ^{o}C/min). The exothermic peak at 130.0 ^{o}C can be assigned to the decomposition process of **1**. It is not clear what process is related to the first endothermic peak at 53.1 ^{o}C. The second endothermic peak at 237.1 ^{o}C corresponds to the sublimation process of 3-F-*o*-carborane (generated by thermal decomposition of **1**).

General procedure for the reaction of carboranyl diazonium salt 1 with representative nucleophiles such as CsF, LiCl, MeOH, and H_2O (Table 1, entries 1 and 15). 3-Diazonium-*o*-carborane tetrafluoroborate (1; 25.8 mg, 0.1 mmol) and nucleophiles (0.1 or 1.0 mmol) were dissolved in dry CH_3CN (2 mL) in an open 10 mL Schlenk flask equipped with a magnetic stirring bar. In all cases, bubbles were immediately observed after the nucleophile was added. Under an atmosphere of dry argon, the reaction mixture was further stirred at room temperature for 5 min. Then the reaction was stopped and the resulting mixture was examined by GC-MS analysis. After removal of solvents in vacuo, the residue was examined by ^{1}H NMR spectrum and then subjected to flash column chromatography on Al_2O_3 (activated, neutral,

Merck, 70-230 mesh mesh) using *n*-hexane/EtOAc (100/1 in v/v) as eluent to give the desired product.

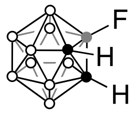

3a: White solid. Yield: 98%. ^{1}H NMR (400 MHz, $CDCl_3$): δ 3.76 (br, 2H) (cage C*H*). $^{19}F\{^{1}H\}$ NMR (376 MHz, $CDCl_3$): δ -201.1 (q, $J_{F\text{-}B}$ = 56.4 Hz). $^{11}B\{^{1}H\}$ NMR (128 MHz, $CDCl_3$): δ -1.1 (1B), -5.5 (2B), -12.7 (1B), -15.2 (3B), -16.5 (2B), -20.0 (1B). The chemical shifts are in accordance with the reported data.[2]

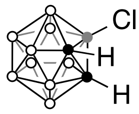

3b: White solid. Yield: 95%. ^{1}H NMR (400 MHz, $CDCl_3$): δ 3.81 (br, 2H) (cage C*H*). The chemical shifts are in accordance with the reported data.[2]

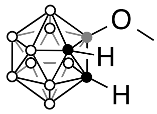

17a: White solid. Yield: 81%. ^{1}H NMR (400 MHz, $CDCl_3$): δ 3.69 (s, 3H) (CH_3), 3.56 (br, 2H) (cage C*H*). $^{13}C\{^{1}H\}$ NMR (100 MHz, $CDCl_3$): δ 57.4 (cage *C*), 55.9. $^{11}B\{^{1}H\}$ NMR (128 MHz, $CDCl_3$): δ 1.3 (1B), -5.4 (2B), -13.1 (1B), -15.0 (3B), -16.1 (2B), -19.9 (1B). HRMS (EI) calcd for $C_3H_{14}{}^{11}B_8{}^{10}B_2O^+$: 174.0841. Found 174.0837.

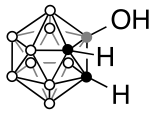

17g: Colorless oil. Yield: quant. ^{1}H NMR (400 MHz, $CDCl_3$): δ 4.43 (br, 2H) (cage C*H*). The chemical shifts are in accordance with the reported data.[3]

For the reaction of 1 with acetonitrile (Table 1, entry 17). Under an atmosphere of dry argon, a dry CH_3CN (2 mL) solution of 3-diazonium-*o*-carborane tetrafluoroborate (**1**; 25.8 mg, 0.1 mmol) in a 10 mL Schlenk flask was stirred at indicated temperatures (room temperature for 48 h or 50 °C for 6 h). Then the reaction was stopped and the resulting mixture was examined by GC-MS analysis. After removal of solvents in vacuo, the residue was examined by ^{1}H NMR spectrum and then subjected to flash column chromatography on Al_2O_3 (activated, neutral, Merck, 70-230 mesh mesh) using *n*-hexane/EtOAc (100/1 in v/v) as eluent to give the hydrolysis product **19a** as a colorless oil (eq S1).

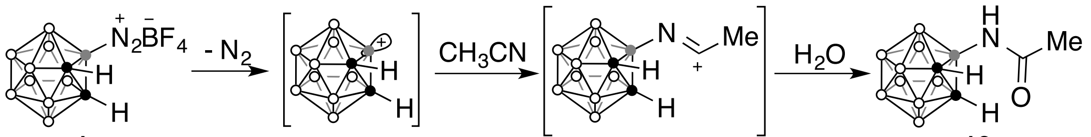

1 boronium cation **19a** (S1)

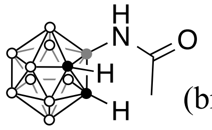

19a: Colorless oil. Yield: 91%. ^{1}H NMR (400 MHz, CD_2Cl_2): δ 5.78 (br, 1H) (N*H*), 4.48 (br, 2H), (cage C*H*), 2.04 (s, 3H) (C*H*$_3$). ^{13}C{^{1}H} NMR (100 MHz, CD_2Cl_2): δ 175.0, 55.6 (cage *C*), 25.1. ^{11}B{^{1}H} NMR (128 MHz, CD_2Cl_2): δ -3.0 (2B), -4.3 (1B), -10.2 (1B), -13.4 (2B), -14.1 (4B). HRMS (EI) calcd for $C_4H_{15}{}^{11}B_8{}^{10}B_2NO^+$: 201.1094. Found 201.1095.

General procedure for reaction of carboranyl diazonium salt 1 with other nucleophiles.

3-Diazonium-*o*-carborane tetrafluoroborate (**1**; 25.8 mg, 0.1 mmol) and nucleophiles (0.1 mmol for inorganic salt and phosphine oxide; 1.0 mmol for alcohol, acid and ketone; 0.4 mmol for Grignard agent and lithium amide; nitriles were utilized as solvent) were dissolved in 2 mL dry CH_3CN (THF for Grignard agents and lithium amides) in an open 10 mL Schlenk flask equipped with a magnetic stirring bar. Under an atmosphere of dry argon, the reaction mixture was further stirred at room temperature(-78 ^{o}C for Grignard agents and lithium amides, 50 ^{o}C for nitriles) for 5 min (15min for Grignard agents and lithium amides, 6 h for nitriles). Then the reaction was stopped and the resulting mixture was examined by GC-MS analysis. After removal of solvents in vacuo, the residue was examined by ^{1}H NMR spectrum and then subjected to flash column chromatography on Al_2O_3 (activated, neutral, Merck, 70-230 mesh mesh) using *n*-hexane/EtOAc (100/1 in v/v) as eluent to give the desired product.

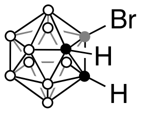

3c: White solid. Yield: 87%. ^{1}H NMR (400 MHz, $CDCl_3$): δ 3.84 (br, 2H) (cage C*H*). The chemical shifts are in accordance with the reported data.[4]

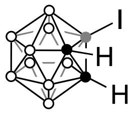

3d: White solid. Yield: 95%. ^{1}H NMR (400 MHz, CD_2Cl_2): δ 3.84 (br, 2H) (cage C*H*). The

chemical shifts are in accordance with the reported data.[5]

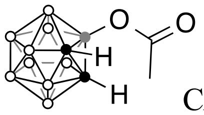

4: Colorless crystals. Yield: 92%. ^{1}H NMR (400 MHz, $CDCl_3$): δ 4.22 (br, 2H) (cage C*H*), 2.17 (s, 3H) (C*H*$_3$). ^{13}C{^{1}H} NMR (100 MHz, $CDCl_3$): δ 170.4, 55.0 (cage *C*), 22.6. ^{11}B{^{1}H} NMR (128 MHz, $CDCl_3$): δ -5.0 (3B), -11.1 (1B), -14.0 (2B), -14.8 (1B), -15.9 (3B). HRMS (EI) calcd for $C_4H_{14}{}^{11}B_8{}^{10}B_2O_2{}^{+}$: 202.0942. Found 202.0937.

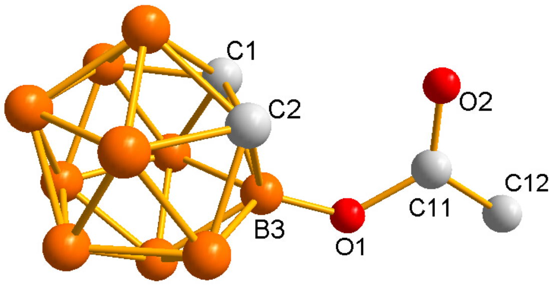

Figure S2. Molecular Structure of **4**.

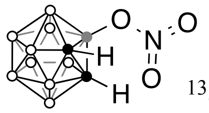

5: White solid. Yield: quant. ^{1}H NMR (400 MHz, $CDCl_3$): δ 3.95 (br, 2H) (cage C*H*). ^{13}C{^{1}H} NMR (100 MHz, $CDCl_3$): δ 55.7 (cage *C*). ^{11}B{^{1}H} NMR (128 MHz, $CDCl_3$): δ -3.7 (2B), -4.2 (1B), -10.4 (1B), -14.0 (2B), -15.1 (4B). HRMS (EI) calcd for $C_2H_{11}{}^{11}B_8{}^{10}B_2NO_3{}^{+}$: 205.0550. Found 205.0543.

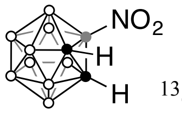

6: Colorless crystals. Yield: quant. ^{1}H NMR (400 MHz, $CDCl_3$): δ 3.89 (br, 2H) (cage C*H*). ^{13}C{^{1}H} NMR (100 MHz, $CDCl_3$): δ 55.0 (cage *C*). ^{11}B{^{1}H} NMR (128 MHz, $CDCl_3$): δ -3.5 (3B), -10.2 (1B), -13.8 (2B), -14.8 (4B). HRMS (EI) calcd for $C_2H_{11}{}^{11}B_8{}^{10}B_2NO_2{}^{+}$: 189.0556. Found 189.0553.

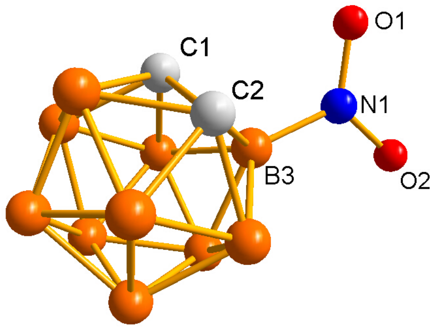

Figure S3. Molecular Structure of **6**.

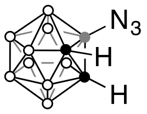

7: White solid. Yield: quant. ^{1}H NMR (400 MHz, $CDCl_3$): δ 3.61 (br, 2H) (cage C*H*). $^{13}C\{^1H\}$ NMR (100 MHz, $CDCl_3$): δ 56.0 (cage *C*). $^{11}B\{^1H\}$ NMR (128 MHz, $CDCl_3$): δ -3.8 (2B), -5.1 (1B), -11.1 (1B), -14.2 (4B), -14.9 (2B). HRMS (EI) calcd for $C_2H_{11}{}^{11}B_8{}^{10}B_2N_3{}^+$: 185.0702. Found 185.0701. IR (KBr, cm^{-1}) : 3066 (cage CH), 2574 (cage BH), 2149 (N_3).

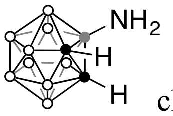

8: White solid. Yield: 76%. ^{1}H NMR (400 MHz, $CDCl_3$): δ 4.21 (br, 2H) (cage C*H*). The chemical shifts are in accordance with the reported data.[6]

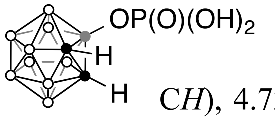

9: Colorless oil. Yield: 43%. ^{1}H NMR (400 MHz, acetone-d_6): δ 4.88 (br, 2H) (cage C*H*), 4.72 (br, 2H) (O*H*). $^{13}C\{^1H\}$ NMR (100 MHz, acetone-d_6): δ 58.5 (cage *C*). $^{11}B\{^1H\}$ NMR (128 MHz, acetone-d_6): δ -2.8 (1B), -5.0 (2B), -11.0 (1B), -13.8 (2B), -15.0 (3B), -16.3 (1B). HRMS (EI) calcd for $C_2H_{13}{}^{11}B_8{}^{10}B_2PO_4{}^+$: 240.0374. Found 240.0371.

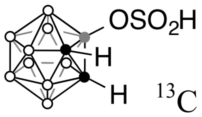

10: Colorless oil. Yield: 73%. ^{1}H NMR (400 MHz, $CDCl_3$): δ 4.21 (br, 2H) (cage C*H*). $^{13}C\{^1H\}$ NMR (100 MHz, $CDCl_3$): δ 58.8 (cage *C*). $^{11}B\{^1H\}$ NMR (128 MHz, $CDCl_3$): δ 0.7 (1B), -3.5 (2B), -10.7 (1B), -13.1 (2B), -14.4 (3B), -17.6 (1B). HRMS (EI) calcd for $C_2H_{12}{}^{11}B_8{}^{10}B_2SO_3{}^+$: 224.1210. Found 224.1203.

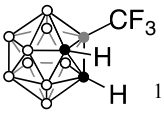

11: White solid. Yield: 73%. ^{1}H NMR (400 MHz, $CDCl_3$): δ 3.91 (br, 2H) (cage C*H*). ^{13}C{^{1}H} NMR (100 MHz, $CDCl_3$): δ 58.8 (cage *C*), the *C* connected to the cage B atom was not observed. ^{11}B{^{1}H} NMR (128 MHz, $CDCl_3$): δ 1.0 (1B), -3.3 (2B), -10.4 (1B), -12.9 (2B), -14.1 (3B), -17.3 (1B). HRMS (EI) calcd for $C_3H_{11}{}^{11}B_8{}^{10}B_2F_3{}^+$: 212.0560. Found 212.0554.

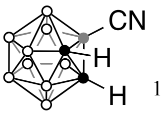

12: Colorless oil. Yield: 94%. ^{1}H NMR (400 MHz, $CDCl_3$): δ 3.54 (br, 2H) (cage C*H*). ^{13}C{^{1}H} NMR (100 MHz, $CDCl_3$): δ 53.9 (cage *C*), the *C* connected to the cage B atom was not observed. ^{11}B{^{1}H} NMR (128 MHz, $CDCl_3$): δ -0.9 (1B), -5.2 (2B), -12.4 (1B), -14.8 (2B), -16.1 (3B), -19.3 (1B). HRMS (EI) calcd for $C_3H_{11}{}^{11}B_8{}^{10}B_2N^+$: 169.0675. Found 169.0678.

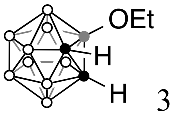

13: Colorless oil. Yield: 76%. ^{1}H NMR (400 MHz, $CDCl_3$): δ 3.96 (q, *J* = 7.2 Hz, 2H), 3.56 (br, 2H) (cage C*H*), 1.29 (t, *J* = 7.2 Hz, 3H). ^{13}C{^{1}H} NMR (100 MHz, $CDCl_3$): δ 66.0, 56.0 (cage *C*), 17.0. ^{11}B{^{1}H} NMR (128 MHz, $CDCl_3$): δ 0.98 (1B), -5.5 (2B), -13.0 (1B), -14.8 (2B), -15.5 (1B), -16.1 (2B), -20.0 (1B). HRMS (EI) calcd for $C_4H_{16}{}^{11}B_8{}^{10}B_2O^+$: 188.1107. Found 188.1105.

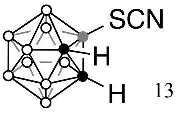

14: White solid. Yield: 92%. ^{1}H NMR (400 MHz, $CDCl_3$): δ 3.77 (br, 2H) (cage C*H*). ^{13}C{^{1}H} NMR (100 MHz, $CDCl_3$): δ 118.7, 56.7 (cage *C*). ^{11}B{^{1}H} NMR (128 MHz, $CDCl_3$): δ -3.78 (2B), -10.3 (1B), -12.0 (1B), -13.9 (2B), -15.0 (4B). HRMS (EI) calcd for $C_3H_{11}{}^{11}B_8{}^{10}B_2SN^+$: 201.1323. Found 201.1319.

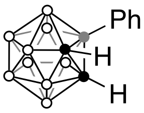

15a: White solid. Yield: 78%. ^{1}H NMR (400 MHz, $CDCl_3$): δ 8.01-7.31 (m, 5H) (aromatic C*H*), 3.71 (br, 2H) (cage C*H*). The chemical shifts are in accordance with the reported data.[7]

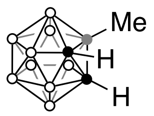

15b: White solid. Yield: 81%. ^{1}H NMR (400 MHz, $CDCl_3$): δ 4.03 (br, 2H) (cage C*H*), 2.09 (s, 3H) (CH_3). $^{13}C\{^{1}H\}$ NMR (100 MHz, $CDCl_3$): δ 55.5 (cage *C*), 22.9. $^{11}B\{^{1}H\}$ NMR (128 MHz, $CDCl_3$): δ -3.9 (3B), -10.0 (1B), -13.0 (2B), -13.7 (1B), -14.8 (3B). HRMS (EI) calcd for $C_3H_{14}{}^{11}B_8{}^{10}B_2{}^{+}$: 158.0847. Found 158.0844.

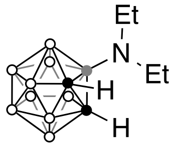

16a: Colorless oil. Yield: 54%. ^{1}H NMR (400 MHz, $CDCl_3$): δ 3.78 (br, 2H) (cage C*H*), 3.65 (m, 4H), 1.28 (m, 3H), 1.08 (m, 3H) (CH_3). $^{13}C\{^{1}H\}$ NMR (100 MHz, $CDCl_3$): δ 55.4 (cage *C*), 48.4, 39.6, 14.5, 11.2. $^{11}B\{^{1}H\}$ NMR (128 MHz, $CDCl_3$): δ -1.1 (1B), -4.5 (2B), -10.9 (1B), -13.7 (2B), -14.9 (3B), -16.3 (1B). HRMS (EI) calcd for $C_6H_{21}{}^{11}B_8{}^{10}B_2N^{+}$: 215.1792. Found 215.1797.

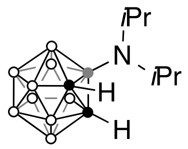

16b: Colorless oil. Yield: 43%. ^{1}H NMR (400 MHz, $CDCl_3$): δ 3.47 (br, 2H) (cage C*H*), 3.41 (m, 2H), 1.18 (d, *J* = 6.8 Hz, 12H) (CH_3). $^{13}C\{^{1}H\}$ NMR (100 MHz, $CDCl_3$): δ 56.8 (cage *C*), 49.0, 22.6. $^{11}B\{^{1}H\}$ NMR (128 MHz, $CDCl_3$): δ -1.14 (1B), -4.5 (2B), -10.9 (1B), -13.7 (2B), -14.9 (3B), -16.3 (1B). HRMS (EI) calcd for $C_8H_{25}{}^{11}B_8{}^{10}B_2N^{+}$: 243.2324. Found 243.2328.

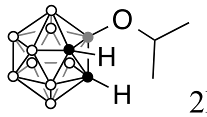

17b: Colorless oil. Yield: 95%. ^{1}H NMR (400 MHz, $CDCl_3$): δ 4.32 (m, 1H), 3.53 (br, 2H) (cage C*H*), 1.27 (d, *J* = 6.0 Hz, 6H). $^{13}C\{^{1}H\}$ NMR (100 MHz, $CDCl_3$): δ 73.0, 56.1 (cage *C*), 24.2. $^{11}B\{^{1}H\}$ NMR (128 MHz, $CDCl_3$): δ 1.3 (1B), -5.4 (2B), -13.1 (1B), -15.0 (3B), -16.1 (2B), -19.9 (1B). HRMS (EI) calcd for $C_5H_{18}{}^{11}B_8{}^{10}B_2O^{+}$: 202.1373. Found 202.1372.

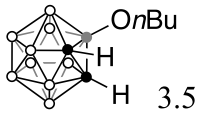

17c: Colorless oil. Yield: 87%. ^{1}H NMR (400 MHz, $CDCl_3$): δ 3.89 (t, *J* = 6.4 Hz, 2H), 3.56 (br, 2H) (cage C*H*), 1.62 (m, 2H), 1.39 (m, 2H), 0.94 (t, *J* = 7.2 Hz, 3H). $^{13}C\{^{1}H\}$ NMR (100 MHz, $CDCl_3$): δ 70.6, 56.0 (cage *C*), 33.3, 19.0, 13.9. $^{11}B\{^{1}H\}$ NMR (128 MHz, $CDCl_3$): δ 0.7 (1B), -5.9 (2B), -13.4 (1B), -15.3 (3B), -16.5 (2B), -20.4 (1B). HRMS (EI) calcd for $C_6H_{20}{}^{11}B_8{}^{10}B_2O^{+}$:

216.1639. Found 216.1635.

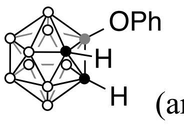

17e: Colorless oil. Yield: 81%. ^{1}H NMR (400 MHz, $CDCl_3$): δ 7.34 (m, 2H), 7.13 (m, 3H) (aromatic C*H*), 3.75 (br, 2H) (cage C*H*). $^{13}C\{^{1}H\}$ NMR (100 MHz, $CDCl_3$): δ 155.7, 130.0, 123.9, 119.8, 56.2 (cage *C*). $^{11}B\{^{1}H\}$ NMR (128 MHz, $CDCl_3$): δ -0.6 (1B), -4.9 (2B), -12.1 (1B), -14.5 (2B), -15.2 (1B), -15.8 (2B), -18.9 (1B). HRMS (EI) calcd for $C_8H_{16}{}^{11}B_8{}^{10}B_2O^+$: 236.1536. Found 236.1639.

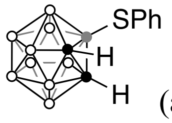

17f: Colorless oil. Yield: 77%. ^{1}H NMR (400 MHz, $CDCl_3$): δ 6.96 (m, 2H), 6.84 (m, 3H) (aromatic C*H*), 3.75 (br, 2H) (cage C*H*). $^{13}C\{^{1}H\}$ NMR (100 MHz, $CDCl_3$): δ 153.8, 131.3, 127.9, 121.6, 56.2 (cage *C*). $^{11}B\{^{1}H\}$ NMR (128 MHz, $CDCl_3$): δ -2.9 (2B), -9.5 (1B), -11.2 (1B), -12.1 (2B), -13.1 (1B), -14.1 (3B). HRMS (EI) calcd for $C_8H_{16}{}^{11}B_8{}^{10}B_2S^+$: 252.2190. Found 252.2188.

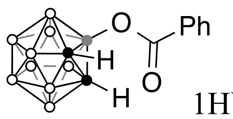

18: Colorless oil. Yield: 81%. ^{1}H NMR (400 MHz, CD_2Cl_2): δ 7.74 (m, 2H), 7.65 (m, 1H), 7.47 (m, 2H) (aromatic C*H*), 4.70 (br, 2H) (cage C*H*). $^{13}C\{^{1}H\}$ NMR (100 MHz, CD_2Cl_2): δ 162.0, 134.3, 132.1, 129.8, 127.2, 57.8 (cage *C*). $^{11}B\{^{1}H\}$ NMR (128 MHz, CD_2Cl_2): δ 0.4 (1B), -6.4 (2B), -12.7 (1B), -15.0 (2B), -15.7 (1B), -16.7 (2B), -21.4 (1B). HRMS (EI) calcd for $C_9H_{16}{}^{11}B_8{}^{10}B_2O_2{}^+$: 264.1638. Found 264.1635.

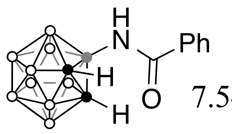

19b: White solid. Yield: 83%. ^{1}H NMR (400 MHz, CD_2Cl_2): δ 7.75 (d, *J* = 7.6 Hz, 2H), 7.54 (m, 1H), 7.44 (m, 1H) (aromatic C*H*), 4.81 (br, 2H) (cage C*H*). $^{13}C\{^{1}H\}$ NMR (100 MHz, CD_2Cl_2): δ 161.4, 132.9, 132.7, 129.1, 127.6, 127.5, 58.2 (cage *C*). $^{11}B\{^{1}H\}$ NMR (128 MHz, CD_2Cl_2): δ -4.3 (2B), -6.3 (1B), -10.0 (1B), -13.8 (6B). HRMS (EI) calcd for $C_9H_{17}{}^{11}B_8{}^{10}B_2ON^+$: 263.1790. Found 263.1788.

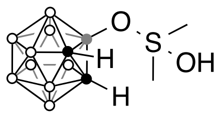

20: White solid. Yield: 95%. ^{1}H NMR (400 MHz, CD_2Cl_2): δ 5.43 (br, 1H) (O*H*), 3.70 (br, 2H) (cage C*H*), 2.61 (s, 6H) (CH_3). $^{13}C\{^1H\}$ NMR (100 MHz, CD_2Cl_2): δ 57.2 (cage *C*), 56.1, 41.0. $^{11}B\{^1H\}$ NMR (128 MHz, CD_2Cl_2): δ 0.5 (1B), -6.3 (2B), -12.7 (1B), -15.0 (2B), -15.6 (1B), -16.6 (2B), -21.3 (1B). HRMS (EI) calcd for $C_4H_{18}{}^{11}B_8{}^{10}B_2OS^+$: 222.1914. Found 222.1918.

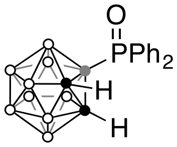

21a: White solid. Yield: 53%. ^{1}H NMR (400 MHz, acetone-d$_6$): δ 7.88 (m, 4H), 7.65-7.53 (m, 6H) (aromatic C*H*), 4.88 (br, 2H) (cage C*H*). $^{13}C\{^1H\}$ NMR (100 MHz, acetone-d$_6$): δ 136.2 (d, $J_{C\text{-}P}$ = 71.7 Hz), 132.4 (d, $J_{C\text{-}P}$ = 2.8 Hz), 131.1 (d, $J_{C\text{-}P}$ = 10.5 Hz), 128.5 (d, $J_{C\text{-}P}$ = 2.8 Hz), 46.6 (cage *C*). $^{11}B\{^1H\}$ NMR (128 MHz, acetone-d$_6$): δ -2.5 (3B), -8.6 (1B), -11.6 (2B), -12.3 (1B), -13.5 (3B). $^{31}P\{^1H\}$ NMR (162 MHz, acetone-d$_6$): δ 29.0 (br, 1P). HRMS (EI) calcd for $C_{14}H_{21}{}^{11}B_8{}^{10}B_2PO^+$: 344.2315. Found 344.2311.

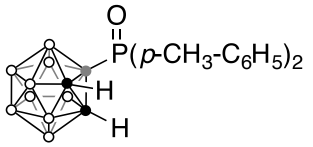

21b: White solid. Yield: 62%. ^{1}H NMR (400 MHz, CD_2Cl_2): δ 7.67 (m, 4H), 7.45 (m, 4H) (aromatic C*H*), 3.73 (br, 2H) (cage C*H*), 2.48 (br, 6H). $^{13}C\{^1H\}$ NMR (100 MHz, CD_2Cl_2): δ 146.9 (d, $J_{C\text{-}P}$ = 73.1 Hz), 132.5 (d, $J_{C\text{-}P}$ = 13.0 Hz), 130.9 (d, $J_{C\text{-}P}$ = 14.0 Hz), 130.6 (d, $J_{C\text{-}P}$ = 7.8 Hz), 57.1 (cage *C*), 22.1, 21. 9. $^{11}B\{^1H\}$ NMR (128 MHz, CD_2Cl_2): δ -2.2 (3B), -8.3 (1B), -11.3 (2B), -12.0 (1B), -13.2 (3B). $^{31}P\{^1H\}$ NMR (162 MHz, acetone-d$_6$): δ 33.9 (br, 1P). HRMS (EI) calcd for $C_{16}H_{25}{}^{11}B_8{}^{10}B_2PO^+$: 372.2847. Found 372.2846.

A 0.5 mmol scale reaction of carboranyl diazonium salt 1 with CsF.

Under an atmosphere of dry argon, dry CH_3CN (3 mL) was added to an open Schlenk flask (10 mL) containing 3-diazonium-*o*-carborane tetrafluoroborate (**1**; 129.0 mg, 0.5 mmol) and CsF (76.0 mg, 0.5 mmol). Bubbles were immediately observed at the surface of CsF. The reaction mixture was further stirred at room temperature for 60 min. The resulting mixture was examined by GC-MS analysis. After

removal of solvents in vacuo, the residue was examined by ^{1}H NMR spectrum and then subjected to flash column chromatography on Al_2O_3 (activated, neutral, Merck, 70-230 mesh mesh) using *n*-hexane/EtOAc (100/1 in V/V) as eluent to give **3a** as a white solid (66.8 mg, 83%).

Caution: For nucleophiles such as CsF that are poorly dissolved in CH_3CN, CH_3CN can be added to the mixture of diaoznium salt **1** and nucleophile at room temperature. For nucleophiles that have good solubility in CH_3CN, such as LiCl, we recommend that the nucleophiles should be added in small portion to a CH_3CN solution of **1** at a lower temperature (-30 ^oC) to avoid rapid nitrogen gas evolution.

Reaction of carboranyl diazonium salt 1 with ethers.

Reaction of carboranyl diazonium salt 1 with anhydrous diethyl ether. Under an atmosphere of dry argon, a suspension of 3-diazonium-*o*-carborane tetrafluoroborate (**1**; 25.8 mg, 0.1 mmol) in anhydrous diethyl ether (2 mL) in a 10 mL Schlenk flask was stirred at room temperature for 24 h. No bubble was observed during this period. Then the reaction was stopped and diethyl ether was removed in vacuo. The resulting residue was redissolved in anhydrous acetonitrile-d_3 and examined by ^{1}H NMR spectrum. The results show that precursor **1** was recovered (> 99%, eq S2).

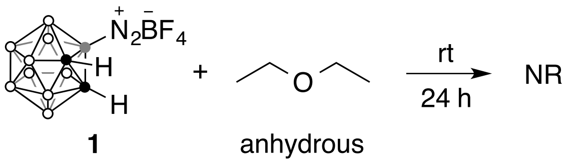

(S2)

Reaction of carboranyl diazonium salt 1 with wet diethyl ether. Under an atmosphere of dry argon, to a 10 mL Schlenk flask containing 3-diazonium-*o*-carborane tetrafluoroborate (**1**; 25.8 mg, 0.1 mmol) was added wet diethyl ether (2 mL, containing ~5% water) at room temperature with stirring. Bubbles were observed immediately. After stirring at room temperature for 1 min, the reaction was stopped and the solvents was removed in vacuo. The resulting residue was examined by GC-MS analysis and ^{1}H NMR spectrum. Then the crude reaction mixture was subjected to flash column chromatography

on Al_2O_3 (active, neutral, Merck, 70-230 mesh mesh) using *n*-hexane/EtOAc (100/1 in v/v) as eluent to give **13** as the only product (17.9 mg, 95%). No 3-hydroxyl-*o*-$C_2B_{10}H_{11}$ **17g** was detected (eq S4).

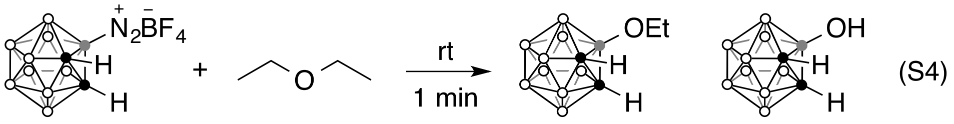

Reaction of carboranyl diazonium salt 1 with anhydrous THF. Under an atmosphere of dry argon, a solution of 3-diazonium-*o*-carborane tetrafluoroborate (**1**; 25.8 mg, 0.1 mmol) in anhydrous THF (2 mL) in a 10 mL Schlenk flask was stirred at room temperature. Gelation occurred in 10 min with the observation of gas bubbles (eq S3 and Figure S3).

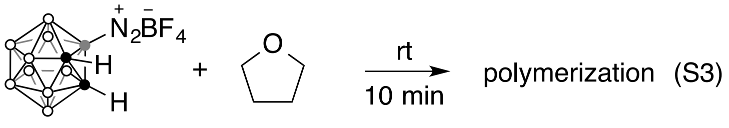

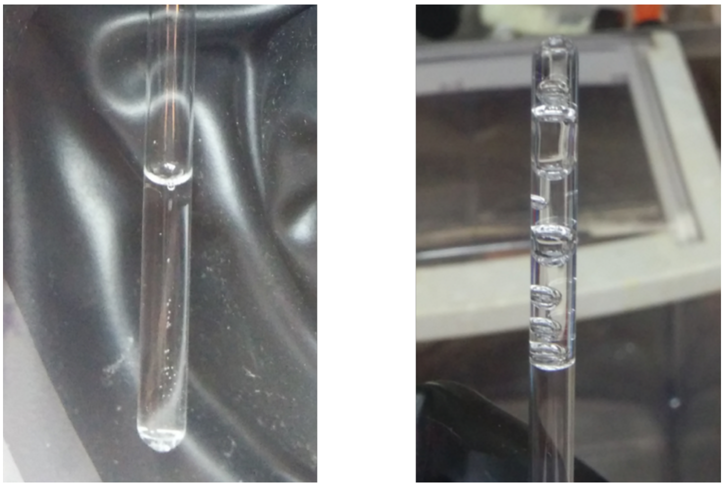

Figure S4. THF solution of **1**: gradually bubbles (t = 30s, left) and gel formation (t = 10 min, right).

Rreaction of carboranyl diazonium salt 1 with wet *tert*-butyl methyl ether. Under an atmosphere of dry argon, to a 10 mL Schlenk flask containing 3-diazonium-*o*-carborane tetrafluoroborate (**1**; 25.8 mg, 0.1 mmol) was added wet *tert*-butyl methyl ether (2 mL, containing ~5% water) at room temperature with stirring. Bubbles were observed immediately. After stirring at room temperature for 1 min, the reaction

was stopped and the solvents was removed in vacuo. The resulting residue was examined by GC-MS analysis and ^{1}H NMR spectrum. Then the crude reaction mixture was subjected to flash column chromatography on Al_2O_3 (active, neutral, Merck, 70-230 mesh mesh) using *n*-hexane/EtOAc (100/1 in v/v) as eluent to give **17a** as the only product (17.4 mg, quant). No 3-O^{t}Butyl-*o*-$C_2B_{10}H_{11}$ **17d** was detected (eq S5).

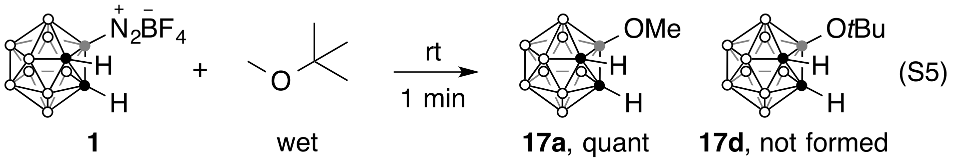

Formation of HBF_4 in the reaction of carboranyl diazonium salt 1 with methanol. The formation of HBF_4 was indicated by comparing the crude ^{11}B and ^{19}F NMR spectra of the reaction mixture with the CH_3CN solution of HBF_4 (48 wt. % in H_2O) as external standard (Figures S5 and S6).

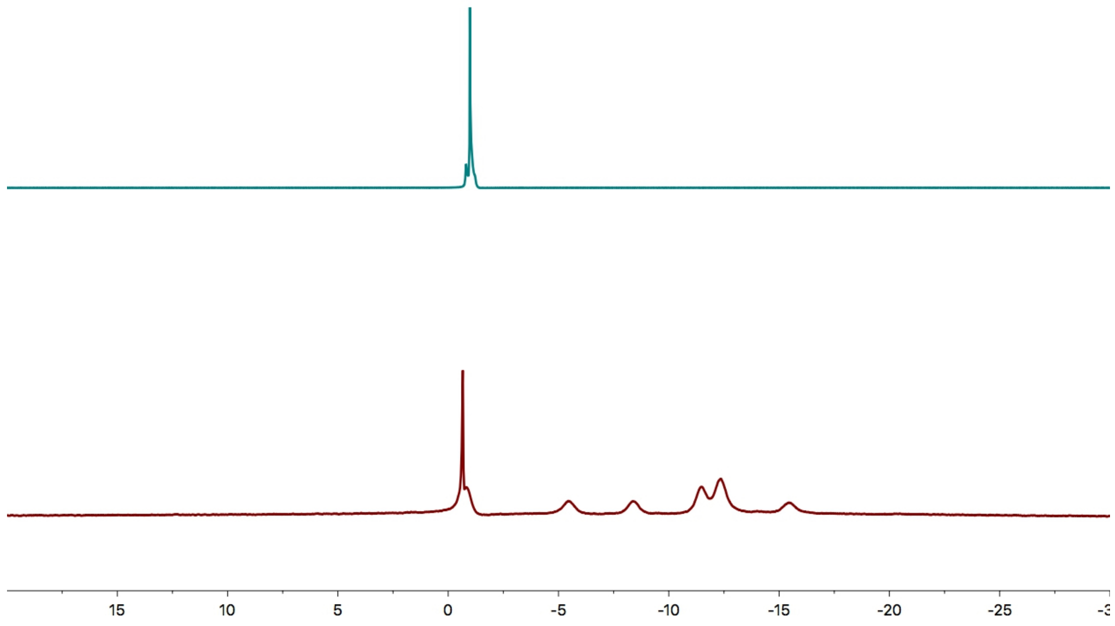

Figure S5. ^{11}B NMR spectra of HBF_4 (48 wt. % in H_2O) in CH_3CN (top) and the crude reaction mixture from the reaction of precursor **1** with methanol in CH_3CN (bottom).

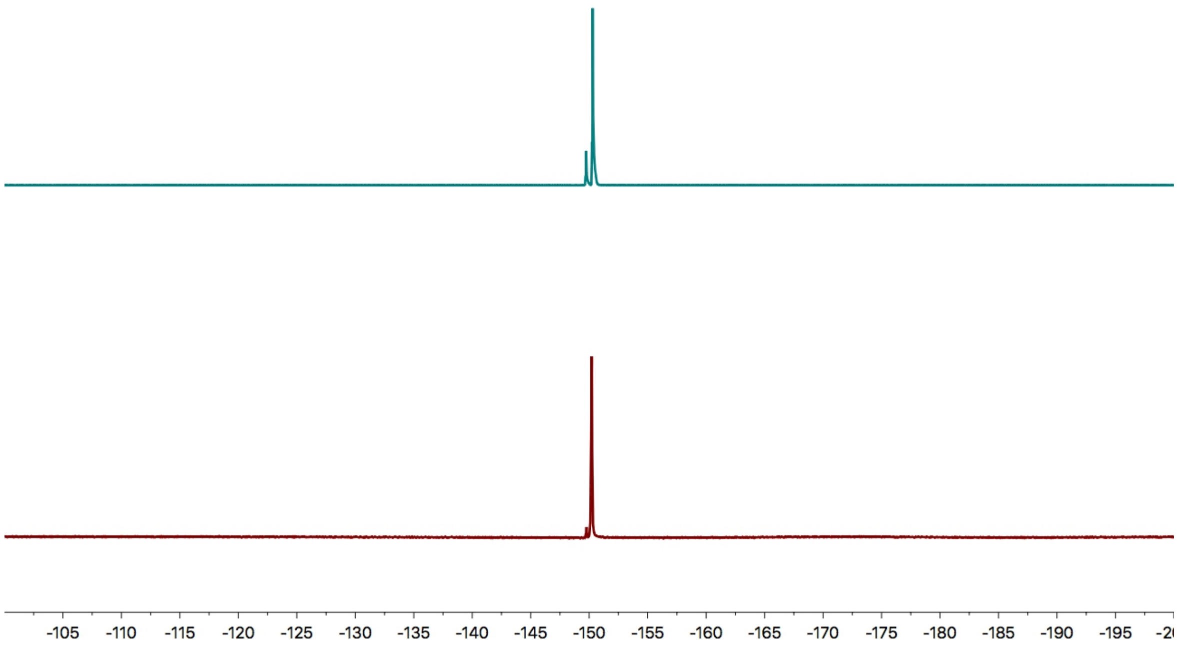

Figure S6. ^{19}F NMR spectra of HBF_4 (48 wt. % in H_2O) in CH_3CN (top) and the crude reaction mixture from the reaction of precursor **1** with methanol in CH_3CN (bottom).

Proof-of-concept synthesis of 3-fluoro-*o*-carborane under reported reaction conditions.[8] Under an atmosphere of dry argon, to a 5 mL Schlenk flask containing 3-diazonium-*o*-carborane tetrafluoroborate (**1**; 5.8 mg, 0.02 mmol), CsF (3.4 mg, 0.02 mmol), Kryptofix 222 (8.4 mg, 0.02 mmol) was added anhydrous CH_3CN (1 mL) at room temperature with stirring. Bubbles were observed immediately and ceased in 1 min. Then the reaction was stopped and the solvent was removed in vacuo. The resulting residue was examined by GC-MS analysis and ^{1}H NMR spectrum. Then the crude reaction mixture was dissolved in diethyl ether and quickly passed through a short column of silica gel (230-400 mesh) to remove the inorganic salt. Removal of the solvent afforded 3-fluoro-*o*-carborane **3a** as white solid (3.1 mg, 98%) (eq S6).

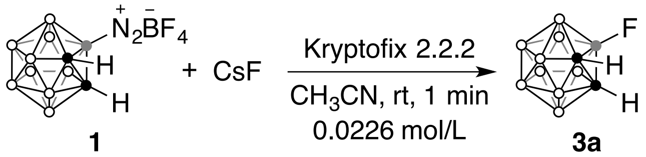

(S6)

X-ray structure determination. All data were collected at 293 K on a Bruker SMART 1000 CCD diffractometer using Mo-K_α radiation. An empirical absorption correction was applied using the SADABS program.[9] All structures were solved by direct methods and subsequent Fourier difference techniques and refined anisotropically for all non-hydrogen atoms by full-matrix least squares calculations on F^2 using the SHELXTL program package.[10] All hydrogen atoms were geometrically fixed using the riding model.

CCDC 1473011 (**4**) and 1473012 (**6**) contain the supplementary crystallographic data for this paper. These data can be obtained free of charge from The Cambridge Crystallographic Data Centre via www.ccdc.cam.ac.uk/data_request/cif.

References

(1) D. Zhao, J. Zhang and Z. Xie, *Angew. Chem., Int. Ed.,* **2014***, 53,* 8488–8491.

(2) J. S. Roscoe, S. Kongpricha and S. Papetti, *Inorg. Chem.*, 1970, **9**, 1561–1563.

(3) L. I. Zakharkin, V. N. Kalinin and V. V. Gedymin, *J. Organomet. Chem.*, 1969, **16**, 371–379.

(4) J. Li and M. Jones, Jr, *Inorg. Chem.*, 1990, **29**, 4162–4163.

(5) J. Li, C. F. Logan and M. Jones, Jr, *Inorg. Chem.*, 1991, **30**, 4866–4868.

(6) Y, Sevryugina, R. L. Julius and M. F. Hawthorne, *Inorg. Chem.*, 2010, **49**, 10627–10634.

(7) K. Aizawa, K. Ohta and Y. Endo, *Inorg. Chem.*, 2001, **40**, 6555–6562.

(8) K. B. Gona, V. Gómez-Vallejo, D. Padro and J. Llop, *Chem. Commun.*, 2013, **49**, 11491−11493.

(9) G. M. Sheldrick, SADABS: Program for Empirical Absorption Correction of Area Detector Data. University of Göttingen, Germany, 1996.

(10) G. M. Sheldrick, SHELXTL 5.10 for Windows NT: Structure Determination Software Programs. Bruker Analytical X-ray Systems, Inc., Madison, Wisconsin, USA, 1997.

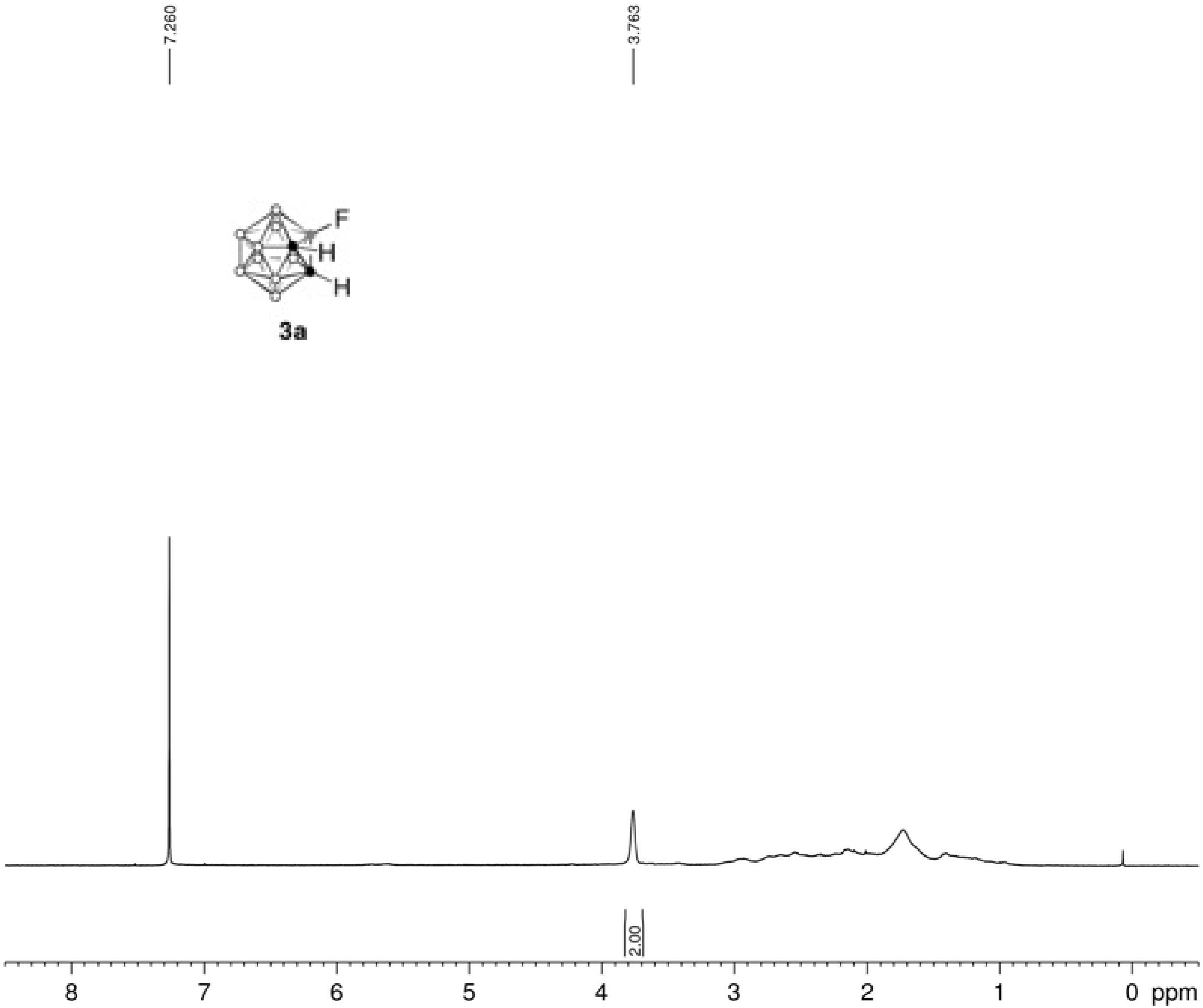

3a

ZD-7375-CDCl3-H

Current Data Parameters
NAME ZD-7375-CDCl3-H
EXPNO 2
PROCNO 1

F2 - Acquisition Parameters
Date_ 20151112
Time 14.01 h
INSTRUM spect
PROBHD Z108618_0257 (
PULPROG zg30
TD 65536
SOLVENT CDCl3
NS 8
DS 2
SWH 8012.820 Hz
FIDRES 0.122266 Hz
AQ 4.0894465 sec
RG 144
DW 62.400 usec
DE 6.50 usec
TE 294.5 K
D1 1.00000000 sec
TD0 1
SFO1 400.2324714 MHz
NUC1 1H
P1 12.80 usec
PLW1 13.56000042 W

F2 - Processing parameters
SI 65536
SF 400.2300101 MHz
WDW EM
SSB 0
LB 0.30 Hz
GB 0
PC 1.00

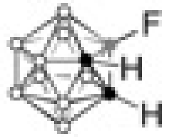

3a

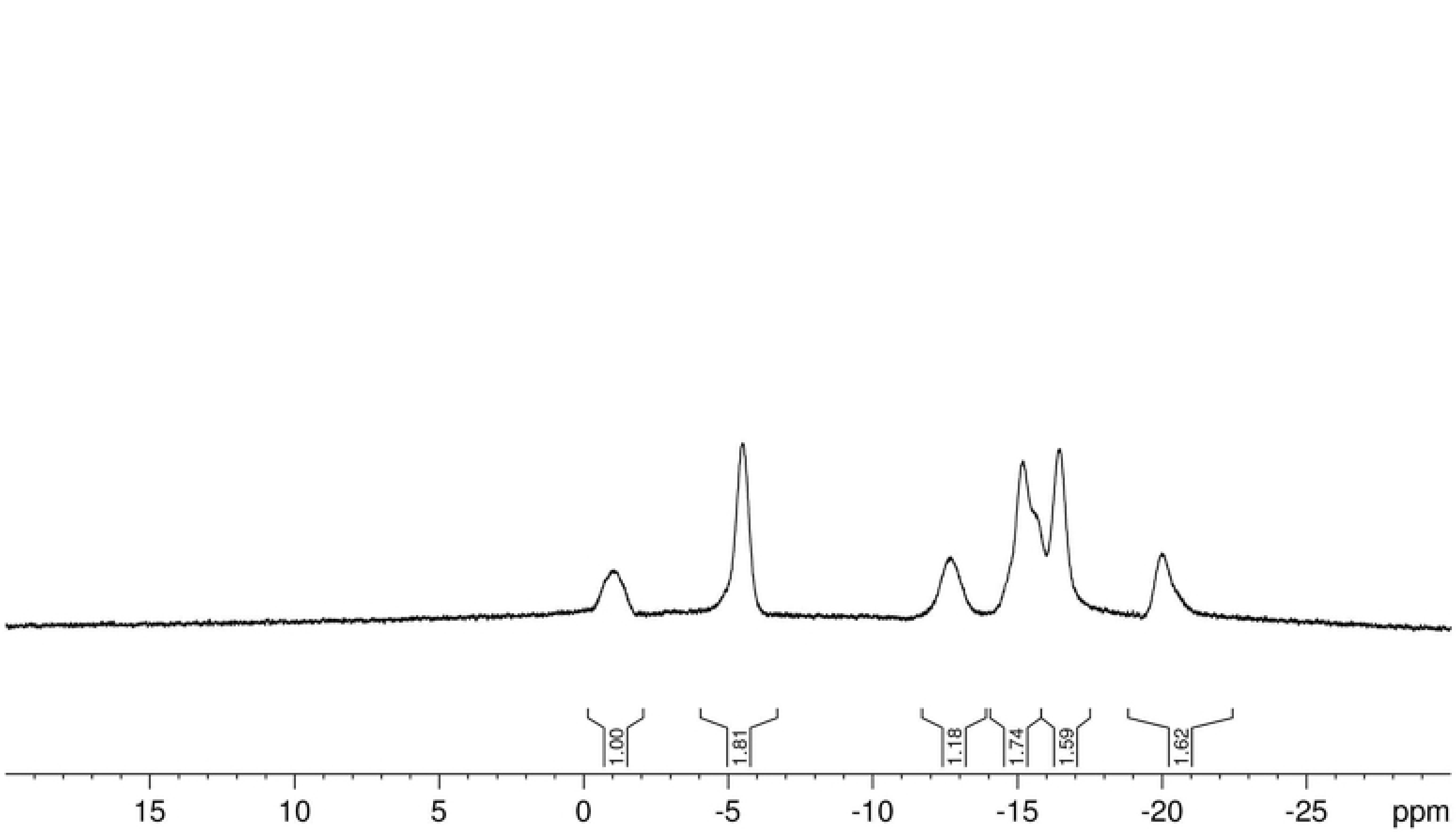

ZD-7375-CDCl3-B(de)

Current Data Parameters
NAME ZD-7375-CDCl3-B(de)
EXPNO 1
PROCNO 1

F2 - Acquisition Parameters
Date_ 20151112
Time 14.04 h
INSTRUM spect
PROBHD Z108618_0257 (
PULPROG zgdc
TD 65536
SOLVENT CDCl3
NS 13
DS 4
SWH 24038.461 Hz
FIDRES 0.366798 Hz
AQ 1.3631488 sec
RG 362
DW 20.800 usec
DE 6.50 usec
TE 295.1 K
D1 2.00000000 sec
D11 0.03000000 sec
TD0 1
SFO1 128.4096890 MHz
NUC1 11B
P1 7.50 usec
PLW1 55.09999847 W
SFO2 400.2316009 MHz
NUC2 1H
CPDPRG[2 waltz16
PCPD2 90.00 usec
PLW2 13.56000042 W
PLW12 0.27428001 W

F2 - Processing parameters
SI 32768
SF 128.4097615 MHz
WDW EM
SSB 0
LB 1.00 Hz
GB 0
PC 1.40

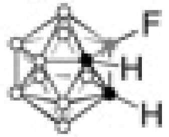

3a

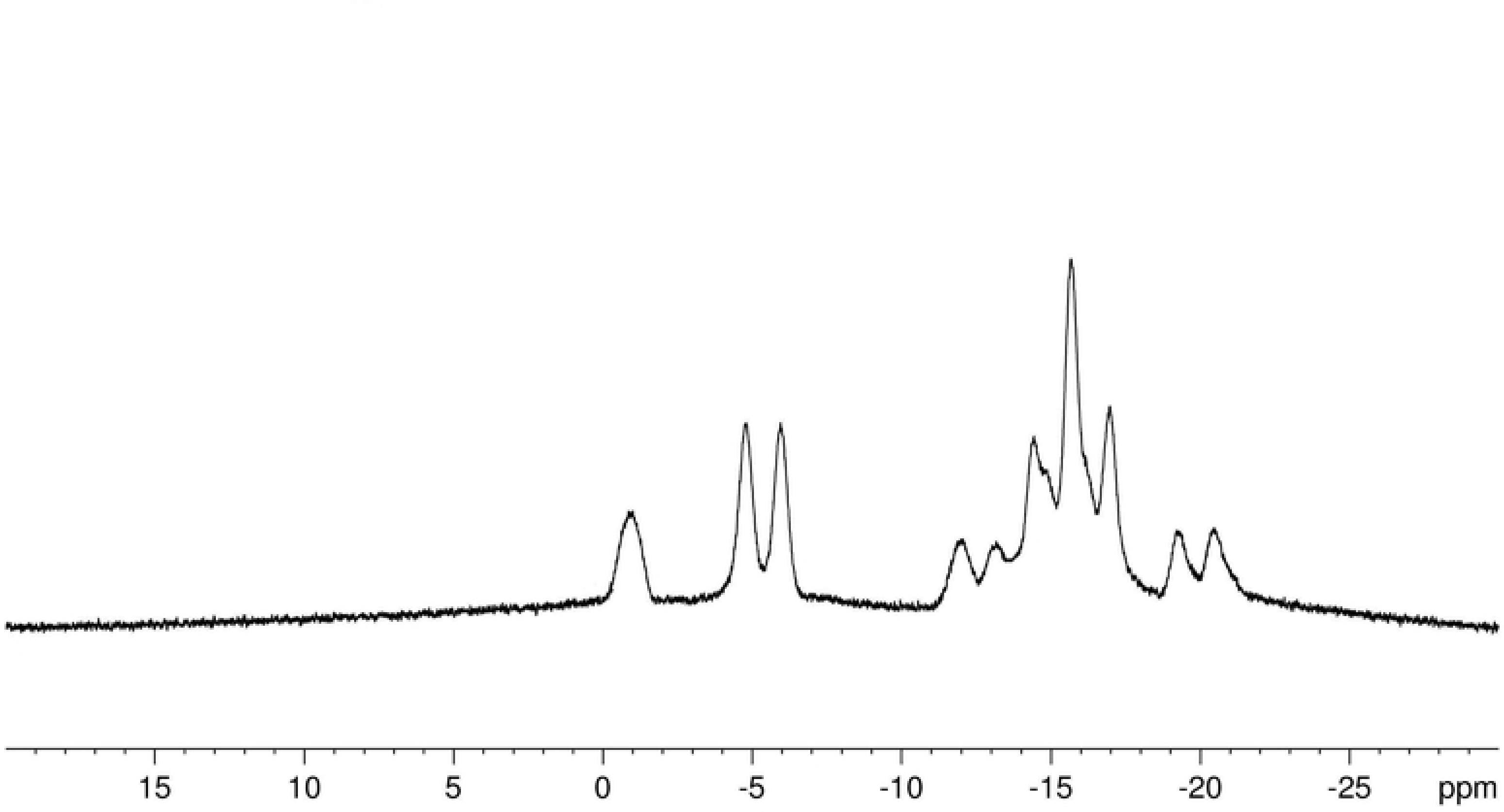

ZD-7375-CDCl3-B(c)

```
Current Data Parameters
NAME      ZD-7375-CDCl3-B(c)
EXPNO                  1
PROCNO                 1

F2 - Acquisition Parameters
Date_           20151112
Time               14.05 h
INSTRUM            spect
PROBHD    Z108618_0257 (
PULPROG               zg
TD                 65536
SOLVENT            CDCl3
NS                    18
DS                     2
SWH            24038.461 Hz
FIDRES          0.366798 Hz
AQ             1.3631488 sec
RG                   101
DW                20.800 usec
DE                  6.50 usec
TE                 294.8 K
D1            2.00000000 sec
TD0                    1
SFO1         128.4096890 MHz
NUC1                 11B
P1                  7.50 usec
PLW1         55.09999847 W

F2 - Processing parameters
SI                 32768
SF           128.4097430 MHz
WDW                   EM
SSB       0
LB                  1.00 Hz
GB        0
PC                  1.40
```

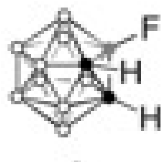

3a

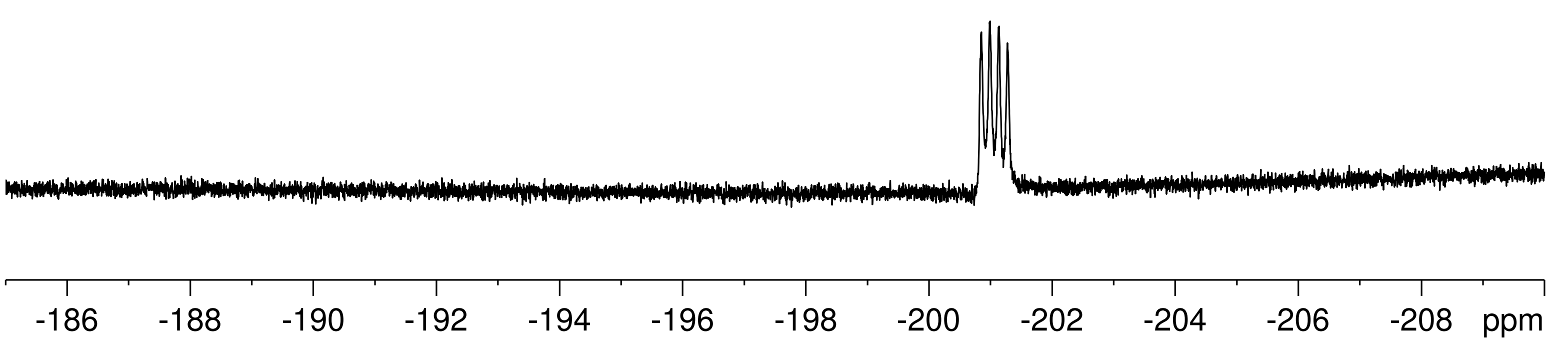

ZD-7375-CDCl3-F

Current Data Parameters
NAME ZD-7375-CDCl3-F
EXPNO 1
PROCNO 1

F2 - Acquisition Parameters
Date_ 20151112
Time 14.07 h
INSTRUM spect
PROBHD Z108618_0257 (
PULPROG zgfhigqn.2
TD 131072
SOLVENT CDCl3
NS 32
DS 4
SWH 89285.711 Hz
FIDRES 0.681196 Hz
AQ 0.7340032 sec
RG 645
DW 5.600 usec
DE 6.50 usec
TE 294.9 K
D1 1.00000000 sec
D11 0.03000000 sec
D12 0.00002000 sec
TD0 1
SFO1 376.5548010 MHz
NUC1 19F
P1 14.70 usec
PLW1 18.36000061 W
SFO2 400.2316009 MHz
NUC2 1H
CPDPRG[2 waltz16
PCPD2 90.00 usec
PLW2 13.56000042 W
PLW12 0.27428001 W

F2 - Processing parameters
SI 65536
SF 376.5924602 MHz
WDW EM
SSB 0
LB 0.30 Hz
GB 0
PC 1.00

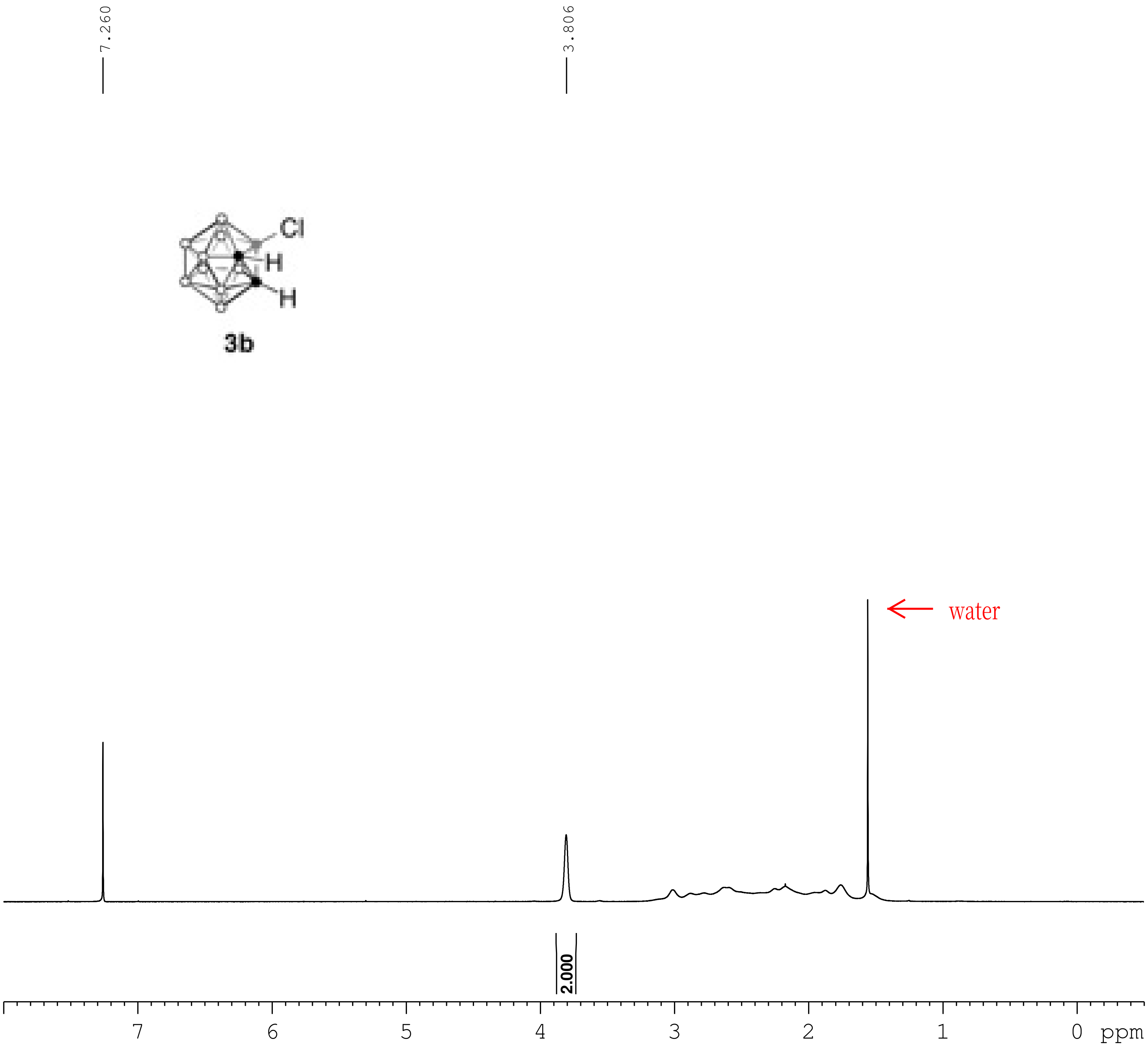

ZD-3-Cl-CDCl3-H

Current Data Parameters
NAME ZD-3-Cl-CDCl3-H
EXPNO 1
PROCNO 1

F2 - Acquisition Parameters
Date_ 20160126
Time 21.34 h
INSTRUM spect
PROBHD Z824601_0021 (
PULPROG zg
TD 65536
SOLVENT CDCl3
NS 8
DS 0
SWH 8012.820 Hz
FIDRES 0.122266 Hz
AQ 4.0894465 sec
RG 203
DW 62.400 usec
DE 6.50 usec
TE 294.1 K
D1 1.00000000 sec
TD0 1
SFO1 400.1316005 MHz
NUC1 1H
P1 15.00 usec
PLW1 8.31000042 W

F2 - Processing parameters
SI 65536
SF 400.1300099 MHz
WDW EM
SSB 0
LB 0.30 Hz
GB 0
PC 1.00

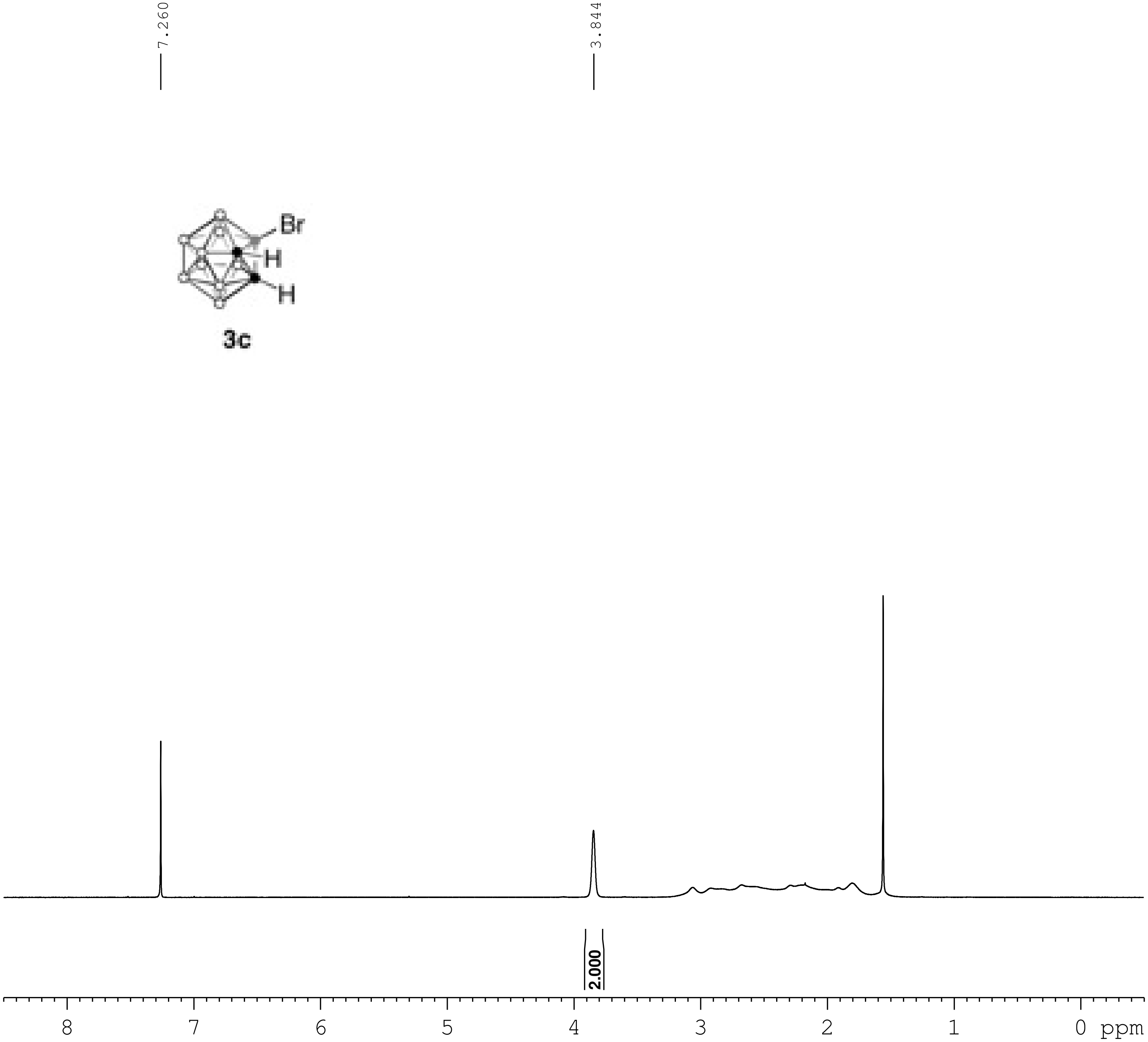

ZD-3-Br-CDCl3-H

```
Current Data Parameters
NAME     ZD-3-Br-CDCl3-H
EXPNO                  1
PROCNO                 1

F2 - Acquisition Parameters
Date_          20160126
Time              21.39 h
INSTRUM           spect
PROBHD   Z824601_0021 (
PULPROG              zg
TD                65536
SOLVENT           CDCl3
NS                    8
DS                    0
SWH            8012.820 Hz
FIDRES         0.122266 Hz
AQ            4.0894465 sec
RG                  203
DW               62.400 usec
DE                 6.50 usec
TE                294.2 K
D1           1.00000000 sec
TD0                   1
SFO1        400.1316005 MHz
NUC1                 1H
P1                15.00 usec
PLW1         8.31000042 W

F2 - Processing parameters
SI                65536
SF          400.1300098 MHz
WDW                  EM
SSB      0
LB                 0.30 Hz
GB       0
PC                 1.00
```

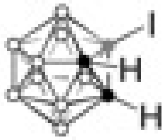

3d

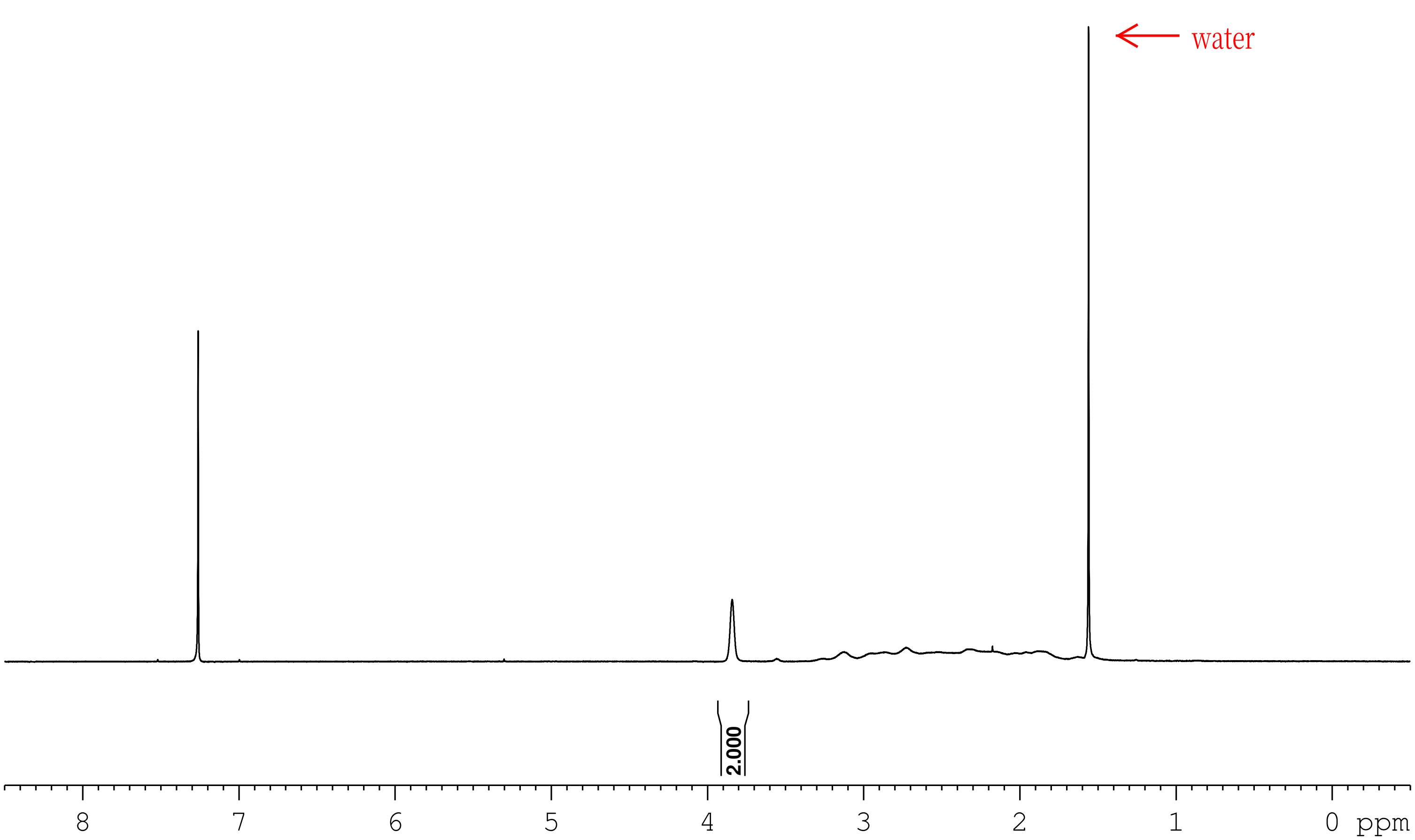

ZD-3-I-CDCl3-H

Current Data Parameters
NAME ZD-3-I-CDCl3-H
EXPNO 1
PROCNO 1

F2 - Acquisition Parameters
Date_ 20160126
Time 21.43 h
INSTRUM spect
PROBHD Z824601_0021 (
PULPROG zg
TD 65536
SOLVENT CDCl3
NS 8
DS 0
SWH 8012.820 Hz
FIDRES 0.122266 Hz
AQ 4.0894465 sec
RG 203
DW 62.400 usec
DE 6.50 usec
TE 294.2 K
D1 1.00000000 sec
TD0 1
SFO1 400.1316005 MHz
NUC1 1H
P1 15.00 usec
PLW1 8.31000042 W

F2 - Processing parameters
SI 65536
SF 400.1300098 MHz
WDW EM
SSB 0
LB 0.30 Hz
GB 0
PC 1.00

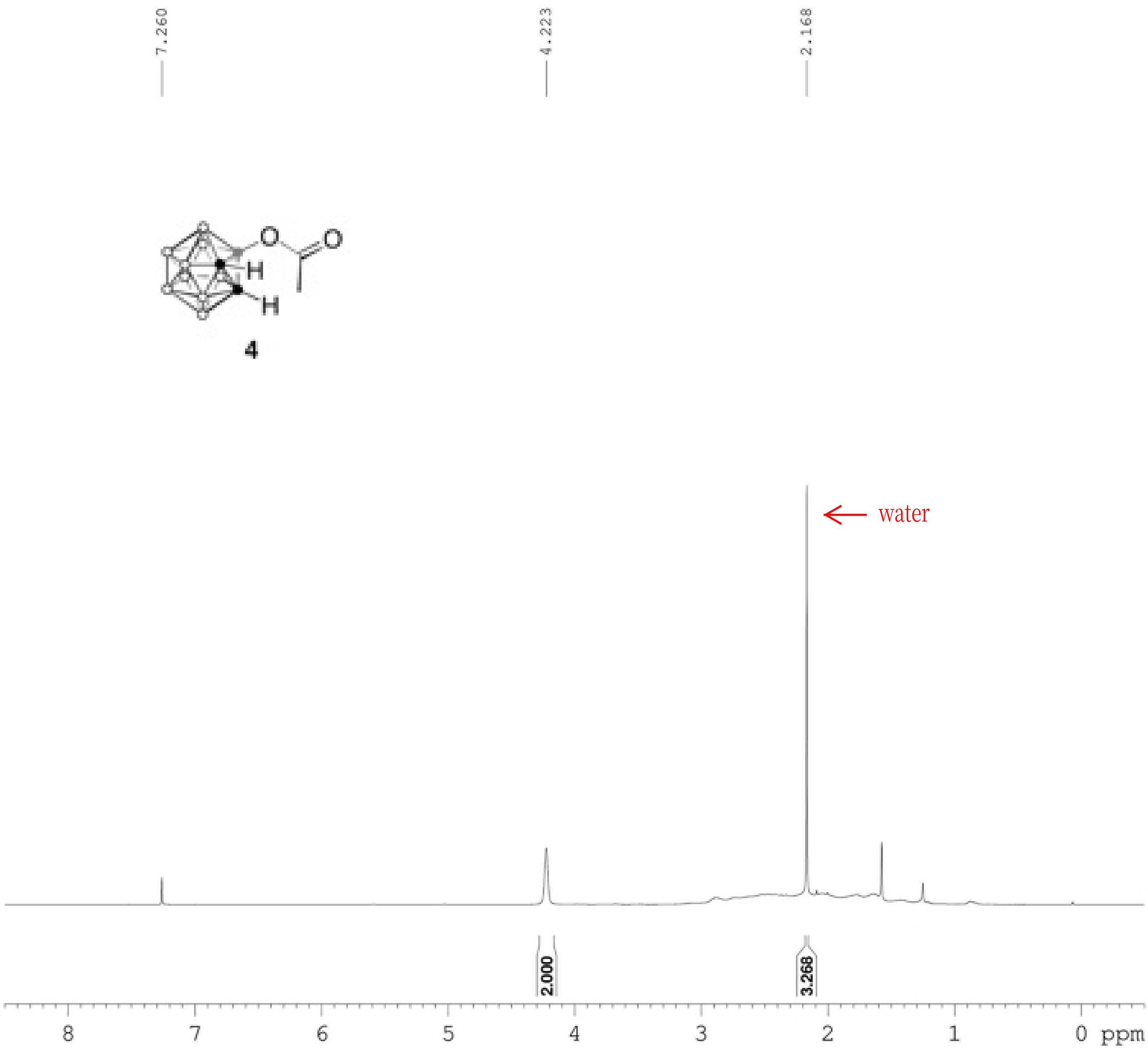

4

NAME	ZD-770-CDCl3-H
EXPNO	1
PROCNO	1
Date_	20140916
Time	19.40
INSTRUM	spect
PROBHD	5 mm PADUL 13C
PULPROG	zg
TD	65536
SOLVENT	CDCl3
NS	16
DS	0
SWH	10000.000 Hz
FIDRES	0.152588 Hz
AQ	3.2768500 sec
RG	144
DW	50.000 usec
DE	6.50 usec
TE	294.4 K
D1	1.00000000 sec
TD0	1
======== CHANNEL f1 ========	
NUC1	1H
P1	14.83 usec
PL1	0.00 dB
PL1W	8.31434441 W
SFO1	400.1318000 MHz
SI	65536
SF	400.1300083 MHz
WDW	EM
SSB	0
LB	0.30 Hz
GB	0
PC	1.00

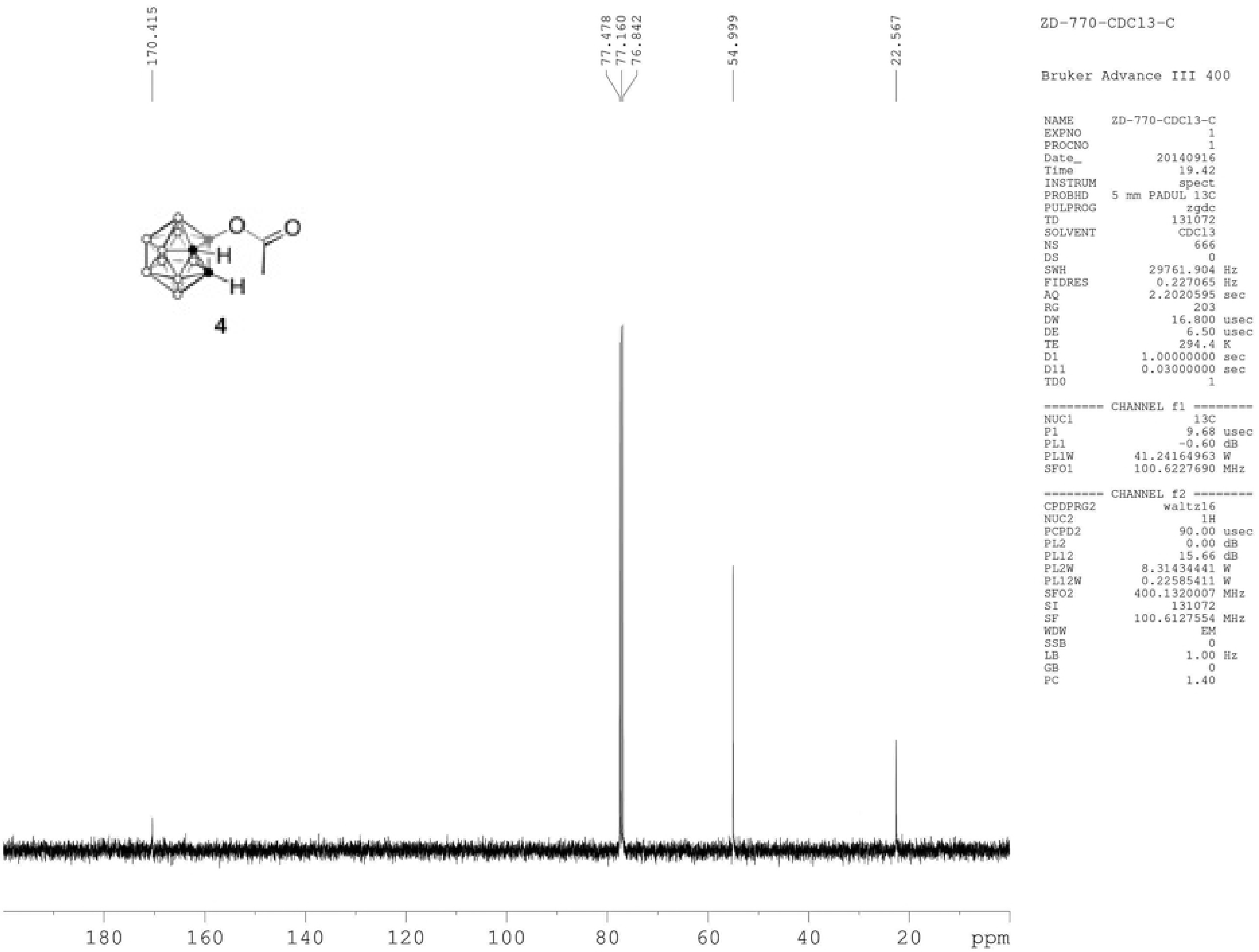
170.415
77.478
77.160
76.842
54.999
22.567
H
H
O
O
4
ZD-770-CDCl3-C
Bruker Advance III 400
NAME ZD-770-CDCl3-C
EXPNO 1
PROCNO 1
Date_ 20140916
Time 19.42
INSTRUM spect
PROBHD 5 mm PADUL 13C
PULPROG zgdc
TD 131072
SOLVENT CDCl3
NS 666
DS 0
SWH 29761.904 Hz
FIDRES 0.227065 Hz
AQ 2.2020595 sec
RG 203
DW 16.800 usec
DE 6.50 usec
TE 294.4 K
D1 1.00000000 sec
D11 0.03000000 sec
TD0 1
======== CHANNEL f1 ========
NUC1 13C
P1 9.68 usec
PL1 -0.60 dB
PL1W 41.24164963 W
SFO1 100.6227690 MHz
======== CHANNEL f2 ========
CPDPRG2 waltz16
NUC2 1H
PCPD2 90.00 usec
PL2 0.00 dB
PL12 15.66 dB
PL2W 8.31434441 W
PL12W 0.22585411 W
SFO2 400.1320007 MHz
SI 131072
SF 100.6127554 MHz
WDW EM
SSB 0
LB 1.00 Hz
GB 0
PC 1.40
180
160
140
120
100
80
60
40
20
ppm

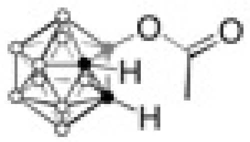

4

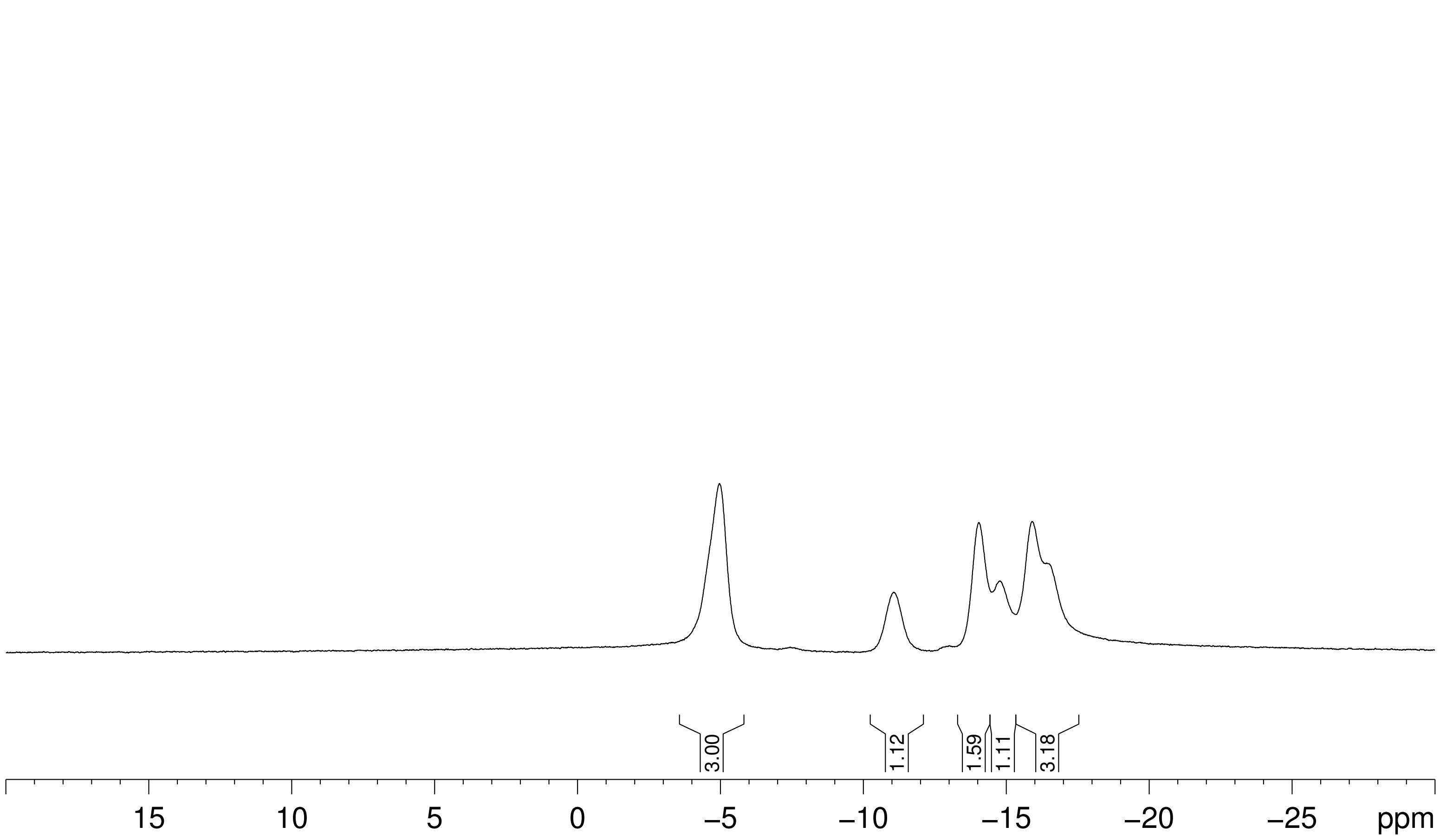

ZD-770-CDCl3-B(de)

NAME	ZD-770-CDCl3-B(de)
EXPNO	1
PROCNO	1
Date_	20140916
Time	20.29
INSTRUM	spect
PROBHD	5 mm PABBO BB-
PULPROG	zgdc
TD	65536
SOLVENT	CDCl3
NS	4
DS	0
SWH	25510.203 Hz
FIDRES	0.389255 Hz
AQ	1.2845556 sec
RG	287
DW	19.600 usec
DE	6.50 usec
TE	295.8 K
D1	5.00000000 sec
D11	0.03000000 sec
TD0	1
======== CHANNEL f1 ========	
NUC1	11B
P1	7.60 usec
PL1	-3.00 dB
PL1W	55.13059616 W
SFO1	128.3968556 MHz
======== CHANNEL f2 ========	
CPDPRG2	waltz16
NUC2	1H
PCPD2	90.00 usec
PL2	-1.00 dB
PL12	15.16 dB
PL2W	13.56617069 W
PL12W	0.32844096 W
SFO2	400.1916008 MHz
SI	32768
SF	128.3968847 MHz
WDW	EM
SSB	0
LB	3.00 Hz
GB	0
PC	1.40

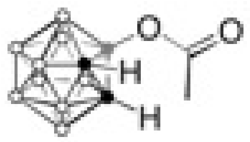

4

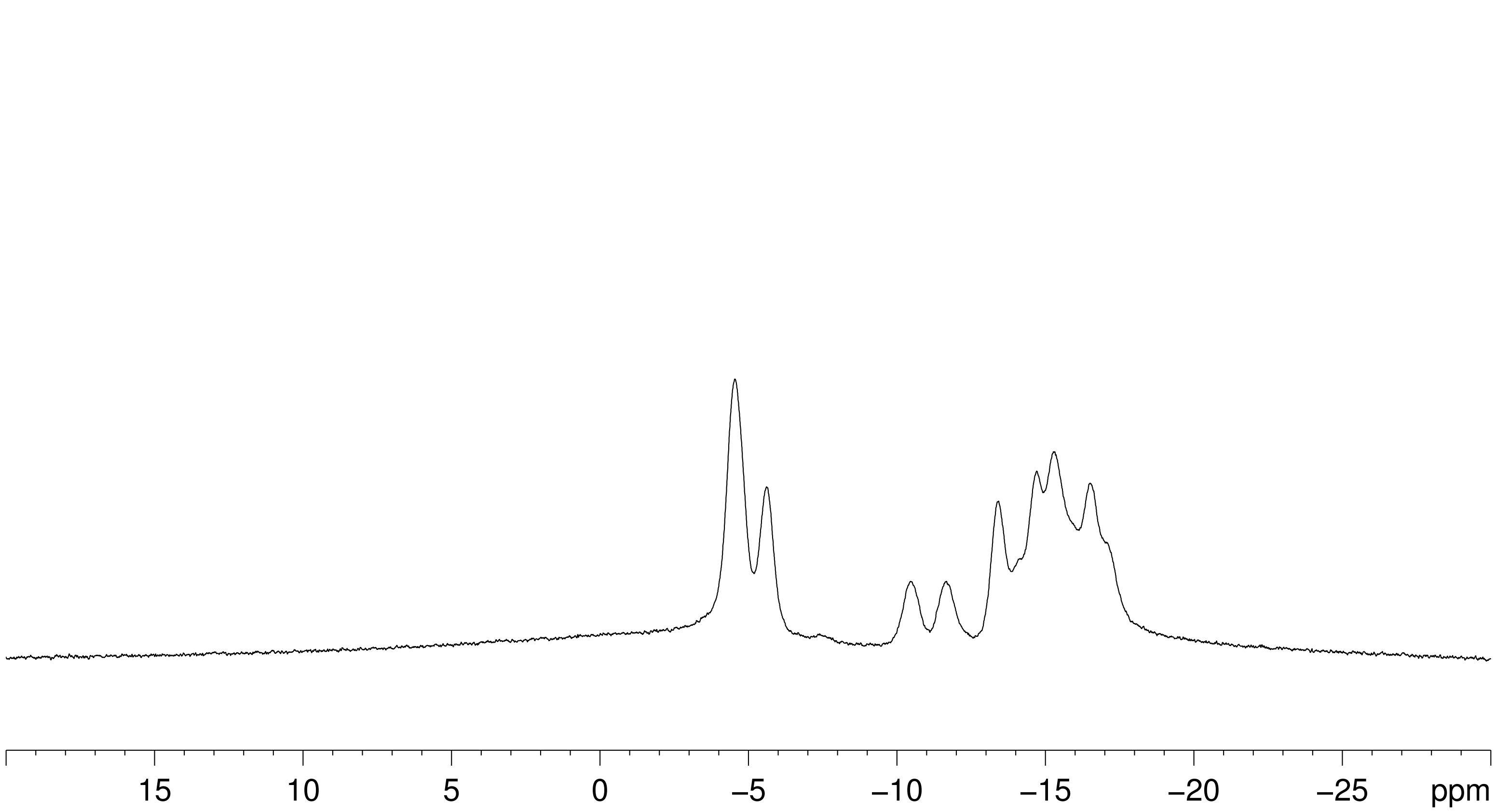

ZD-770-CDCl3-B(c)

NAME	ZD-770-CDCl3-B(c)
EXPNO	1
PROCNO	1
Date_	20140916
Time	20.30
INSTRUM	spect
PROBHD	5 mm PABBO BB-
PULPROG	zg30
TD	65536
SOLVENT	CDCl3
NS	8
DS	0
SWH	25510.203 Hz
FIDRES	0.389255 Hz
AQ	1.2845556 sec
RG	161
DW	19.600 usec
DE	6.50 usec
TE	295.5 K
D1	5.00000000 sec
TD0	1

======== CHANNEL f1 ========

NUC1	11B
P1	7.60 usec
PL1	-3.00 dB
PL1W	55.13059616 W
SFO1	128.3968556 MHz
SI	32768
SF	128.3968865 MHz
WDW	EM
SSB	0
LB	3.00 Hz
GB	0
PC	1.40

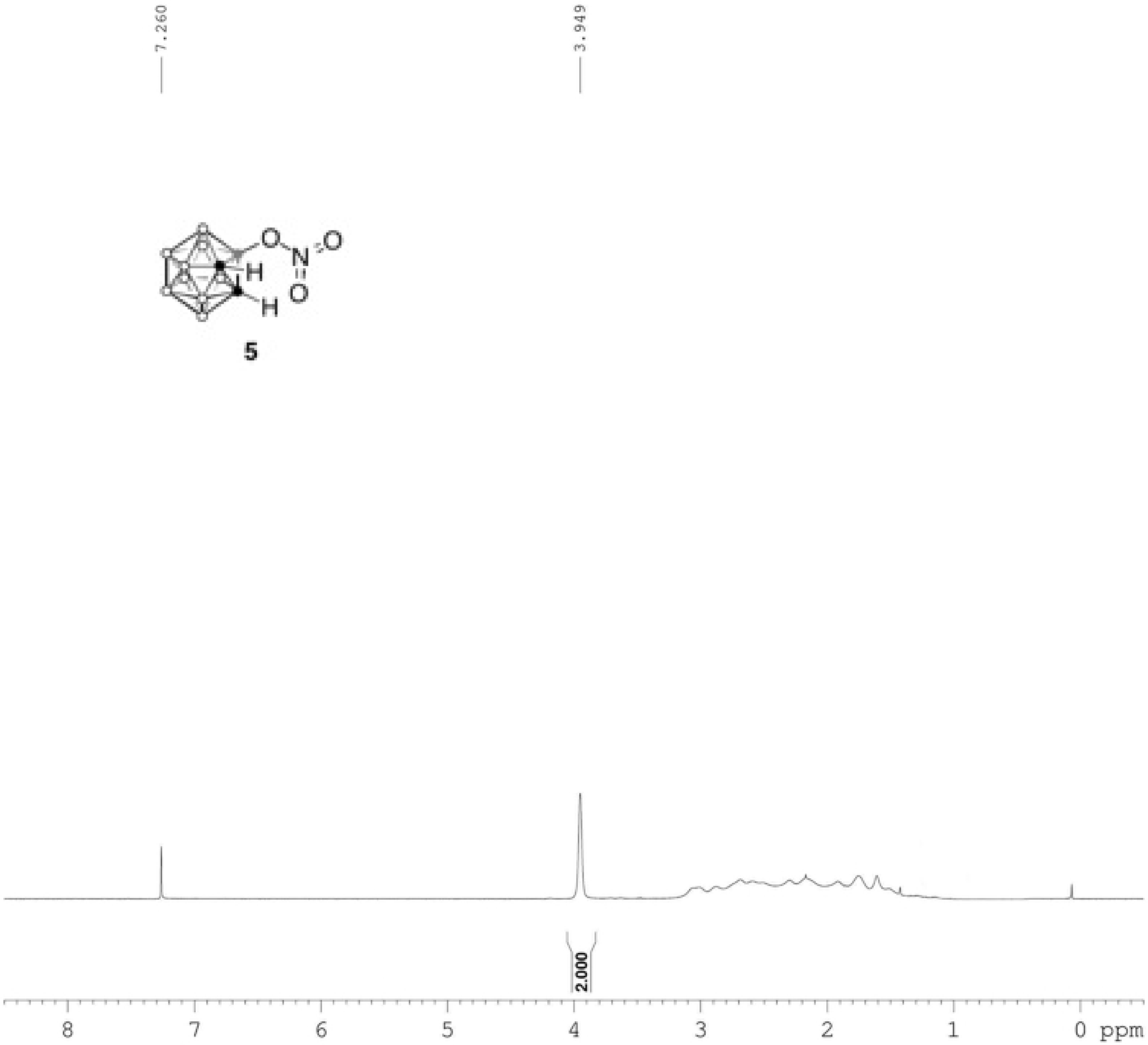

ZD-777-1-CDCl3-H

Bruker Advance III 400

NAME	ZD-777-1-CDCl3-H	
EXPNO	1	
PROCNO	1	
Date_	20140917	
Time	16.38	
INSTRUM	spect	
PROBHD	5 mm PADUL 13C	
PULPROG	zg	
TD	65536	
SOLVENT	CDCl3	
NS	4	
DS	0	
SWH	10000.000	Hz
FIDRES	0.152588	Hz
AQ	3.2768500	sec
RG	144	
DW	50.000	usec
DE	6.50	usec
TE	294.3	K
D1	1.00000000	sec
TD0	1	

======== CHANNEL f1 ========

NUC1	1H	
P1	14.83	usec
PL1	0.00	dB
PL1W	8.31434441	W
SFO1	400.1318000	MHz
SI	65536	
SF	400.1300082	MHz
WDW	EM	
SSB	0	
LB	0.30	Hz
GB	0	
PC	1.00	

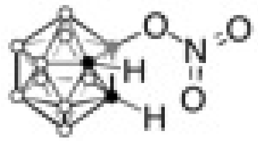

5

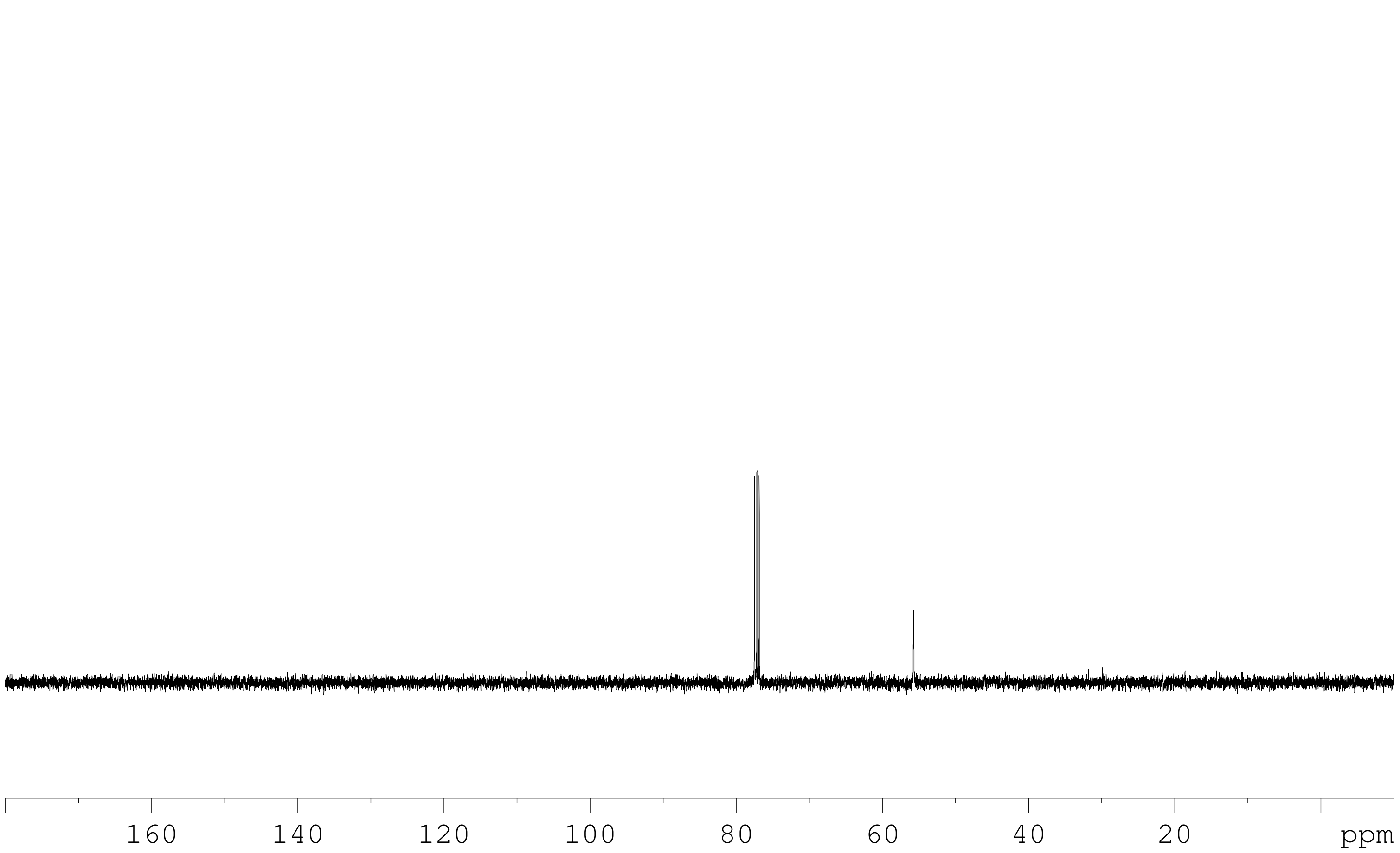

ZD-777-1-CDCl3-C

Bruker Advance III 400

NAME	ZD-777-1-CDCl3-C	
EXPNO	1	
PROCNO	1	
Date_	20140917	
Time	16.42	
INSTRUM	spect	
PROBHD	5 mm PADUL 13C	
PULPROG	zgdc	
TD	131072	
SOLVENT	CDCl3	
NS	128	
DS	0	
SWH	29761.904	Hz
FIDRES	0.227065	Hz
AQ	2.2020595	sec
RG	203	
DW	16.800	usec
DE	6.50	usec
TE	294.3	K
D1	1.00000000	sec
D11	0.03000000	sec
TD0	1	

======== CHANNEL f1 ========

NUC1	13C	
P1	9.68	usec
PL1	-0.60	dB
PL1W	41.24164963	W
SFO1	100.6227690	MHz

======== CHANNEL f2 ========

CPDPRG2	waltz16	
NUC2	1H	
PCPD2	90.00	usec
PL2	0.00	dB
PL12	15.66	dB
PL2W	8.31434441	W
PL12W	0.22585411	W
SFO2	400.1320007	MHz
SI	131072	
SF	100.6127559	MHz
WDW	EM	
SSB	0	
LB	1.00	Hz
GB	0	
PC	1.40	

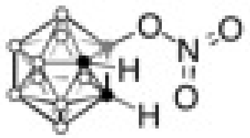

5

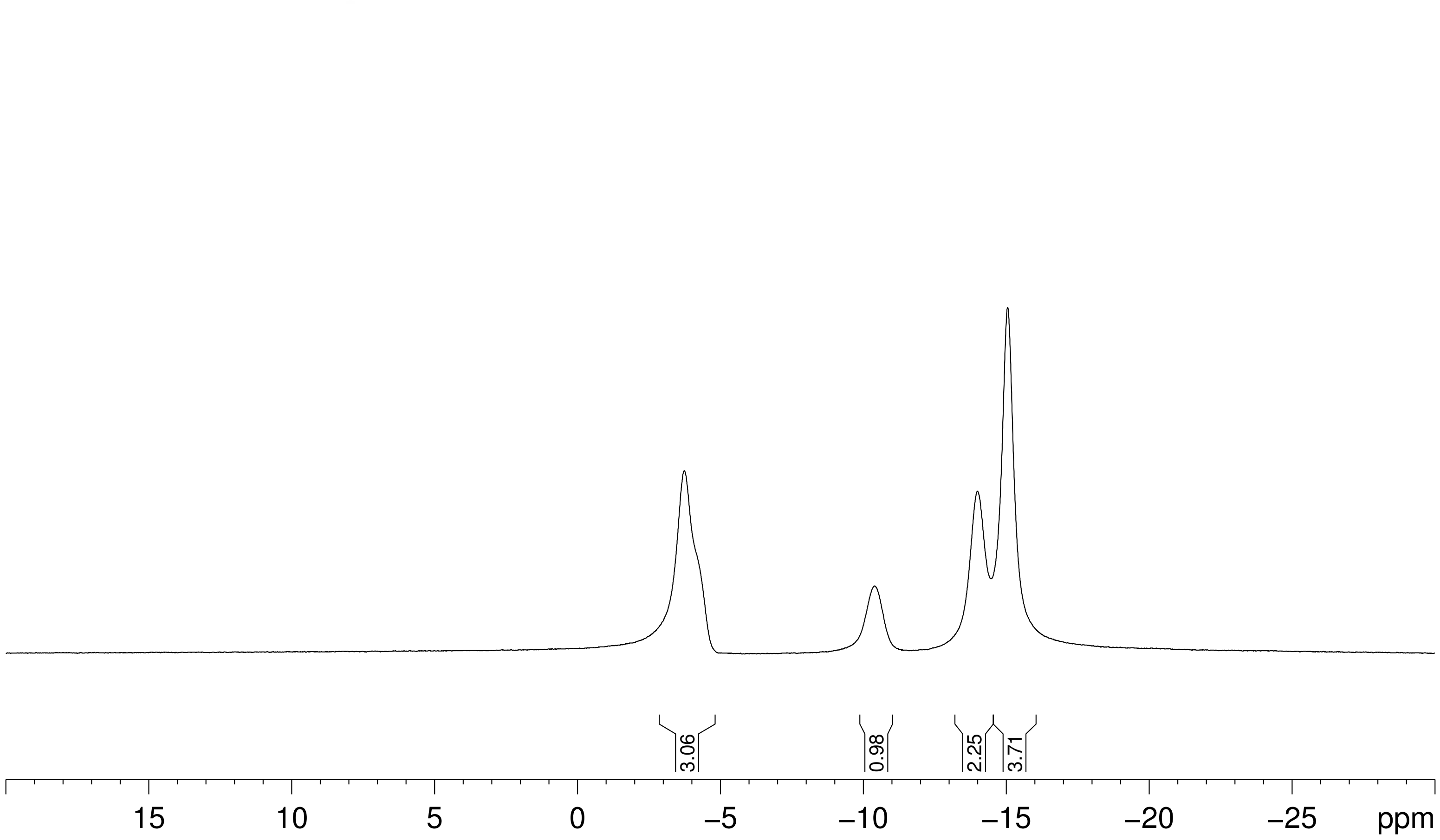

ZD-777-1-CDCl3-B(de)

NAME	ZD-777-1-CDCl3-B(de)	
EXPNO	1	
PROCNO	1	
Date_	20140917	
Time	16.56	
INSTRUM	spect	
PROBHD	5 mm PABBO BB-	
PULPROG	zgdc	
TD	65536	
SOLVENT	CDCl3	
NS	12	
DS	0	
SWH	25510.203	Hz
FIDRES	0.389255	Hz
AQ	1.2845556	sec
RG	287	
DW	19.600	usec
DE	6.50	usec
TE	296.4	K
D1	5.00000000	sec
D11	0.03000000	sec
TD0	1	
======== CHANNEL f1 ========		
NUC1	11B	
P1	7.60	usec
PL1	-3.00	dB
PL1W	55.13059616	W
SFO1	128.3968556	MHz
======== CHANNEL f2 ========		
CPDPRG2	waltz16	
NUC2	1H	
PCPD2	90.00	usec
PL2	-1.00	dB
PL12	15.16	dB
PL2W	13.56617069	W
PL12W	0.32844096	W
SFO2	400.1916008	MHz
SI	32768	
SF	128.3968847	MHz
WDW	EM	
SSB	0	
LB	3.00	Hz
GB	0	
PC	1.40	

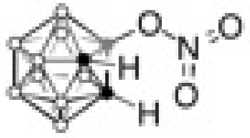

5

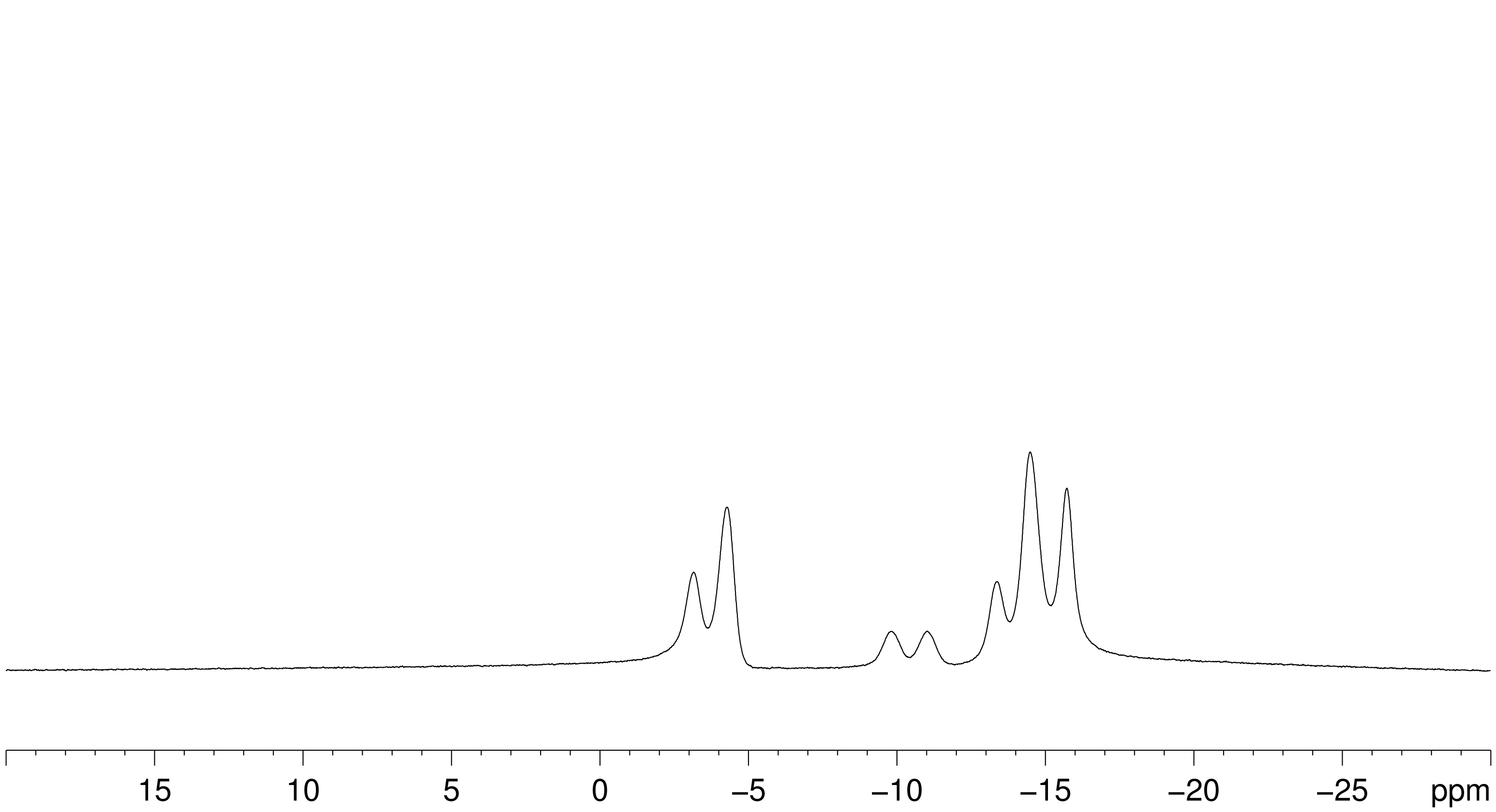

ZD-777-1-CDCl3-B(c)

NAME	ZD-777-1-CDCl3-B(c)	
EXPNO	1	
PROCNO	1	
Date_	20140917	
Time	16.58	
INSTRUM	spect	
PROBHD	5 mm PABBO BB-	
PULPROG	zg30	
TD	65536	
SOLVENT	CDCl3	
NS	16	
DS	0	
SWH	25510.203	Hz
FIDRES	0.389255	Hz
AQ	1.2845556	sec
RG	161	
DW	19.600	usec
DE	6.50	usec
TE	296.0	K
D1	5.00000000	sec
TD0	1	

======== CHANNEL f1 ========

NUC1	11B	
P1	7.60	usec
PL1	-3.00	dB
PL1W	55.13059616	W
SFO1	128.3968556	MHz
SI	32768	
SF	128.3968865	MHz
WDW	EM	
SSB	0	
LB	3.00	Hz
GB	0	
PC	1.40	

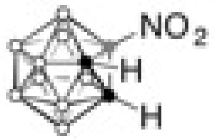

6

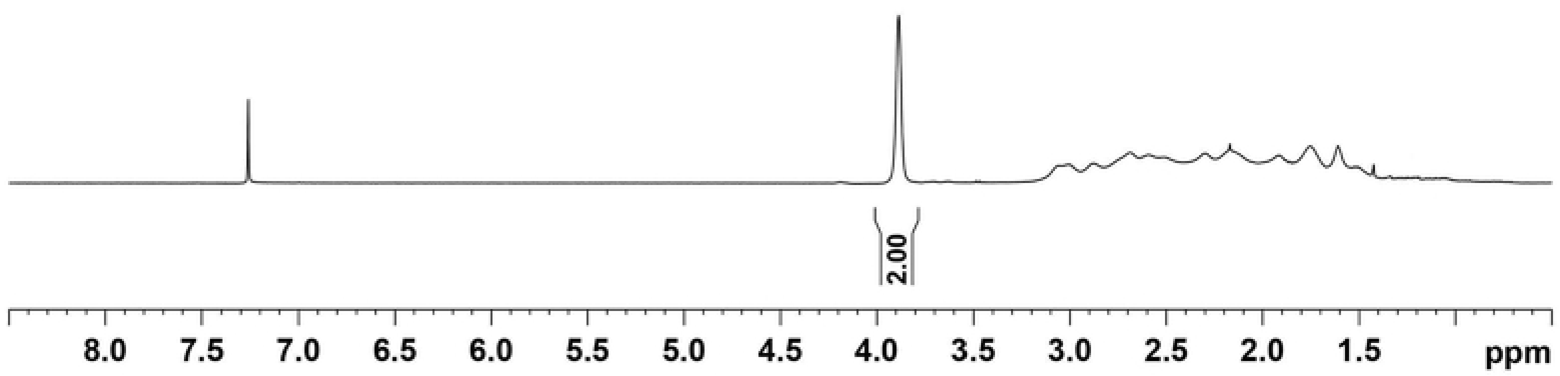

NAME	ZD-777-2-CDCl3-H
EXPNO	1
PROCNO	1
Date_	20140918
Time	17.21
INSTRUM	spect
PROBHD	5 mm PADUL 13C
PULPROG	zg
TD	65536
SOLVENT	CDCl3
NS	4
DS	0
SWH	10000.000 Hz
FIDRES	0.152588 Hz
AQ	3.2768500 sec
RG	144
DW	50.000 usec
DE	6.50 usec
TE	294.3 K
D1	1.00000000 sec
TD0	1

======== CHANNEL f1 ========

NUC1	1H
P1	14.83 usec
PL1	0.00 dB
PL1W	8.31434441 W
SFO1	400.1318000 MHz
SI	65536
SF	400.1300082 MHz
WDW	EM
SSB	0
LB	0.30 Hz
GB	0
PC	1.00

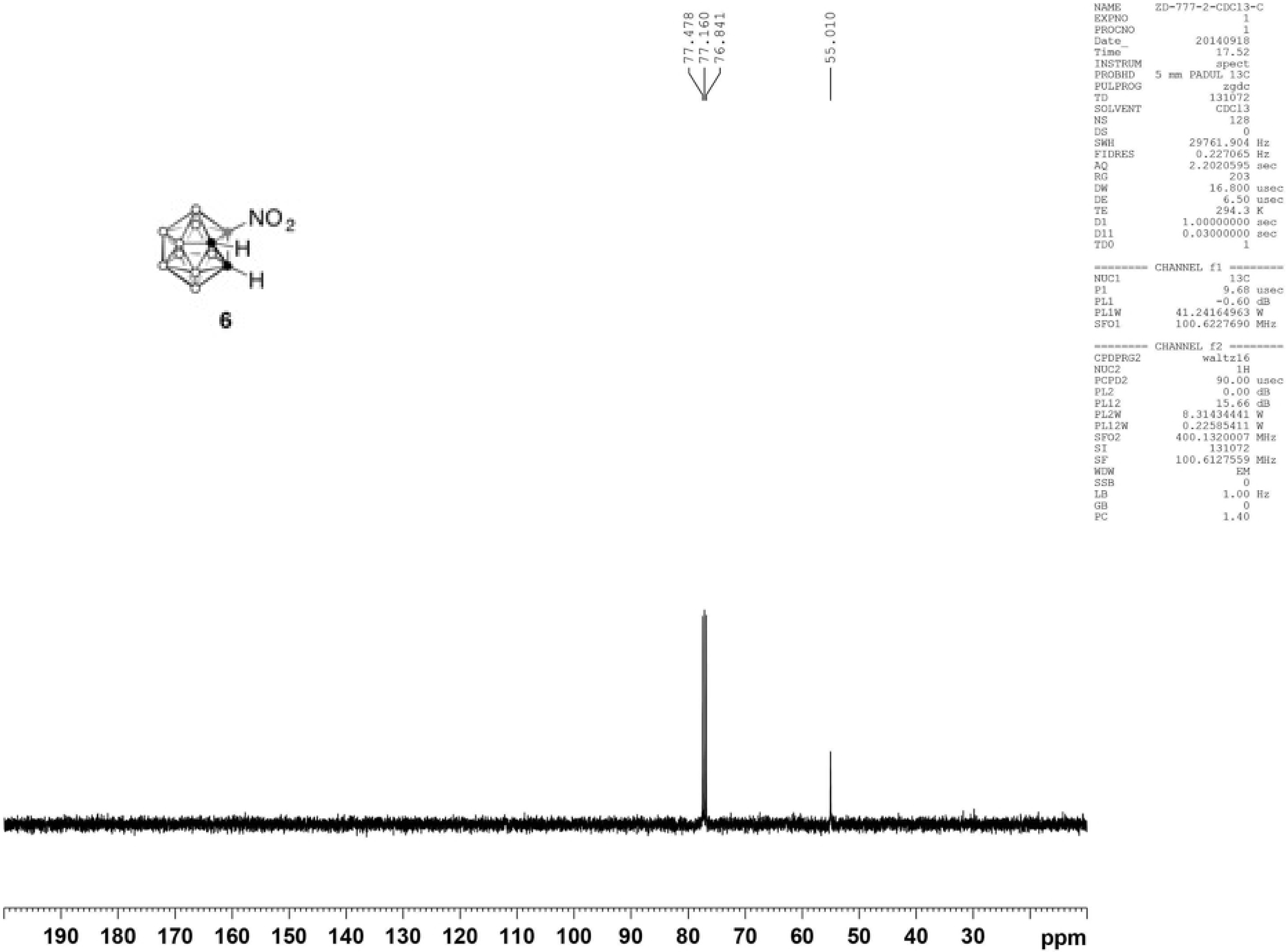
77.478
77.160
76.841
55.010
NO2
H
H
6
NAME ZD-777-2-CDCl3-C
EXPNO 1
PROCNO 1
Date_ 20140918
Time 17.52
INSTRUM spect
PROBHD 5 mm PADUL 13C
PULPROG zgdc
TD 131072
SOLVENT CDCl3
NS 128
DS 0
SWH 29761.904 Hz
FIDRES 0.227065 Hz
AQ 2.2020595 sec
RG 203
DW 16.800 usec
DE 6.50 usec
TE 294.3 K
D1 1.00000000 sec
D11 0.03000000 sec
TD0 1
======== CHANNEL f1 ========
NUC1 13C
P1 9.68 usec
PL1 -0.60 dB
PL1W 41.24164963 W
SFO1 100.6227690 MHz
======== CHANNEL f2 ========
CPDPRG2 waltz16
NUC2 1H
PCPD2 90.00 usec
PL2 0.00 dB
PL12 15.66 dB
PL2W 8.31434441 W
PL12W 0.22585411 W
SFO2 400.1320007 MHz
SI 131072
SF 100.6127559 MHz
WDW EM
SSB 0
LB 1.00 Hz
GB 0
PC 1.40
190 180 170 160 150 140 130 120 110 100 90 80 70 60 50 40 30 ppm

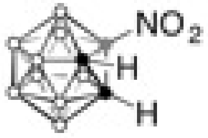

6

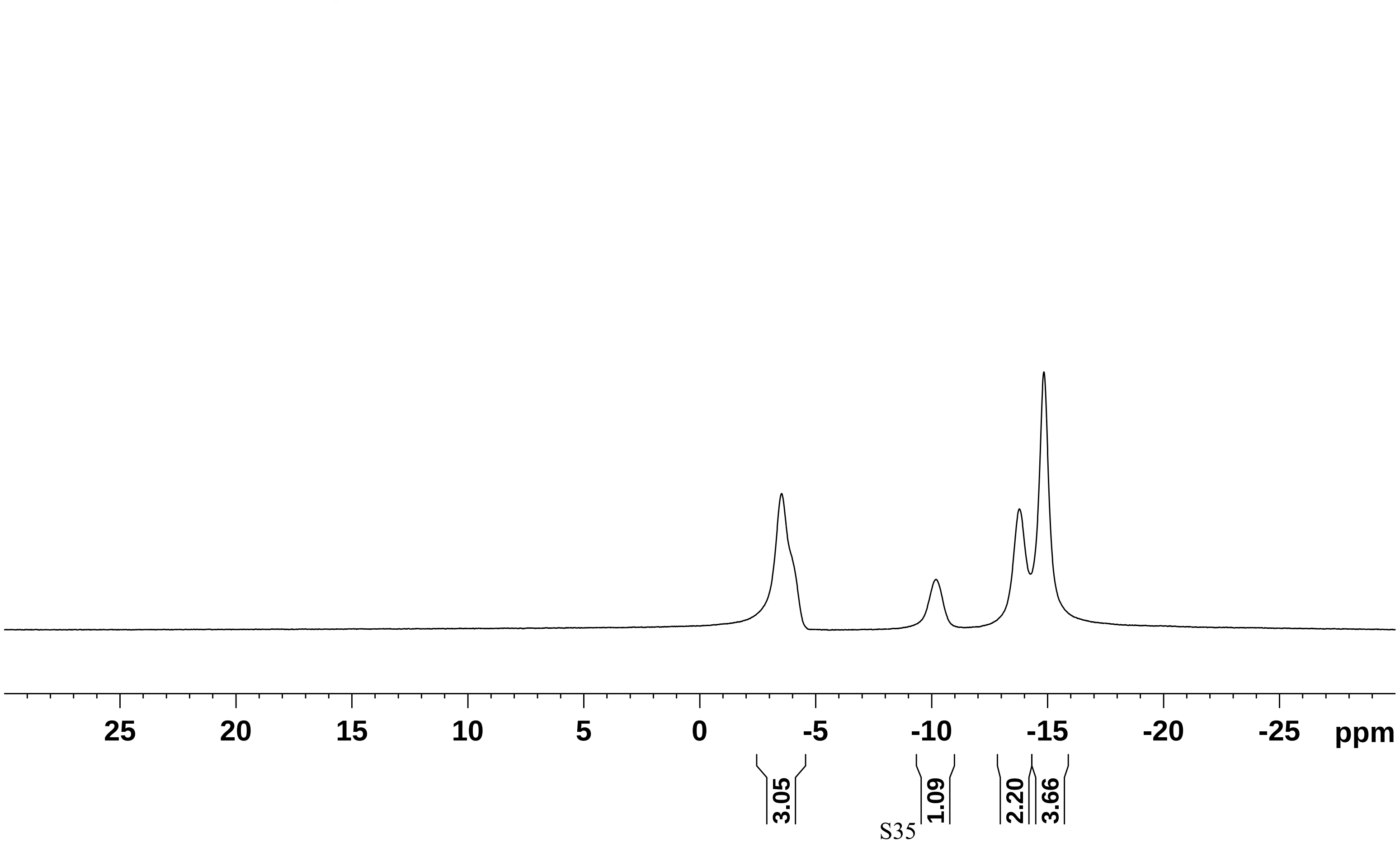


```
NAME       ZD-777-2-CDCl3-B(de)
EXPNO                  1
PROCNO                 1
Date_           20140918
Time               18.33
INSTRUM            spect
PROBHD   5 mm PABBO BB-
PULPROG             zgdc
TD                 65536
SOLVENT            CDCl3
NS                    12
DS                     0
SWH            25510.203 Hz
FIDRES          0.389255 Hz
AQ             1.2845556 sec
RG                   287
DW                19.600 usec
DE                  6.50 usec
TE                 296.4 K
D1            5.00000000 sec
D11           0.03000000 sec
TD0                    1

======== CHANNEL f1 ========
NUC1                 11B
P1                  7.60 usec
PL1                -3.00 dB
PL1W         55.13059616 W
SFO1         128.3968556 MHz

======== CHANNEL f2 ========
CPDPRG2          waltz16
NUC2                  1H
PCPD2              90.00 usec
PL2                -1.00 dB
PL12               15.16 dB
PL2W         13.56617069 W
PL12W         0.32844096 W
SFO2         400.1916008 MHz
SI                 32768
SF           128.3968582 MHz
WDW                   EM
SSB                    0
LB                  3.00 Hz
GB                     0
PC                  1.40
```

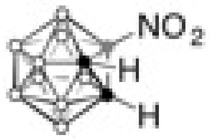

6

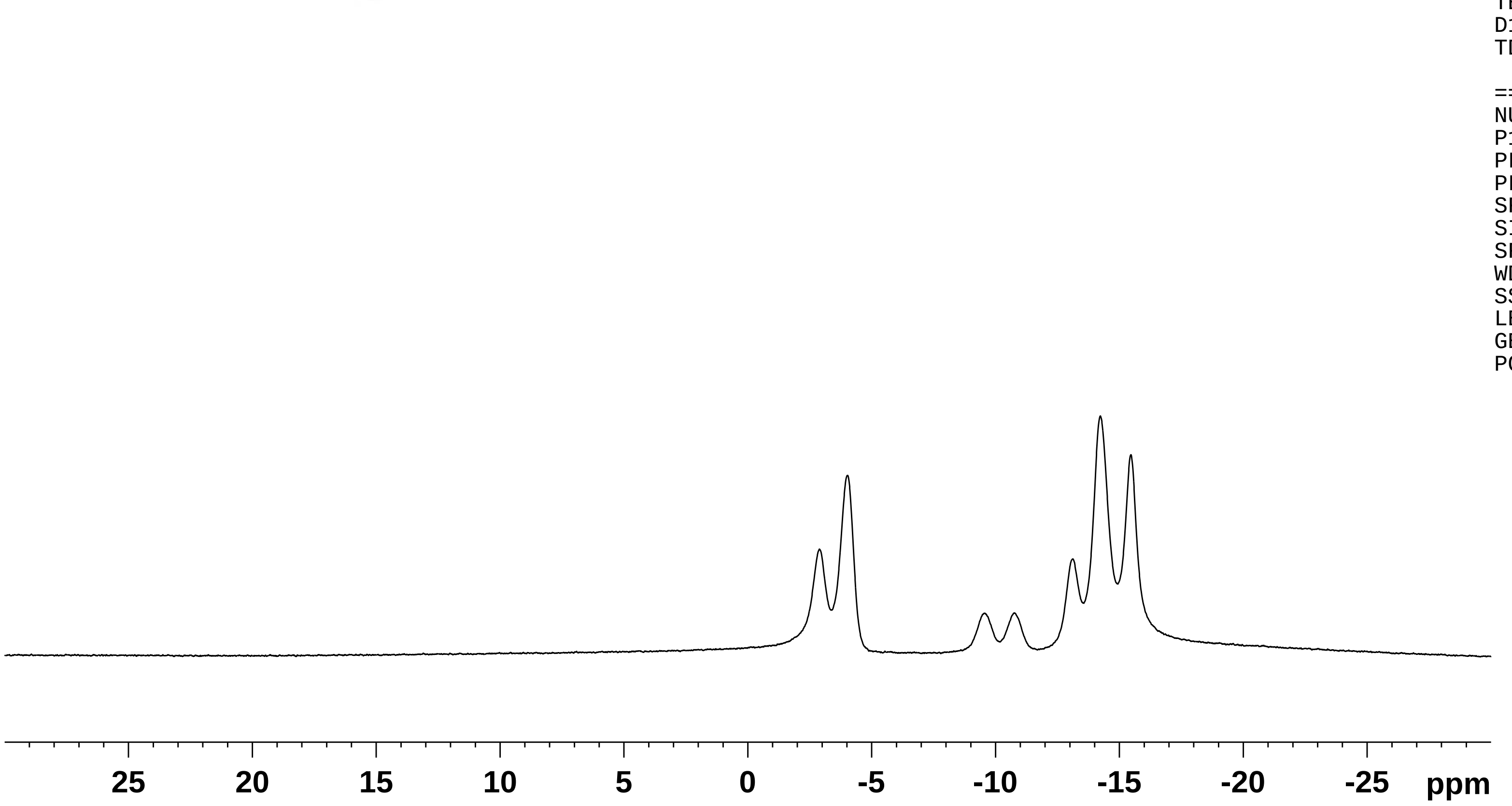

NAME	ZD-777-2-CDCl3-B(c)
EXPNO	1
PROCNO	1
Date_	20140918
Time	18.34
INSTRUM	spect
PROBHD	5 mm PABBO BB-
PULPROG	zg30
TD	65536
SOLVENT	CDCl3
NS	16
DS	0
SWH	25510.203 Hz
FIDRES	0.389255 Hz
AQ	1.2845556 sec
RG	161
DW	19.600 usec
DE	6.50 usec
TE	296.0 K
D1	5.00000000 sec
TD0	1

======== CHANNEL f1 ========

NUC1	11B
P1	7.60 usec
PL1	-3.00 dB
PL1W	55.13059616 W
SFO1	128.3968556 MHz
SI	32768
SF	128.3968533 MHz
WDW	EM
SSB	0
LB	3.00 Hz
GB	0
PC	1.40

ZD-3-N3-CDCl3-H

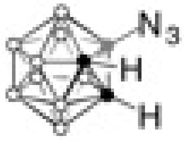

7

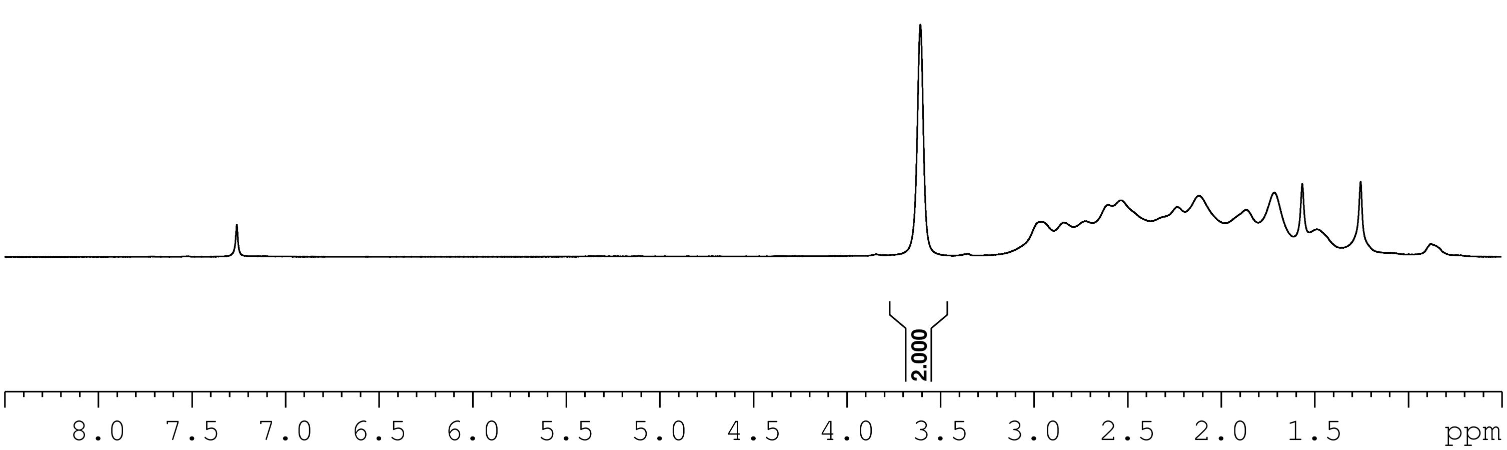


```
Current Data Parameters
NAME     ZD-3-N3-CDCl3-H
EXPNO                  1
PROCNO                 1

F2 - Acquisition Parameters
Date_           20160126
Time               21.47 h
INSTRUM            spect
PROBHD   Z824601_0021 (
PULPROG               zg
TD                 65536
SOLVENT            CDCl3
NS                     8
DS                     0
SWH             8012.820 Hz
FIDRES          0.122266 Hz
AQ             4.0894465 sec
RG                    64
DW                62.400 usec
DE                  6.50 usec
TE                 294.1 K
D1            1.00000000 sec
TD0                    1
SFO1         400.1316005 MHz
NUC1                  1H
P1                 15.00 usec
PLW1          8.31000042 W

F2 - Processing parameters
SI                 65536
SF           400.1300098 MHz
WDW                   EM
SSB      0
LB                  0.30 Hz
GB       0
PC                  1.00
```

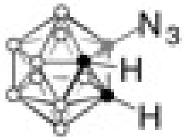

7

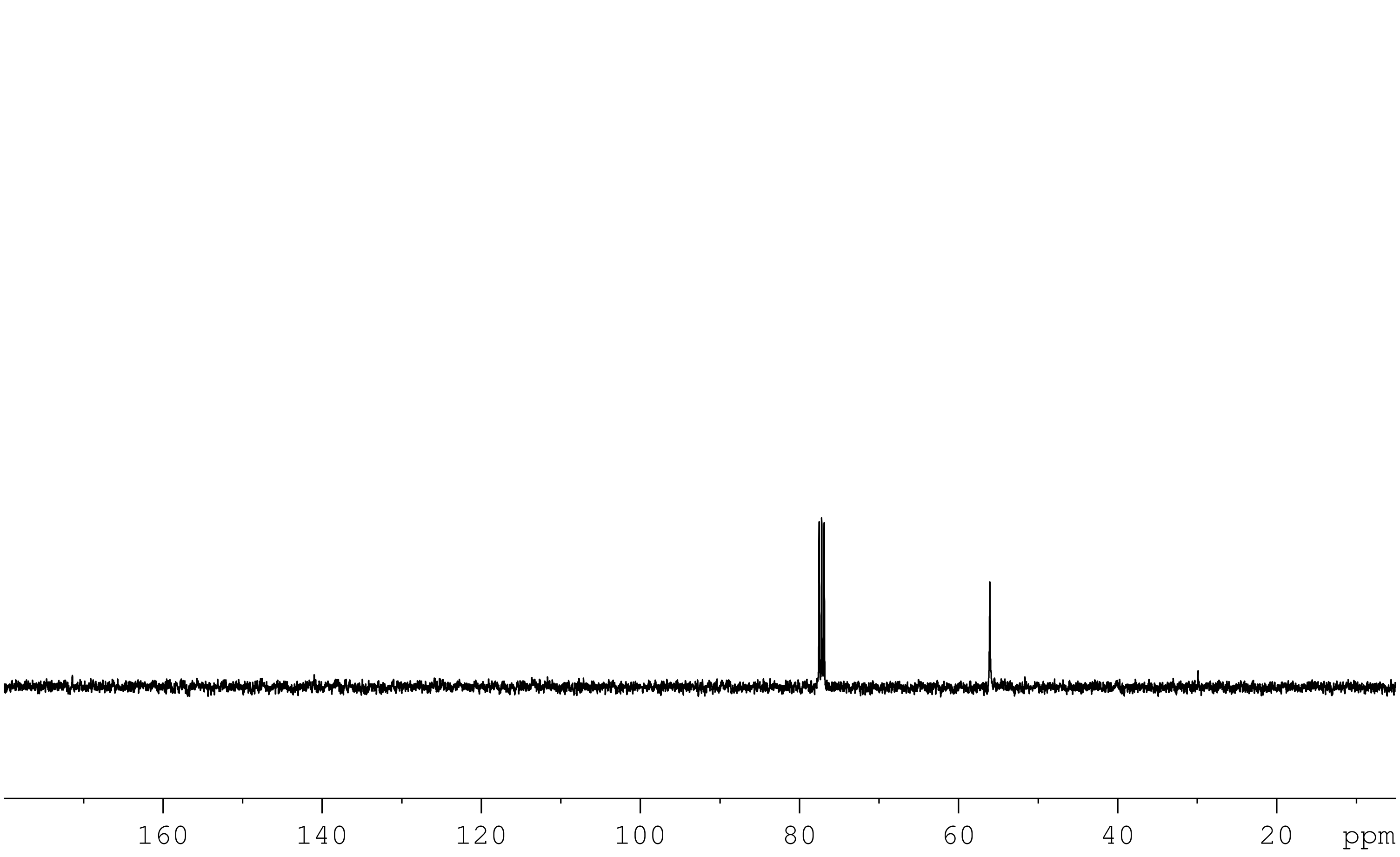

ZD-3-N3-CDCl3-C

Current Data Parameters		
NAME	ZD-3-N3-CDCl3-C	
EXPNO	1	
PROCNO	1	
F2 - Acquisition Parameters		
Date_	20160126	
Time	21.50	h
INSTRUM	spect	
PROBHD	Z824601_0021 (	
PULPROG	zgdc	
TD	131072	
SOLVENT	CDCl3	
NS	80	
DS	0	
SWH	25252.525	Hz
FIDRES	0.192661	Hz
AQ	2.5952256	sec
RG	203	
DW	19.800	usec
DE	6.50	usec
TE	294.4	K
D1	1.00000000	sec
D11	0.03000000	sec
TD0	1	
SFO1	100.6227690	MHz
NUC1	13C	
P1	9.50	usec
PLW1	41.25000000	W
SFO2	400.1320007	MHz
NUC2	1H	
CPDPRG[2	waltz16	
PCPD2	90.00	usec
PLW2	8.31000042	W
PLW12	0.23083000	W
F2 - Processing parameters		
SI	131072	
SF	100.6127569	MHz
WDW	EM	
SSB	0	
LB	3.00	Hz
GB	0	
PC	1.40	

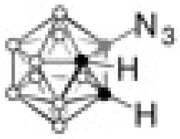

7

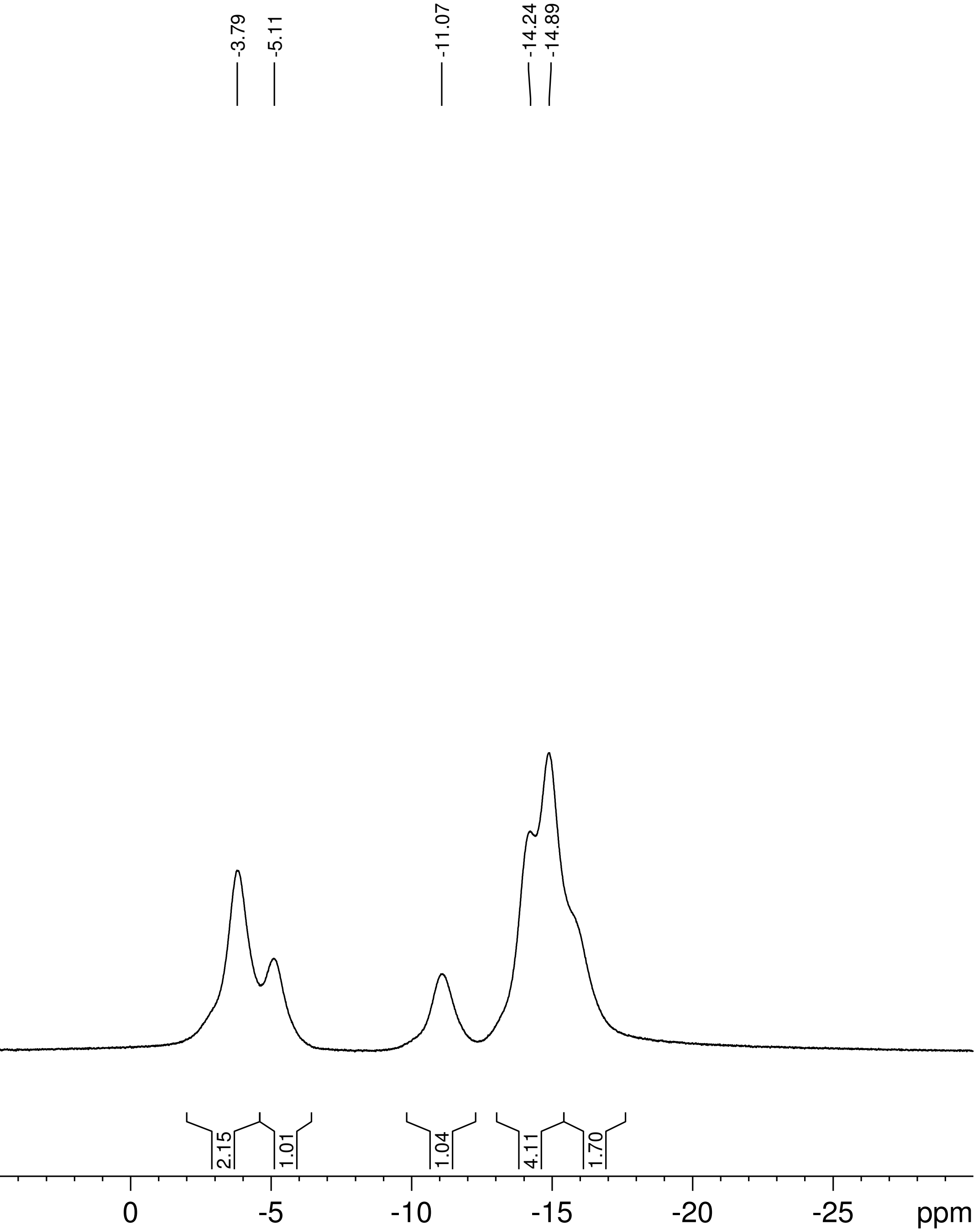

ZD-3-N3-CDCl3-B(de)

Current Data Parameters
NAME ZD-3-N3-CDCl3-B(de)
EXPNO 1
PROCNO 1

F2 - Acquisition Parameters
Date_ 20160126
Time 22.01 h
INSTRUM spect
PROBHD Z108618_0257 (
PULPROG zgdc
TD 65536
SOLVENT CD2Cl2
NS 8
DS 4
SWH 24038.461 Hz
FIDRES 0.366798 Hz
AQ 1.3631488 sec
RG 203
DW 20.800 usec
DE 6.50 usec
TE 296.2 K
D1 2.00000000 sec
D11 0.03000000 sec
TD0 1
SFO1 128.4096890 MHz
NUC1 11B
P1 7.50 usec
PLW1 55.09999847 W
SFO2 400.2316009 MHz
NUC2 1H
CPDPRG[2 waltz16
PCPD2 90.00 usec
PLW2 13.56000042 W
PLW12 0.27428001 W

F2 - Processing parameters
SI 32768
SF 128.4097615 MHz
WDW EM
SSB 0
LB 1.00 Hz
GB 0
PC 1.40

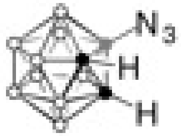

7

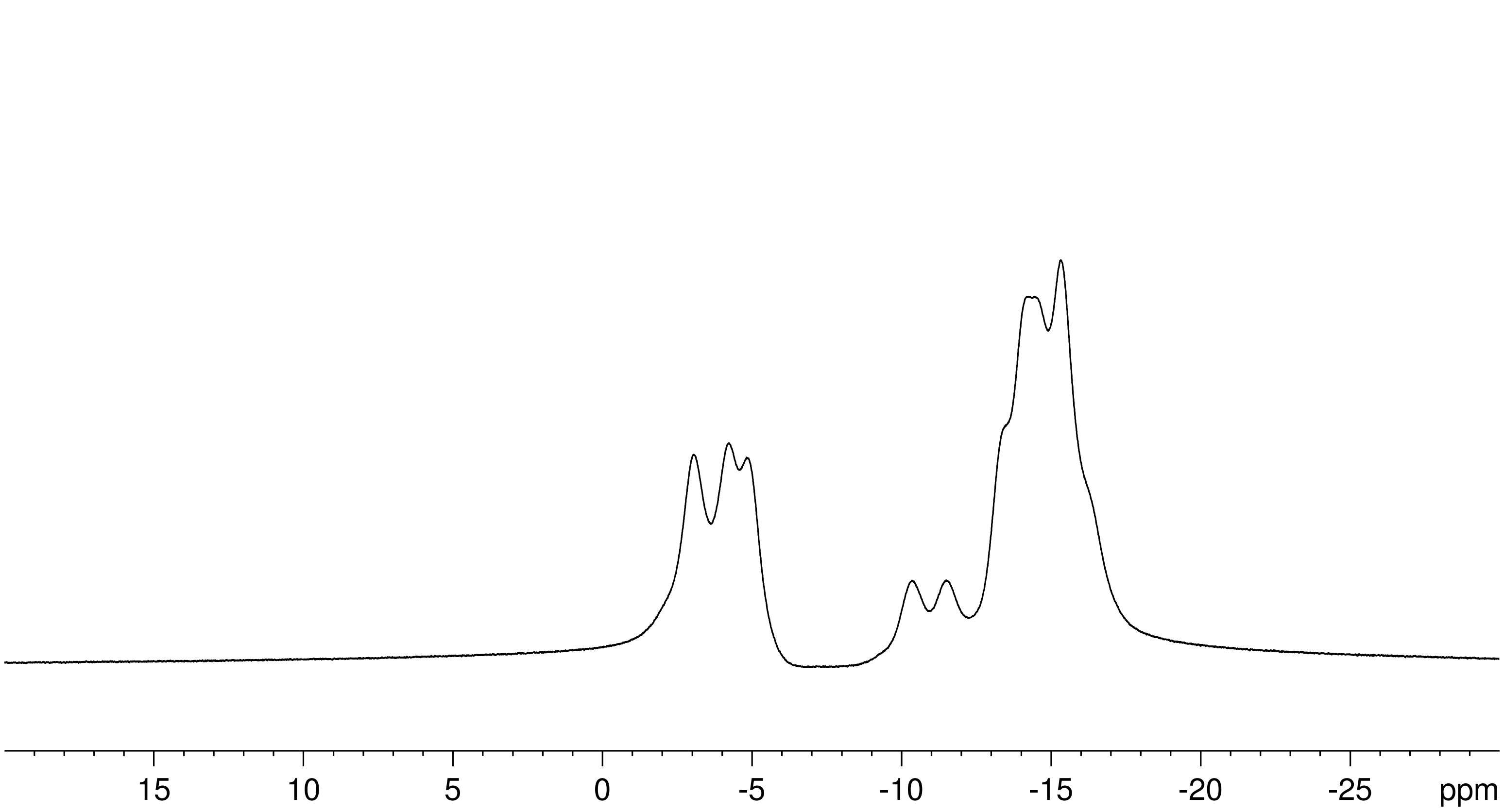

ZD-3-N3-CDCl3-B(c)

Current Data Parameters

NAME	ZD-3-N3-CDCl3-B(c)
EXPNO	1
PROCNO	1

F2 - Acquisition Parameters

Date_	20160126
Time	22.03 h
INSTRUM	spect
PROBHD	Z108618_0257 (
PULPROG	zg
TD	65536
SOLVENT	CD2Cl2
NS	43
DS	2
SWH	24038.461 Hz
FIDRES	0.366798 Hz
AQ	1.3631488 sec
RG	101
DW	20.800 usec
DE	6.50 usec
TE	295.8 K
D1	2.00000000 sec
TD0	1
SFO1	128.4096890 MHz
NUC1	11B
P1	7.50 usec
PLW1	55.09999847 W

F2 - Processing parameters

SI	32768
SF	128.4097430 MHz
WDW	EM
SSB	0
LB	1.00 Hz
GB	0
PC	1.40

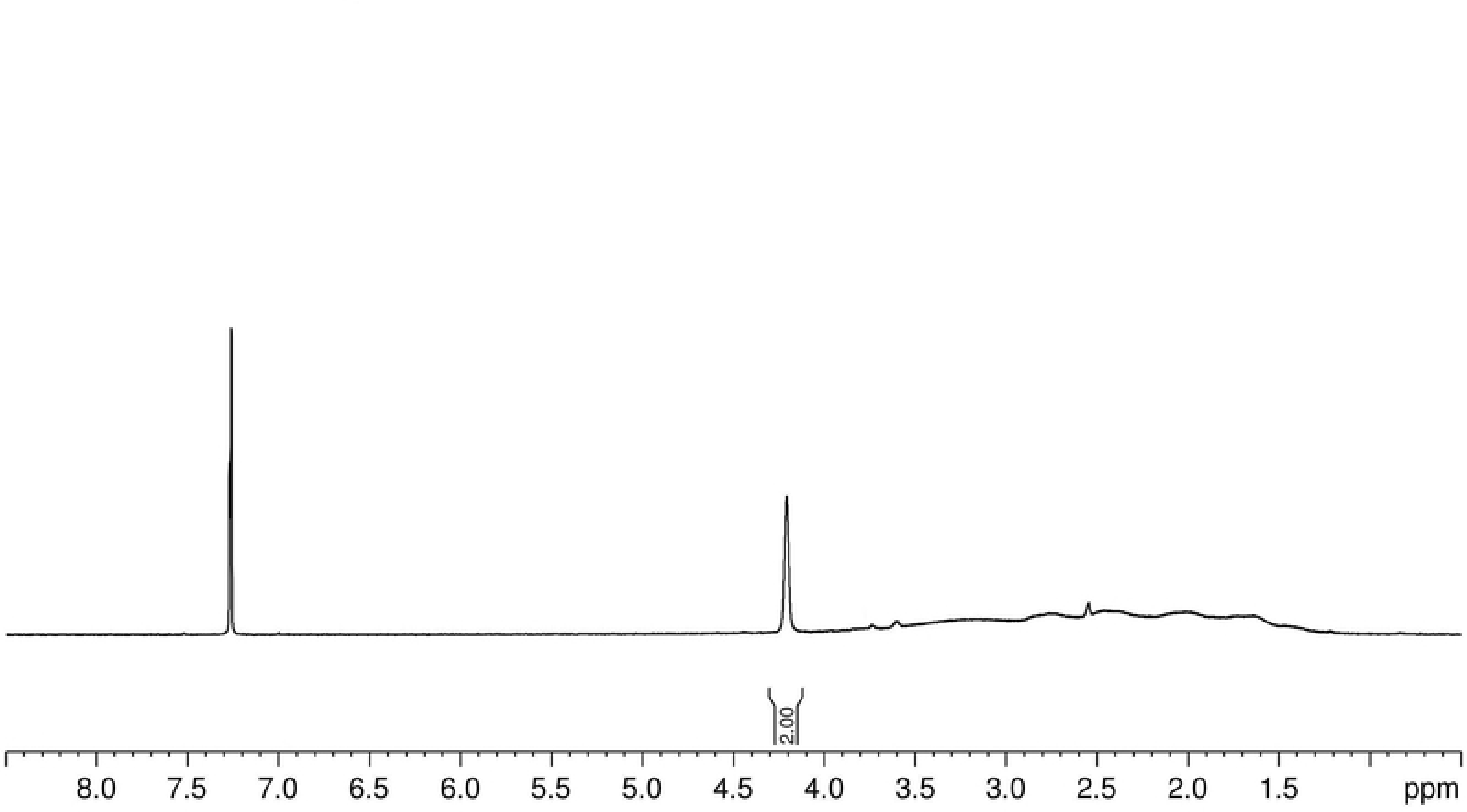

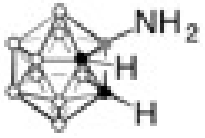

8

Current Data Parameters
NAME ZD-3-NH2-CDCl3-H
EXPNO 1
PROCNO 1

F2 - Acquisition Parameters
Date_ 20150321
Time 11.45 h
INSTRUM spect
PROBHD Z108618_0257 (
PULPROG zg30
TD 65536
SOLVENT CDCl3
NS 8
DS 2
SWH 8012.820 Hz
FIDRES 0.122266 Hz
AQ 4.0894465 sec
RG 203
DW 62.400 usec
DE 6.50 usec
TE 294.7 K
D1 1.00000000 sec
TD0 1
SFO1 400.2324714 MHz
NUC1 1H
P1 12.80 usec
PLW1 13.56000042 W

F2 - Processing parameters
SI 65536
SF 400.2300094 MHz
WDW EM
SSB 0
LB 0.30 Hz
GB 0
PC 1.00

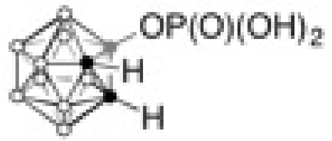

9

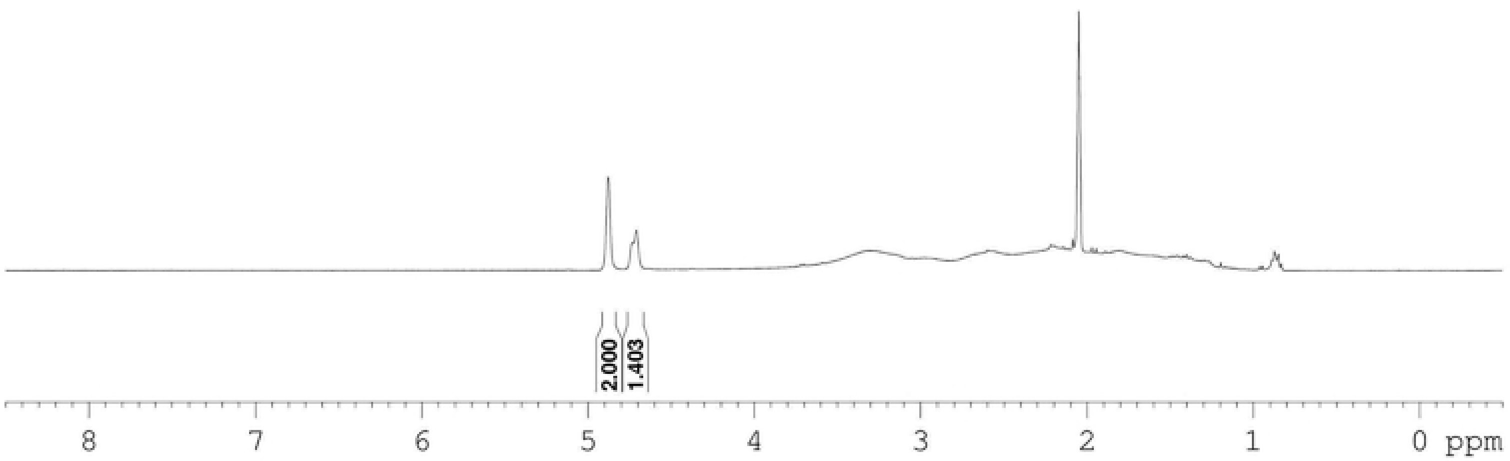

ZD-774-acetone-H

Bruker Advance III 400

NAME	ZD-774-acetone-H
EXPNO	1
PROCNO	1
Date_	20140920
Time	11.24
INSTRUM	spect
PROBHD	5 mm PADUL 13C
PULPROG	zg
TD	65536
SOLVENT	Acetone
NS	16
DS	0
SWH	10000.000 Hz
FIDRES	0.152588 Hz
AQ	3.2768500 sec
RG	144
DW	50.000 usec
DE	6.50 usec
TE	294.3 K
D1	1.00000000 sec
TD0	1
======== CHANNEL f1 ========	
NUC1	1H
P1	14.83 usec
PL1	0.00 dB
PL1W	8.31434441 W
SFO1	400.1318000 MHz
SI	65536
SF	400.1300059 MHz
WDW	EM
SSB	0
LB	0.30 Hz
GB	0
PC	1.00

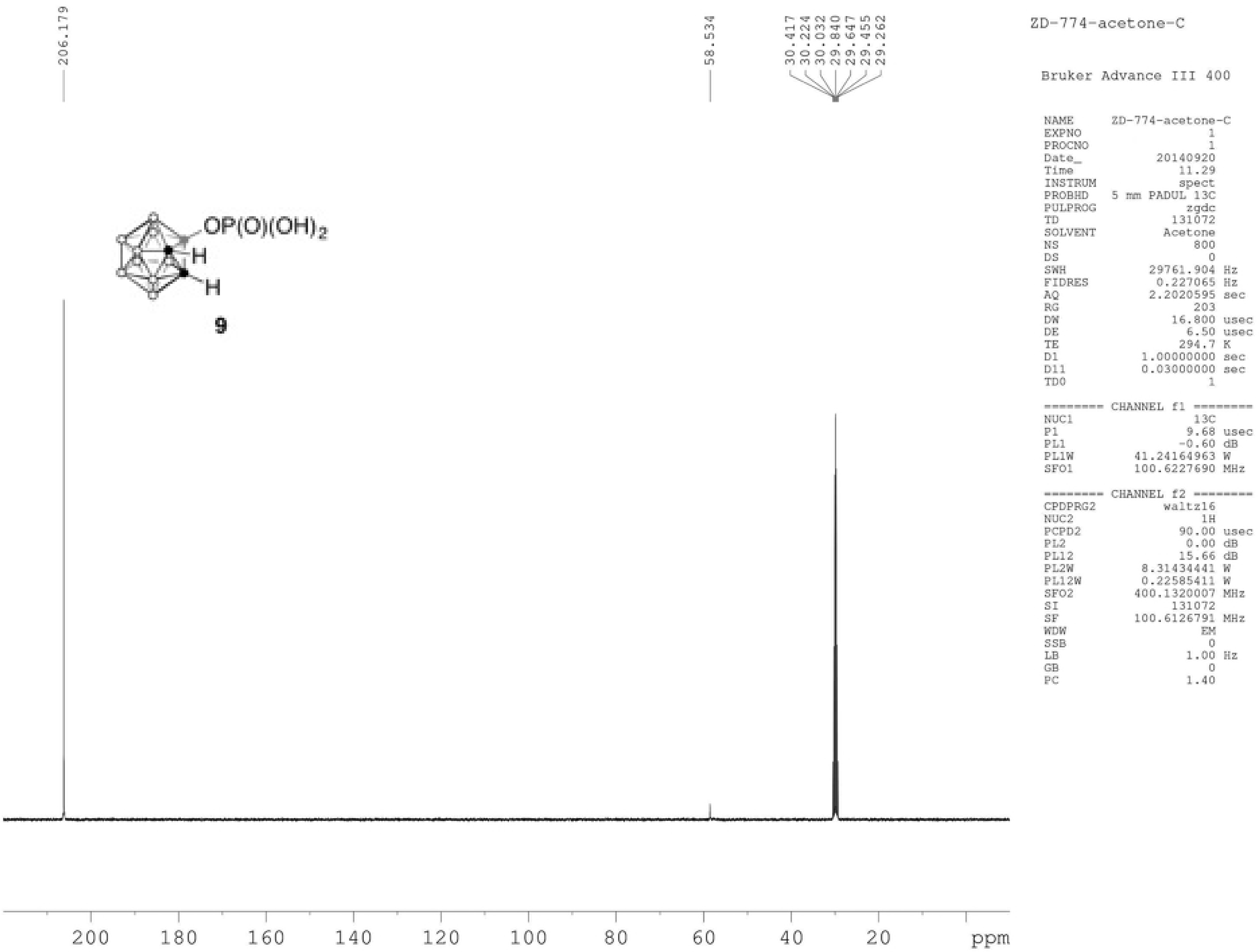
206.179
58.534
30.417
30.224
30.032
29.840
29.647
29.455
29.262
ZD-774-acetone-C
Bruker Advance III 400
NAME ZD-774-acetone-C
EXPNO 1
PROCNO 1
Date_ 20140920
Time 11.29
INSTRUM spect
PROBHD 5 mm PADUL 13C
PULPROG zgdc
TD 131072
SOLVENT Acetone
NS 800
DS 0
SWH 29761.904 Hz
FIDRES 0.227065 Hz
AQ 2.2020595 sec
RG 203
DW 16.800 usec
DE 6.50 usec
TE 294.7 K
D1 1.00000000 sec
D11 0.03000000 sec
TD0 1
======== CHANNEL f1 ========
NUC1 13C
P1 9.68 usec
PL1 -0.60 dB
PL1W 41.24164963 W
SFO1 100.6227690 MHz
======== CHANNEL f2 ========
CPDPRG2 waltz16
NUC2 1H
PCPD2 90.00 usec
PL2 0.00 dB
PL12 15.66 dB
PL2W 8.31434441 W
PL12W 0.22585411 W
SFO2 400.1320007 MHz
SI 131072
SF 100.6126791 MHz
WDW EM
SSB 0
LB 1.00 Hz
GB 0
PC 1.40
OP(O)(OH)2
H
H
9
200
180
160
140
120
100
80
60
40
20
ppm

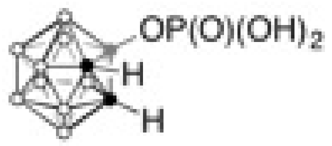

9

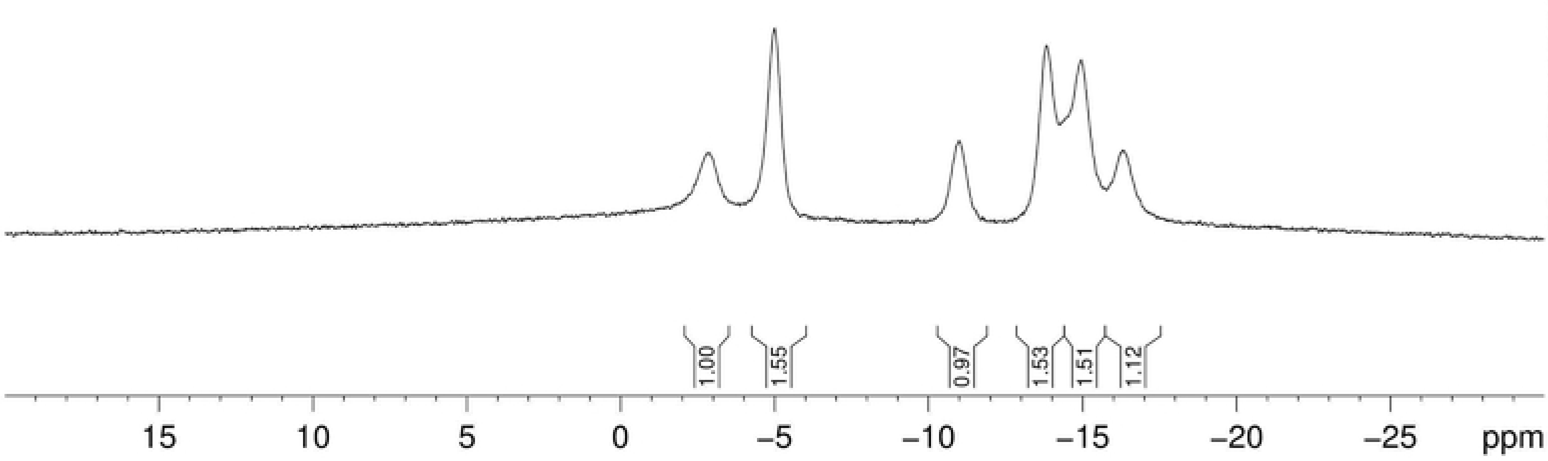

NAME	ZD-774-Acetone-B(de)	
EXPNO	1	
PROCNO	1	
Date_	20141011	
Time	10.54	
INSTRUM	spect	
PROBHD	5 mm PABBO BB-	
PULPROG	zgdc	
TD	65536	
SOLVENT	Acetone	
NS	8	
DS	0	
SWH	25510.203	Hz
FIDRES	0.389255	Hz
AQ	1.2845556	sec
RG	362	
DW	19.600	usec
DE	6.50	usec
TE	299.1	K
D1	5.00000000	sec
D11	0.03000000	sec
TD0	1	
========	CHANNEL f1	========
NUC1	11B	
P1	7.60	usec
PL1	-3.00	dB
PL1W	55.13059616	W
SFO1	128.3968556	MHz
========	CHANNEL f2	========
CPDPRG2	waltz16	
NUC2	1H	
PCPD2	90.00	usec
PL2	-1.00	dB
PL12	15.16	dB
PL2W	13.56617069	W
PL12W	0.32844096	W
SFO2	400.1916008	MHz
SI	32768	
SF	128.3968847	MHz
WDW	EM	
SSB	0	
LB	3.00	Hz
GB	0	
PC	1.40	

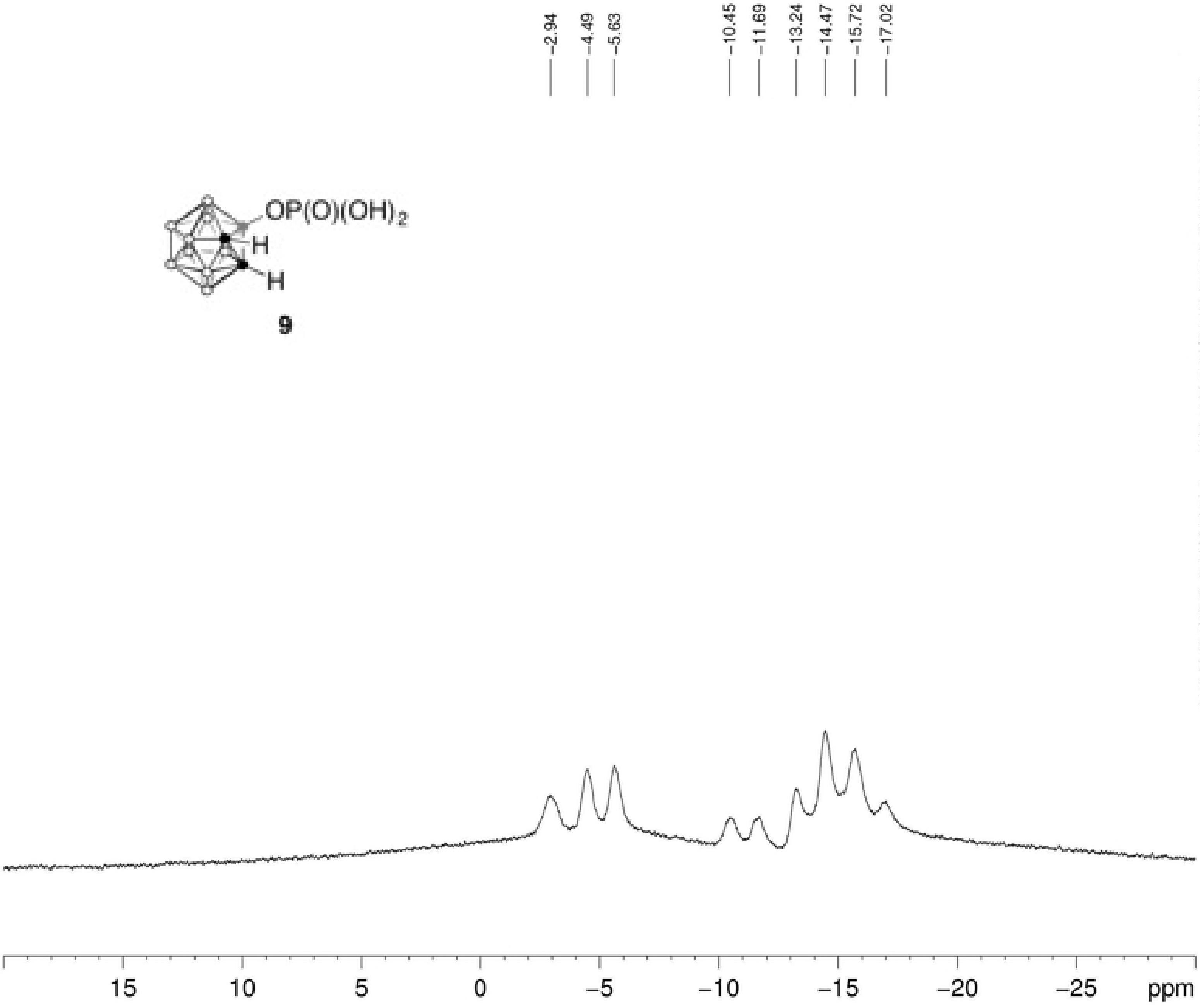

NAME	ZD-774-Acetone-B(c)
EXPNO	1
PROCNO	1
Date_	20141011
Time	10.55
INSTRUM	spect
PROBHD	5 mm PABBO BB-
PULPROG	zg30
TD	65536
SOLVENT	Acetone
NS	12
DS	0
SWH	25510.203 Hz
FIDRES	0.389255 Hz
AQ	1.2845556 sec
RG	161
DW	19.600 usec
DE	6.50 usec
TE	299.1 K
D1	5.00000000 sec
TD0	1
======== CHANNEL f1 ========	
NUC1	11B
P1	7.60 usec
PL1	-3.00 dB
PL1W	55.13059616 W
SFO1	128.3968556 MHz
SI	32768
SF	128.3968865 MHz
WDW	EM
SSB	0
LB	3.00 Hz
GB	0
PC	1.40

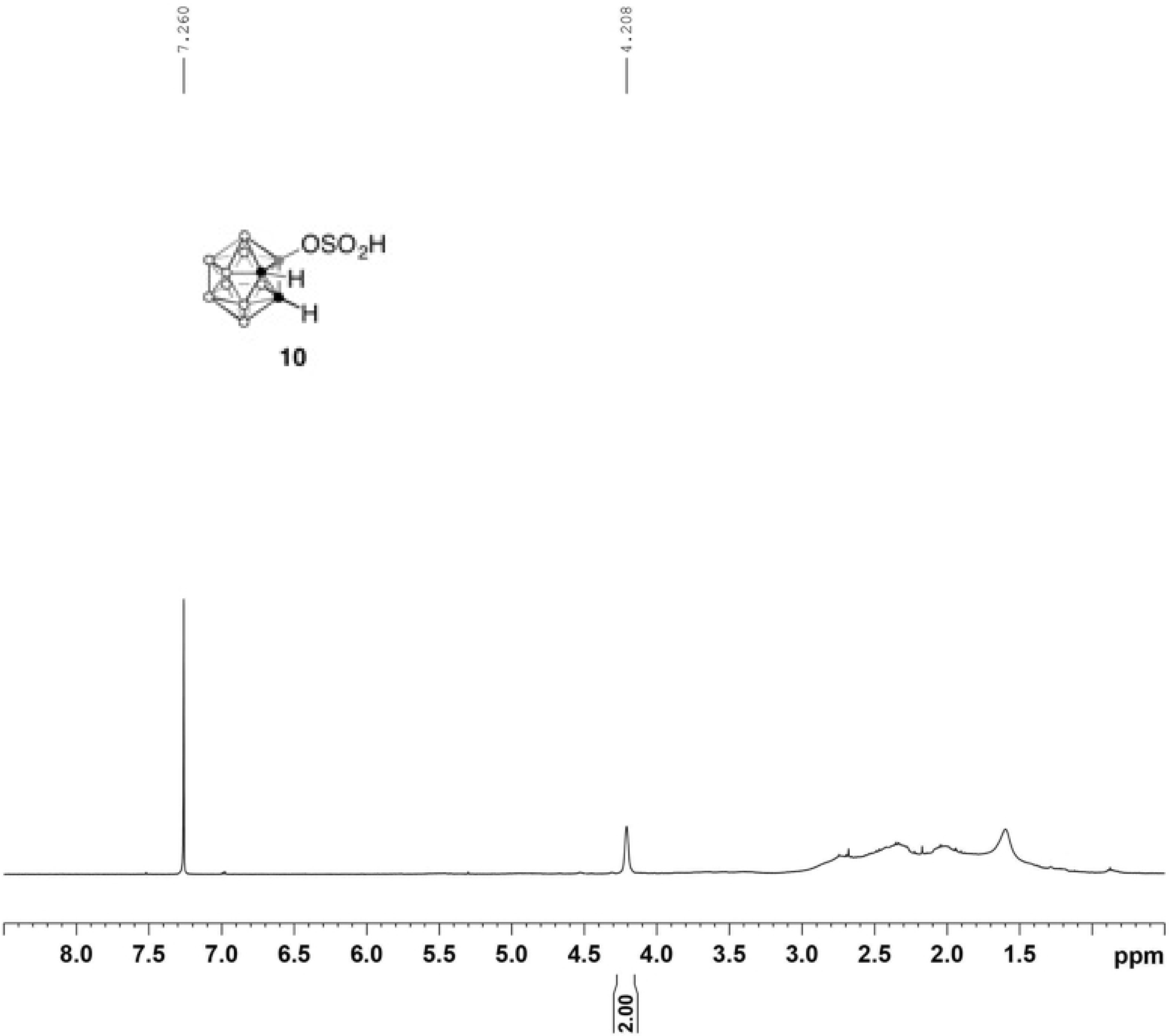

NAME	ZD-10-CDCl3-H
EXPNO	1
PROCNO	1
Date_	20160129
Time	22.59 h
INSTRUM	spect
PROBHD	Z824601_0021 (
PULPROG	zg
TD	65536
SOLVENT	CDCl3
NS	16
DS	0
SWH	8012.820 Hz
FIDRES	0.122266 Hz
AQ	4.0894966 sec
RG	181
DW	62.400 usec
DE	6.50 usec
TE	294.0 K
D1	1.00000000 sec
TD0	1
SFO1	400.1316005 MHz
NUC1	1H
P1	15.00 usec
SI	65536
SF	400.1300098 MHz
WDW	EM
SSB	0
LB	0.30 Hz
GB	0
PC	1.00

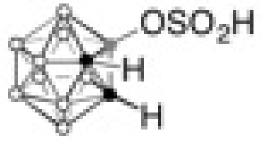

10

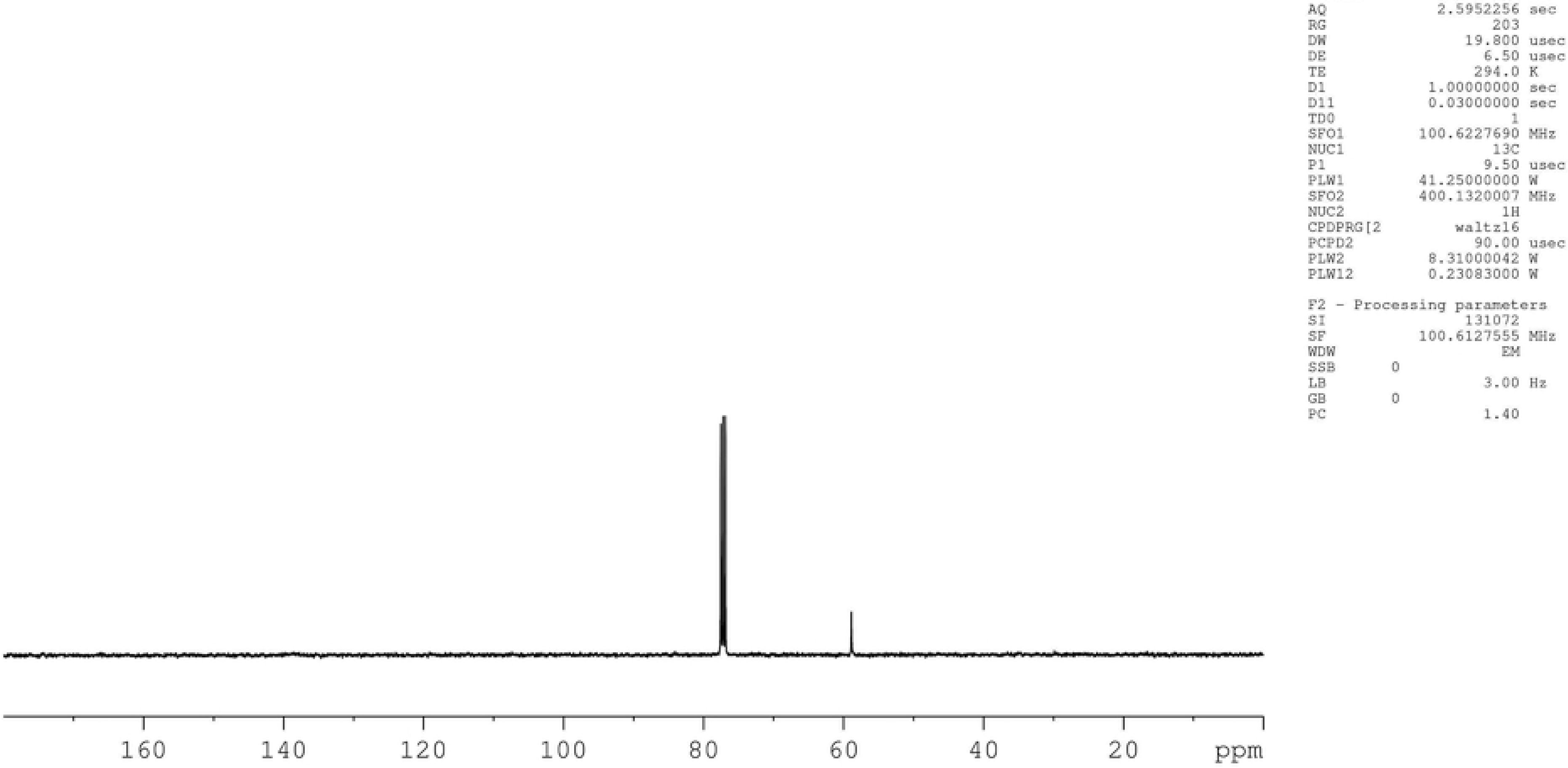

77.477
77.160
76.842
58.792
160
140
120
100
80
60
40
20
ppm
ZD-3-10-CDCl3-C
Current Data Parameters
NAME 1
EXPNO 1
PROCNO 1
F2 - Acquisition Parameters
Date_ 20150915
Time 12.22 h
INSTRUM spect
PROBHD Z824601_0021 (
PULPROG zgdc
TD 131072
SOLVENT CDCl3
NS 1180
DS 0
SWH 25252.525 Hz
FIDRES 0.192661 Hz
AQ 2.5952256 sec
RG 203
DW 19.800 usec
DE 6.50 usec
TE 294.0 K
D1 1.00000000 sec
D11 0.03000000 sec
TD0 1
SFO1 100.6227690 MHz
NUC1 13C
P1 9.50 usec
PLW1 41.25000000 W
SFO2 400.1320007 MHz
NUC2 1H
CPDPRG[2 waltz16
PCPD2 90.00 usec
PLW2 8.31000042 W
PLW12 0.23083000 W
F2 - Processing parameters
SI 131072
SF 100.6127555 MHz
WDW EM
SSB 0
LB 3.00 Hz
GB 0
PC 1.40

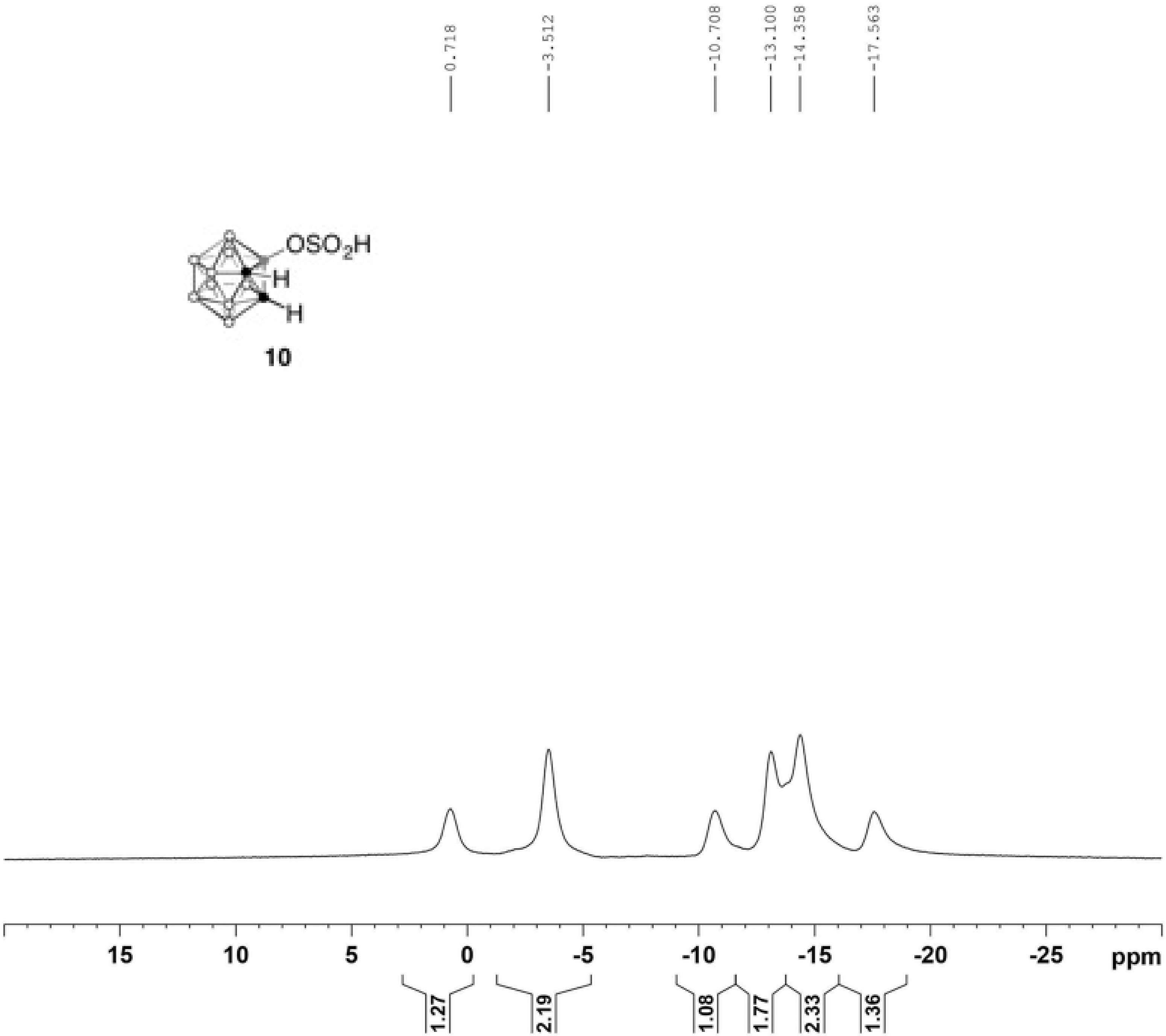

10

NAME	ZD-10-CDCl3-B(de)
EXPNO	1
PROCNO	1
Date_	20150730
Time	12.52
INSTRUM	spect
PROBHD	5 mm PABBO BB-
PULPROG	zgdc
TD	65536
SOLVENT	CDCl3
NS	23
DS	0
SWH	25510.203 Hz
FIDRES	0.389255 Hz
AQ	1.2845556 sec
RG	362
DW	19.600 usec
DE	6.50 usec
TE	300.8 K
D1	5.00000000 sec
D11	0.03000000 sec
TD0	1

======== CHANNEL f1 ========

NUC1	11B
P1	7.60 usec
PL1	-3.00 dB
PL1W	55.13059616 W
SFO1	128.3968556 MHz

======== CHANNEL f2 ========

CPDPRG2	waltz16
NUC2	1H
PCPD2	90.00 usec
PL2	-1.00 dB
PL12	15.16 dB
PL2W	13.56617069 W
PL12W	0.32844096 W
SFO2	400.1916008 MHz
SI	32768
SF	128.3966840 MHz
WDW	EM
SSB	0
LB	3.00 Hz
GB	0
PC	1.40

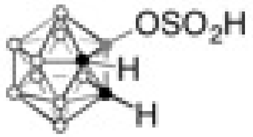

10

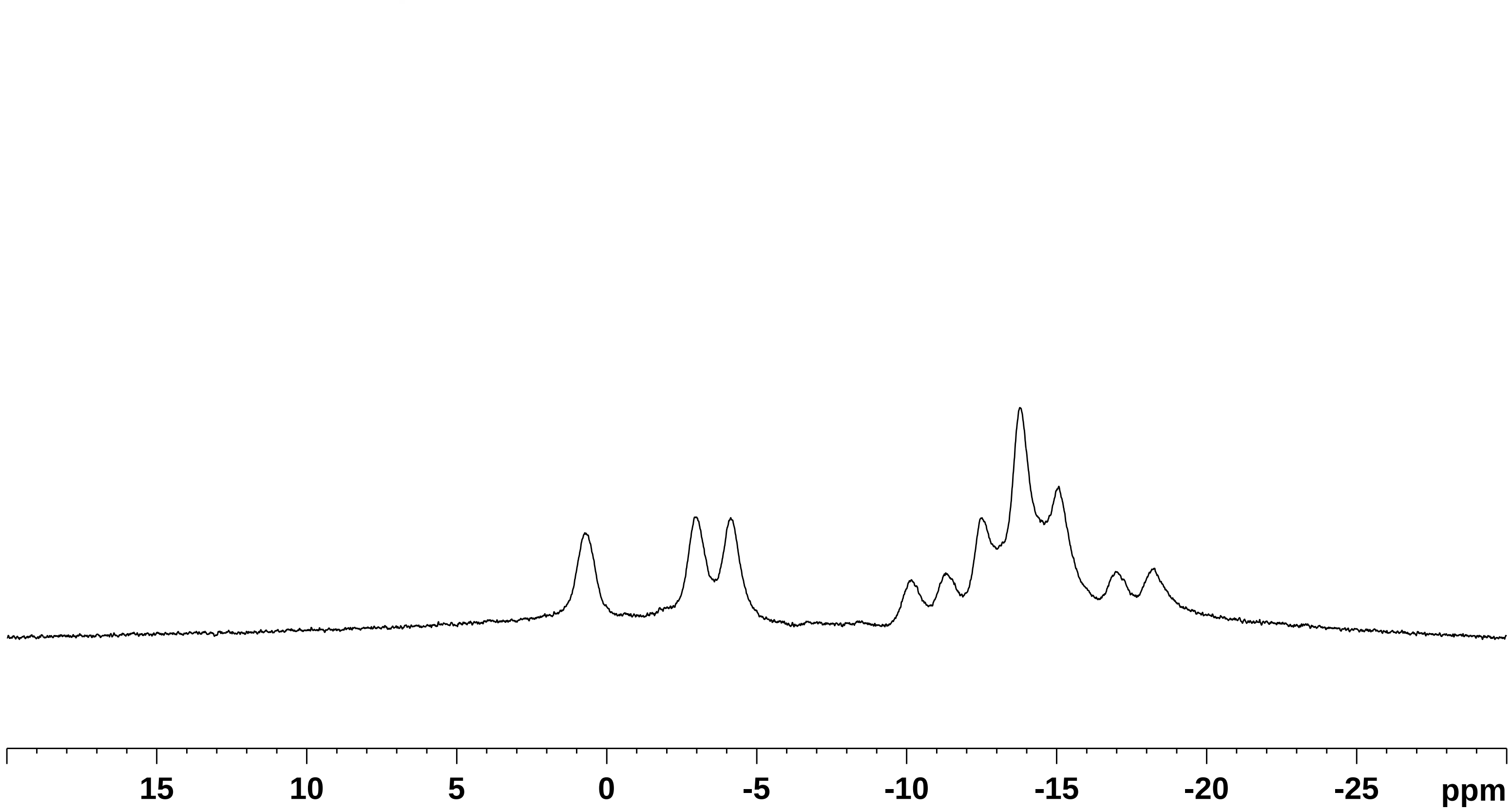


```
NAME      ZD-10-CDCl3-B(c)
EXPNO                    1
PROCNO                   1
Date_             20150730
Time                 12.33
INSTRUM              spect
PROBHD    5 mm PABBO BB-
PULPROG               zg30
TD                   65536
SOLVENT              CDCl3
NS                      12
DS                       0
SWH              25510.203 Hz
FIDRES            0.389255 Hz
AQ               1.2845556 sec
RG                     456
DW                  19.600 usec
DE                    6.50 usec
TE                   300.3 K
D1              5.00000000 sec
TD0                      1

======== CHANNEL f1 ========
NUC1                   11B
P1                    7.60 usec
PL1                  -3.00 dB
PL1W           55.13059616 W
SFO1           128.3968556 MHz
SI                   32768
SF             128.3966897 MHz
WDW                     EM
SSB                      0
LB                    3.00 Hz
GB                       0
PC                    1.40
```

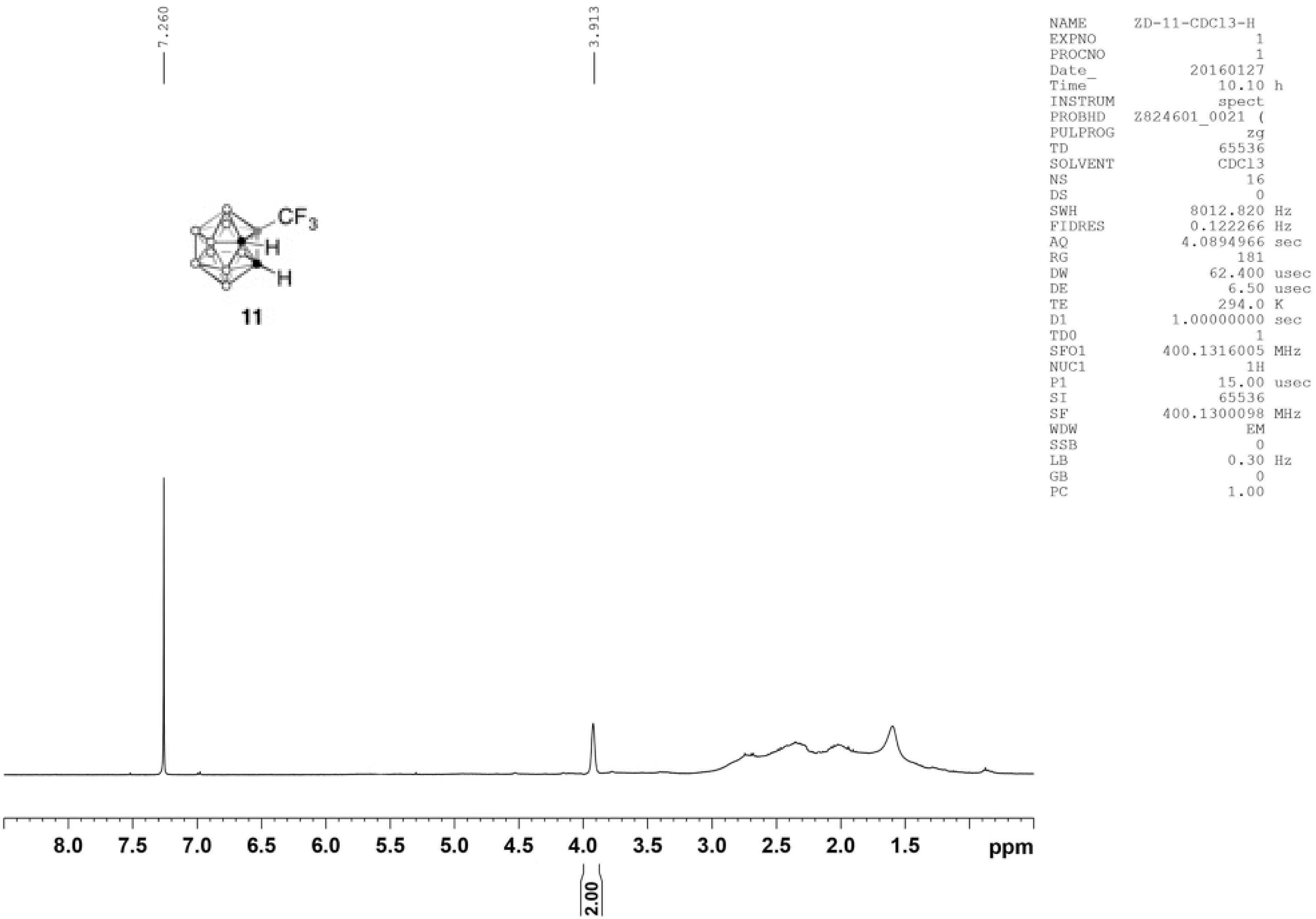
7.260
3.913
CF3
H
H
11
8.0 7.5 7.0 6.5 6.0 5.5 5.0 4.5 4.0 3.5 3.0 2.5 2.0 1.5 ppm
2.00
NAME ZD-11-CDCl3-H
EXPNO 1
PROCNO 1
Date_ 20160127
Time 10.10 h
INSTRUM spect
PROBHD Z824601_0021 (
PULPROG zg
TD 65536
SOLVENT CDCl3
NS 16
DS 0
SWH 8012.820 Hz
FIDRES 0.122266 Hz
AQ 4.0894966 sec
RG 181
DW 62.400 usec
DE 6.50 usec
TE 294.0 K
D1 1.00000000 sec
TD0 1
SFO1 400.1316005 MHz
NUC1 1H
P1 15.00 usec
SI 65536
SF 400.1300098 MHz
WDW EM
SSB 0
LB 0.30 Hz
GB 0
PC 1.00

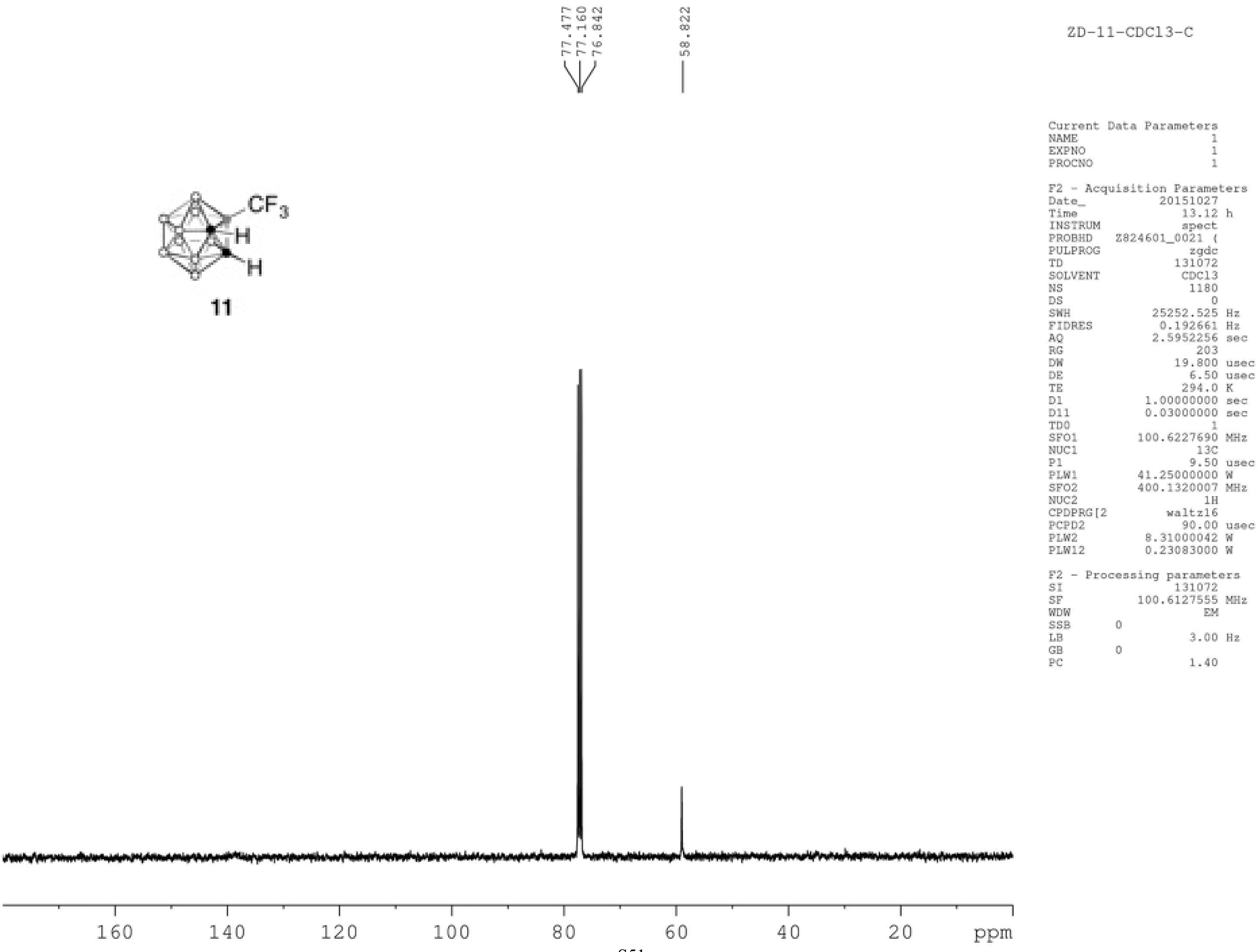
CF3
H
H
11
77.477
77.160
76.842
58.822
160
140
120
100
80
60
40
20
ppm
ZD-11-CDCl3-C
Current Data Parameters
NAME 1
EXPNO 1
PROCNO 1
F2 - Acquisition Parameters
Date_ 20151027
Time 13.12 h
INSTRUM spect
PROBHD Z824601_0021 (
PULPROG zgdc
TD 131072
SOLVENT CDCl3
NS 1180
DS 0
SWH 25252.525 Hz
FIDRES 0.192661 Hz
AQ 2.5952256 sec
RG 203
DW 19.800 usec
DE 6.50 usec
TE 294.0 K
D1 1.00000000 sec
D11 0.03000000 sec
TD0 1
SFO1 100.6227690 MHz
NUC1 13C
P1 9.50 usec
PLW1 41.25000000 W
SFO2 400.1320007 MHz
NUC2 1H
CPDPRG[2 waltz16
PCPD2 90.00 usec
PLW2 8.31000042 W
PLW12 0.23083000 W
F2 - Processing parameters
SI 131072
SF 100.6127555 MHz
WDW EM
SSB 0
LB 3.00 Hz
GB 0
PC 1.40

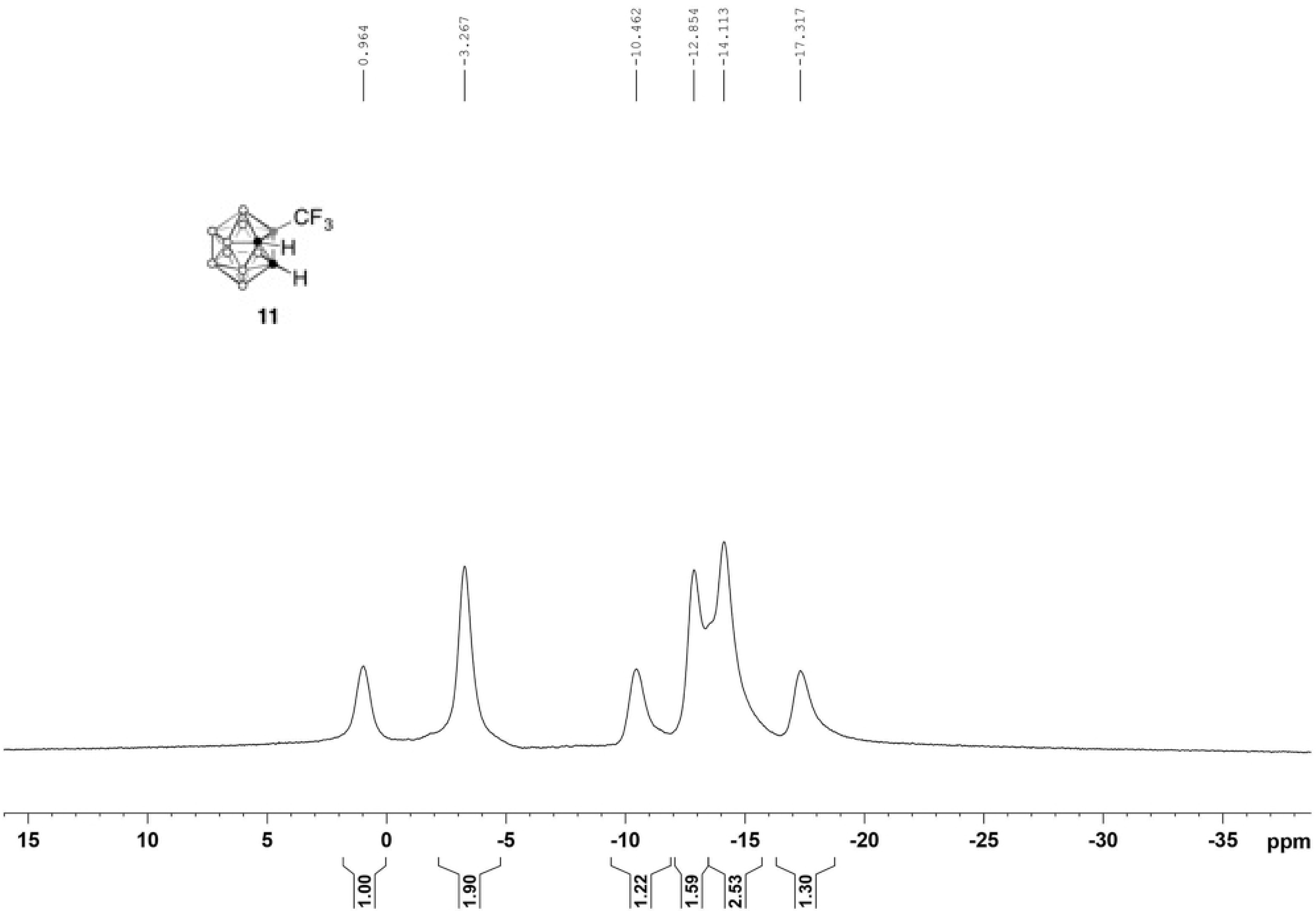
CF3
H
H
11
0.964
-3.267
-10.462
-12.854
-14.113
-17.317
15
10
5
0
-5
-10
-15
-20
-25
-30
-35
ppm
1.00
1.90
1.22
1.59
2.53
1.30

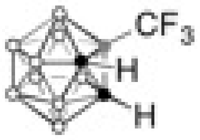

11

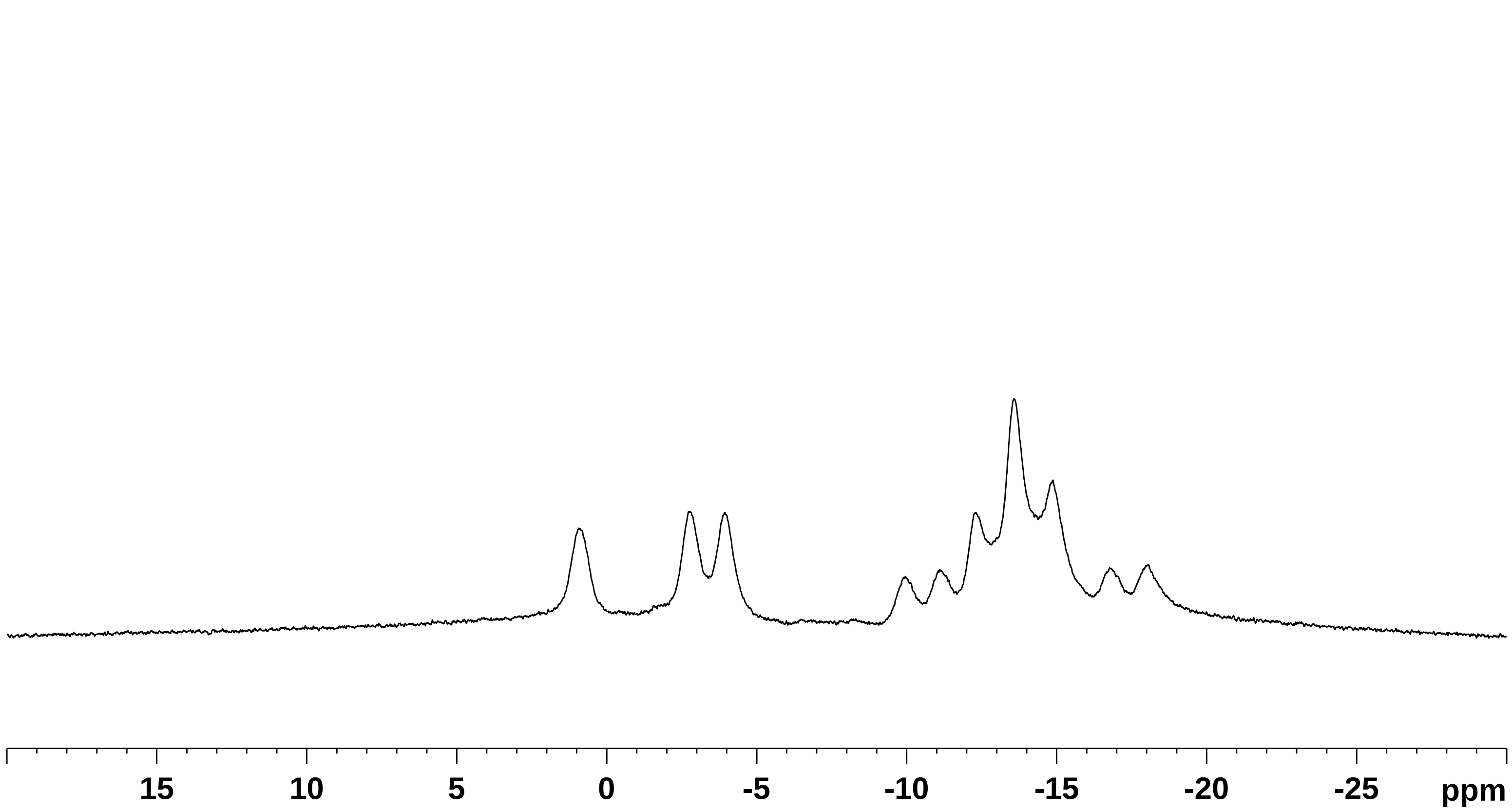

NAME	ZD-361-1-CDCl3-B(c)
EXPNO	1
PROCNO	1
Date_	20150730
Time	21.46
INSTRUM	spect
PROBHD	5 mm PABBO BB-
PULPROG	zg30
TD	65536
SOLVENT	CDCl3
NS	12
DS	0
SWH	25510.203 Hz
FIDRES	0.389255 Hz
AQ	1.2845556 sec
RG	456
DW	19.600 usec
DE	6.50 usec
TE	300.3 K
D1	5.00000000 sec
TD0	1

======== CHANNEL f1 ========

NUC1	11B
P1	7.60 usec
PL1	-3.00 dB
PL1W	55.13059616 W
SFO1	128.3968556 MHz
SI	32768
SF	128.3966640 MHz
WDW	EM
SSB	0
LB	3.00 Hz
GB	0
PC	1.40

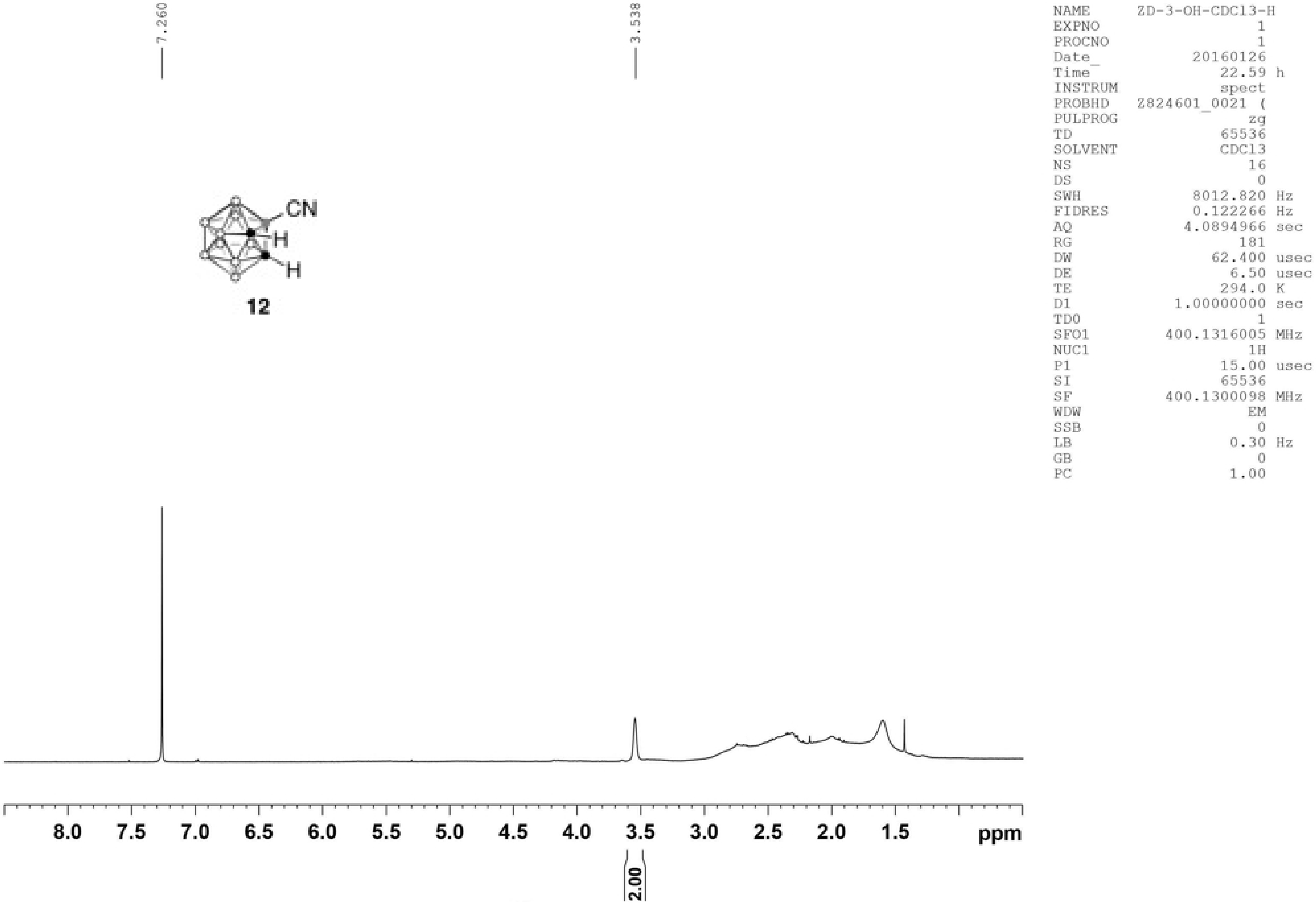
7.260
3.538
CN
H
H
12
NAME ZD-3-OH-CDCl3-H
EXPNO 1
PROCNO 1
Date_ 20160126
Time_ 22.59 h
INSTRUM spect
PROBHD Z824601_0021 (
PULPROG zg
TD 65536
SOLVENT CDCl3
NS 16
DS 0
SWH 8012.820 Hz
FIDRES 0.122266 Hz
AQ 4.0894966 sec
RG 181
DW 62.400 usec
DE 6.50 usec
TE 294.0 K
D1 1.00000000 sec
TD0 1
SFO1 400.1316005 MHz
NUC1 1H
P1 15.00 usec
SI 65536
SF 400.1300098 MHz
WDW EM
SSB 0
LB 0.30 Hz
GB 0
PC 1.00
8.0 7.5 7.0 6.5 6.0 5.5 5.0 4.5 4.0 3.5 3.0 2.5 2.0 1.5 ppm
2.00

ZD-3-12-CDCl3-C

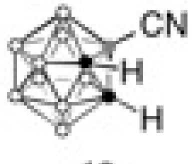

12

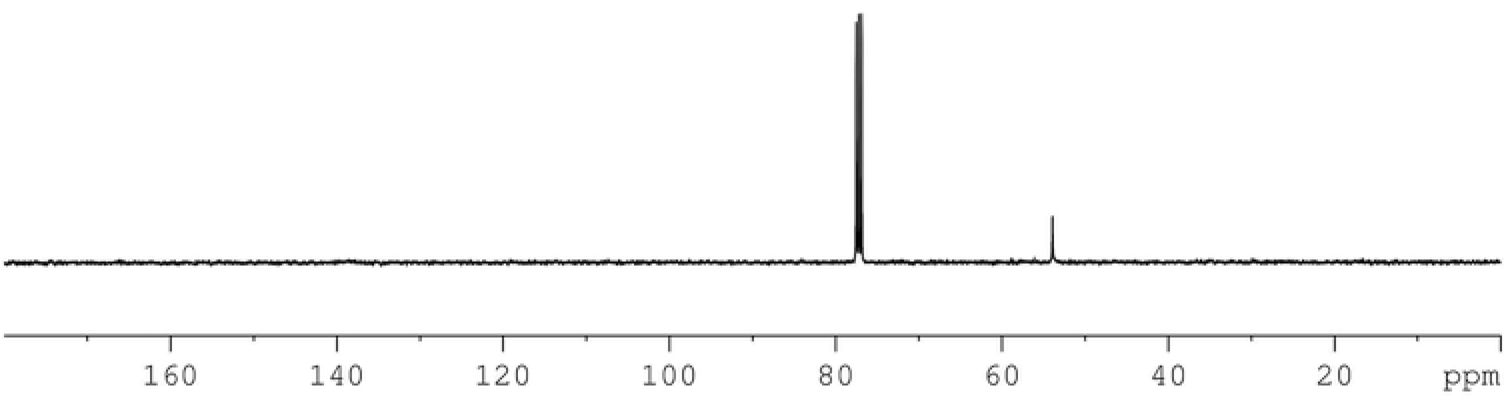

Current Data Parameters
NAME 1
EXPNO 1
PROCNO 1

F2 - Acquisition Parameters
Date_ 20160103
Time 21.29 h
INSTRUM spect
PROBHD Z824601_0021 (
PULPROG zgdc
TD 131072
SOLVENT CDCl3
NS 1180
DS 0
SWH 25252.525 Hz
FIDRES 0.192661 Hz
AQ 2.5952256 sec
RG 203
DW 19.800 usec
DE 6.50 usec
TE 294.0 K
D1 1.00000000 sec
D11 0.03000000 sec
TD0 1
SFO1 100.6227690 MHz
NUC1 13C
P1 9.50 usec
PLW1 41.25000000 W
SFO2 400.1320007 MHz
NUC2 1H
CPDPRG[2 waltz16
PCPD2 90.00 usec
PLW2 8.31000042 W
PLW12 0.23083000 W

F2 - Processing parameters
SI 131072
SF 100.6127555 MHz
WDW EM
SSB 0
LB 3.00 Hz
GB 0
PC 1.40

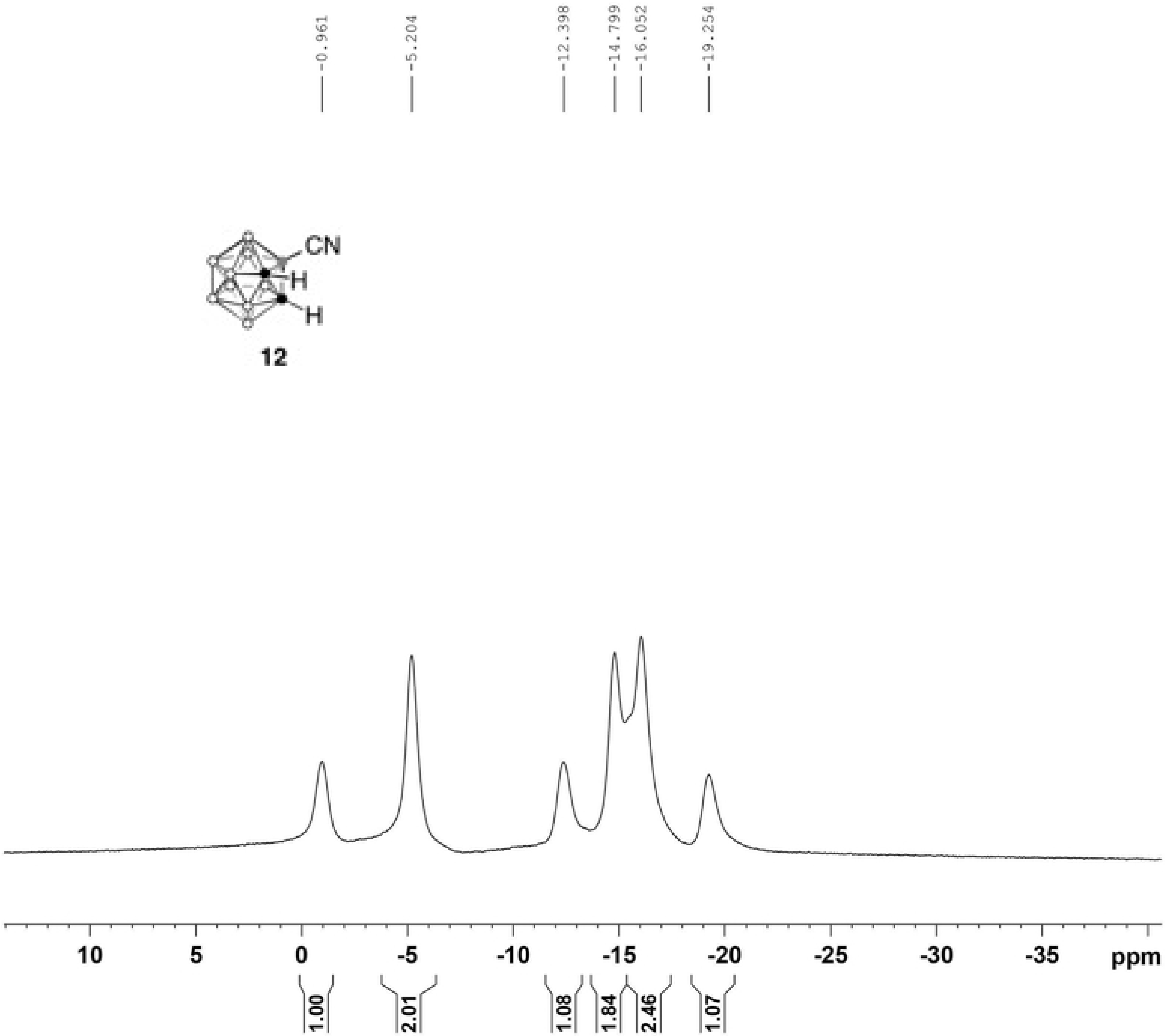

NAME	ZD-12-CDCl3-B(de)
EXPNO	1
PROCNO	1
Date_	20150630
Time	09.52
INSTRUM	spect
PROBHD	5 mm PABBO BB-
PULPROG	zgdc
TD	65536
SOLVENT	CDCl3
NS	23
DS	0
SWH	25510.203 Hz
FIDRES	0.389255 Hz
AQ	1.2845556 sec
RG	362
DW	19.600 usec
DE	6.50 usec
TE	300.8 K
D1	5.00000000 sec
D11	0.03000000 sec
TD0	1
======== CHANNEL f1 ========	
NUC1	11B
P1	7.60 usec
PL1	-3.00 dB
PL1W	55.13059616 W
SFO1	128.3968556 MHz
======== CHANNEL f2 ========	
CPDPRG2	waltz16
NUC2	1H
PCPD2	90.00 usec
PL2	-1.00 dB
PL12	15.16 dB
PL2W	13.56617069 W
PL12W	0.32844096 W
SFO2	400.1916008 MHz
SI	32768
SF	128.3969030 MHz
WDW	EM
SSB	0
LB	3.00 Hz
GB	0
PC	1.40

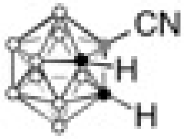

12

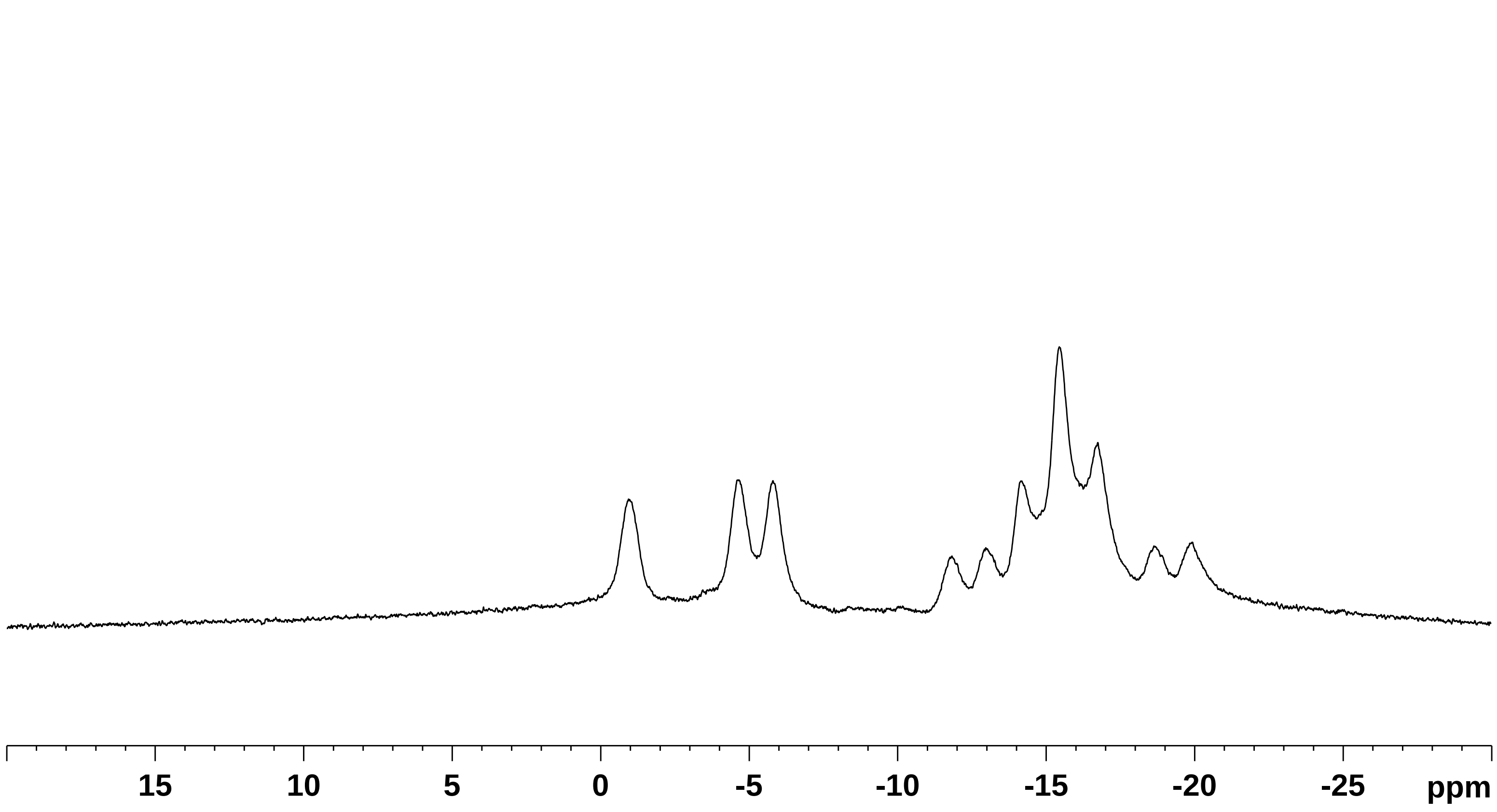

NAME	ZD-361-1-CDCl3-B(c)	
EXPNO	1	
PROCNO	1	
Date_	20150630	
Time	09.53	
INSTRUM	spect	
PROBHD	5 mm PABBO BB-	
PULPROG	zg30	
TD	65536	
SOLVENT	CDCl3	
NS	12	
DS	0	
SWH	25510.203	Hz
FIDRES	0.389255	Hz
AQ	1.2845556	sec
RG	456	
DW	19.600	usec
DE	6.50	usec
TE	300.3	K
D1	5.00000000	sec
TD0	1	

======== CHANNEL f1 ========

NUC1	11B	
P1	7.60	usec
PL1	-3.00	dB
PL1W	55.13059616	W
SFO1	128.3968556	MHz
SI	32768	
SF	128.3969035	MHz
WDW	EM	
SSB	0	
LB	3.00	Hz
GB	0	
PC	1.40	

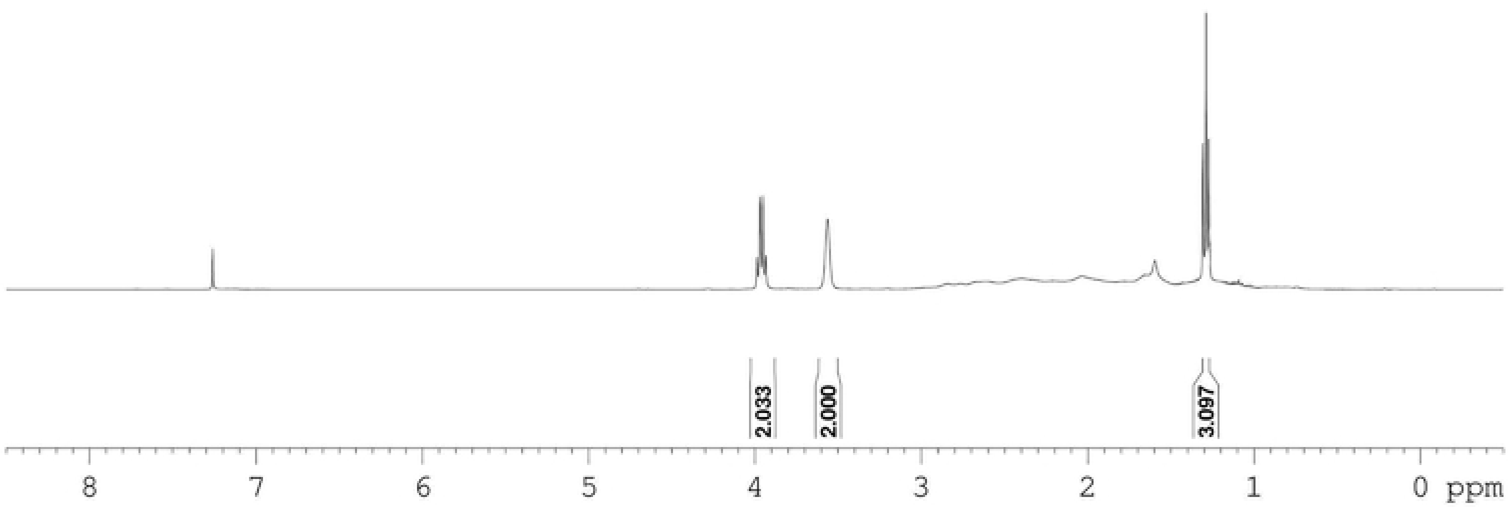

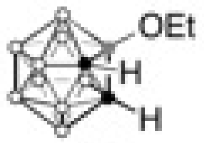

13

ZD-405-3-CDCl3-H

Bruker Advance III 400

NAME	ZD-405-3-CDCl3-H
EXPNO	1
PROCNO	1
Date_	20140730
Time	17.05
INSTRUM	spect
PROBHD	5 mm PADUL 13C
PULPROG	zg30
TD	65536
SOLVENT	CDCl3
NS	8
DS	2
SWH	8223.685 Hz
FIDRES	0.125483 Hz
AQ	3.9846387 sec
RG	203
DW	60.800 usec
DE	6.50 usec
TE	293.8 K
D1	2.00000000 sec
TD0	1

======== CHANNEL f1 ========

NUC1	1H
P1	14.83 usec
PL1	0.00 dB
PL1W	8.31434441 W
SFO1	400.1324710 MHz
SI	32768
SF	400.1300085 MHz
WDW	EM
SSB	0
LB	0.30 Hz
GB	0
PC	1.00

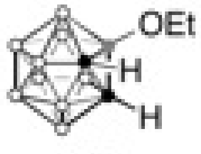

13

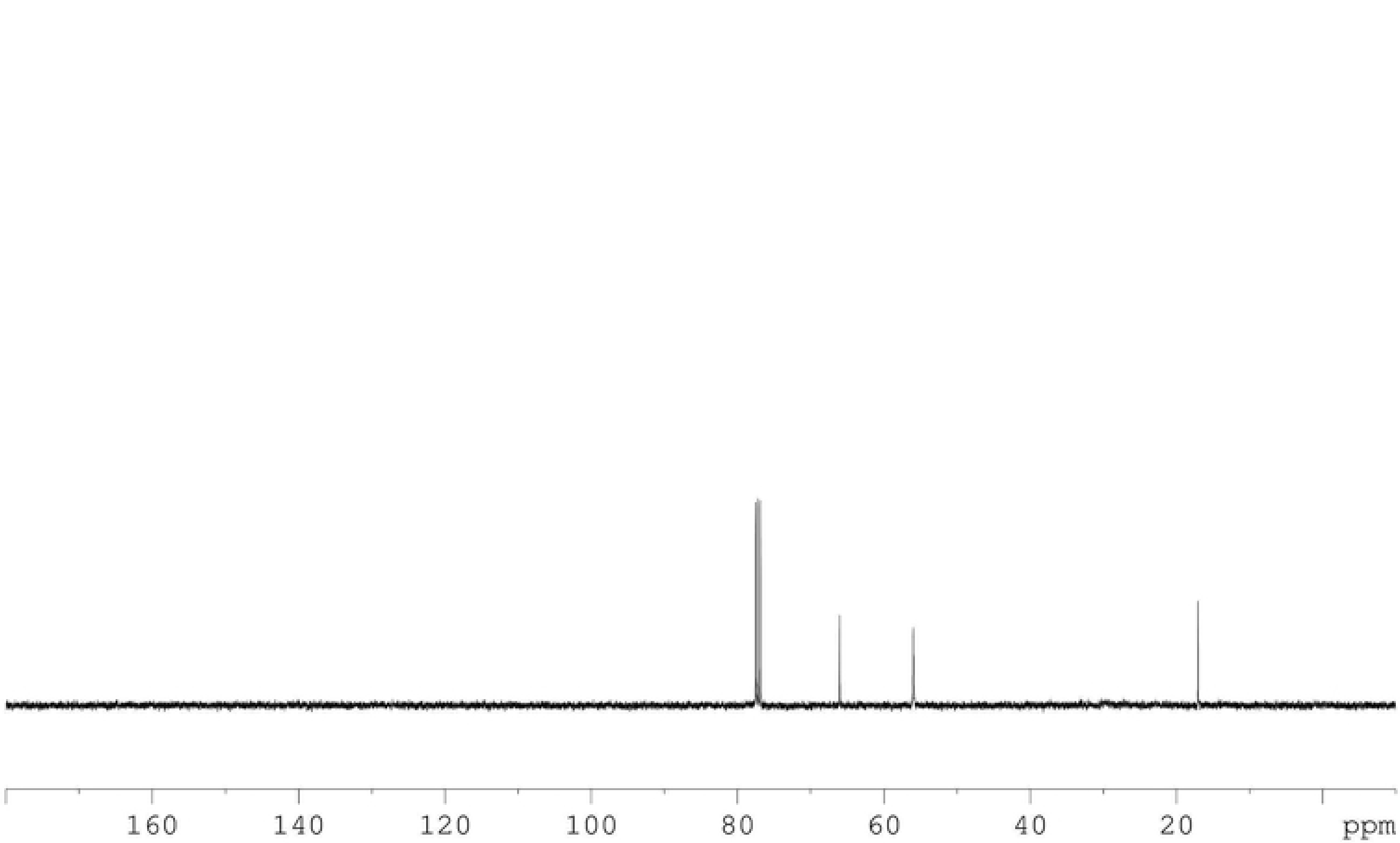

ZD-405-3-CDCl3-C

Bruker Advance III 400

```
NAME       ZD-405-3-CDCl3-C
EXPNO                     1
PROCNO                    1
Date_              20140730
Time                  22.04
INSTRUM               spect
PROBHD     5 mm PADUL 13C
PULPROG                zgdc
TD                   131072
SOLVENT               CDCl3
NS                      400
DS                        0
SWH              29761.904 Hz
FIDRES            0.227065 Hz
AQ               2.2020595 sec
RG                      203
DW                   16.800 usec
DE                     6.50 usec
TE                    294.4 K
D1               1.00000000 sec
D11              0.03000000 sec
TD0                       1

======== CHANNEL f1 ========
NUC1                    13C
P1                     9.68 usec
PL1                   -0.60 dB
PL1W            41.24164963 W
SFO1            100.6227690 MHz

======== CHANNEL f2 ========
CPDPRG2             waltz16
NUC2                     1H
PCPD2                 90.00 usec
PL2                    0.00 dB
PL12                  15.66 dB
PL2W             8.31434441 W
PL12W            0.22585411 W
SFO2            400.1320007 MHz
SI                   131072
SF              100.6127557 MHz
WDW                      EM
SSB                       0
LB                     1.00 Hz
GB                        0
PC                     1.40
```

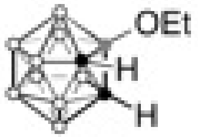

13

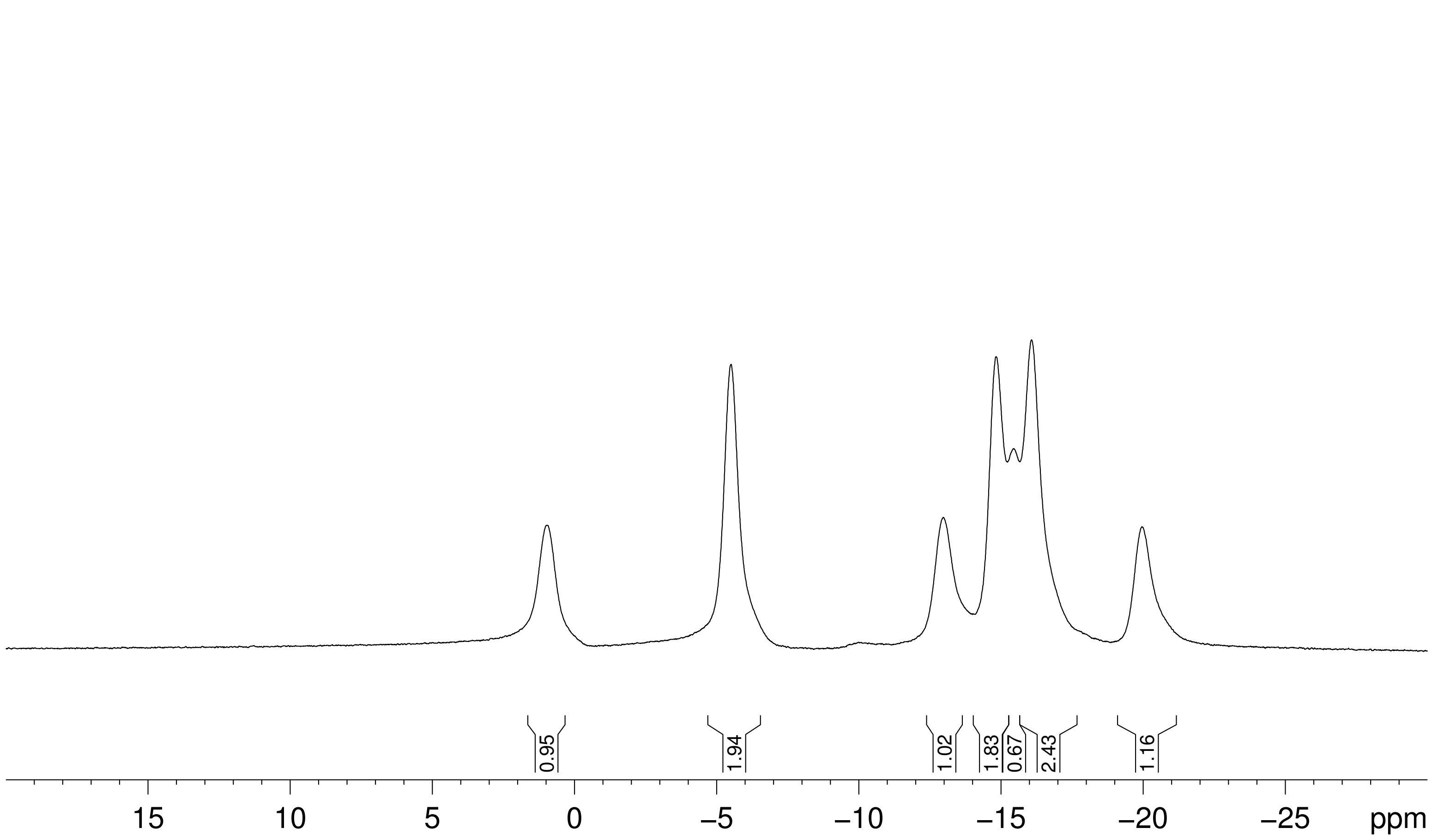

ZD-405-3-CDCl3-B(de)

NAME	ZD-405-3-CDCl3-B(de)	
EXPNO	1	
PROCNO	1	
Date_	20140730	
Time	20.29	
INSTRUM	spect	
PROBHD	5 mm PABBO BB-	
PULPROG	zgdc	
TD	65536	
SOLVENT	CDCl3	
NS	8	
DS	0	
SWH	25510.203	Hz
FIDRES	0.389255	Hz
AQ	1.2845556	sec
RG	256	
DW	19.600	usec
DE	6.50	usec
TE	298.6	K
D1	5.00000000	sec
D11	0.03000000	sec
TD0	1	

======== CHANNEL f1 ========

NUC1	11B	
P1	7.60	usec
PL1	-3.00	dB
PL1W	55.13059616	W
SFO1	128.3968556	MHz

======== CHANNEL f2 ========

CPDPRG2	waltz16	
NUC2	1H	
PCPD2	90.00	usec
PL2	-1.00	dB
PL12	15.16	dB
PL2W	13.56617069	W
PL12W	0.32844096	W
SFO2	400.1916008	MHz
SI	32768	
SF	128.3968847	MHz
WDW	EM	
SSB	0	
LB	3.00	Hz
GB	0	
PC	1.40	

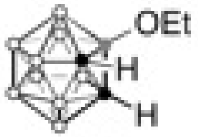

13

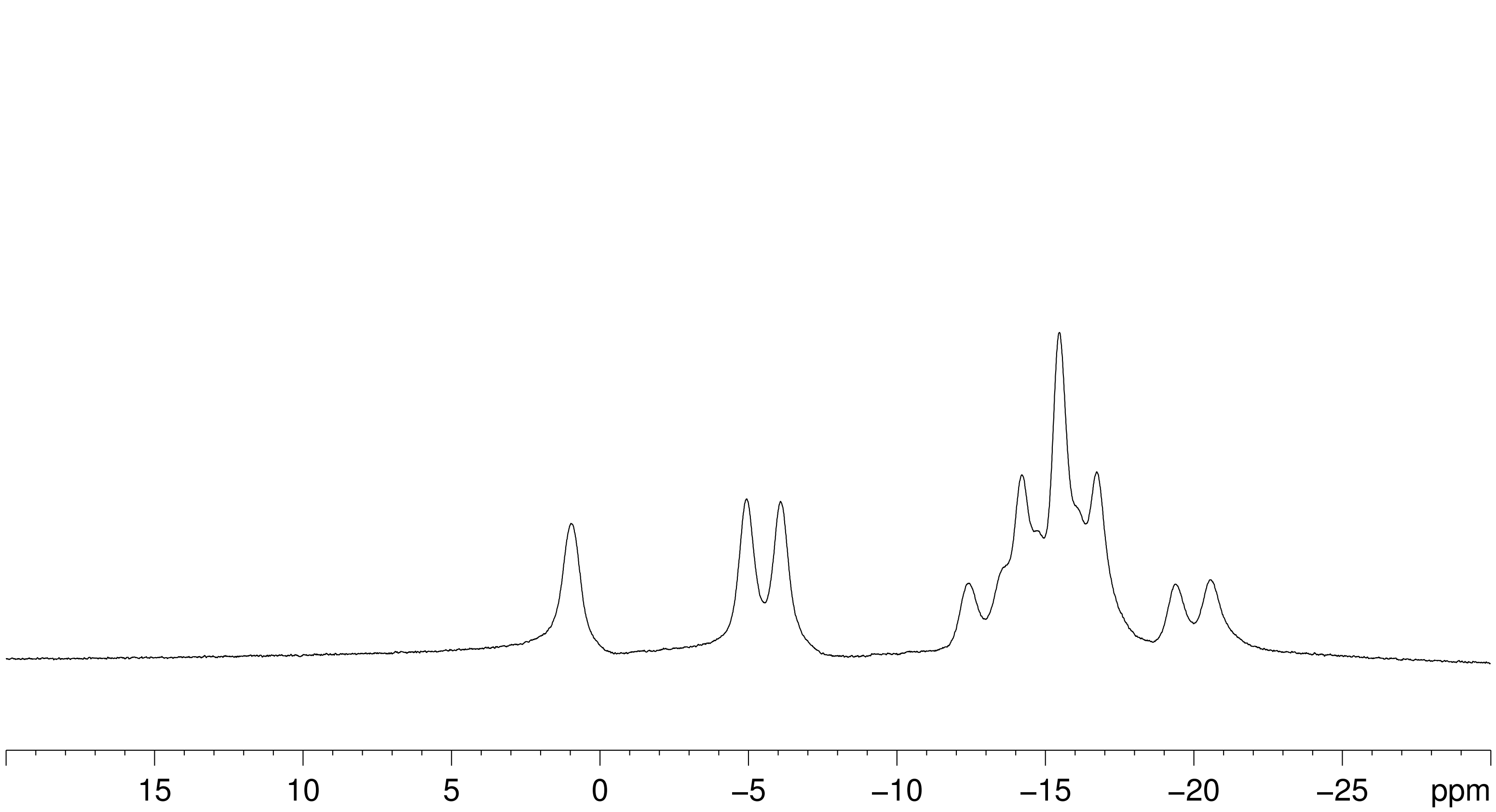

ZD-405-3-CDCl3-B(c)

```
NAME       ZD-405-3-CDCl3-B(c)
EXPNO                        1
PROCNO                       1
Date_                 20140730
Time                     20.31
INSTRUM                  spect
PROBHD        5 mm PABBO BB-
PULPROG                   zg30
TD                       65536
SOLVENT                  CDCl3
NS                          16
DS                           0
SWH                  25510.203 Hz
FIDRES                0.389255 Hz
AQ                   1.2845556 sec
RG                         161
DW                      19.600 usec
DE                        6.50 usec
TE                       298.2 K
D1                  5.00000000 sec
TD0                          1

======== CHANNEL f1 ========
NUC1                       11B
P1                        7.60 usec
PL1                      -3.00 dB
PL1W               55.13059616 W
SFO1               128.3968556 MHz
SI                       32768
SF                 128.3968865 MHz
WDW                         EM
SSB                          0
LB                        3.00 Hz
GB                           0
PC                        1.40
```

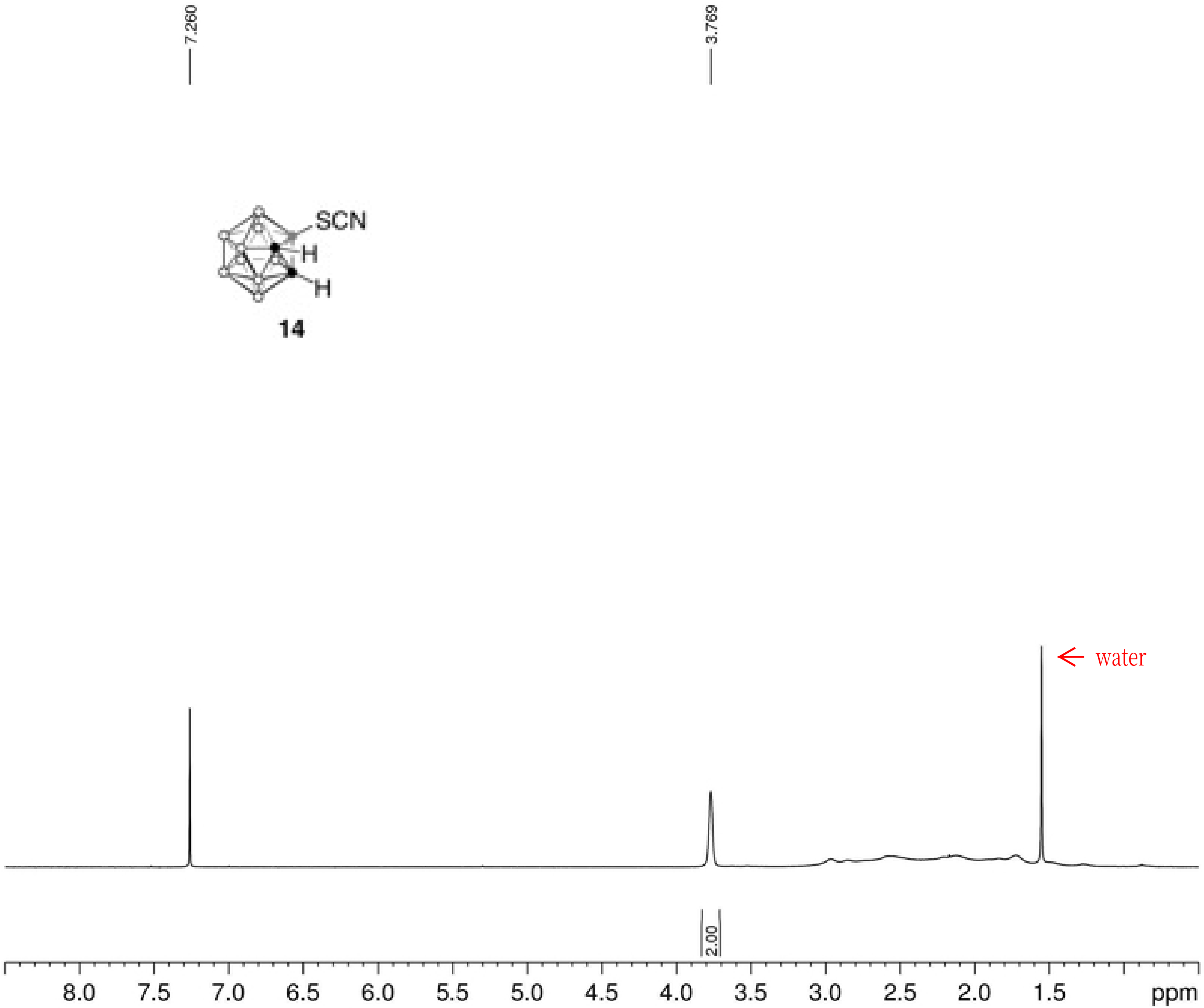

14

ZD-3-SCN-CDCl3-H

Current Data Parameters
NAME ZD-3-SCN-CDCl3-H
EXPNO 2
PROCNO 1

F2 - Acquisition Parameters
Date_ 20160127
Time 13.55 h
INSTRUM spect
PROBHD Z108618_0257 (
PULPROG zg30
TD 65536
SOLVENT CDCl3
NS 8
DS 2
SWH 8012.820 Hz
FIDRES 0.122266 Hz
AQ 4.0894465 sec
RG 203
DW 62.400 usec
DE 6.50 usec
TE 298.2 K
D1 1.00000000 sec
TD0 1
SFO1 400.2324714 MHz
NUC1 1H
P1 12.80 usec
PLW1 13.56000042 W

F2 - Processing parameters
SI 65536
SF 400.2300101 MHz
WDW EM
SSB 0
LB 0.30 Hz
GB 0
PC 1.00

ZD-3-SCN-CDCl3-C

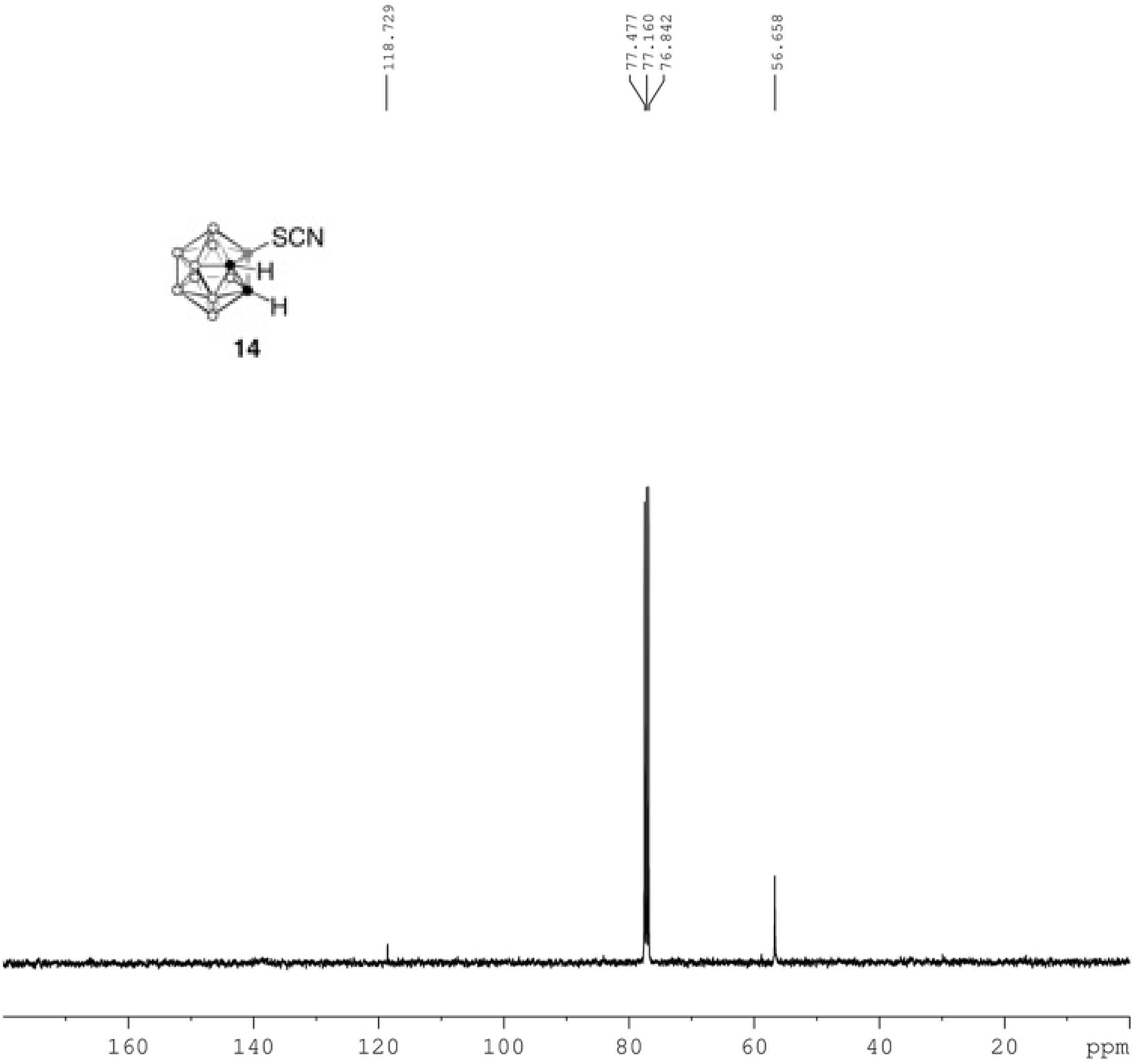


```
Current Data Parameters
NAME      ZD-3-SCN-CDCl3-C
EXPNO                    1
PROCNO                   1

F2 - Acquisition Parameters
Date_             20160127
Time                 14.08 h
INSTRUM              spect
PROBHD   Z824601_0021 (
PULPROG               zgdc
TD                  131072
SOLVENT              CDCl3
NS                    1180
DS                       0
SWH              25252.525 Hz
FIDRES            0.192661 Hz
AQ               2.5952256 sec
RG                     203
DW                  19.800 usec
DE                    6.50 usec
TE                   294.0 K
D1              1.00000000 sec
D11             0.03000000 sec
TD0                      1
SFO1           100.6227690 MHz
NUC1                   13C
P1                    9.50 usec
PLW1           41.25000000 W
SFO2           400.1320007 MHz
NUC2                    1H
CPDPRG[2           waltz16
PCPD2                90.00 usec
PLW2            8.31000042 W
PLW12           0.23083000 W

F2 - Processing parameters
SI                  131072
SF             100.6127555 MHz
WDW                     EM
SSB      0
LB                    3.00 Hz
GB       0
PC                    1.40
```

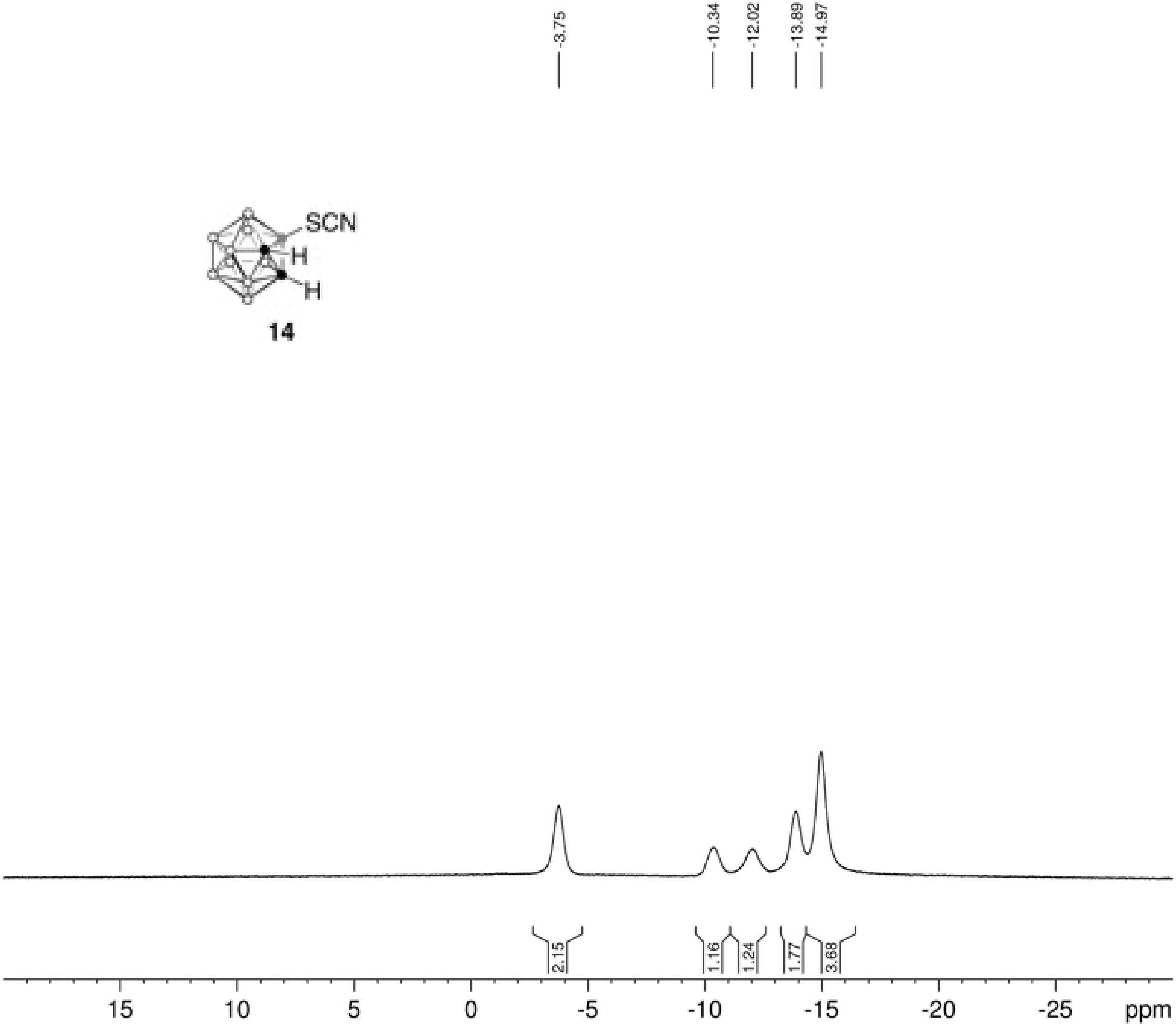

ZD-3-SCN-CDCl3-B(de)

```
Current Data Parameters
NAME      ZD-3-SCN-CDCl3-B(de)
EXPNO                        1
PROCNO                       1

F2 - Acquisition Parameters
Date_              20160127
Time                  13.58 h
INSTRUM               spect
PROBHD    Z108618_0257 (
PULPROG                zgdc
TD                    65536
SOLVENT               CDCl3
NS                        8
DS                        4
SWH               24038.461 Hz
FIDRES             0.366798 Hz
AQ                1.3631488 sec
RG                      322
DW                   20.800 usec
DE                     6.50 usec
TE                    298.4 K
D1               2.00000000 sec
D11              0.03000000 sec
TD0                       1
SFO1            128.4096890 MHz
NUC1                    11B
P1                     7.50 usec
PLW1            55.09999847 W
SFO2            400.2316009 MHz
NUC2                     1H
CPDPRG[2            waltz16
PCPD2                 90.00 usec
PLW2            13.56000042 W
PLW12            0.27428001 W

F2 - Processing parameters
SI                    32768
SF              128.4097615 MHz
WDW                      EM
SSB      0
LB                     1.00 Hz
GB       0
PC                     1.40
```

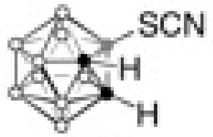

14

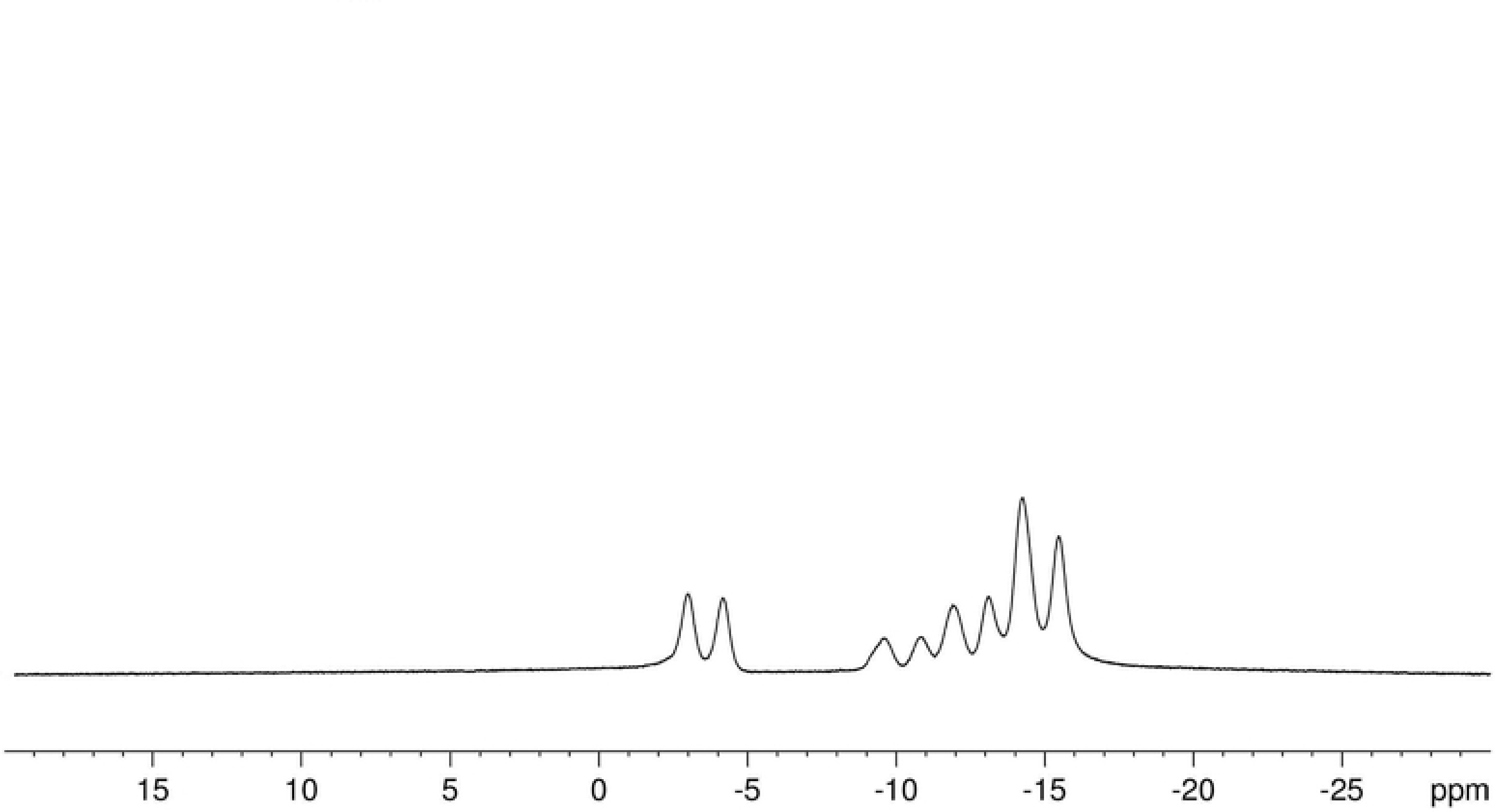

ZD-3-SCN-CDCl3-B(c)

```
Current Data Parameters
NAME       ZD-3-SCN-CDCl3-B(c)
EXPNO                        1
PROCNO                       1

F2 - Acquisition Parameters
Date_             20160127
Time                 13.59 h
INSTRUM              spect
PROBHD    Z108618_0257 (
PULPROG                 zg
TD                   65536
SOLVENT              CDCl3
NS                      24
DS                       2
SWH              24038.461 Hz
FIDRES            0.366798 Hz
AQ               1.3631488 sec
RG                     101
DW                  20.800 usec
DE                    6.50 usec
TE                   298.3 K
D1              2.00000000 sec
TD0                      1
SFO1           128.4096890 MHz
NUC1                   11B
P1                    7.50 usec
PLW1           55.09999847 W

F2 - Processing parameters
SI                   32768
SF             128.4097430 MHz
WDW                     EM
SSB       0
LB                    1.00 Hz
GB        0
PC                    1.40
```

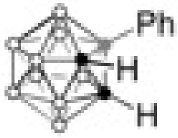

15a

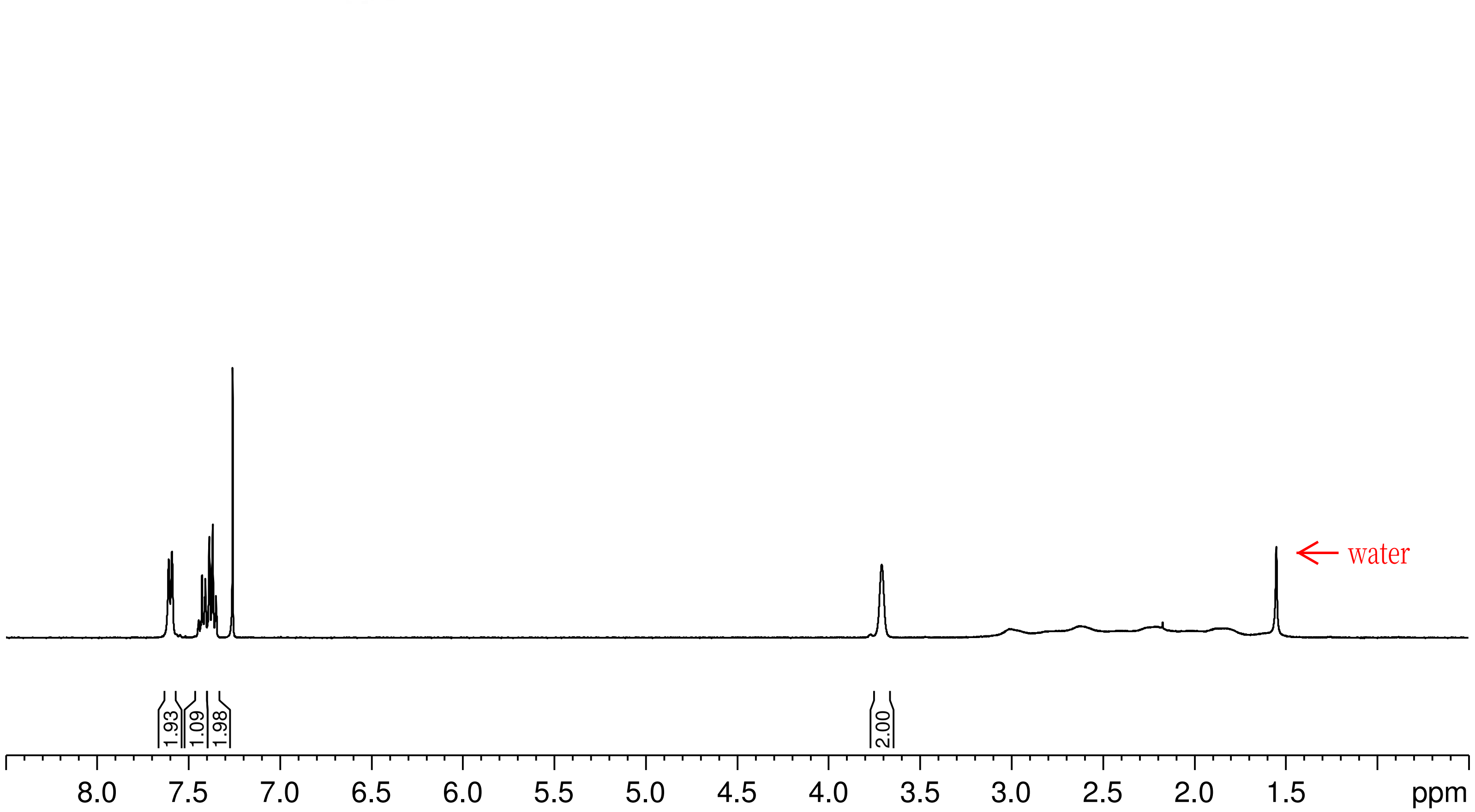

Current Data Parameters
NAME ZD-3-Ph-CDCl3-H
EXPNO 1
PROCNO 1

F2 - Acquisition Parameters
Date_ 20160321
Time 11.41 h
INSTRUM spect
PROBHD Z108618_0257 (
PULPROG zg30
TD 65536
SOLVENT CDCl3
NS 4
DS 2
SWH 8012.820 Hz
FIDRES 0.122266 Hz
AQ 4.0894465 sec
RG 203
DW 62.400 usec
DE 6.50 usec
TE 294.6 K
D1 1.00000000 sec
TD0 1
SFO1 400.2324714 MHz
NUC1 1H
P1 12.80 usec
PLW1 13.56000042 W

F2 - Processing parameters
SI 65536
SF 400.2300098 MHz
WDW EM
SSB 0
LB 0.30 Hz
GB 0
PC 1.00

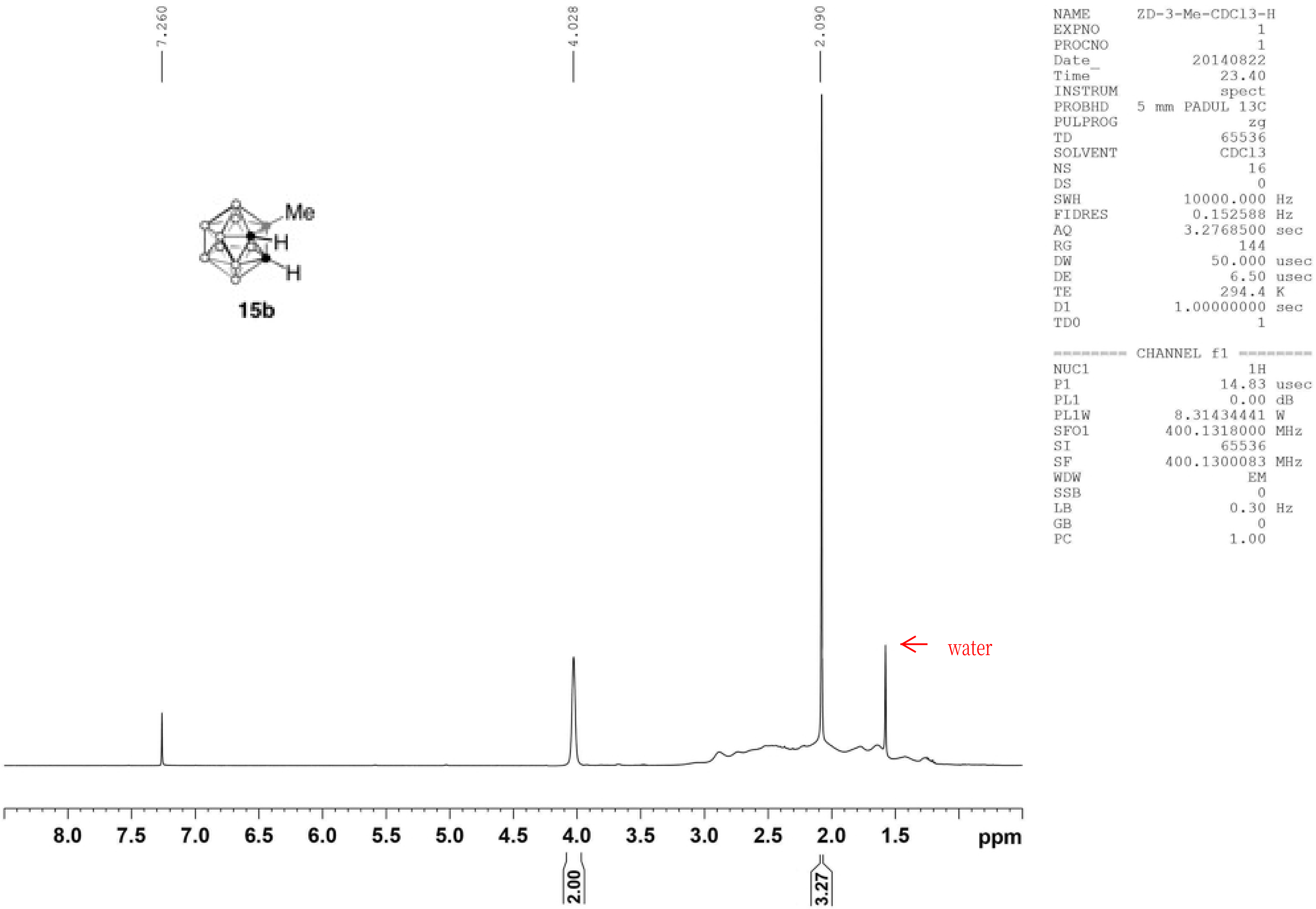
7.260
4.028
2.090
Me
H
H
15b
water
8.0
7.5
7.0
6.5
6.0
5.5
5.0
4.5
4.0
3.5
3.0
2.5
2.0
1.5
ppm
2.00
3.27
NAME ZD-3-Me-CDCl3-H
EXPNO 1
PROCNO 1
Date_ 20140822
Time 23.40
INSTRUM spect
PROBHD 5 mm PADUL 13C
PULPROG zg
TD 65536
SOLVENT CDCl3
NS 16
DS 0
SWH 10000.000 Hz
FIDRES 0.152588 Hz
AQ 3.2768500 sec
RG 144
DW 50.000 usec
DE 6.50 usec
TE 294.4 K
D1 1.00000000 sec
TD0 1
======== CHANNEL f1 ========
NUC1 1H
P1 14.83 usec
PL1 0.00 dB
PL1W 8.31434441 W
SFO1 400.1318000 MHz
SI 65536
SF 400.1300083 MHz
WDW EM
SSB 0
LB 0.30 Hz
GB 0
PC 1.00

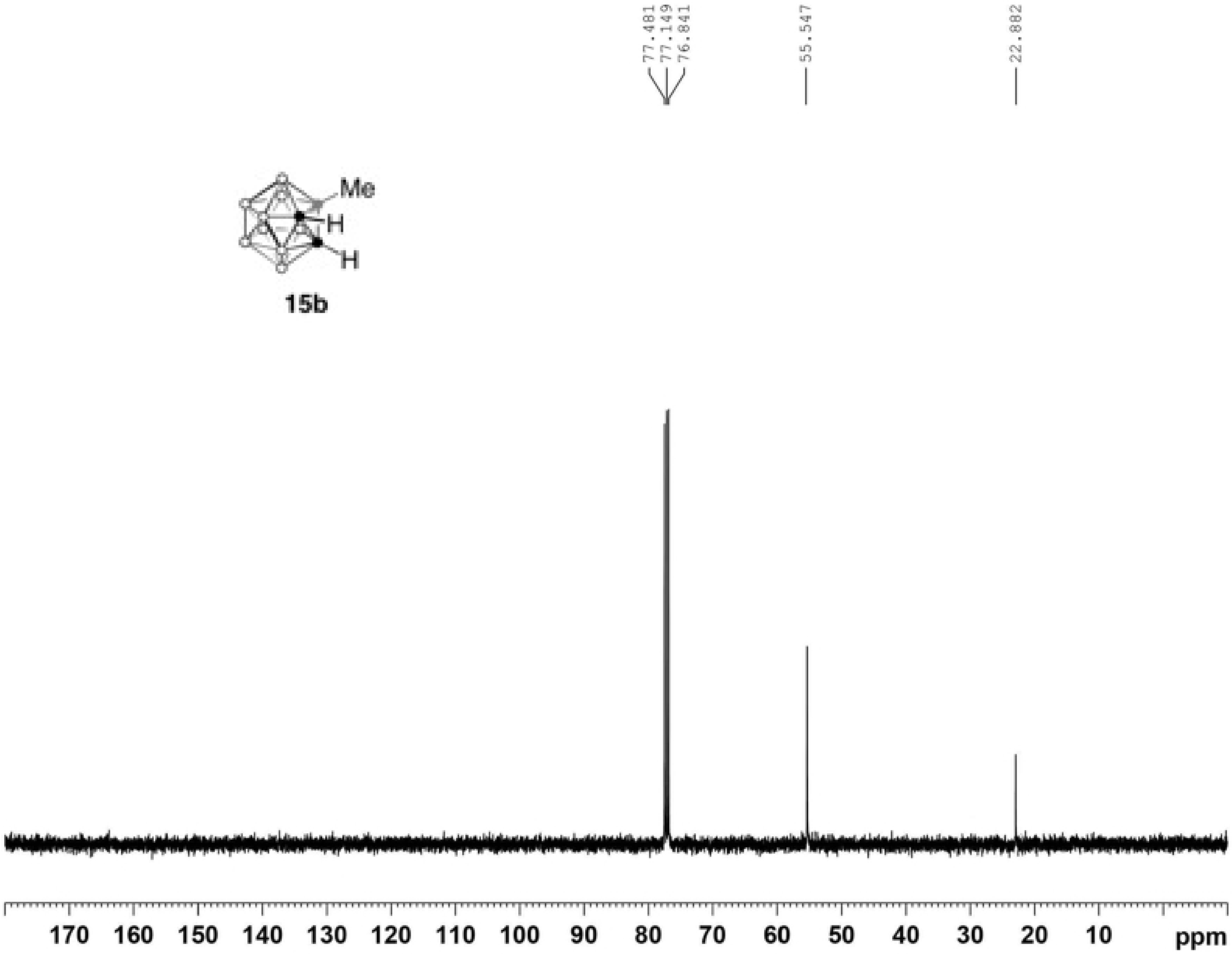

NAME	ZD-3-Me-CDCl3-C
EXPNO	1
PROCNO	1
Date_	20140822
Time	23.42
INSTRUM	spect
PROBHD	5 mm PADUL 13C
PULPROG	zgdc
TD	131072
SOLVENT	CDCl3
NS	666
DS	0
SWH	29761.904 Hz
FIDRES	0.227065 Hz
AQ	2.2020595 sec
RG	203
DW	16.800 usec
DE	6.50 usec
TE	294.4 K
D1	1.00000000 sec
D11	0.03000000 sec
TD0	1
======== CHANNEL f1 ========	
NUC1	13C
P1	9.68 usec
PL1	-0.60 dB
PL1W	41.24164963 W
SFO1	100.6227690 MHz
======== CHANNEL f2 ========	
CPDPRG2	waltz16
NUC2	1H
PCPD2	90.00 usec
PL2	0.00 dB
PL12	15.66 dB
PL2W	8.31434441 W
PL12W	0.22585411 W
SFO2	400.1320007 MHz
SI	131072
SF	100.6127554 MHz
WDW	EM
SSB	0
LB	1.00 Hz
GB	0
PC	1.40

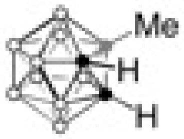

15b

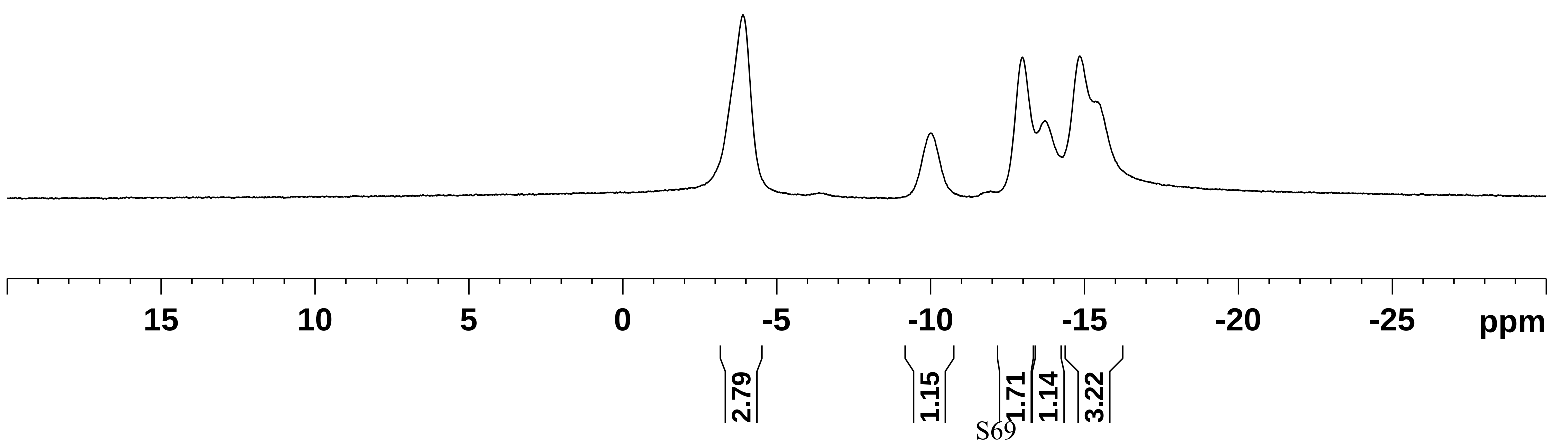


```
NAME       ZD-3-Me-CDCl3-B(de)
EXPNO                    1
PROCNO                   1
Date_             20140822
Time                 23.25
INSTRUM              spect
PROBHD     5 mm PABBO BB-
PULPROG               zgdc
TD                   65536
SOLVENT              CDCl3
NS                       4
DS                       0
SWH              25510.203 Hz
FIDRES            0.389255 Hz
AQ               1.2845556 sec
RG                     287
DW                  19.600 usec
DE                    6.50 usec
TE                   295.8 K
D1              5.00000000 sec
D11             0.03000000 sec
TD0                      1

======== CHANNEL f1 ========
NUC1                   11B
P1                    7.60 usec
PL1                  -3.00 dB
PL1W           55.13059616 W
SFO1           128.3968556 MHz

======== CHANNEL f2 ========
CPDPRG2            waltz16
NUC2                    1H
PCPD2                90.00 usec
PL2                  -1.00 dB
PL12                 15.16 dB
PL2W           13.56617069 W
PL12W           0.32844096 W
SFO2           400.1916008 MHz
SI                   32768
SF             128.3967486 MHz
WDW                     EM
SSB                      0
LB                    3.00 Hz
GB                       0
PC                    1.40
```

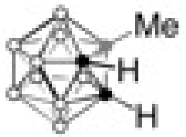

15b

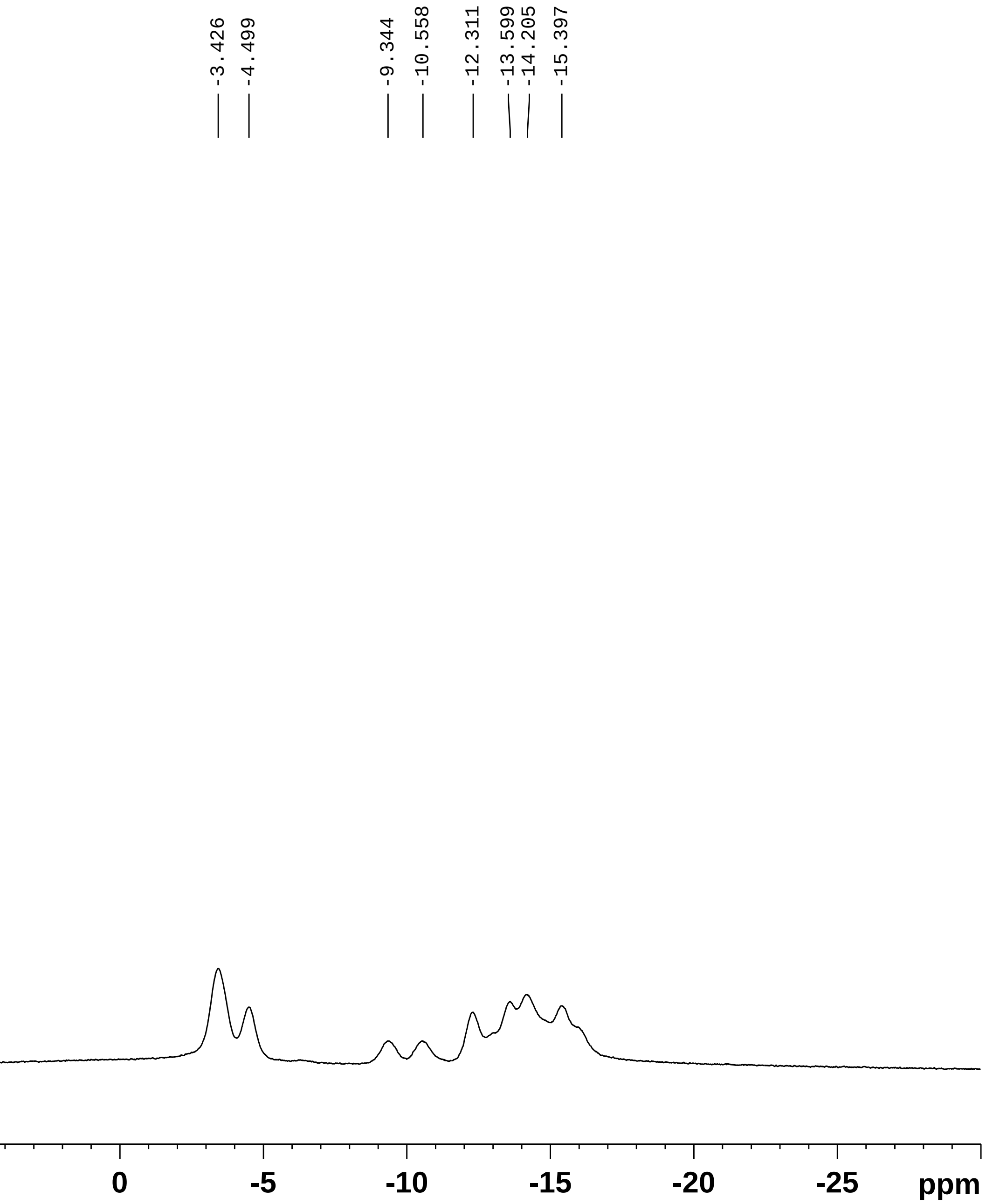

NAME	ZD-3-Me-CDCl3-B(c)	
EXPNO	1	
PROCNO	1	
Date_	20140822	
Time	23.28	
INSTRUM	spect	
PROBHD	5 mm PABBO BB-	
PULPROG	zg30	
TD	65536	
SOLVENT	CDCl3	
NS	8	
DS	0	
SWH	25510.203	Hz
FIDRES	0.389255	Hz
AQ	1.2845556	sec
RG	161	
DW	19.600	usec
DE	6.50	usec
TE	295.5	K
D1	5.00000000	sec
TD0	1	

======== CHANNEL f1 ========

NUC1	11B	
P1	7.60	usec
PL1	-3.00	dB
PL1W	55.13059616	W
SFO1	128.3968556	MHz
SI	32768	
SF	128.3967436	MHz
WDW	EM	
SSB	0	
LB	3.00	Hz
GB	0	
PC	1.40	

ZD-719-CDCl3-H

Bruker Advance III 400

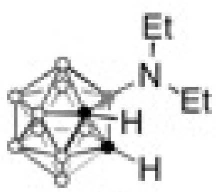

16a

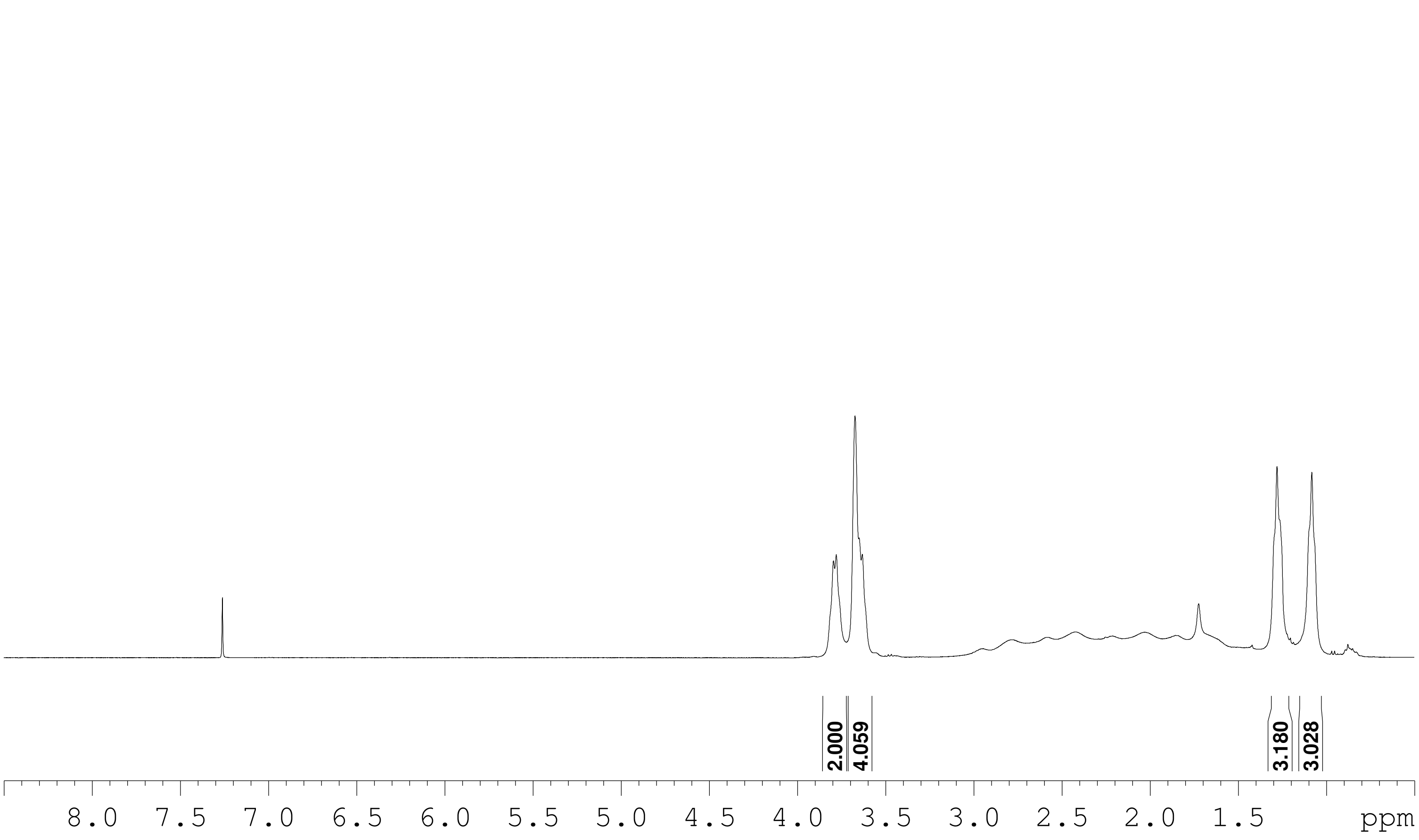

NAME	ZD-719-CDCl3-H	
EXPNO	1	
PROCNO	1	
Date_	20140806	
Time	17.00	
INSTRUM	spect	
PROBHD	5 mm PADUL 13C	
PULPROG	zg30	
TD	65536	
SOLVENT	CDCl3	
NS	8	
DS	2	
SWH	8223.685	Hz
FIDRES	0.125483	Hz
AQ	3.9846387	sec
RG	71.8	
DW	60.800	usec
DE	6.50	usec
TE	293.9	K
D1	2.00000000	sec
TD0	1	

======== CHANNEL f1 ========

NUC1	1H	
P1	14.83	usec
PL1	0.00	dB
PL1W	8.31434441	W
SFO1	400.1324710	MHz
SI	32768	
SF	400.1300084	MHz
WDW	EM	
SSB	0	
LB	0.30	Hz
GB	0	
PC	1.00	

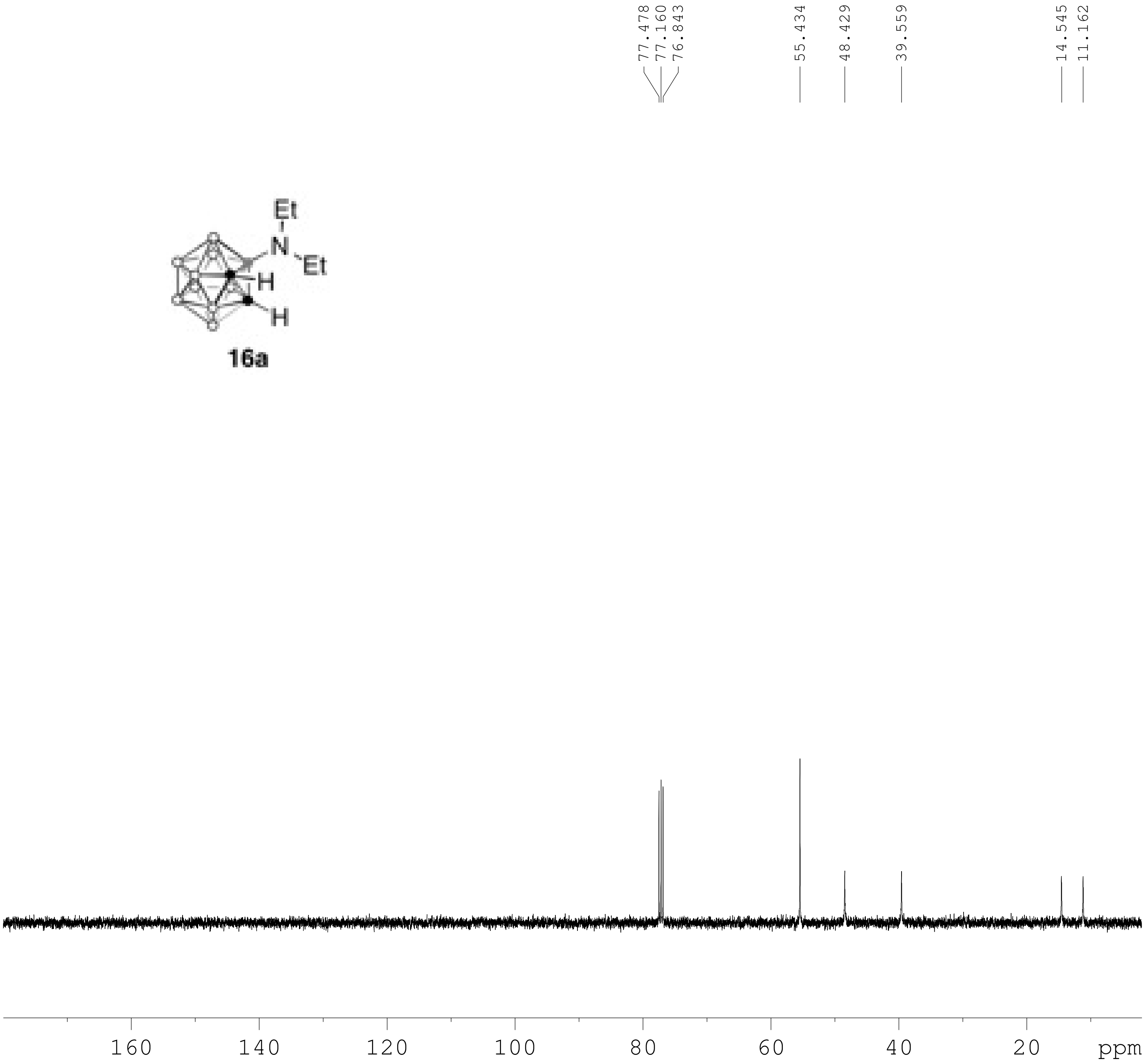

16a

ZD-719-CDCl3-C

Bruker Advance III 400

NAME	ZD-719-CDCl3-C
EXPNO	1
PROCNO	1
Date_	20140806
Time	17.04
INSTRUM	spect
PROBHD	5 mm PADUL 13C
PULPROG	zgdc
TD	131072
SOLVENT	CDCl3
NS	80
DS	0
SWH	29761.904 Hz
FIDRES	0.227065 Hz
AQ	2.2020595 sec
RG	203
DW	16.800 usec
DE	6.50 usec
TE	294.1 K
D1	1.00000000 sec
D11	0.03000000 sec
TD0	1
======== CHANNEL f1 ========	
NUC1	13C
P1	9.68 usec
PL1	-0.60 dB
PL1W	41.24164963 W
SFO1	100.6227690 MHz
======== CHANNEL f2 ========	
CPDPRG2	waltz16
NUC2	1H
PCPD2	90.00 usec
PL2	0.00 dB
PL12	15.66 dB
PL2W	8.31434441 W
PL12W	0.22585411 W
SFO2	400.1320007 MHz
SI	131072
SF	100.6127570 MHz
WDW	EM
SSB	0
LB	1.00 Hz
GB	0
PC	1.40

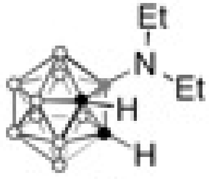

16a

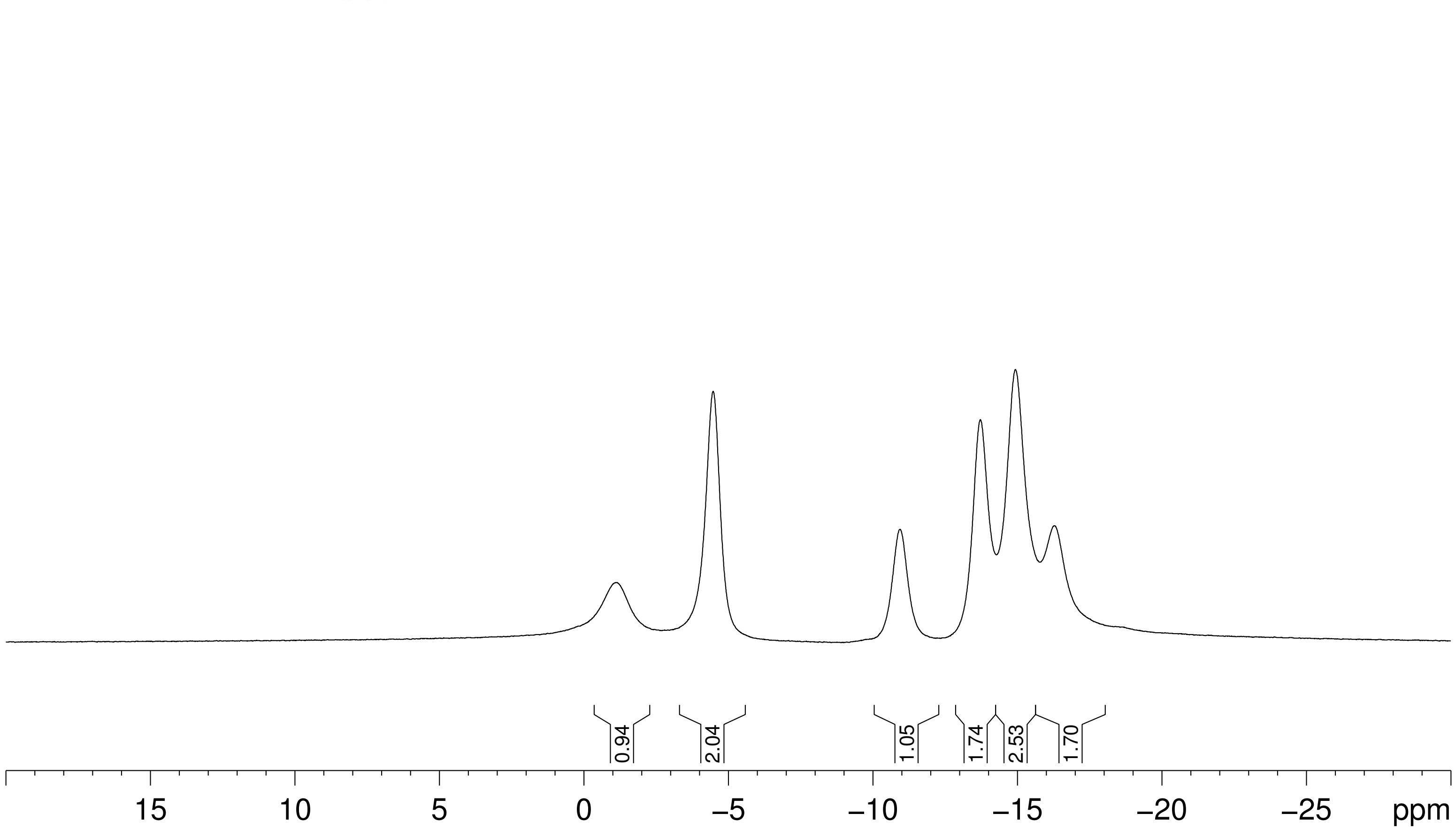

ZD-719-CDCl3-B(de)

NAME	ZD-719-CDCl3-B(de)
EXPNO	1
PROCNO	1
Date_	20140806
Time	17.11
INSTRUM	spect
PROBHD	5 mm PABBO BB-
PULPROG	zgdc
TD	65536
SOLVENT	CDCl3
NS	12
DS	0
SWH	25510.203 Hz
FIDRES	0.389255 Hz
AQ	1.2845556 sec
RG	203
DW	19.600 usec
DE	6.50 usec
TE	298.3 K
D1	5.00000000 sec
D11	0.03000000 sec
TD0	1

======== CHANNEL f1 ========

NUC1	11B
P1	7.60 usec
PL1	-3.00 dB
PL1W	55.13059616 W
SFO1	128.3968556 MHz

======== CHANNEL f2 ========

CPDPRG2	waltz16
NUC2	1H
PCPD2	90.00 usec
PL2	-1.00 dB
PL12	15.16 dB
PL2W	13.56617069 W
PL12W	0.32844096 W
SFO2	400.1916008 MHz
SI	32768
SF	128.3968847 MHz
WDW	EM
SSB	0
LB	3.00 Hz
GB	0
PC	1.40

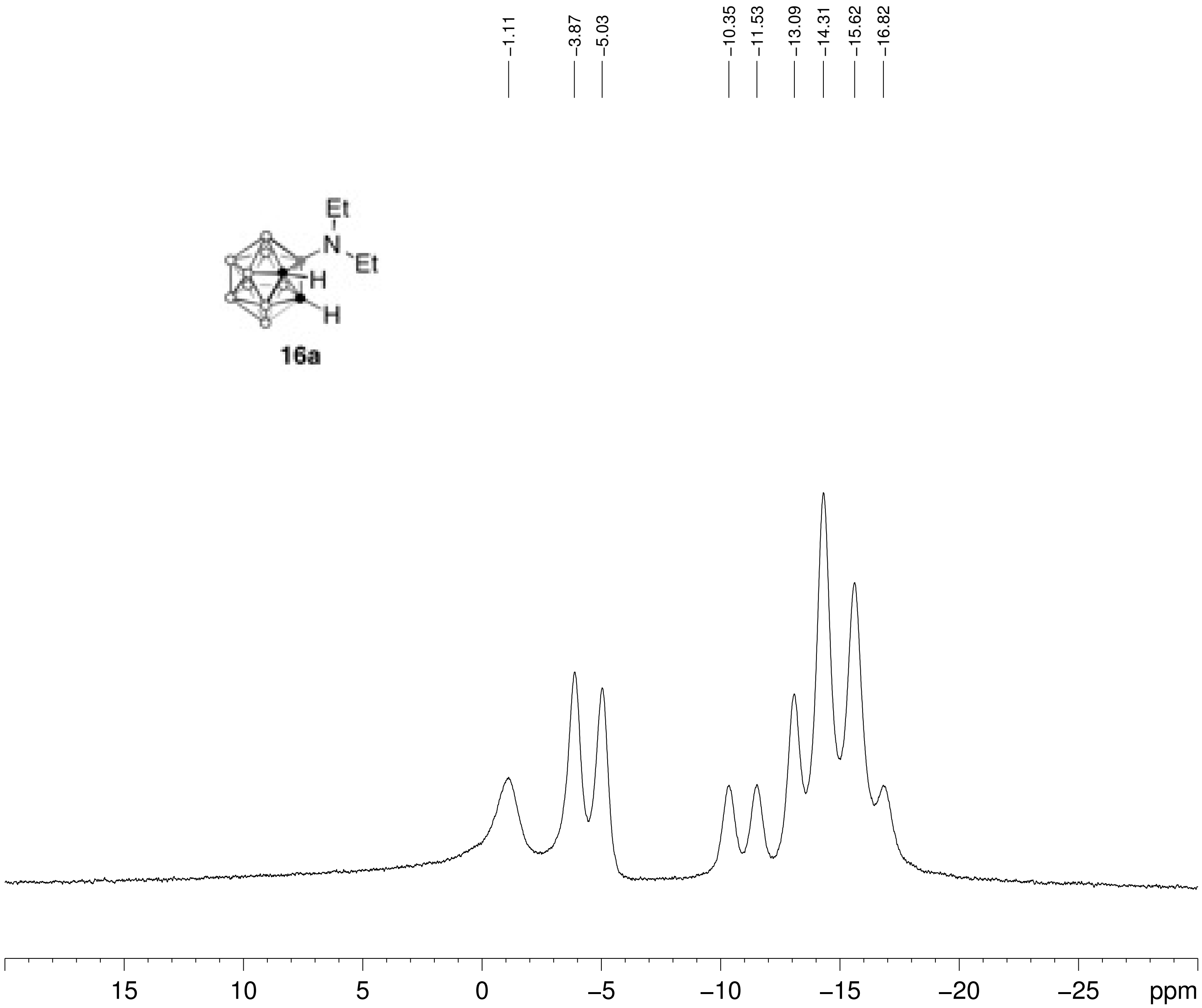

ZD-719-CDCl3-B(c)

NAME	ZD-719-CDCl3-B(c)
EXPNO	1
PROCNO	1
Date_	20140806
Time	17.12
INSTRUM	spect
PROBHD	5 mm PABBO BB-
PULPROG	zg30
TD	65536
SOLVENT	CDCl3
NS	4
DS	0
SWH	25510.203 Hz
FIDRES	0.389255 Hz
AQ	1.2845556 sec
RG	161
DW	19.600 usec
DE	6.50 usec
TE	298.0 K
D1	5.00000000 sec
TD0	1

======== CHANNEL f1 ========

NUC1	11B
P1	7.60 usec
PL1	-3.00 dB
PL1W	55.13059616 W
SFO1	128.3968556 MHz
SI	32768
SF	128.3968865 MHz
WDW	EM
SSB	0
LB	3.00 Hz
GB	0
PC	1.40

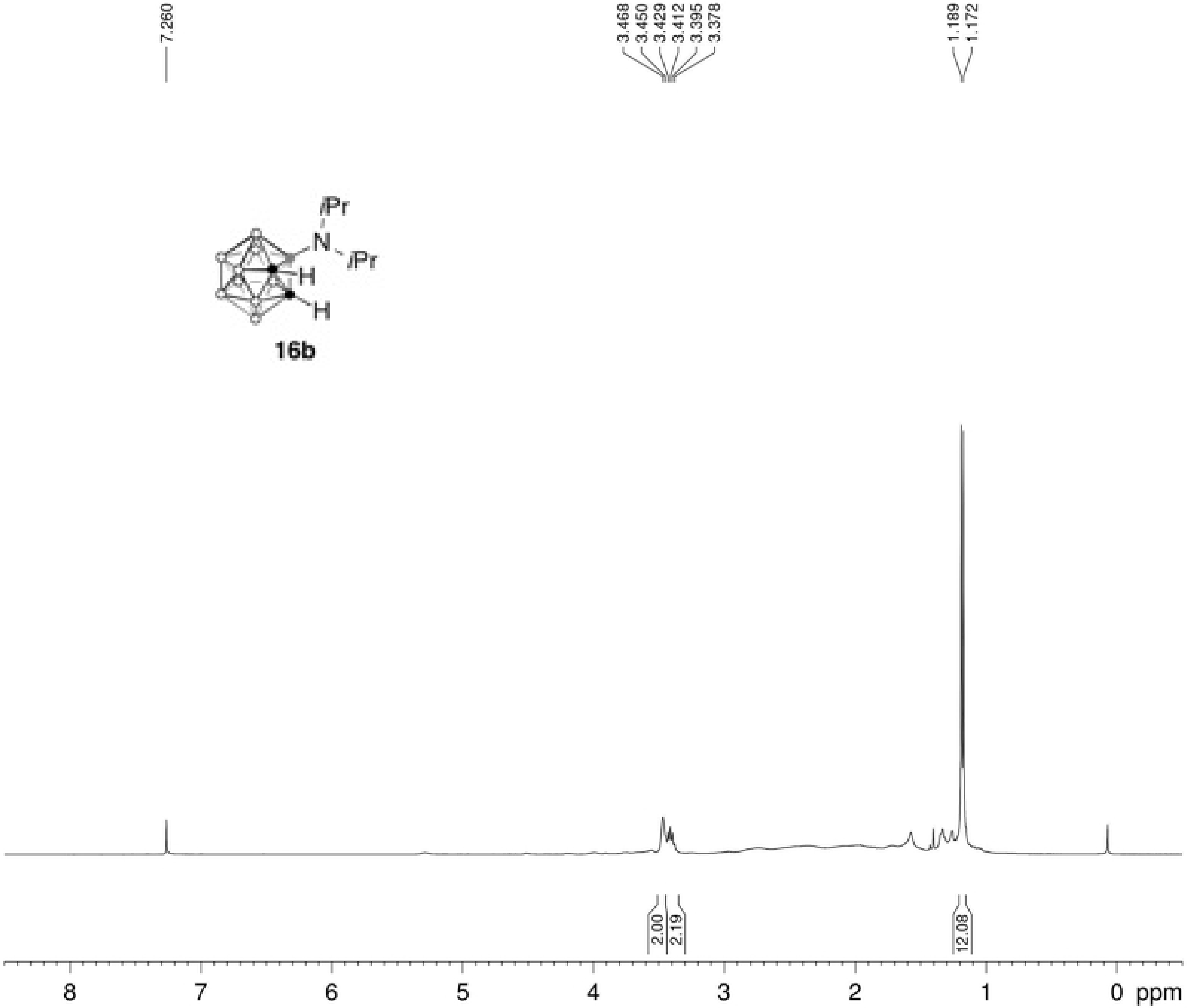

16b

ZD-720-CDCl3-H

NAME ZD-720-CDCl3-H
EXPNO 1
PROCNO 1
Date_ 20140807
Time 11.25
INSTRUM spect
PROBHD 5 mm PABBO BB-
PULPROG zg30
TD 65536
SOLVENT CDCl3
NS 8
DS 2
SWH 8223.685 Hz
FIDRES 0.125483 Hz
AQ 3.9846387 sec
RG 71.8
DW 60.800 usec
DE 6.50 usec
TE 297.9 K
D1 1.00000000 sec
TD0 1

======== CHANNEL f1 =====
NUC1 1H
P1 14.00 usec
PL1 -1.00 dB
PL1W 13.56617069 W
SFO1 400.1924713 MHz
SI 32768
SF 400.1900149 MHz
WDW EM
SSB 0
LB 0.30 Hz
GB 0
PC 1.00

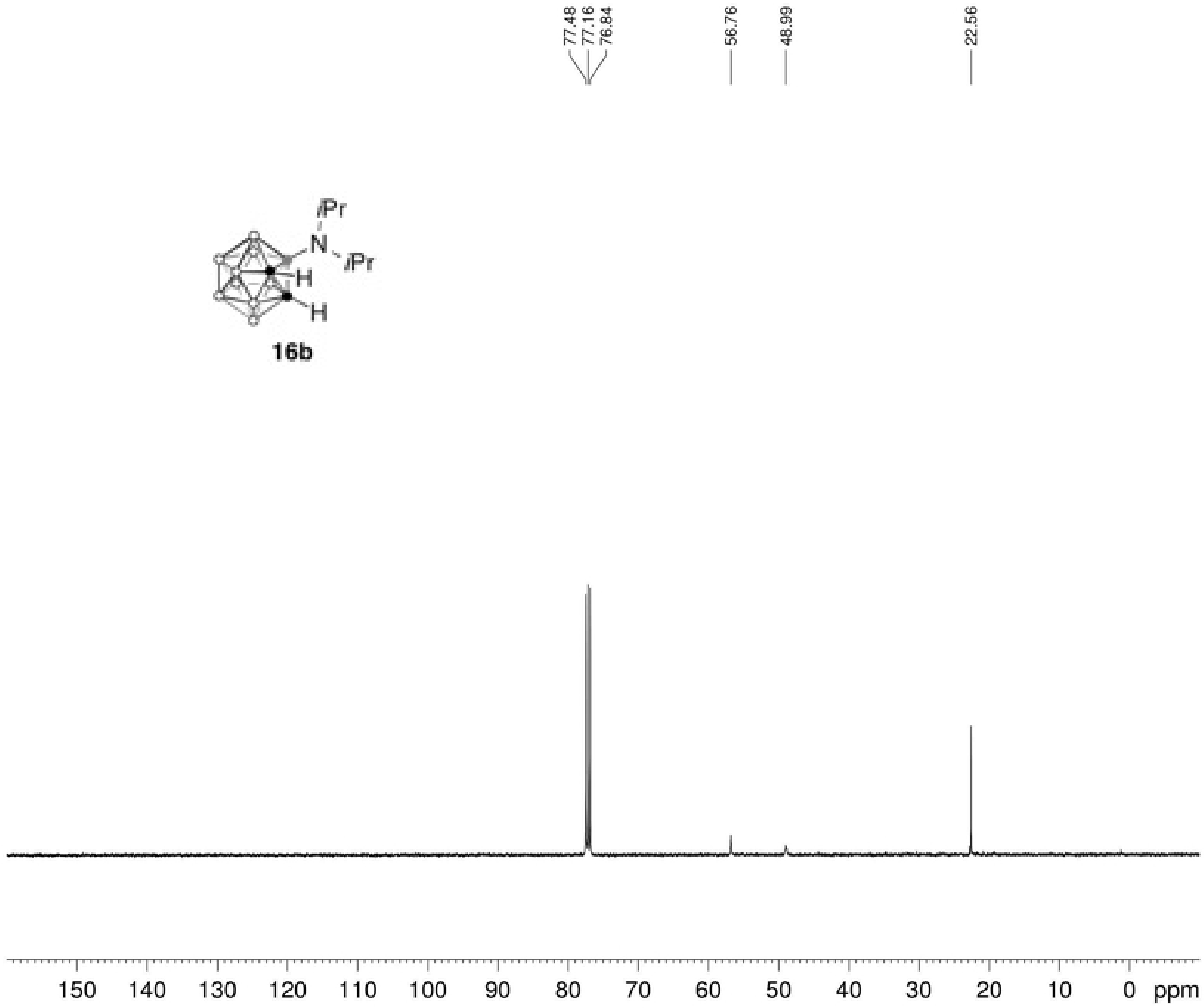

ZD-720-CDCl3-C

```
NAME       ZD-720-CDCl3-C
EXPNO                   1
PROCNO                  1
Date_            20140807
Time                11.30
INSTRUM             spect
PROBHD     5 mm PABBO BB-
PULPROG           zgpg30
TD                 65536
SOLVENT            CDCl3
NS                   684
DS                     4
SWH            24038.461 Hz
FIDRES          0.366798 Hz
AQ             1.3631988 sec
RG                   181
DW                20.800 usec
DE                  6.50 usec
TE                 298.3 K
D1            2.00000000 sec
D11           0.03000000 sec
TD0                    1

======== CHANNEL f1 ========
NUC1                 13C
P1                  9.90 usec
PL1                -2.00 dB
PL1W         55.33689499 W
SFO1        100.6379183 MHz

======== CHANNEL f2 ========
CPDPRG2           waltz16
NUC2                  1H
PCPD2              90.00 usec
PL2                -1.00 dB
PL12               15.16 dB
PL13               18.62 dB
PL2W         13.56617069 W
PL12W         0.32844096 W
PL13W         0.14806664 W
SFO2        400.1916008 MHz
SI                 32768
SF          100.6278433 MHz
WDW                   EM
SSB                    0
LB                  1.00 Hz
GB                     0
PC                  1.40
```

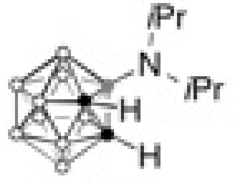

16b

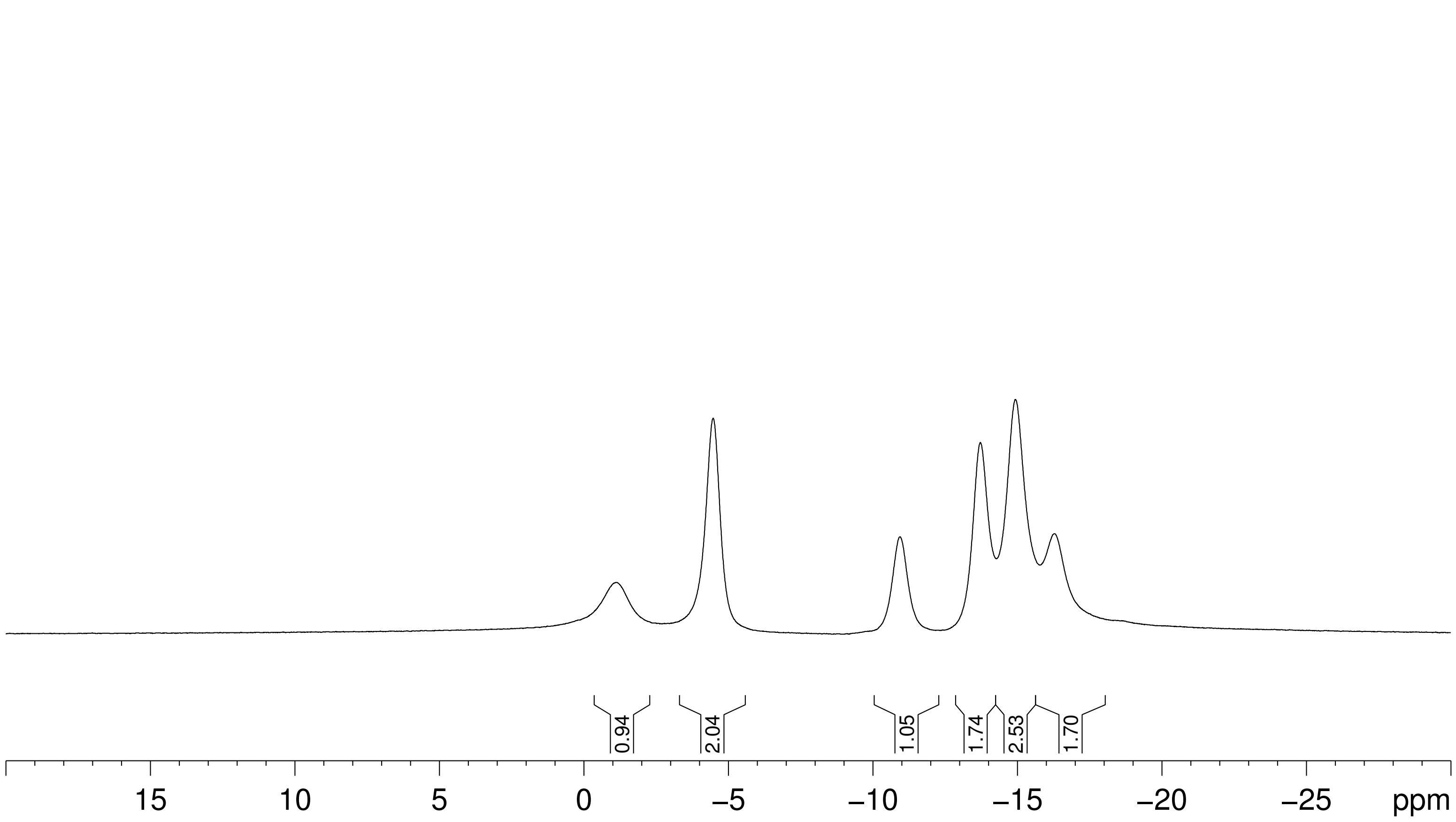

ZD-720-CDCl3-B(de)

```
NAME       ZD-720-CDCl3-B(de)
EXPNO                       1
PROCNO                      1
Date_                20140807
Time                    17.11
INSTRUM                 spect
PROBHD      5 mm PABBO BB-
PULPROG                  zgdc
TD                      65536
SOLVENT                 CDCl3
NS                         12
DS                          0
SWH                25510.203 Hz
FIDRES              0.389255 Hz
AQ                 1.2845556 sec
RG                        203
DW                     19.600 usec
DE                       6.50 usec
TE                      298.3 K
D1                 5.00000000 sec
D11                0.03000000 sec
TD0                         1

======== CHANNEL f1 ========
NUC1                      11B
P1                       7.60 usec
PL1                     -3.00 dB
PL1W              55.13059616 W
SFO1              128.3968556 MHz

======== CHANNEL f2 ========
CPDPRG2               waltz16
NUC2                       1H
PCPD2                   90.00 usec
PL2                     -1.00 dB
PL12                    15.16 dB
PL2W              13.56617069 W
PL12W              0.32844096 W
SFO2              400.1916008 MHz
SI                      32768
SF                128.3968847 MHz
WDW                        EM
SSB                         0
LB                       3.00 Hz
GB                          0
PC                       1.40
```

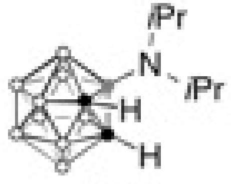

16b

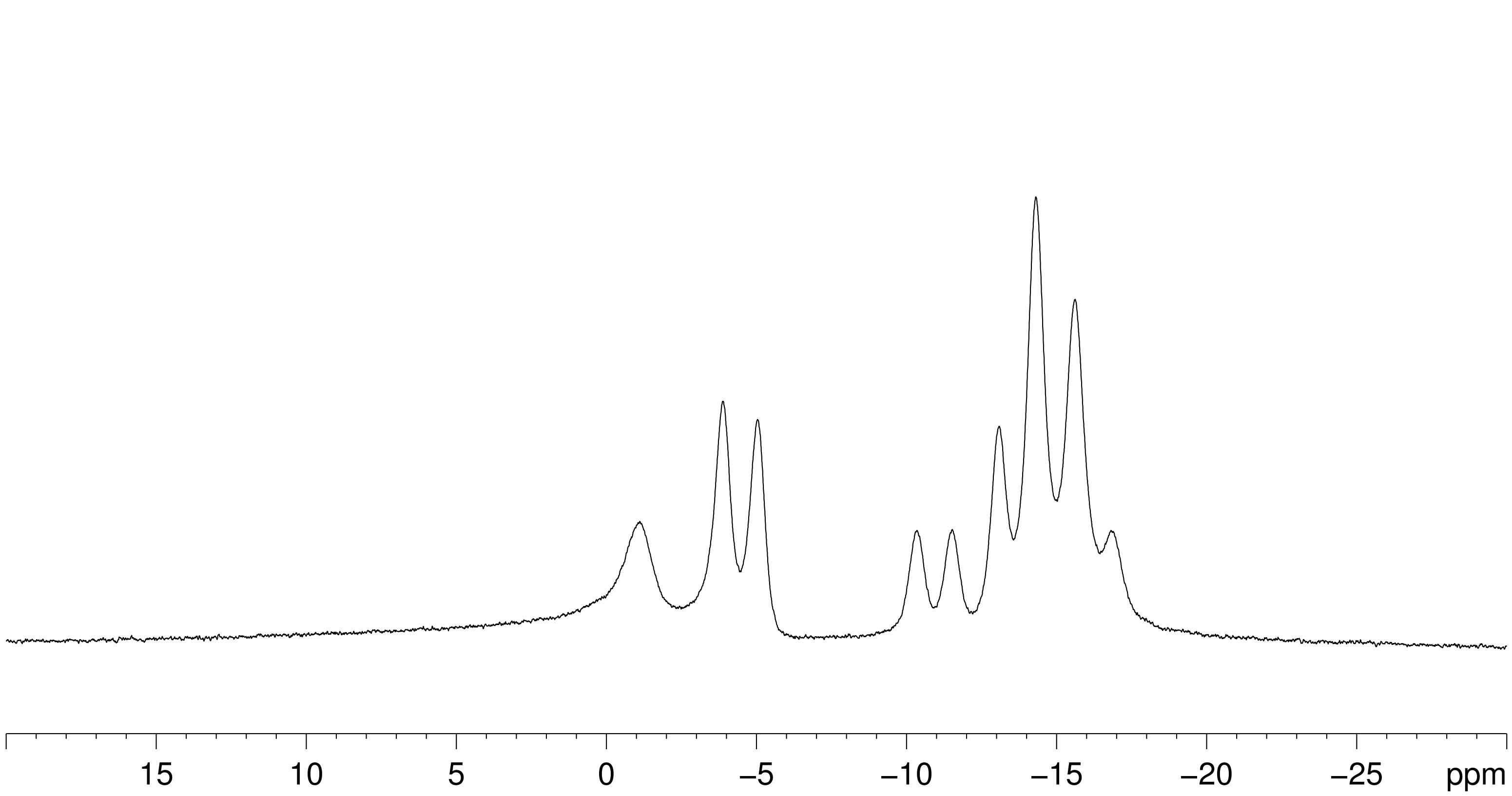

ZD-720-CDCl3-B(c)

```
NAME       ZD-720-CDCl3-B(c)
EXPNO                    1
PROCNO                   1
Date_             20140807
Time                 17.12
INSTRUM              spect
PROBHD     5 mm PABBO BB-
PULPROG               zg30
TD                   65536
SOLVENT              CDCl3
NS                       4
DS                       0
SWH             25510.203 Hz
FIDRES           0.389255 Hz
AQ              1.2845556 sec
RG                     161
DW                  19.600 usec
DE                    6.50 usec
TE                   298.0 K
D1              5.00000000 sec
TD0                      1

======== CHANNEL f1 ========
NUC1                   11B
P1                    7.60 usec
PL1                  -3.00 dB
PL1W           55.13059616 W
SFO1           128.3968556 MHz
SI                   32768
SF             128.3968865 MHz
WDW                     EM
SSB                      0
LB                    3.00 Hz
GB                       0
PC                    1.40
```

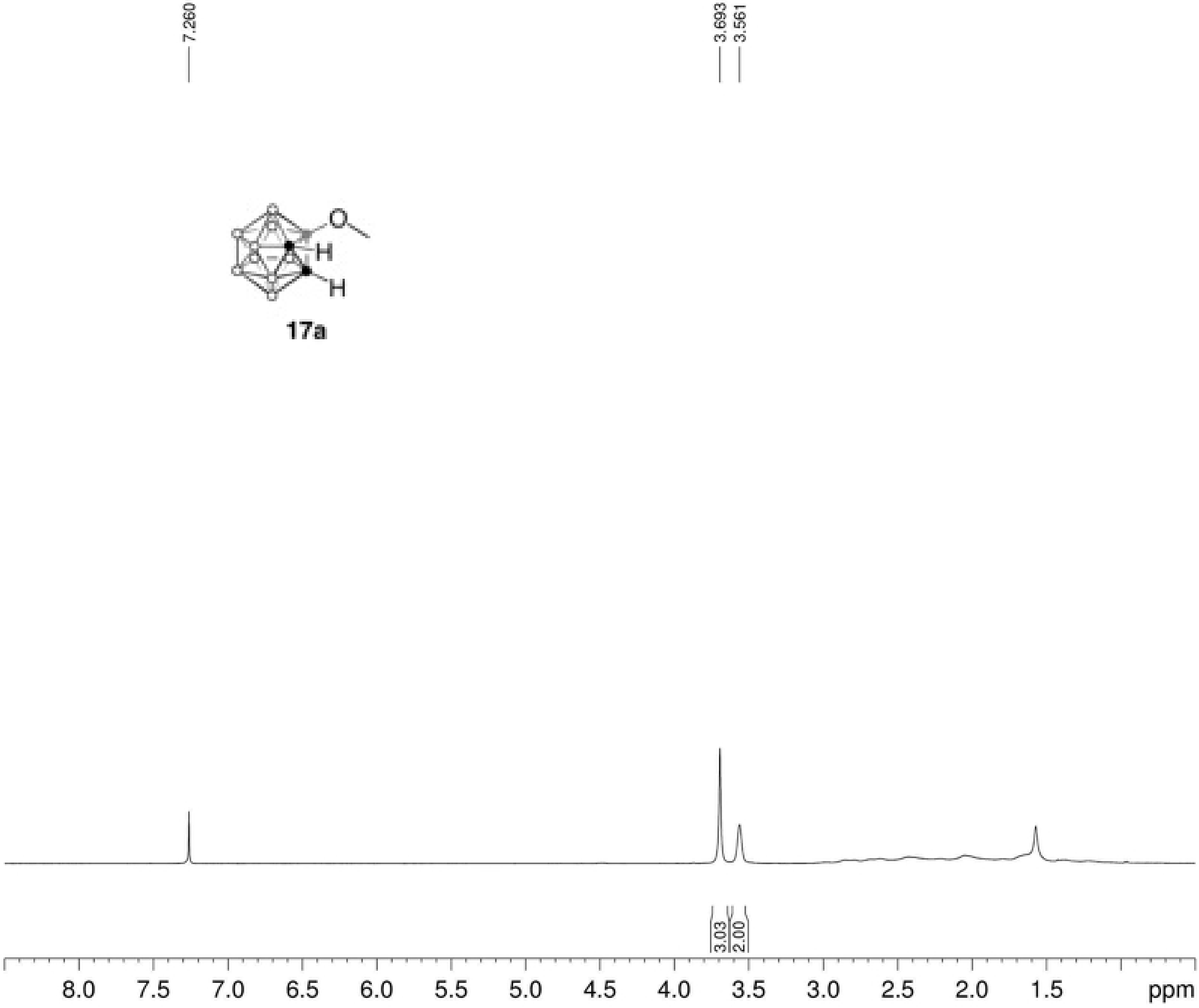

17a

ZD-732-CDCl3-H

NAME	ZD-732-CDCl3-H
EXPNO	1
PROCNO	1
Date_	20140812
Time	20.26
INSTRUM	spect
PROBHD	5 mm PABBO BB-
PULPROG	zg30
TD	65536
SOLVENT	CDCl3
NS	8
DS	2
SWH	8223.685 Hz
FIDRES	0.125483 Hz
AQ	3.9846387 sec
RG	144
DW	60.800 usec
DE	6.50 usec
TE	297.0 K
D1	1.00000000 sec
TD0	1

======== CHANNEL f1 =====

NUC1	1H
P1	14.00 usec
PL1	-1.00 dB
PL1W	13.56617069 W
SFO1	400.1924713 MHz
SI	32768
SF	400.1900153 MHz
WDW	EM
SSB	0
LB	0.30 Hz
GB	0
PC	1.00

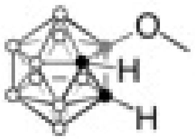

17a

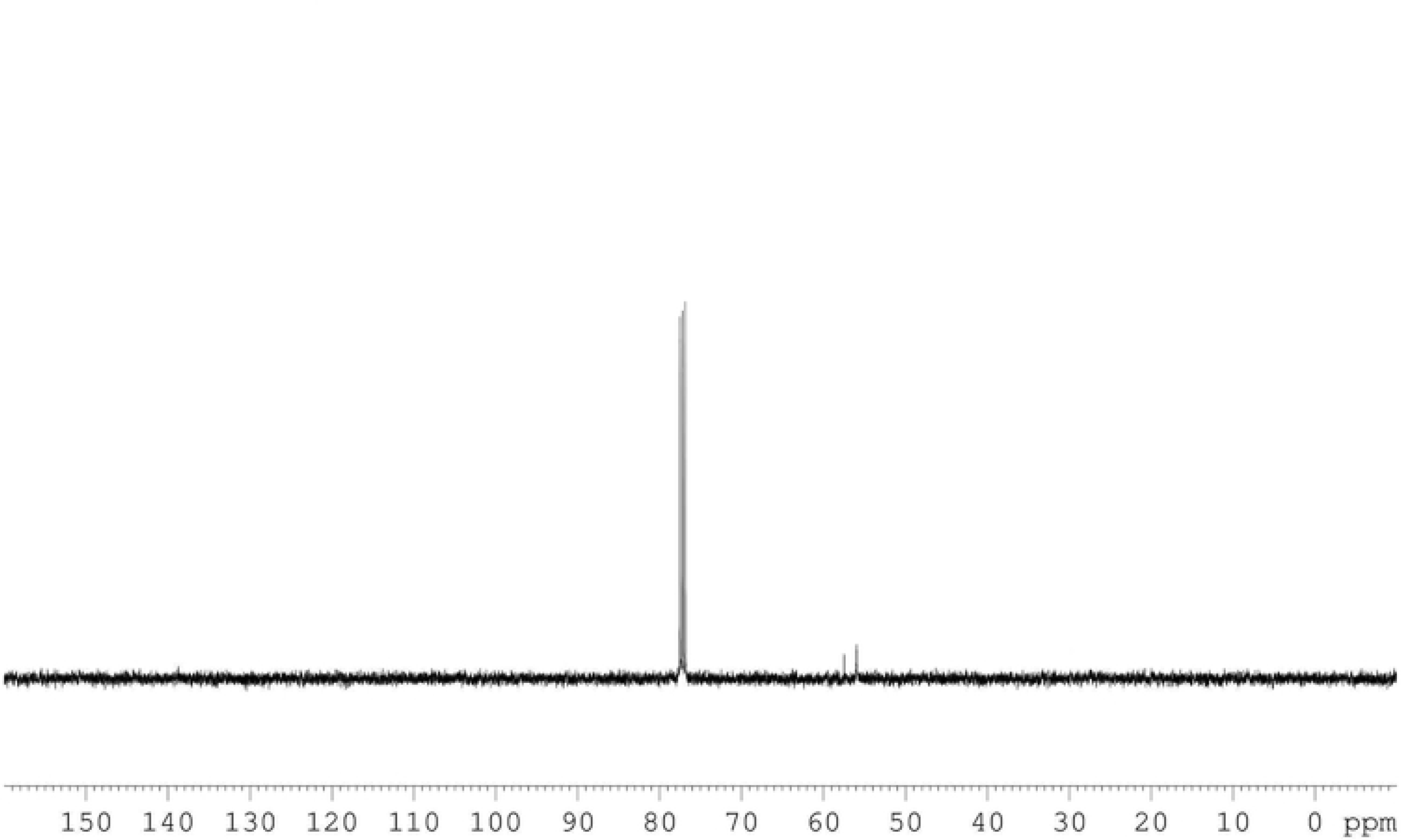

ZD-732-CDCl3-C

Bruker Advance III 400

NAME	ZD-732-CDCl3-C	
EXPNO	1	
PROCNO	1	
Date_	20140812	
Time	20.37	
INSTRUM	spect	
PROBHD	5 mm PADUL 13C	
PULPROG	zgdc	
TD	131072	
SOLVENT	CDCl3	
NS	300	
DS	0	
SWH	29761.904	Hz
FIDRES	0.227065	Hz
AQ	2.2020595	sec
RG	203	
DW	16.800	usec
DE	6.50	usec
TE	294.0	K
D1	1.00000000	sec
D11	0.03000000	sec
TD0	1	
======== CHANNEL f1 ========		
NUC1	13C	
P1	9.68	usec
PL1	-0.60	dB
PL1W	41.24164963	W
SFO1	100.6227690	MHz
======== CHANNEL f2 ========		
CPDPRG2	waltz16	
NUC2	1H	
PCPD2	90.00	usec
PL2	0.00	dB
PL12	15.66	dB
PL2W	8.31434441	W
PL12W	0.22585411	W
SFO2	400.1320007	MHz
SI	131072	
SF	100.6127553	MHz
WDW	EM	
SSB	0	
LB	1.00	Hz
GB	0	
PC	1.40	

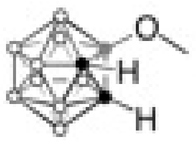

17a

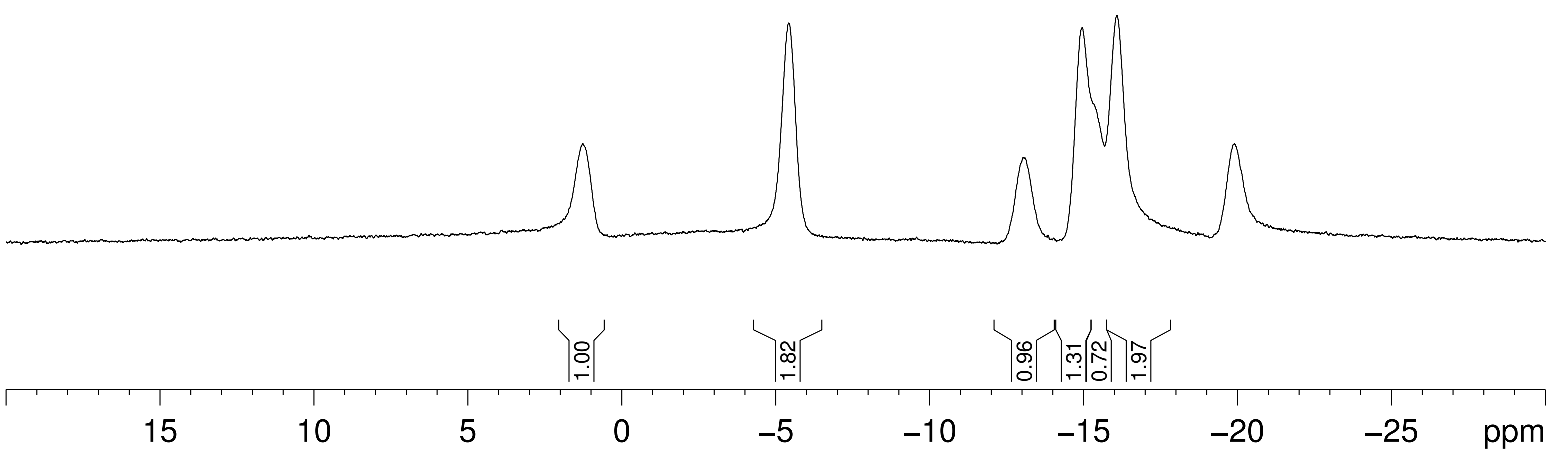

ZD-732-CDCl3-B(de)

NAME	ZD-732-CDCl3-B(de)	
EXPNO	1	
PROCNO	1	
Date_	20140812	
Time	20.28	
INSTRUM	spect	
PROBHD	5 mm PABBO BB-	
PULPROG	zgdc	
TD	65536	
SOLVENT	CDCl3	
NS	4	
DS	0	
SWH	25510.203	Hz
FIDRES	0.389255	Hz
AQ	1.2845556	sec
RG	322	
DW	19.600	usec
DE	6.50	usec
TE	297.3	K
D1	5.00000000	sec
D11	0.03000000	sec
TD0	1	
======== CHANNEL f1 ========		
NUC1	11B	
P1	7.60	usec
PL1	-3.00	dB
PL1W	55.13059616	W
SFO1	128.3968556	MHz
======== CHANNEL f2 ========		
CPDPRG2	waltz16	
NUC2	1H	
PCPD2	90.00	usec
PL2	-1.00	dB
PL12	15.16	dB
PL2W	13.56617069	W
PL12W	0.32844096	W
SFO2	400.1916008	MHz
SI	32768	
SF	128.3968847	MHz
WDW	EM	
SSB	0	
LB	3.00	Hz
GB	0	
PC	1.40	

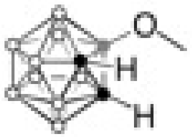

17a

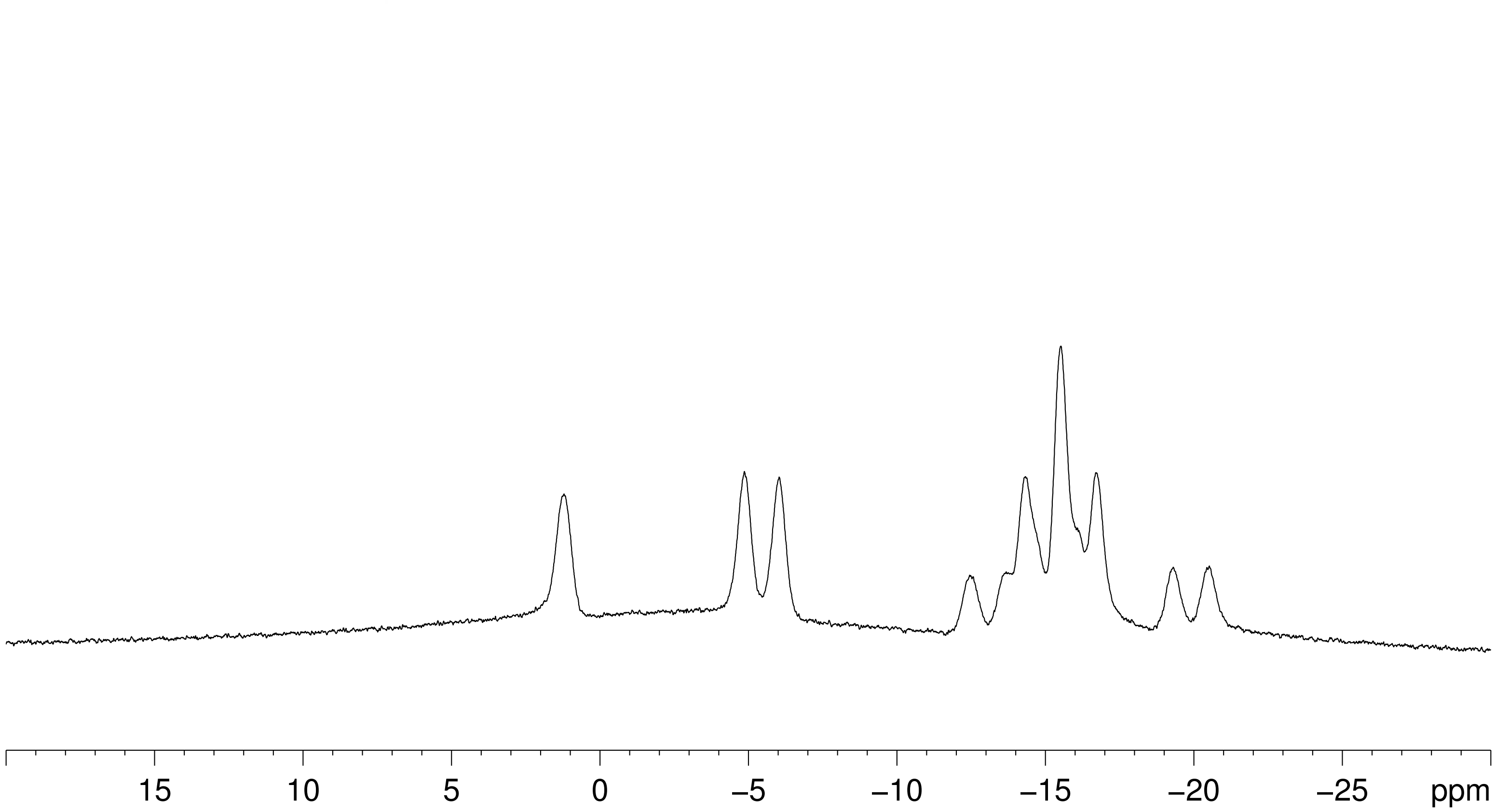

ZD-732-CDCl3-B(c)

NAME	ZD-732-CDCl3-B(c)	
EXPNO	1	
PROCNO	1	
Date_	20140812	
Time	20.29	
INSTRUM	spect	
PROBHD	5 mm PABBO BB-	
PULPROG	zg30	
TD	65536	
SOLVENT	CDCl3	
NS	12	
DS	0	
SWH	25510.203	Hz
FIDRES	0.389255	Hz
AQ	1.2845556	sec
RG	161	
DW	19.600	usec
DE	6.50	usec
TE	297.0	K
D1	5.00000000	sec
TD0	1	
======== CHANNEL f1 ========		
NUC1	11B	
P1	7.60	usec
PL1	-3.00	dB
PL1W	55.13059616	W
SFO1	128.3968556	MHz
SI	32768	
SF	128.3968865	MHz
WDW	EM	
SSB	0	
LB	3.00	Hz
GB	0	
PC	1.40	

ZD-733-CDCl3-H

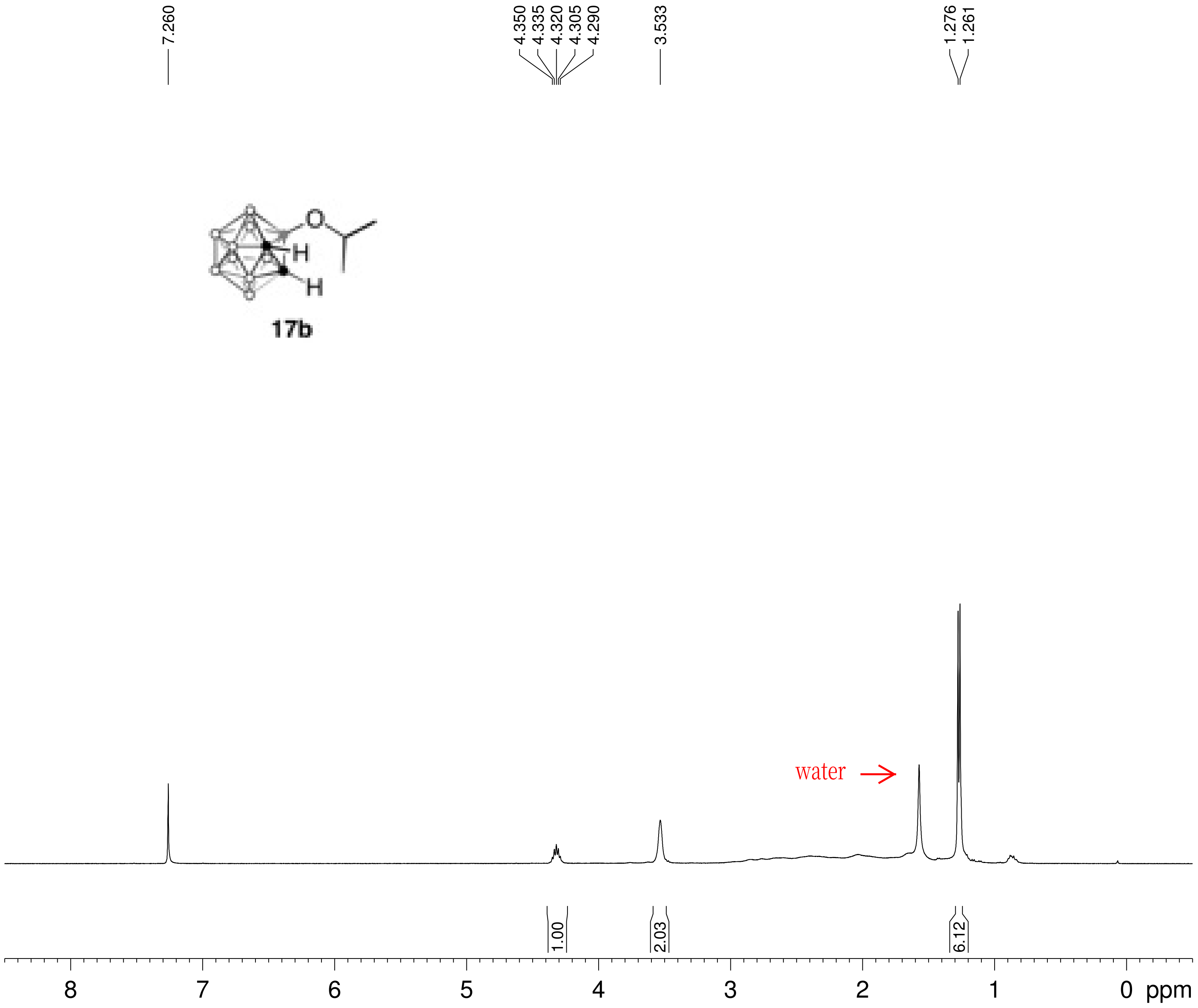

17b

NAME ZD–733–CDCl3–H
EXPNO 1
PROCNO 1
Date_ 20140814
Time 16.02
INSTRUM spect
PROBHD 5 mm PABBO BB–
PULPROG zg30
TD 65536
SOLVENT CDCl3
NS 16
DS 2
SWH 8223.685 Hz
FIDRES 0.125483 Hz
AQ 3.9846387 sec
RG 144
DW 60.800 usec
DE 6.50 usec
TE 297.2 K
D1 1.00000000 sec
TD0 1

======== CHANNEL f1 =====
NUC1 1H
P1 14.00 usec
PL1 –1.00 dB
PL1W 13.56617069 W
SFO1 400.1924713 MHz
SI 32768
SF 400.1900154 MHz
WDW EM
SSB 0
LB 0.30 Hz
GB 0
PC 1.00

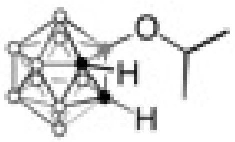

17b

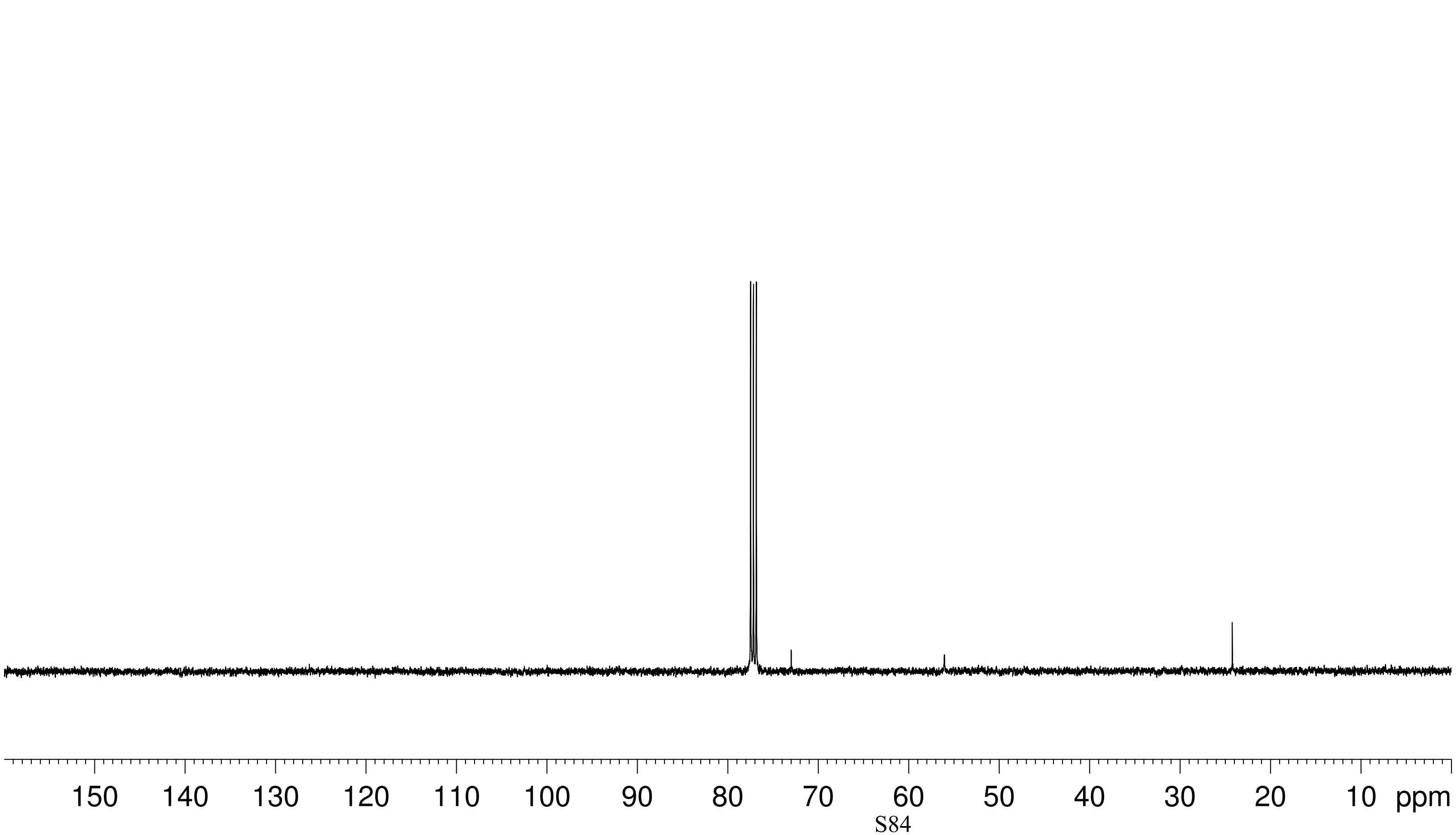

ZD-733-CDCl3-C

NAME	ZD-733-CDCl3-C	
EXPNO	1	
PROCNO	1	
Date_	20140814	
Time	16.07	
INSTRUM	spect	
PROBHD	5 mm PABBO BB-	
PULPROG	zgpg30	
TD	65536	
SOLVENT	CDCl3	
NS	300	
DS	4	
SWH	24038.461	Hz
FIDRES	0.366798	Hz
AQ	1.3631988	sec
RG	181	
DW	20.800	usec
DE	6.50	usec
TE	297.8	K
D1	2.00000000	sec
D11	0.03000000	sec
TD0	1	
======== CHANNEL f1 ========		
NUC1	13C	
P1	9.90	usec
PL1	-2.00	dB
PL1W	55.33689499	W
SFO1	100.6379183	MHz
======== CHANNEL f2 ========		
CPDPRG2	waltz16	
NUC2	1H	
PCPD2	90.00	usec
PL2	-1.00	dB
PL12	15.16	dB
PL13	18.62	dB
PL2W	13.56617069	W
PL12W	0.32844096	W
PL13W	0.14806664	W
SFO2	400.1916008	MHz
SI	32768	
SF	100.6278429	MHz
WDW	EM	
SSB	0	
LB	1.00	Hz
GB	0	
PC	1.40	

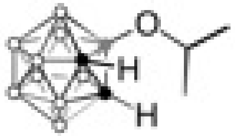

17b

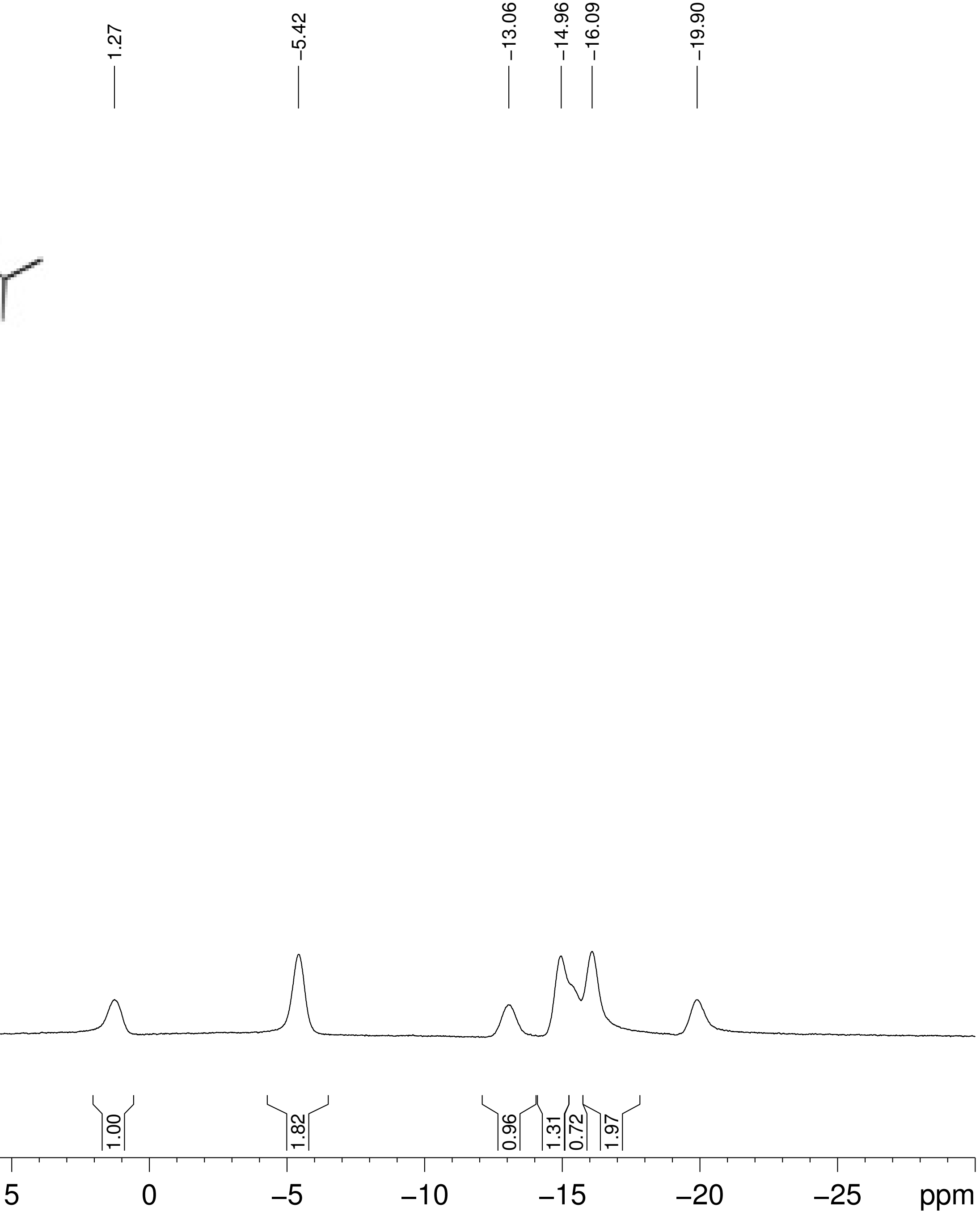

ZD-733-CDCl3-B(de)

NAME	ZD-733-CDCl3-B(de)
EXPNO	1
PROCNO	1
Date_	20140814
Time	16.28
INSTRUM	spect
PROBHD	5 mm PABBO BB-
PULPROG	zgdc
TD	65536
SOLVENT	CDCl3
NS	4
DS	0
SWH	25510.203 Hz
FIDRES	0.389255 Hz
AQ	1.2845556 sec
RG	322
DW	19.600 usec
DE	6.50 usec
TE	297.3 K
D1	5.00000000 sec
D11	0.03000000 sec
TD0	1
======== CHANNEL f1 ========	
NUC1	11B
P1	7.60 usec
PL1	-3.00 dB
PL1W	55.13059616 W
SFO1	128.3968556 MHz
======== CHANNEL f2 ========	
CPDPRG2	waltz16
NUC2	1H
PCPD2	90.00 usec
PL2	-1.00 dB
PL12	15.16 dB
PL2W	13.56617069 W
PL12W	0.32844096 W
SFO2	400.1916008 MHz
SI	32768
SF	128.3968847 MHz
WDW	EM
SSB	0
LB	3.00 Hz
GB	0
PC	1.40

ZD-733-CDCl3-B(c)

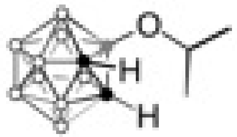

17b

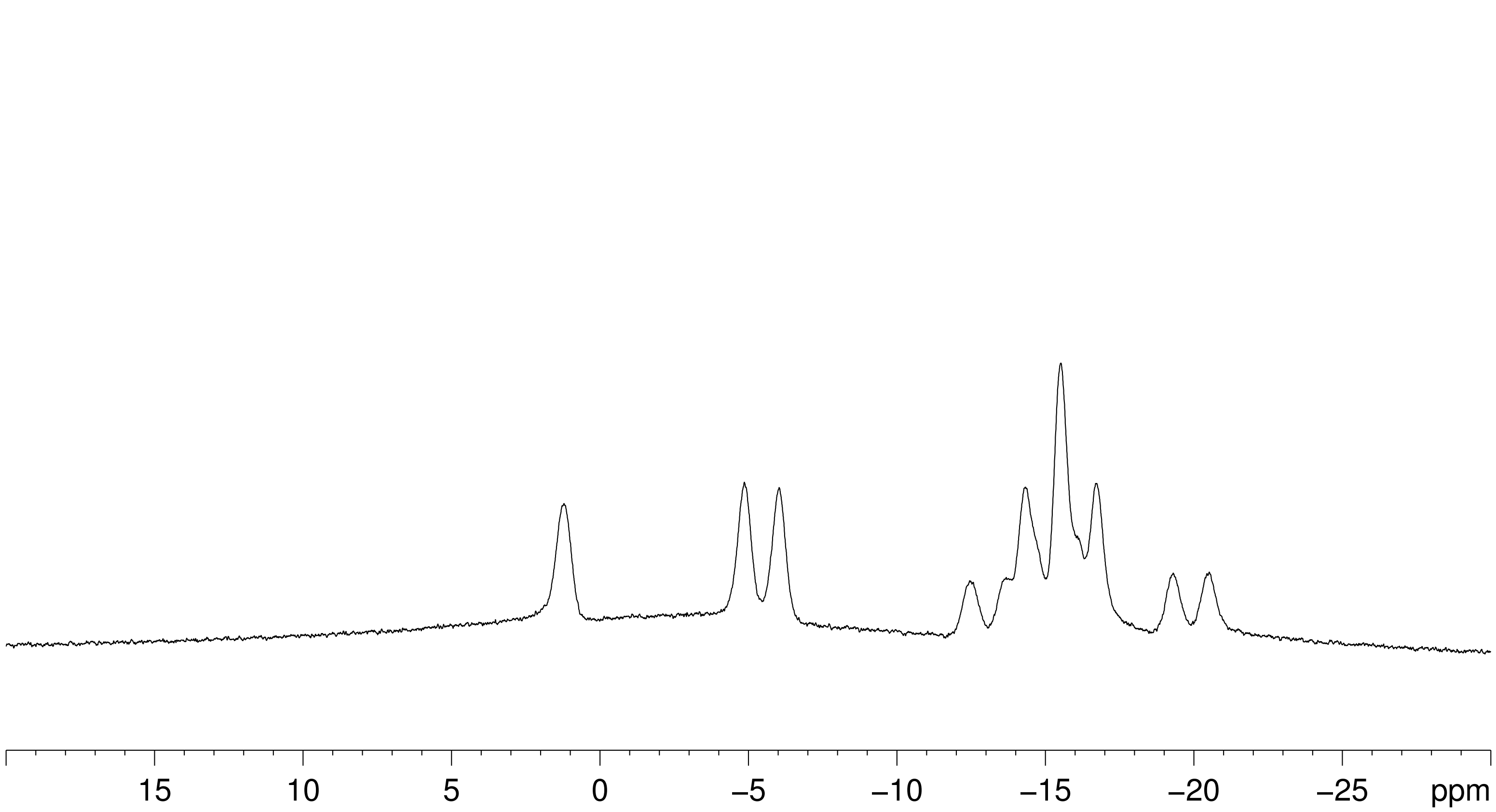

NAME	ZD-733-CDCl3-B(c)	
EXPNO	1	
PROCNO	1	
Date_	20140814	
Time	16.29	
INSTRUM	spect	
PROBHD	5 mm PABBO BB-	
PULPROG	zg30	
TD	65536	
SOLVENT	CDCl3	
NS	12	
DS	0	
SWH	25510.203	Hz
FIDRES	0.389255	Hz
AQ	1.2845556	sec
RG	161	
DW	19.600	usec
DE	6.50	usec
TE	297.0	K
D1	5.00000000	sec
TD0	1	

======== CHANNEL f1 ========

NUC1	11B	
P1	7.60	usec
PL1	-3.00	dB
PL1W	55.13059616	W
SFO1	128.3968556	MHz
SI	32768	
SF	128.3968865	MHz
WDW	EM	
SSB	0	
LB	3.00	Hz
GB	0	
PC	1.40	

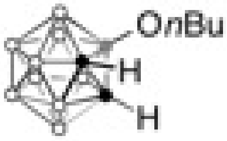

17c

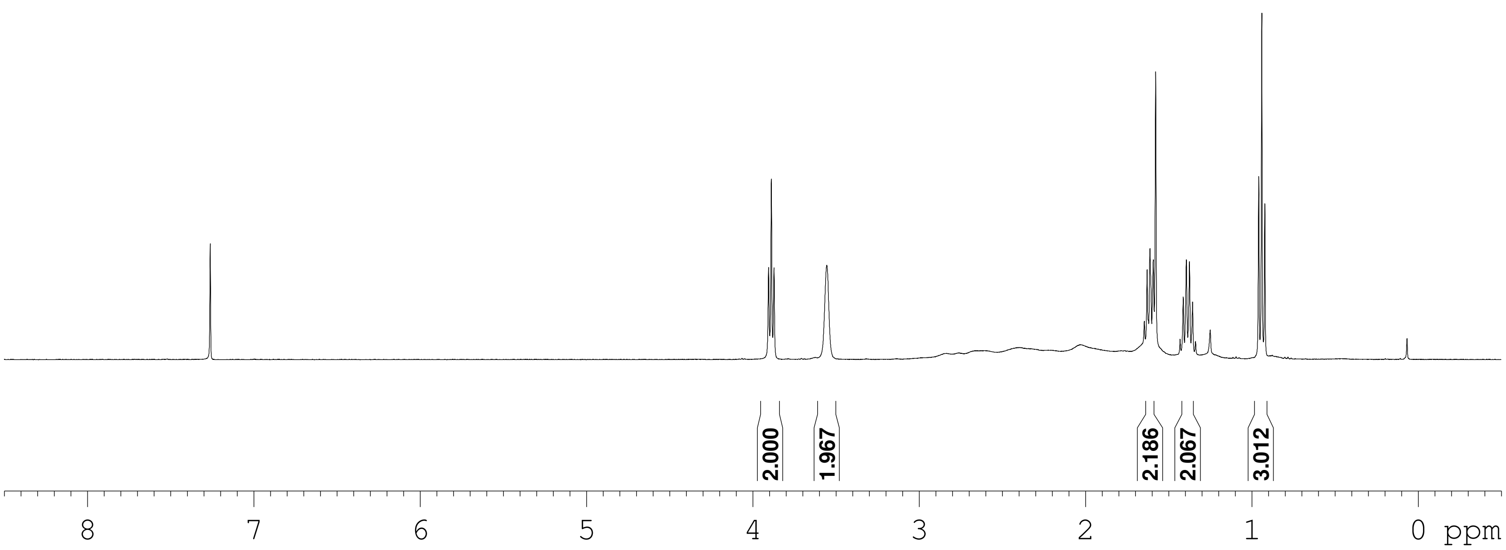

ZD-746-CDCl3-H

Bruker Advance III 400

NAME	ZD-746-CDCl3-H
EXPNO	1
PROCNO	1
Date_	20140823
Time	13.22
INSTRUM	spect
PROBHD	5 mm PADUL 13C
PULPROG	zg30
TD	65536
SOLVENT	CDCl3
NS	9
DS	2
SWH	8223.685 Hz
FIDRES	0.125483 Hz
AQ	3.9846387 sec
RG	203
DW	60.800 usec
DE	6.50 usec
TE	294.1 K
D1	2.00000000 sec
TD0	1

======== CHANNEL f1 ========

NUC1	1H
P1	14.83 usec
PL1	0.00 dB
PL1W	8.31434441 W
SFO1	400.1324710 MHz
SI	32768
SF	400.1300085 MHz
WDW	EM
SSB	0
LB	0.30 Hz
GB	0
PC	1.00

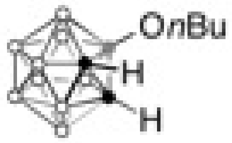

17c

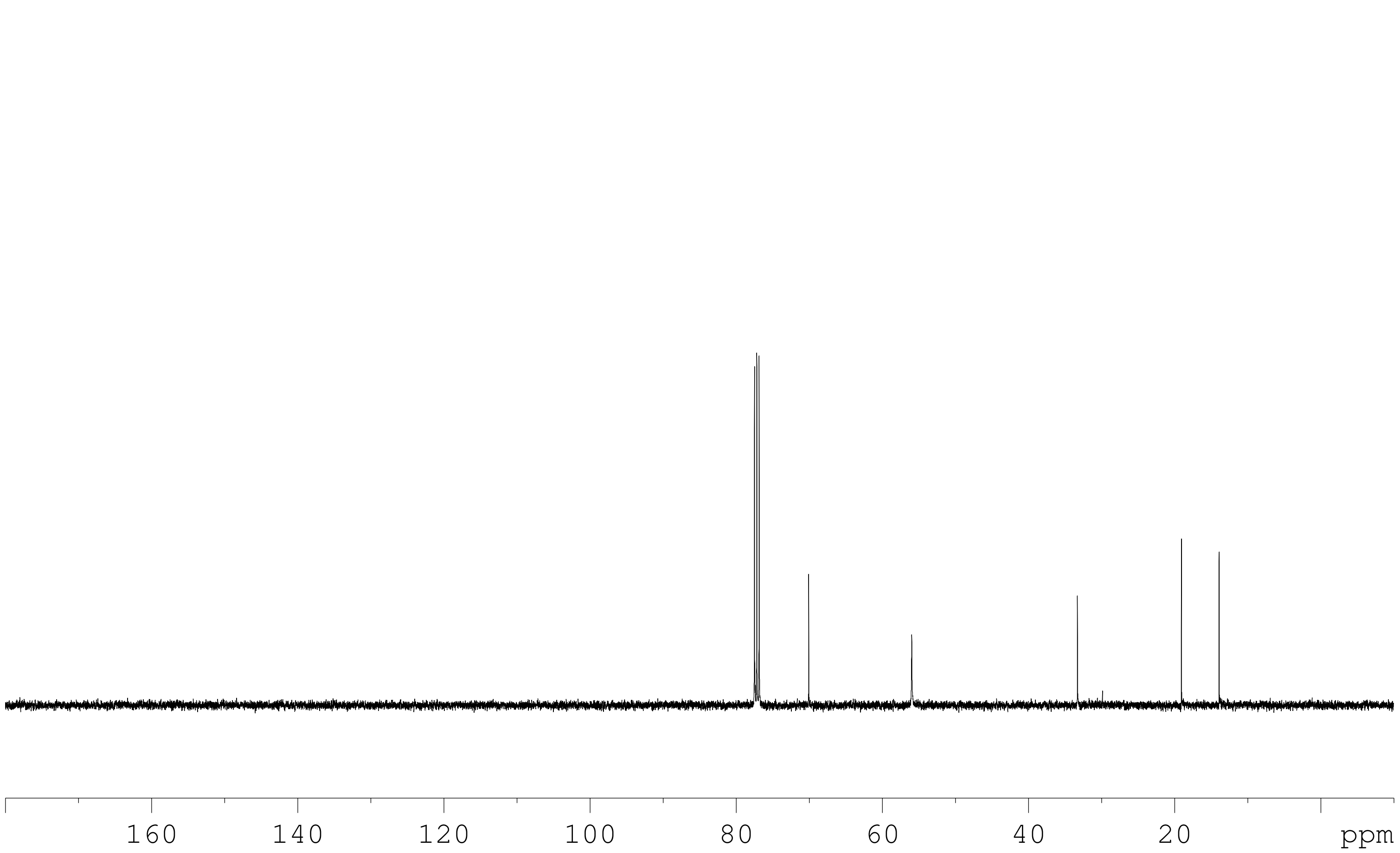

ZD-746-CDCl3-C

Bruker Advance III 400

NAME	ZD-746-CDCl3-C
EXPNO	1
PROCNO	1
Date_	20140823
Time	13.24
INSTRUM	spect
PROBHD	5 mm PADUL 13C
PULPROG	zgdc
TD	131072
SOLVENT	CDCl3
NS	912
DS	0
SWH	29761.904 Hz
FIDRES	0.227065 Hz
AQ	2.2020595 sec
RG	203
DW	16.800 usec
DE	6.50 usec
TE	294.3 K
D1	1.00000000 sec
D11	0.03000000 sec
TD0	1

======== CHANNEL f1 ========

NUC1	13C
P1	9.68 usec
PL1	-0.60 dB
PL1W	41.24164963 W
SFO1	100.6227690 MHz

======== CHANNEL f2 ========

CPDPRG2	waltz16
NUC2	1H
PCPD2	90.00 usec
PL2	0.00 dB
PL12	15.66 dB
PL2W	8.31434441 W
PL12W	0.22585411 W
SFO2	400.1320007 MHz
SI	131072
SF	100.6127552 MHz
WDW	EM
SSB	0
LB	1.00 Hz
GB	0
PC	1.40

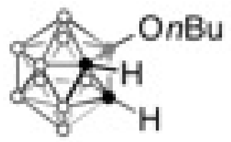

17c

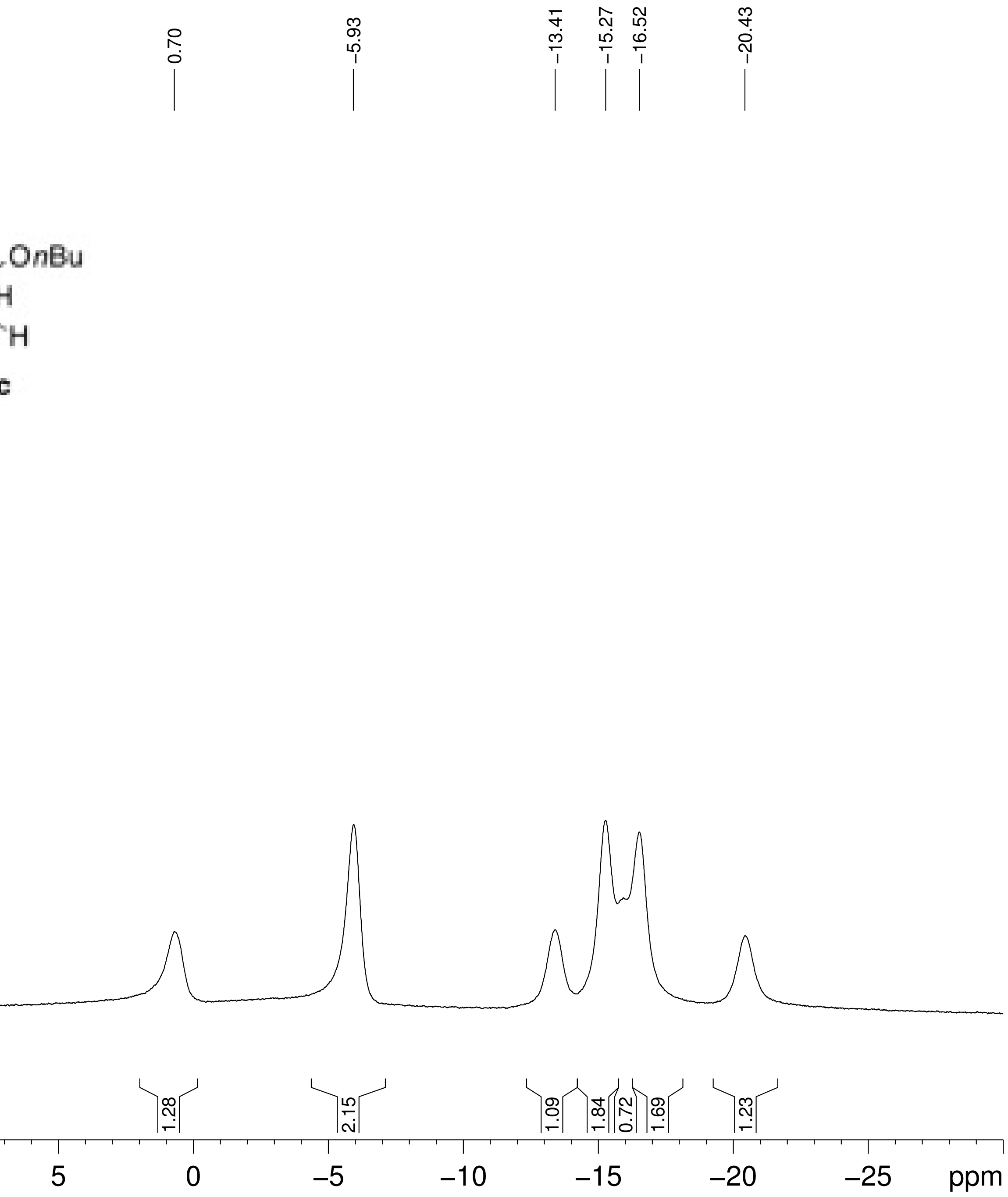

ZD-746-CDCl3-B(de)

NAME	ZD-746-CDCl3-B(de)	
EXPNO	1	
PROCNO	1	
Date_	20140823	
Time	13.08	
INSTRUM	spect	
PROBHD	5 mm PABBO BB-	
PULPROG	zgdc	
TD	65536	
SOLVENT	DMSO	
NS	12	
DS	0	
SWH	25510.203	Hz
FIDRES	0.389255	Hz
AQ	1.2845556	sec
RG	322	
DW	19.600	usec
DE	6.50	usec
TE	297.6	K
D1	5.00000000	sec
D11	0.03000000	sec
TD0	1	

======== CHANNEL f1 ========

NUC1	11B	
P1	7.60	usec
PL1	-3.00	dB
PL1W	55.13059616	W
SFO1	128.3968556	MHz

======== CHANNEL f2 ========

CPDPRG2	waltz16	
NUC2	1H	
PCPD2	90.00	usec
PL2	-1.00	dB
PL12	15.16	dB
PL2W	13.56617069	W
PL12W	0.32844096	W
SFO2	400.1916008	MHz
SI	32768	
SF	128.3968847	MHz
WDW	EM	
SSB	0	
LB	3.00	Hz
GB	0	
PC	1.40	

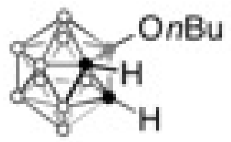

17c

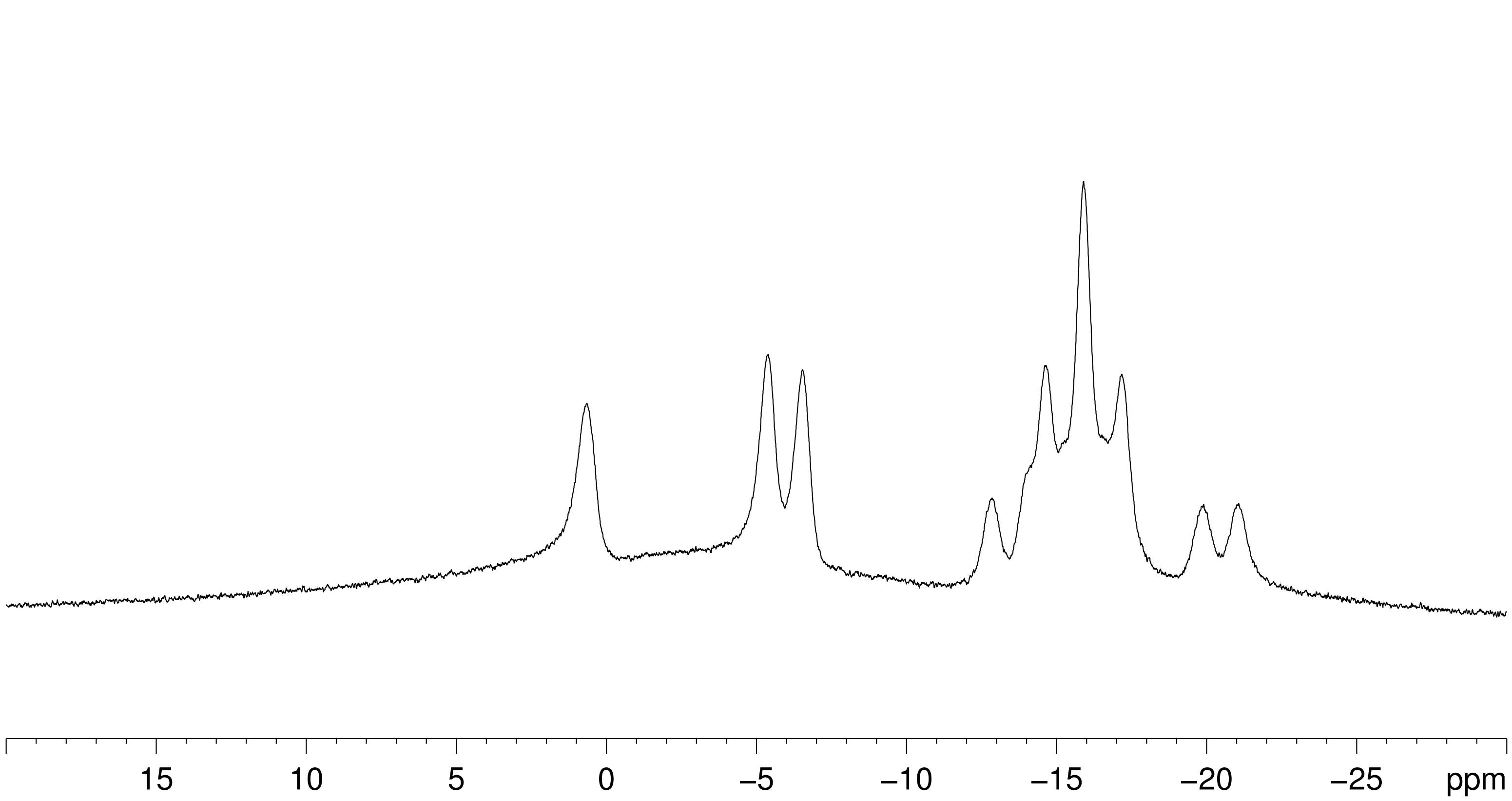

ZD-746-CDCl3-B(c)

NAME	ZD-746-CDCl3-B(c)
EXPNO	1
PROCNO	1
Date_	20140823
Time	13.09
INSTRUM	spect
PROBHD	5 mm PABBO BB-
PULPROG	zg30
TD	65536
SOLVENT	DMSO
NS	12
DS	0
SWH	25510.203 Hz
FIDRES	0.389255 Hz
AQ	1.2845556 sec
RG	161
DW	19.600 usec
DE	6.50 usec
TE	297.2 K
D1	5.00000000 sec
TD0	1

======== CHANNEL f1 ========

NUC1	11B
P1	7.60 usec
PL1	-3.00 dB
PL1W	55.13059616 W
SFO1	128.3968556 MHz
SI	32768
SF	128.3968865 MHz
WDW	EM
SSB	0
LB	3.00 Hz
GB	0
PC	1.40

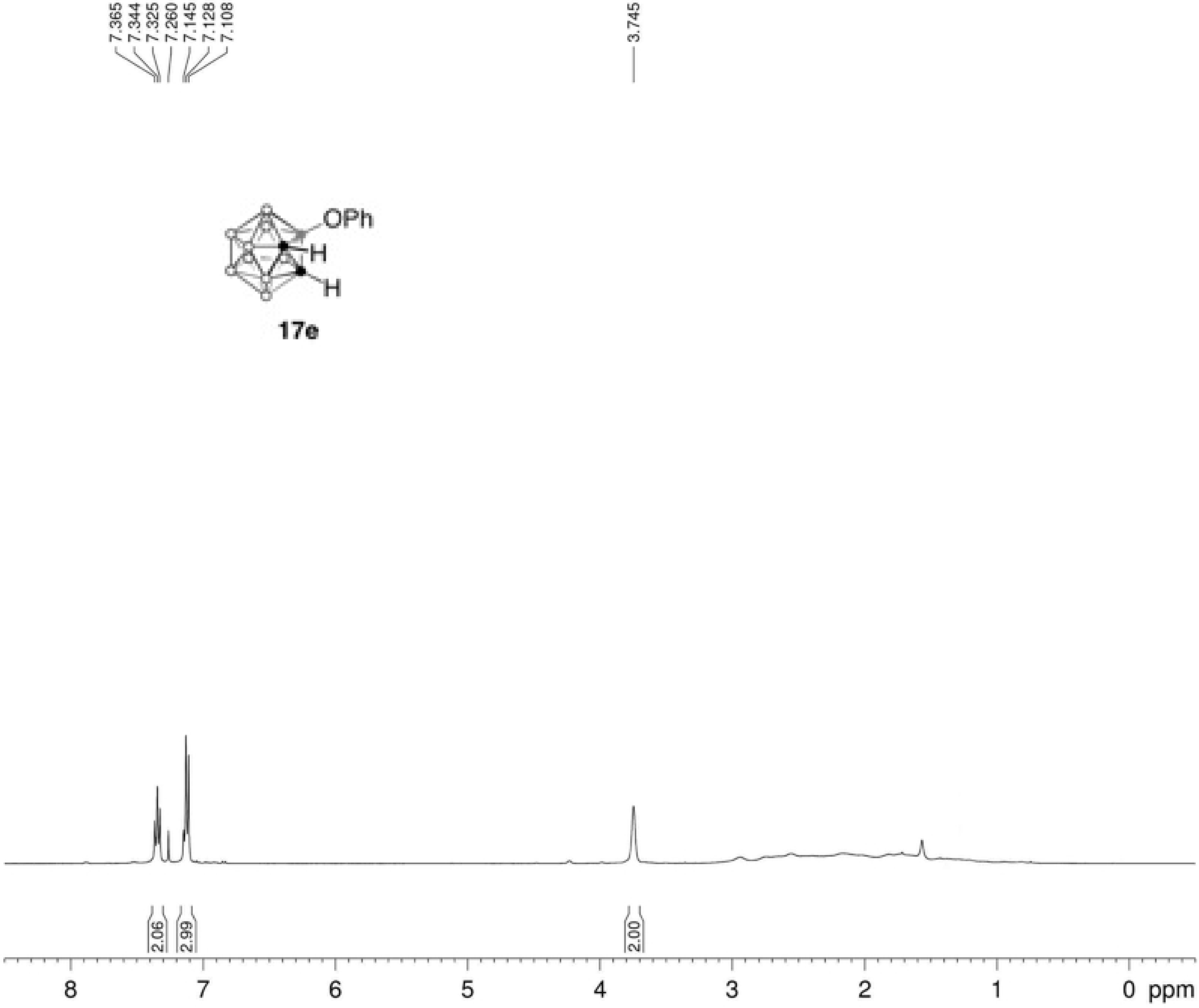

ZD-7142-CDCl3-H

NAME	ZD-7142-CDCl3-H
EXPNO	1
PROCNO	1
Date_	20140802
Time	20.30
INSTRUM	spect
PROBHD	5 mm PABBO BB-
PULPROG	zg30
TD	65536
SOLVENT	CDCl3
NS	8
DS	2
SWH	8223.685 Hz
FIDRES	0.125483 Hz
AQ	3.9846387 sec
RG	64
DW	60.800 usec
DE	6.50 usec
TE	297.9 K
D1	1.00000000 sec
TD0	1

======== CHANNEL f1 =====

NUC1	1H
P1	14.00 usec
PL1	-1.00 dB
PL1W	13.56617069 W
SFO1	400.1924713 MHz
SI	32768
SF	400.1900153 MHz
WDW	EM
SSB	0
LB	0.30 Hz
GB	0
PC	1.00

ZD-7142-CDCl3-C

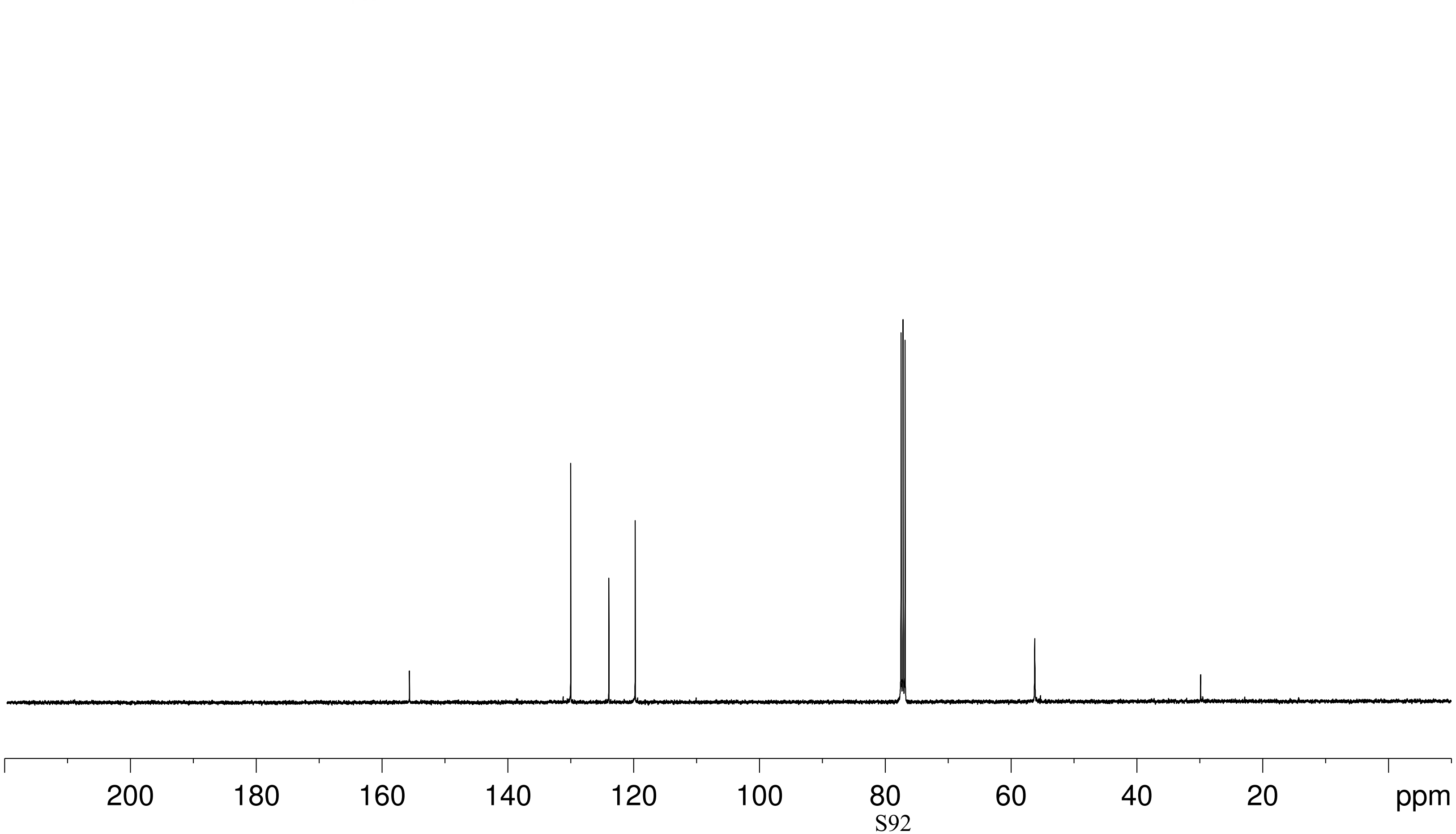

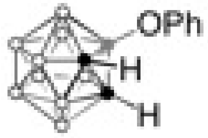

17e

```
NAME       ZD-7142-CDCl3-C
EXPNO                    1
PROCNO                   1
Date_             20140802
Time                 20.43
INSTRUM              spect
PROBHD     5 mm PABBO BB-
PULPROG            zgpg30
TD                   65536
SOLVENT              CDCl3
NS                    1028
DS                       4
SWH              24038.461 Hz
FIDRES            0.366798 Hz
AQ               1.3631988 sec
RG                     203
DW                  20.800 usec
DE                    6.50 usec
TE                   298.4 K
D1              2.00000000 sec
D11             0.03000000 sec
TD0                      1

======== CHANNEL f1 ========
NUC1                   13C
P1                    9.90 usec
PL1                  -2.00 dB
PL1W           55.33689499 W
SFO1          100.6379183 MHz

======== CHANNEL f2 ========
CPDPRG2            waltz16
NUC2                    1H
PCPD2                90.00 usec
PL2                  -1.00 dB
PL12                 15.16 dB
PL13                 18.62 dB
PL2W           13.56617069 W
PL12W           0.32844096 W
PL13W           0.14806664 W
SFO2          400.1916008 MHz
SI                   32768
SF            100.6278442 MHz
WDW                     EM
SSB                      0
LB                    1.00 Hz
GB                       0
PC                    1.40
```

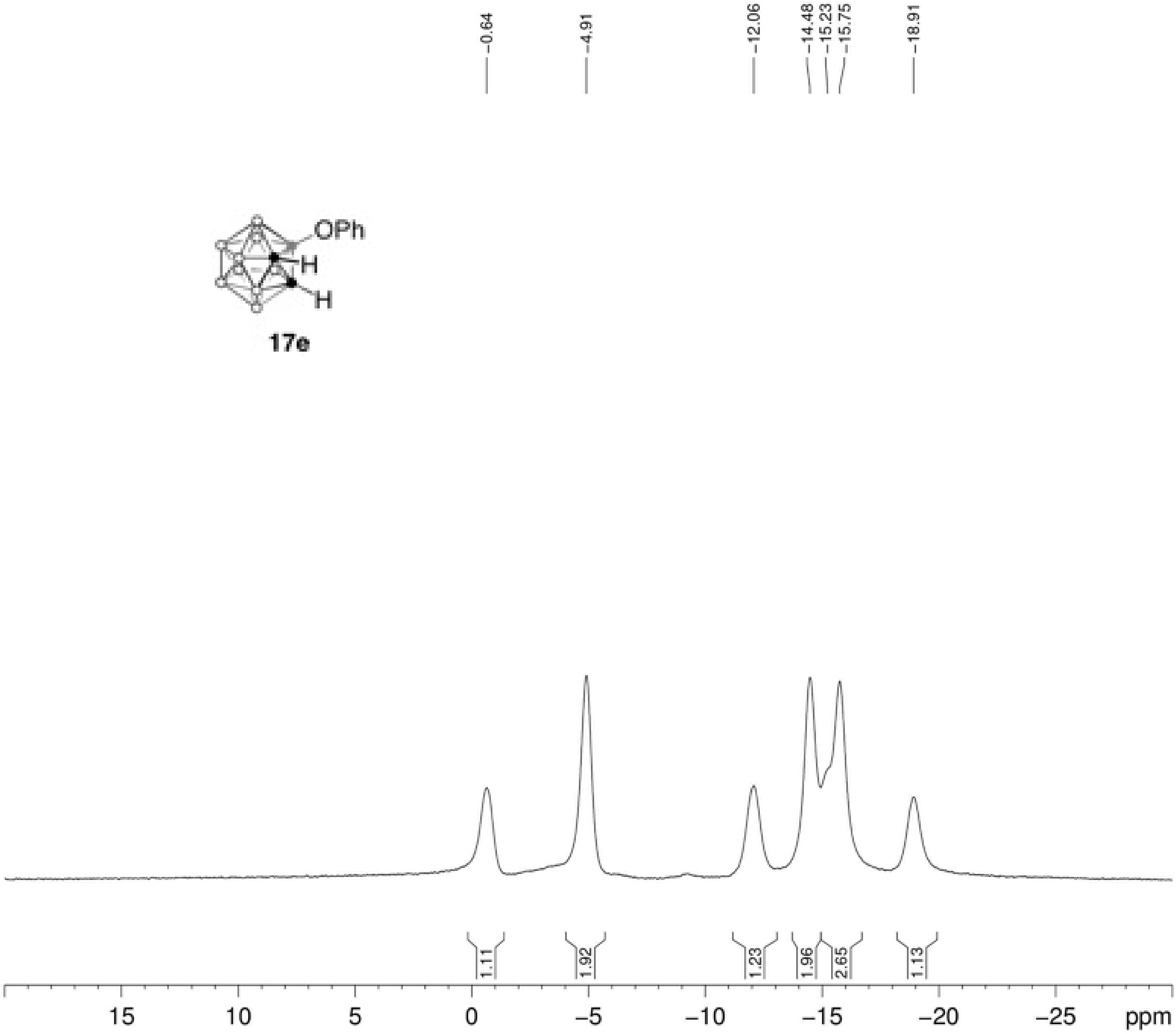

ZD-7142-CDCl3-B(de)

NAME	ZD-7142-CDCl3-B(de)	
EXPNO	1	
PROCNO	1	
Date_	20140802	
Time	21.45	
INSTRUM	spect	
PROBHD	5 mm PABBO BB-	
PULPROG	zgdc	
TD	65536	
SOLVENT	CDCl3	
NS	3	
DS	0	
SWH	25510.203	Hz
FIDRES	0.389255	Hz
AQ	1.2845556	sec
RG	287	
DW	19.600	usec
DE	6.50	usec
TE	298.5	K
D1	5.00000000	sec
D11	0.03000000	sec
TD0	1	

======== CHANNEL f1 ========

NUC1	11B	
P1	7.60	usec
PL1	-3.00	dB
PL1W	55.13059616	W
SFO1	128.3968556	MHz

======== CHANNEL f2 ========

CPDPRG2	waltz16	
NUC2	1H	
PCPD2	90.00	usec
PL2	-1.00	dB
PL12	15.16	dB
PL2W	13.56617069	W
PL12W	0.32844096	W
SFO2	400.1916008	MHz
SI	32768	
SF	128.3968847	MHz
WDW	EM	
SSB	0	
LB	3.00	Hz
GB	0	
PC	1.40	

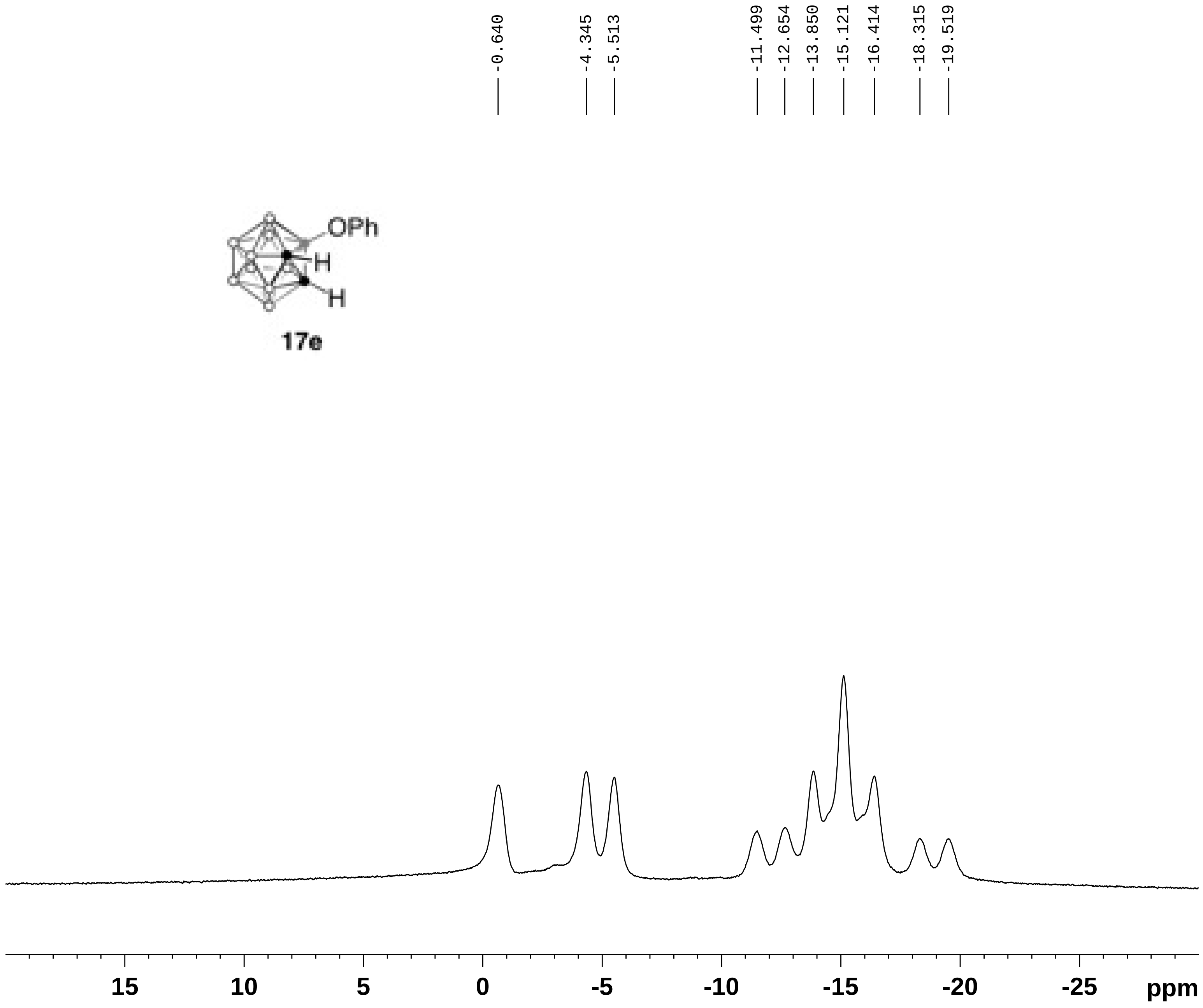

NAME	ZD-7142-CDCl3-B(c)
EXPNO	1
PROCNO	1
Date_	20140802
Time	21.46
INSTRUM	spect
PROBHD	5 mm PABBO BB-
PULPROG	zg30
TD	65536
SOLVENT	CDCl3
NS	9
DS	0
SWH	25510.203 Hz
FIDRES	0.389255 Hz
AQ	1.2845556 sec
RG	161
DW	19.600 usec
DE	6.50 usec
TE	298.2 K
D1	5.00000000 sec
TD0	1
======== CHANNEL f1 ========	
NUC1	11B
P1	7.60 usec
PL1	-3.00 dB
PL1W	55.13059616 W
SFO1	128.3968556 MHz
SI	32768
SF	128.3968865 MHz
WDW	EM
SSB	0
LB	3.00 Hz
GB	0
PC	1.40

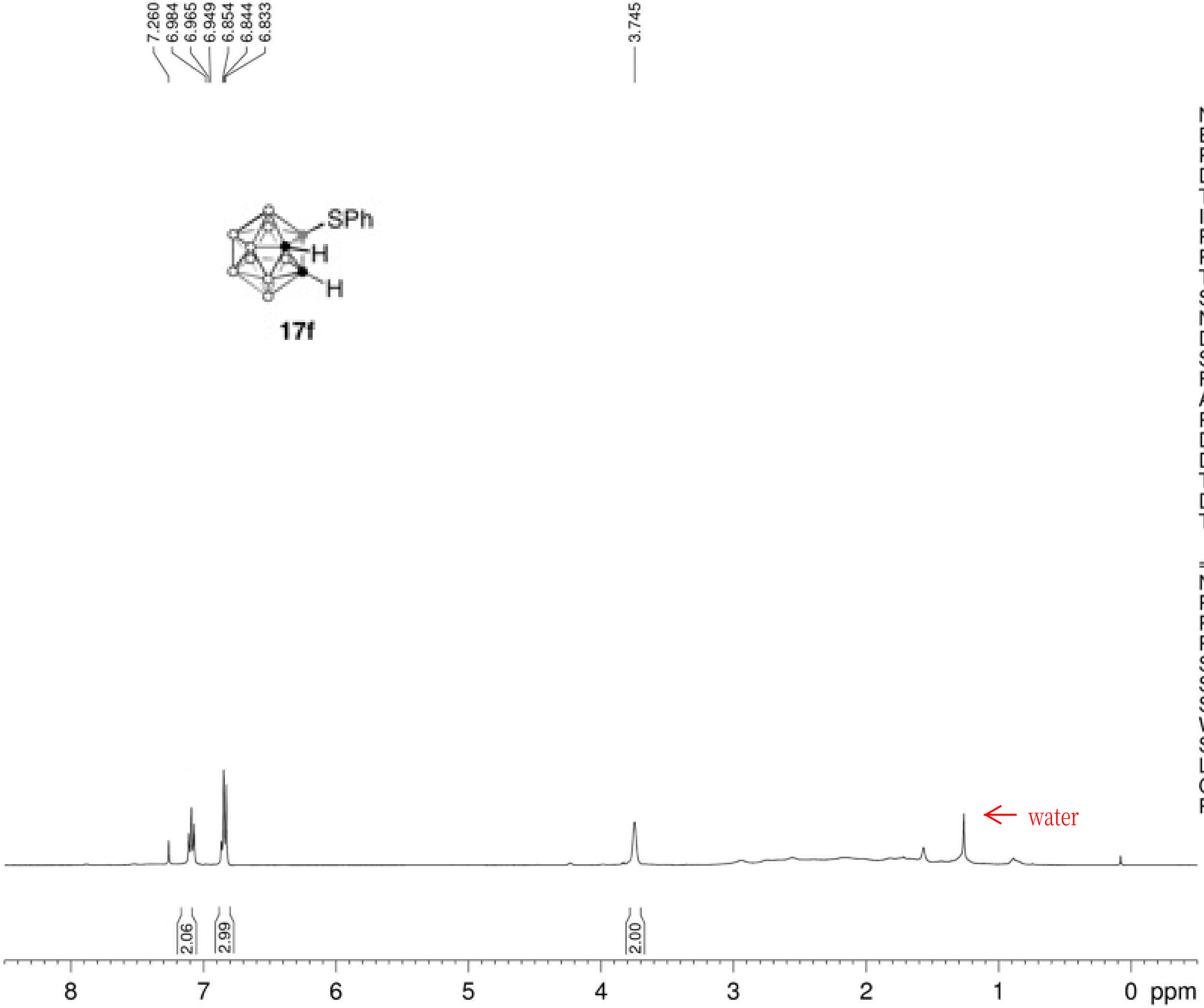

ZD-740-CDCl3-H

NAME ZD–740–CDCl3–H
EXPNO 1
PROCNO 1
Date_ 20140819
Time 20.30
INSTRUM spect
PROBHD 5 mm PABBO BB–
PULPROG zg30
TD 65536
SOLVENT CDCl3
NS 8
DS 2
SWH 8223.685 Hz
FIDRES 0.125483 Hz
AQ 3.9846387 sec
RG 64
DW 60.800 usec
DE 6.50 usec
TE 297.9 K
D1 1.00000000 sec
TD0 1

======== CHANNEL f1 ========
NUC1 1H
P1 14.00 usec
PL1 –1.00 dB
PL1W 13.56617069 W
SFO1 400.1924713 MHz
SI 32768
SF 400.1900153 MHz
WDW EM
SSB 0
LB 0.30 Hz
GB 0
PC 1.00

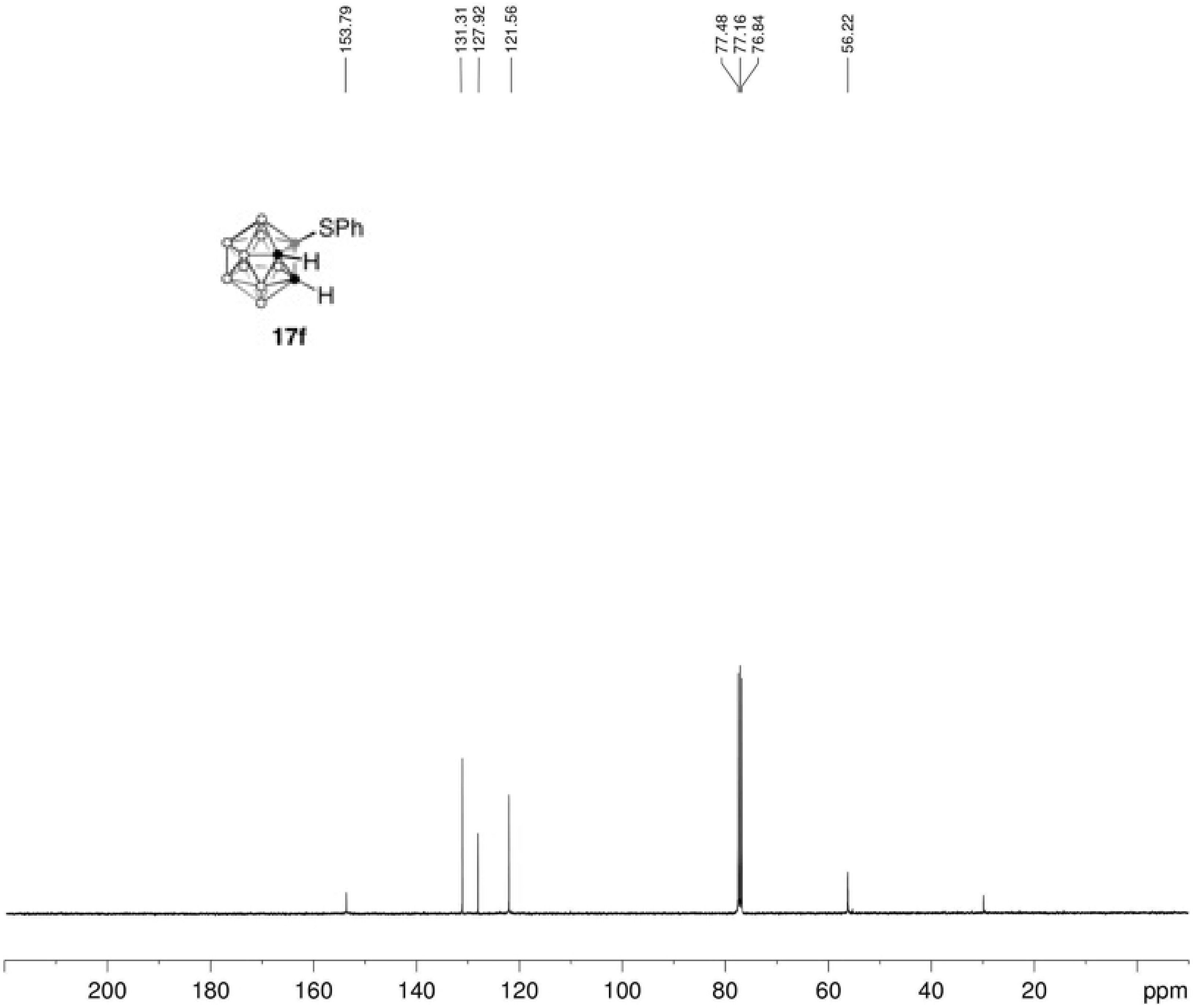

17f

ZD-740-CDCl3-C

NAME	ZD-740-CDCl3-C
EXPNO	1
PROCNO	1
Date_	20140819
Time	20.43
INSTRUM	spect
PROBHD	5 mm PABBO BB-
PULPROG	zgpg30
TD	65536
SOLVENT	CDCl3
NS	1079
DS	4
SWH	24038.461 Hz
FIDRES	0.366798 Hz
AQ	1.3631988 sec
RG	203
DW	20.800 usec
DE	6.50 usec
TE	298.4 K
D1	2.00000000 sec
D11	0.03000000 sec
TD0	1
======== CHANNEL f1 ========	
NUC1	13C
P1	9.90 usec
PL1	-2.00 dB
PL1W	55.33689499 W
SFO1	100.6379183 MHz
======== CHANNEL f2 ========	
CPDPRG2	waltz16
NUC2	1H
PCPD2	90.00 usec
PL2	-1.00 dB
PL12	15.16 dB
PL13	18.62 dB
PL2W	13.56617069 W
PL12W	0.32844096 W
PL13W	0.14806664 W
SFO2	400.1916008 MHz
SI	32768
SF	100.6278442 MHz
WDW	EM
SSB	0
LB	1.00 Hz
GB	0
PC	1.40

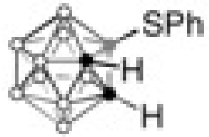

17f

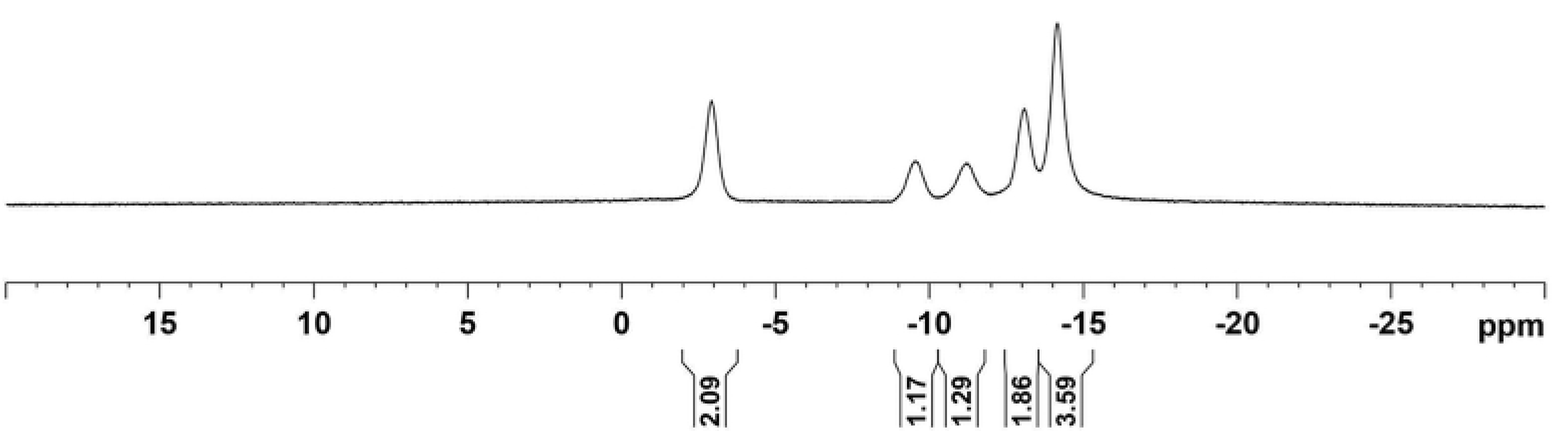

NAME	ZD-3-SPh-CDCl3-B(de)	
EXPNO	1	
PROCNO	1	
Date_	20151127	
Time	10.28	h
INSTRUM	spect	
PROBHD	Z108618_0257 (	
PULPROG	zgdc	
TD	65536	
SOLVENT	CDCl3	
NS	8	
DS	4	
SWH	24038.461	Hz
FIDRES	0.366798	Hz
AQ	1.3631988	sec
RG	322	
DW	20.800	usec
DE	6.50	usec
TE	298.4	K
D1	2.00000000	sec
D11	0.03000000	sec
TD0	1	
SFO1	128.4096890	MHz
NUC1	11B	
P1	7.50	usec
SI	32768	
SF	128.4096561	MHz
WDW	EM	
SSB	0	
LB	1.00	Hz
GB	0	
PC	1.40	

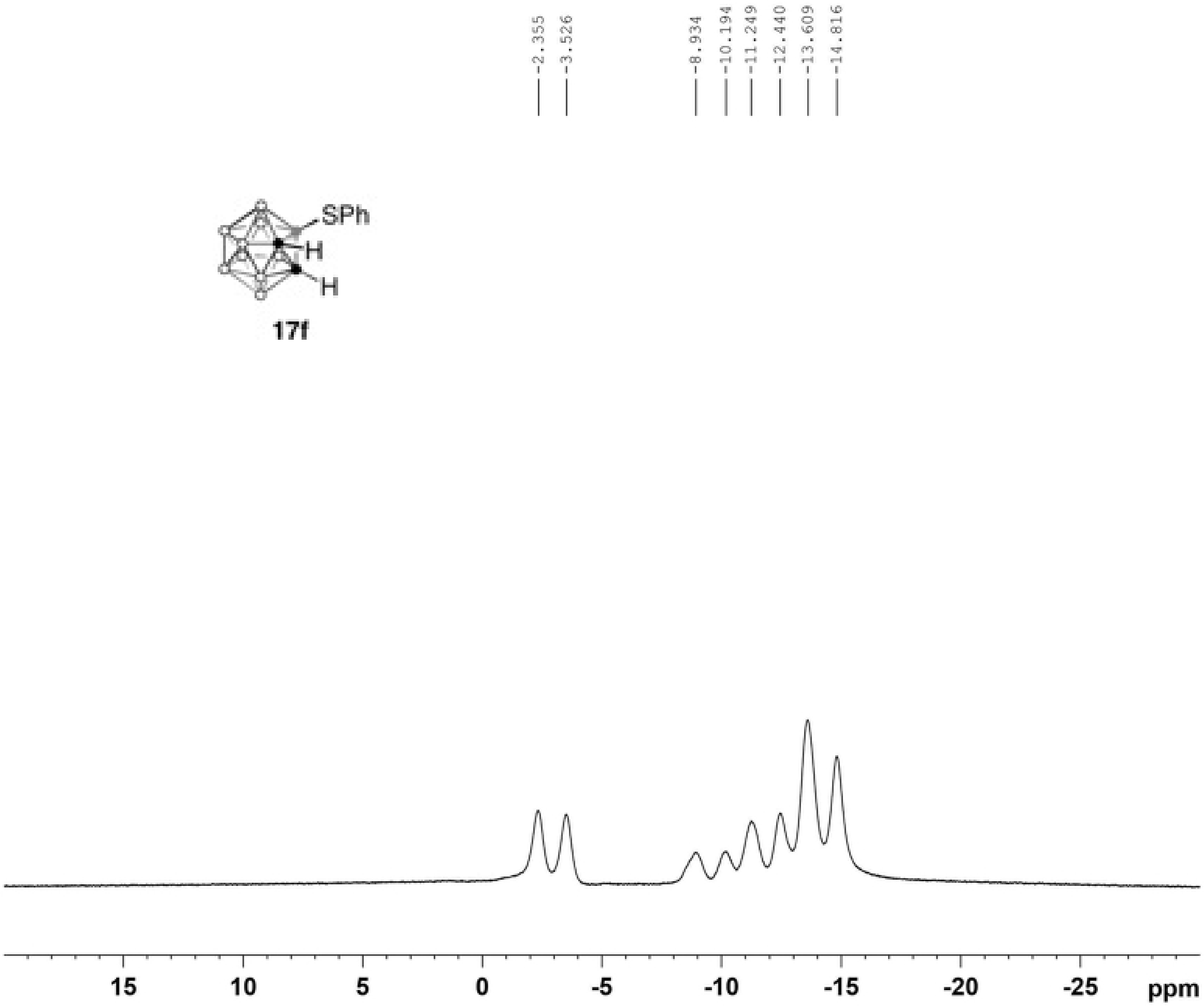

NAME	ZD-3-SPh-CDCl3-B(c)
EXPNO	1
PROCNO	1
Date_	20151127
Time	10.29 h
INSTRUM	spect
PROBHD	Z108618_0257 (
PULPROG	zg
TD	65536
SOLVENT	CDCl3
NS	24
DS	2
SWH	24038.461 Hz
FIDRES	0.366798 Hz
AQ	1.3631988 sec
RG	101
DW	20.800 usec
DE	6.50 usec
TE	298.3 K
D1	2.00000000 sec
TD0	1
SFO1	128.4096890 MHz
NUC1	11B
P1	7.50 usec
SI	32768
SF	128.4096580 MHz
WDW	EM
SSB	0
LB	1.00 Hz
GB	0
PC	1.40

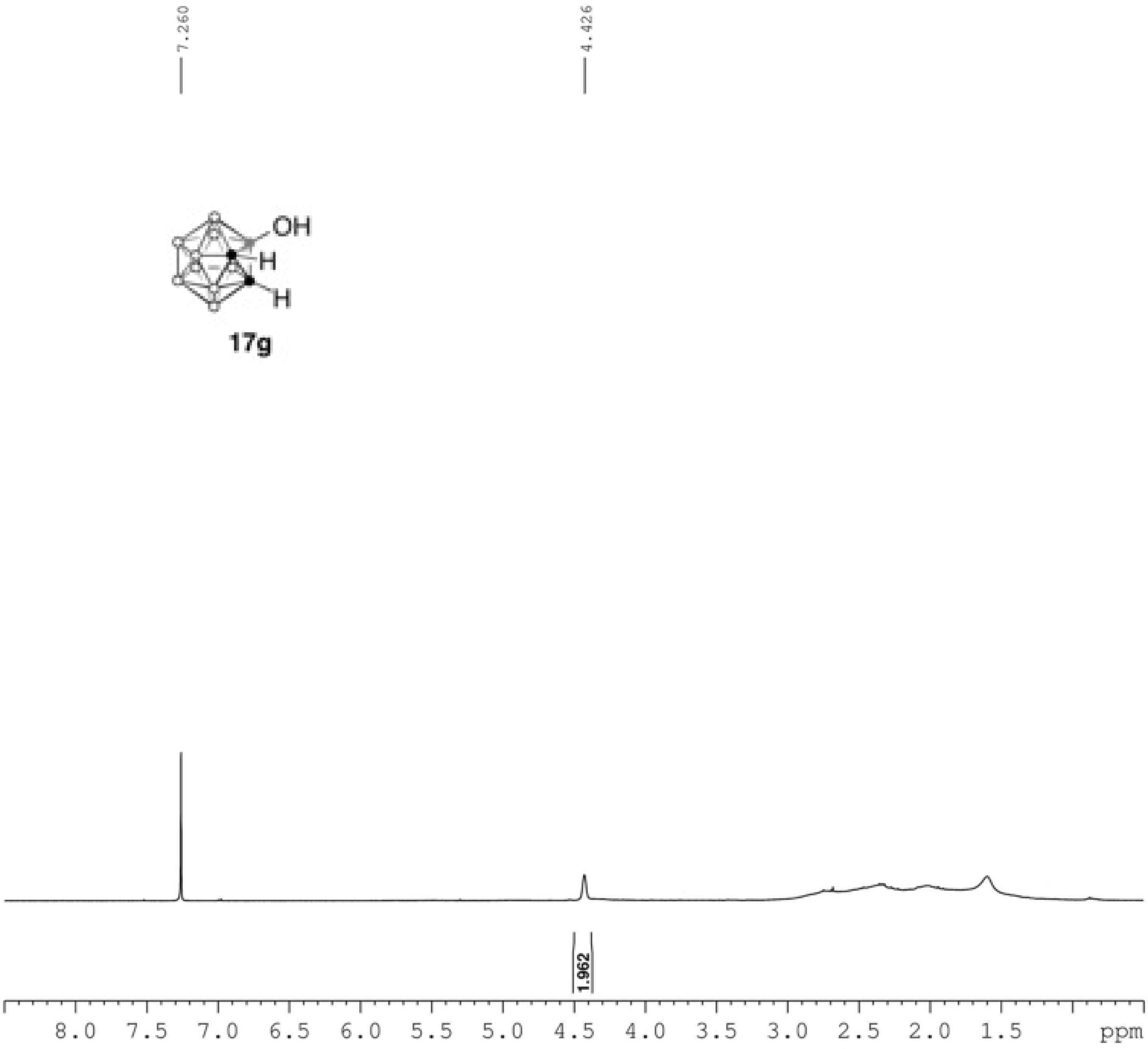

17g

ZD-3-OH-CDCl3-H

Current Data Parameters
NAME ZD-3-OH-CDCl3-H
EXPNO 1
PROCNO 1

F2 - Acquisition Parameters
Date_ 20160126
Time 22.59 h
INSTRUM spect
PROBHD Z824601_0021 (
PULPROG zg
TD 65536
SOLVENT CDCl3
NS 16
DS 0
SWH 8012.820 Hz
FIDRES 0.122266 Hz
AQ 4.0894465 sec
RG 181
DW 62.400 usec
DE 6.50 usec
TE 294.0 K
D1 1.00000000 sec
TD0 1
SFO1 400.1316005 MHz
NUC1 1H
P1 15.00 usec
PLW1 8.31000042 W

F2 - Processing parameters
SI 65536
SF 400.1300098 MHz
WDW EM
SSB 0
LB 0.30 Hz
GB 0
PC 1.00

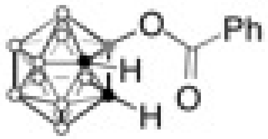

18

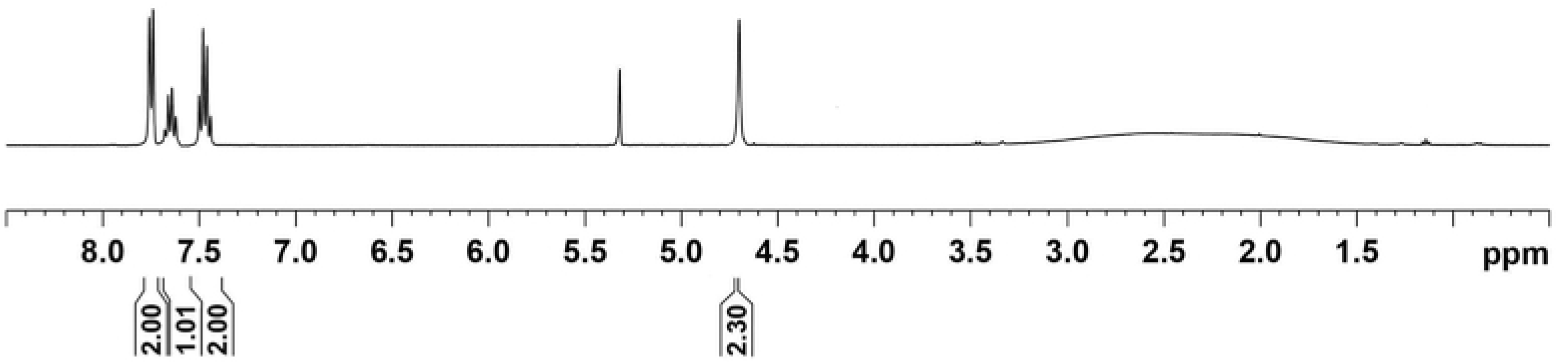

NAME	ZD-3-PhCOOH-CD2Cl2-H	
EXPNO	1	
PROCNO	1	
Date_	20151115	
Time	13.01	h
INSTRUM	spect	
PROBHD	Z824601_0021 (	
PULPROG	zg30	
TD	65536	
SOLVENT	CD2Cl2	
NS	4	
DS	2	
SWH	8012.820	Hz
FIDRES	0.122266	Hz
AQ	4.0894966	sec
RG	181	
DW	62.400	usec
DE	6.50	usec
TE	295.3	K
D1	1.00000000	sec
TD0	1	
SFO1	400.1324708	MHz
NUC1	1H	
P1	15.00	usec
SI	65536	
SF	400.1300148	MHz
WDW	EM	
SSB	0	
LB	0.30	Hz
GB	0	
PC	1.00	

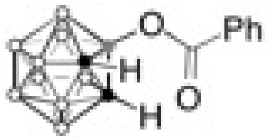

18

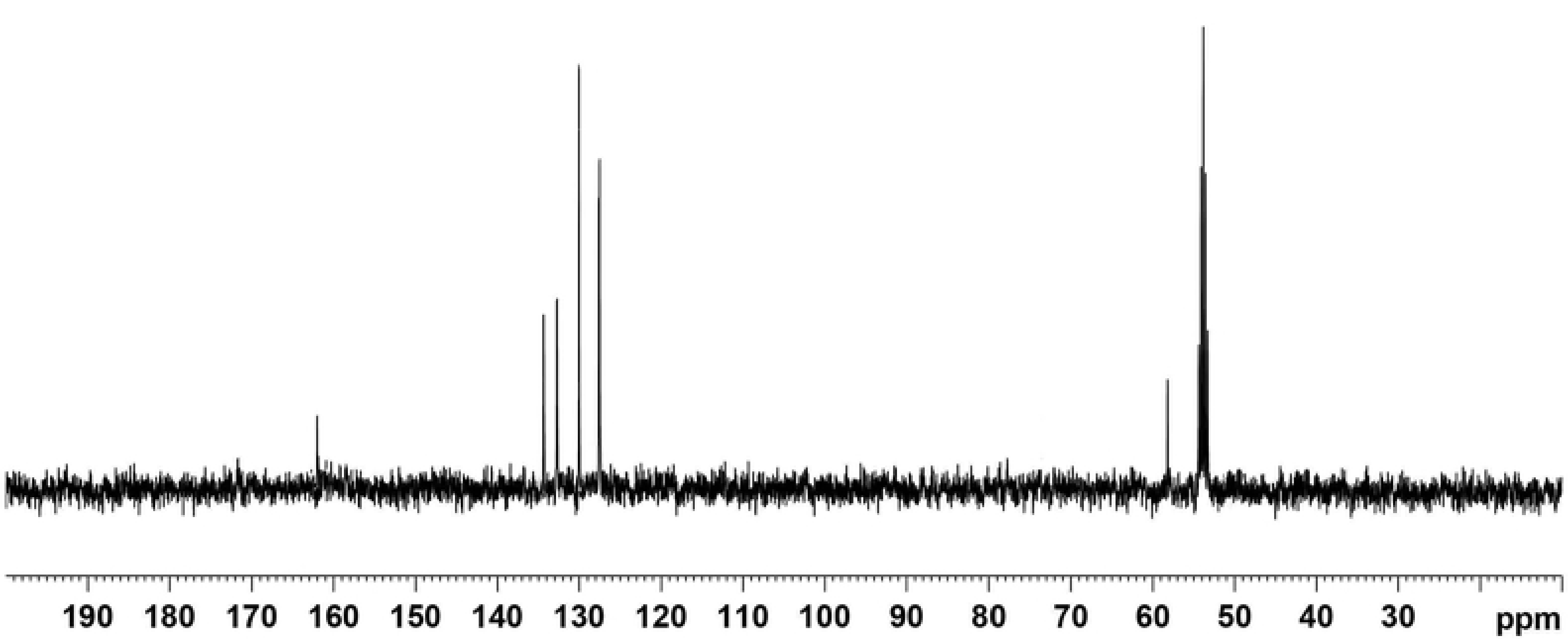

NAME	ZD-3-PhCOOH-CD2Cl2-C
EXPNO	1
PROCNO	1
Date_	2015115
Time	13.04 h
INSTRUM	spect
PROBHD	Z824601_0021 (
PULPROG	zgdc
TD	131072
SOLVENT	CD2Cl2
NS	48
DS	0
SWH	25252.525 Hz
FIDRES	0.192661 Hz
AQ	2.5952756 sec
RG	203
DW	19.800 usec
DE	6.50 usec
TE	295.4 K
D1	1.00000000 sec
D11	0.03000000 sec
TD0	1
SFO1	100.6227690 MHz
NUC1	13C
P1	9.50 usec
SI	131072
SF	100.6127290 MHz
WDW	EM
SSB	0
LB	3.00 Hz
GB	0
PC	1.40

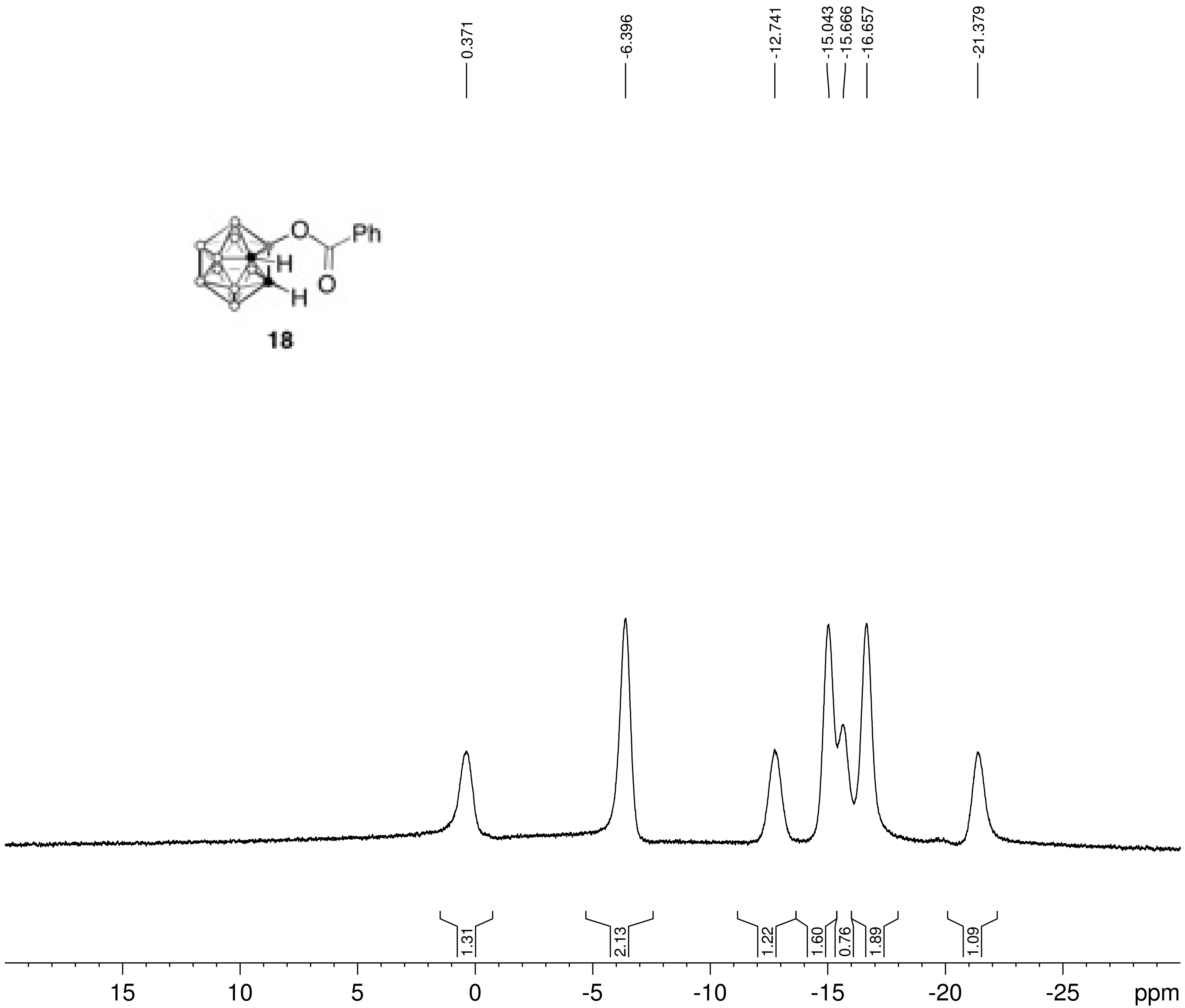

18

ZD-3-COOH-CD2Cl2-B(de)

Current Data Parameters
NAME ZD-3-DMSO-CD2Cl2-B(de)
EXPNO 1
PROCNO 1

F2 - Acquisition Parameters
Date_ 20160131
Time 20.49 h
INSTRUM spect
PROBHD Z108618_0257 (
PULPROG zgdc
TD 65536
SOLVENT D2O
NS 8
DS 4
SWH 24038.461 Hz
FIDRES 0.366798 Hz
AQ 1.3631488 sec
RG 322
DW 20.800 usec
DE 6.50 usec
TE 298.4 K
D1 2.00000000 sec
D11 0.03000000 sec
TD0 1
SFO1 128.4096890 MHz
NUC1 11B
P1 7.50 usec
PLW1 55.09999847 W
SFO2 400.2316009 MHz
NUC2 1H
CPDPRG[2 waltz16
PCPD2 90.00 usec
PLW2 13.56000042 W
PLW12 0.27428001 W

F2 - Processing parameters
SI 32768
SF 128.4097724 MHz
WDW EM
SSB 0
LB 1.00 Hz
GB 0
PC 1.40

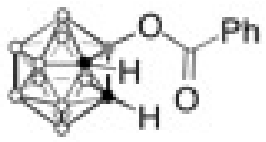

18

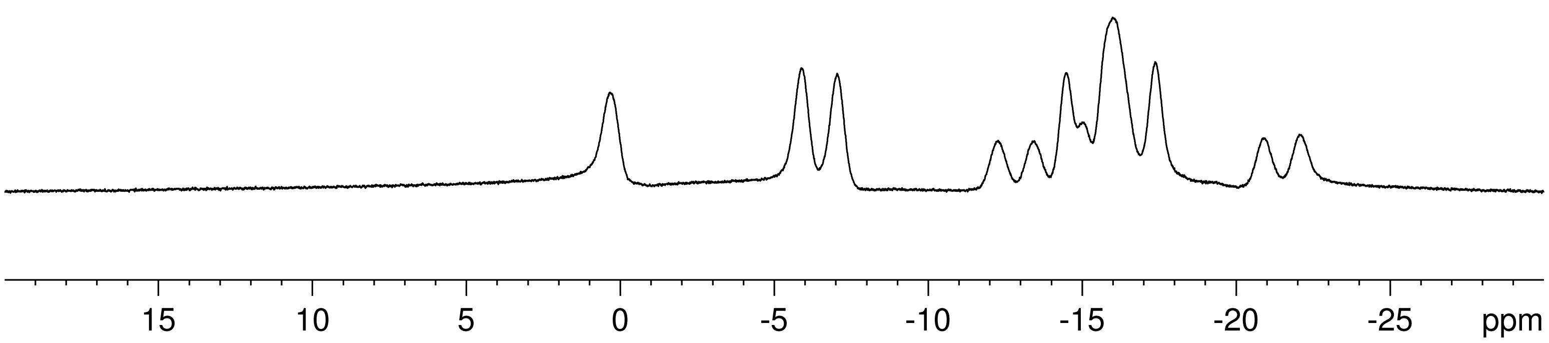

ZD-3-PhCOOH-CD2Cl2-B(c)

Current Data Parameters
NAME ZD-3-DMSO-CD2Cl2-B(c)
EXPNO 1
PROCNO 1

F2 - Acquisition Parameters
Date_ 20160131
Time 20.51 h
INSTRUM spect
PROBHD Z108618_0257 (
PULPROG zg
TD 65536
SOLVENT D2O
NS 24
DS 2
SWH 24038.461 Hz
FIDRES 0.366798 Hz
AQ 1.3631488 sec
RG 101
DW 20.800 usec
DE 6.50 usec
TE 298.2 K
D1 2.00000000 sec
TD0 1
SFO1 128.4096890 MHz
NUC1 11B
P1 7.50 usec
PLW1 55.09999847 W

F2 - Processing parameters
SI 32768
SF 128.4097843 MHz
WDW EM
SSB 0
LB 1.00 Hz
GB 0
PC 1.40

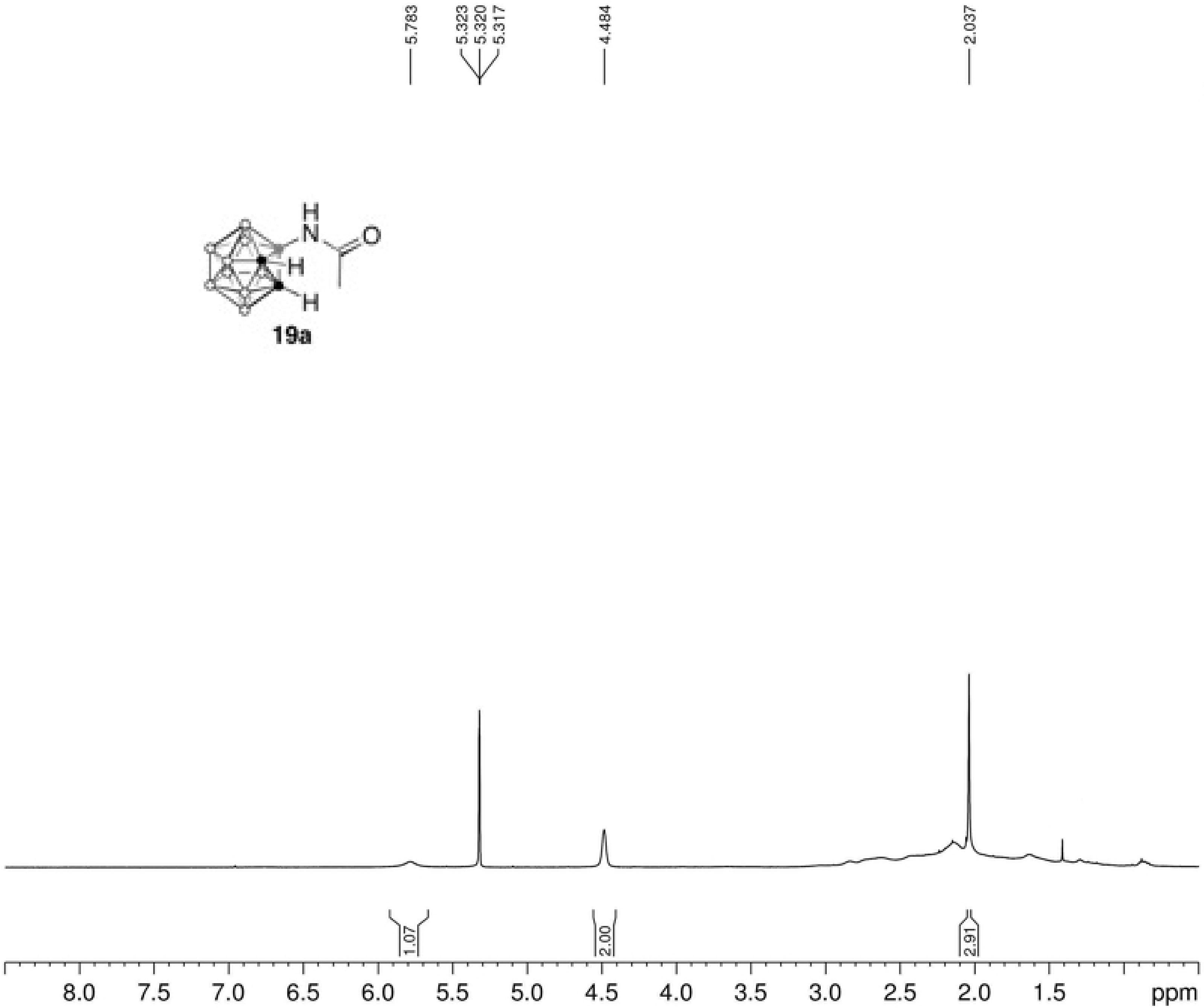

19a

ZD-3-CH3CN-CD2Cl2-H

Current Data Parameters
NAME ZD-3-CH3CN-CD2Cl2-H
EXPNO 1
PROCNO 1

F2 - Acquisition Parameters
Date_ 20160131
Time 20.52 h
INSTRUM spect
PROBHD Z108618_0257 (
PULPROG zg30
TD 65536
SOLVENT CD2Cl2
NS 10
DS 2
SWH 8012.820 Hz
FIDRES 0.122266 Hz
AQ 4.0894465 sec
RG 144
DW 62.400 usec
DE 6.50 usec
TE 298.2 K
D1 1.00000000 sec
TD0 1
SFO1 400.2324714 MHz
NUC1 1H
P1 12.80 usec
PLW1 13.56000042 W

F2 - Processing parameters
SI 65536
SF 400.2300155 MHz
WDW EM
SSB 0
LB 0.30 Hz
GB 0
PC 1.00

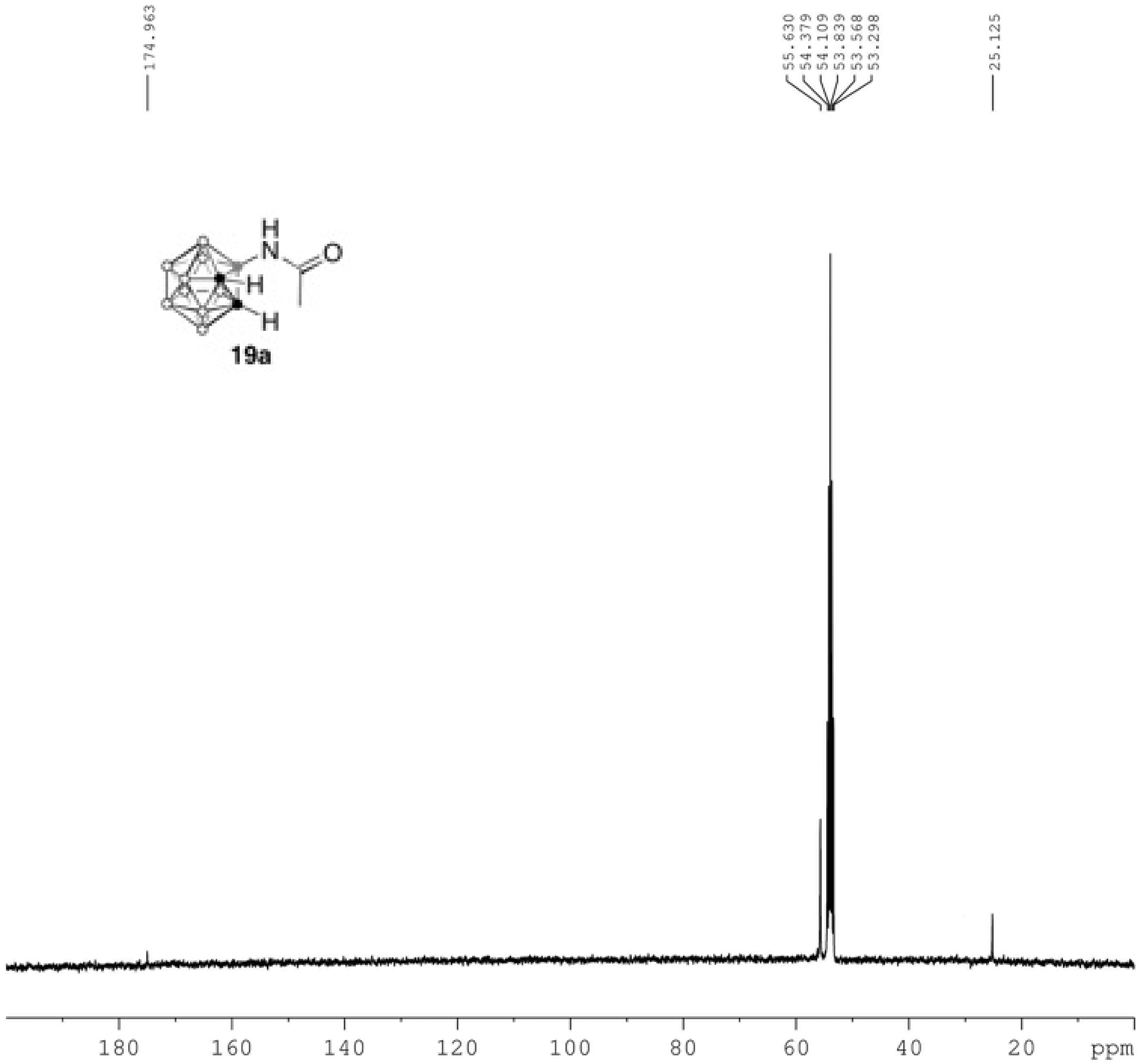


```
ZD-3-CH3CN-CD2Cl2-C

Bruker Advance III 400

Current Data Parameters
NAME     ZD-3-CH3CN-CD2Cl2-C
EXPNO                      1
PROCNO                     1

F2 - Acquisition Parameters
Date_              20160201
Time                   0.14 h
INSTRUM               spect
PROBHD   Z824601_0021 (
PULPROG                zgdc
TD                   131072
SOLVENT              CD2Cl2
NS                     8118
DS                        0
SWH              25252.525 Hz
FIDRES            0.192661 Hz
AQ               2.5952256 sec
RG                      203
DW                   19.800 usec
DE                     6.50 usec
TE                    295.7 K
D1               1.00000000 sec
D11              0.03000000 sec
TD0                       1
SFO1           100.6227690 MHz
NUC1                    13C
P1                     9.50 usec
PLW1            41.25000000 W
SFO2           400.1320007 MHz
NUC2                     1H
CPDPRG[2            waltz16
PCPD2                 90.00 usec
PLW2             8.31000042 W
PLW12            0.23083000 W

F2 - Processing parameters
SI                   131072
SF             100.6127271 MHz
WDW                      EM
SSB      0
LB                     3.00 Hz
GB       0
PC                     1.40
```

19a

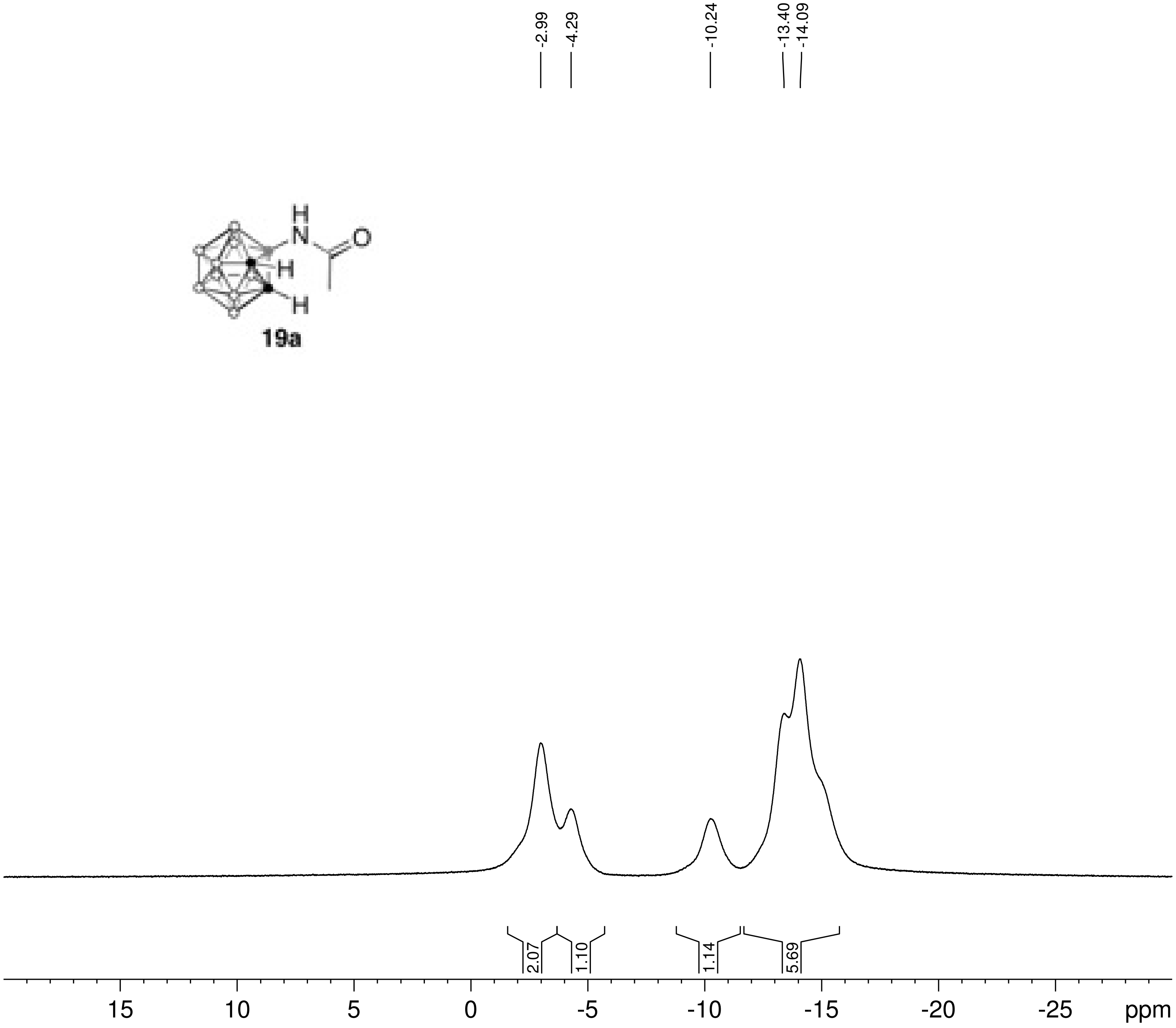

ZD-3-CH3CN-CDCl3-B(de)

Current Data Parameters

NAME	ZD-3-N3-CDCl3-B(de)	
EXPNO	1	
PROCNO	1	

F2 - Acquisition Parameters

Date_	20160131	
Time	20.56	h
INSTRUM	spect	
PROBHD	Z108618_0257 (	
PULPROG	zgdc	
TD	65536	
SOLVENT	CD2Cl2	
NS	8	
DS	4	
SWH	24038.461	Hz
FIDRES	0.366798	Hz
AQ	1.3631488	sec
RG	203	
DW	20.800	usec
DE	6.50	usec
TE	296.2	K
D1	2.00000000	sec
D11	0.03000000	sec
TD0	1	
SFO1	128.4096890	MHz
NUC1	11B	
P1	7.50	usec
PLW1	55.09999847	W
SFO2	400.2316009	MHz
NUC2	1H	
CPDPRG[2	waltz16	
PCPD2	90.00	usec
PLW2	13.56000042	W
PLW12	0.27428001	W

F2 - Processing parameters

SI	32768	
SF	128.4096582	MHz
WDW	EM	
SSB	0	
LB	1.00	Hz
GB	0	
PC	1.40	

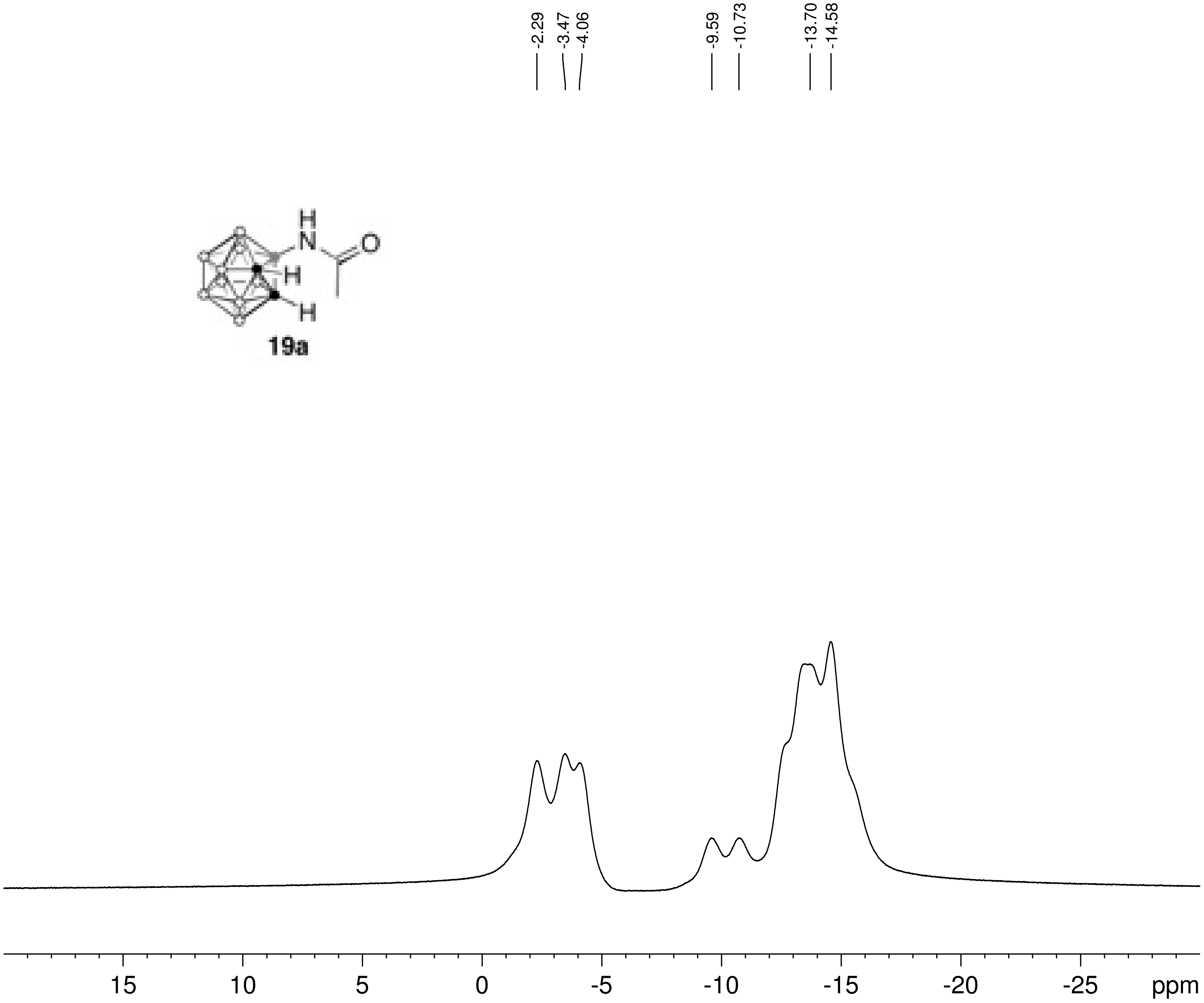

19a

ZD-3-CH3CN-CD2Cl2-B(c)

Current Data Parameters
NAME ZD-3-N3-CDCl3-B(c)
EXPNO 1
PROCNO 1

F2 - Acquisition Parameters
Date_ 20160131
Time 20.58 h
INSTRUM spect
PROBHD Z108618_0257 (
PULPROG zg
TD 65536
SOLVENT CD2Cl2
NS 43
DS 2
SWH 24038.461 Hz
FIDRES 0.366798 Hz
AQ 1.3631488 sec
RG 101
DW 20.800 usec
DE 6.50 usec
TE 295.8 K
D1 2.00000000 sec
TD0 1
SFO1 128.4096890 MHz
NUC1 11B
P1 7.50 usec
PLW1 55.09999847 W

F2 - Processing parameters
SI 32768
SF 128.4096452 MHz
WDW EM
SSB 0
LB 1.00 Hz
GB 0
PC 1.40

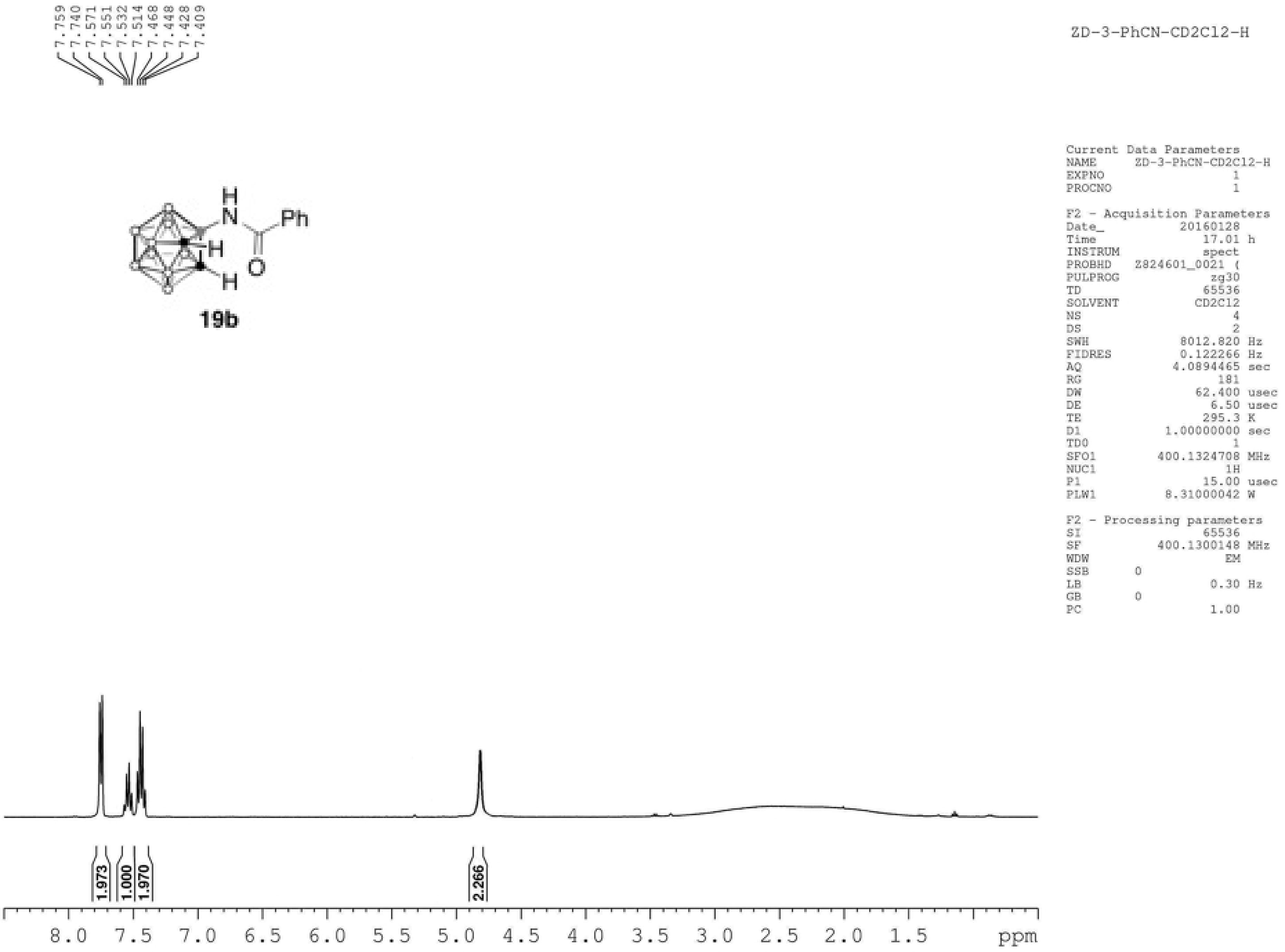
7.759
7.740
7.571
7.551
7.532
7.514
7.468
7.448
7.428
7.409
ZD-3-PhCN-CD2Cl2-H
H
N
Ph
H
O
H
19b
Current Data Parameters
NAME ZD-3-PhCN-CD2Cl2-H
EXPNO 1
PROCNO 1
F2 - Acquisition Parameters
Date_ 20160128
Time 17.01 h
INSTRUM spect
PROBHD Z824601_0021 (
PULPROG zg30
TD 65536
SOLVENT CD2Cl2
NS 4
DS 2
SWH 8012.820 Hz
FIDRES 0.122266 Hz
AQ 4.0894465 sec
RG 181
DW 62.400 usec
DE 6.50 usec
TE 295.3 K
D1 1.00000000 sec
TD0 1
SFO1 400.1324708 MHz
NUC1 1H
P1 15.00 usec
PLW1 8.31000042 W
F2 - Processing parameters
SI 65536
SF 400.1300148 MHz
WDW EM
SSB 0
LB 0.30 Hz
GB 0
PC 1.00
1.973
1.000
1.970
2.266
8.0
7.5
7.0
6.5
6.0
5.5
5.0
4.5
4.0
3.5
3.0
2.5
2.0
1.5
ppm

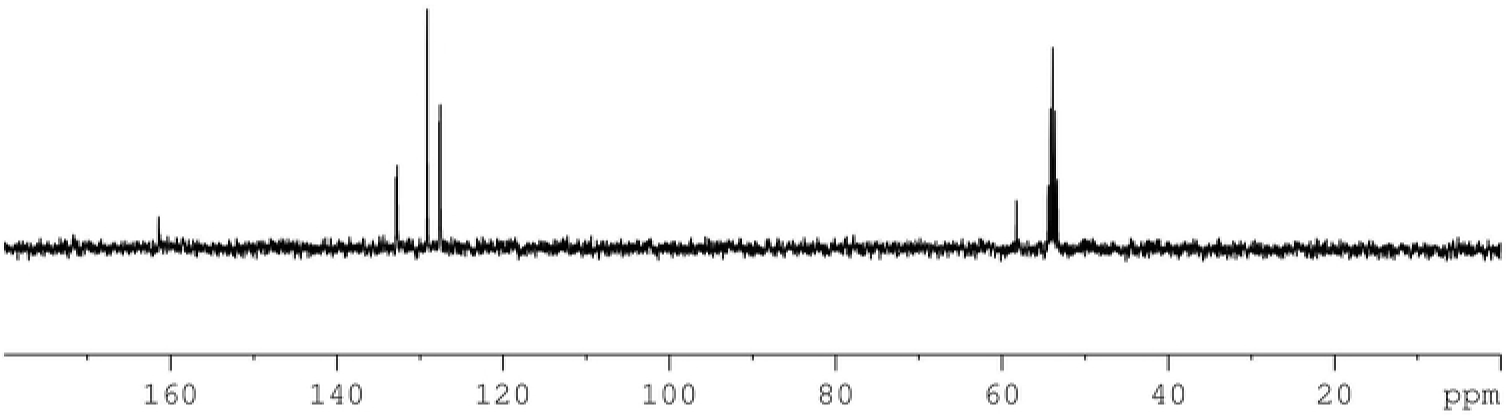

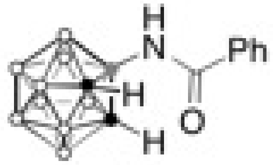

19b

ZD-3-PhCN-CD2Cl2-C

Current Data Parameters
NAME ZD-3-PhCN-CD2Cl2-C
EXPNO 1
PROCNO 1

F2 - Acquisition Parameters
Date_ 20160128
Time 17.04 h
INSTRUM spect
PROBHD Z824601_0021 (
PULPROG zgdc
TD 131072
SOLVENT CD2Cl2
NS 48
DS 0
SWH 25252.525 Hz
FIDRES 0.192661 Hz
AQ 2.5952256 sec
RG 203
DW 19.800 usec
DE 6.50 usec
TE 295.4 K
D1 1.00000000 sec
D11 0.03000000 sec
TD0 1
SFO1 100.6227690 MHz
NUC1 13C
P1 9.50 usec
PLW1 41.25000000 W
SFO2 400.1320007 MHz
NUC2 1H
CPDPRG[2 waltz16
PCPD2 90.00 usec
PLW2 8.31000042 W
PLW12 0.23083000 W

F2 - Processing parameters
SI 131072
SF 100.6127290 MHz
WDW EM
SSB 0
LB 3.00 Hz
GB 0
PC 1.40

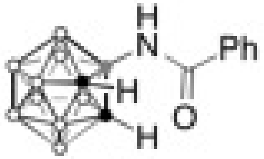

19b

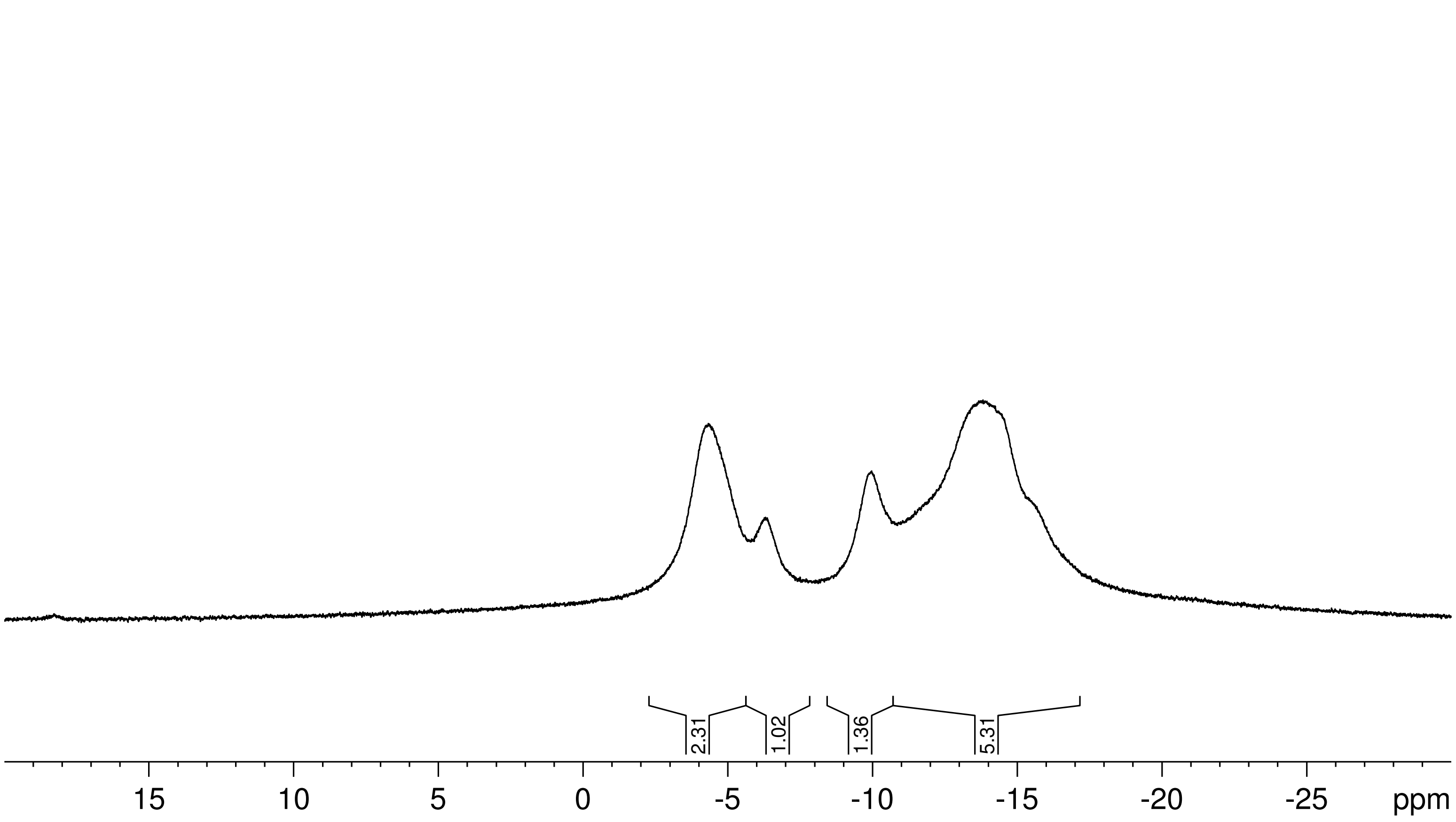

ZD-3-PhCN-CD2Cl2-B(de)

```
Current Data Parameters
NAME      ZD-3-PhCN-CD2Cl2-B(de)
EXPNO                    1
PROCNO                   1

F2 - Acquisition Parameters
Date_             20160128
Time                 17.38 h
INSTRUM              spect
PROBHD    Z108618_0257 (
PULPROG               zgdc
TD                   65536
SOLVENT                D2O
NS                      10
DS                       4
SWH              24038.461 Hz
FIDRES            0.366798 Hz
AQ               1.3631488 sec
RG                     256
DW                  20.800 usec
DE                    6.50 usec
TE                   298.3 K
D1              2.00000000 sec
D11             0.03000000 sec
TD0                      1
SFO1           128.4096890 MHz
NUC1                   11B
P1                    7.50 usec
PLW1           55.09999847 W
SFO2           400.2316009 MHz
NUC2                    1H
CPDPRG[2           waltz16
PCPD2                90.00 usec
PLW2           13.56000042 W
PLW12           0.27428001 W

F2 - Processing parameters
SI                   32768
SF             128.4097615 MHz
WDW                     EM
SSB       0
LB                    1.00 Hz
GB        0
PC                    1.40
```

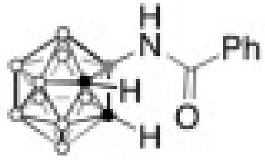

19b

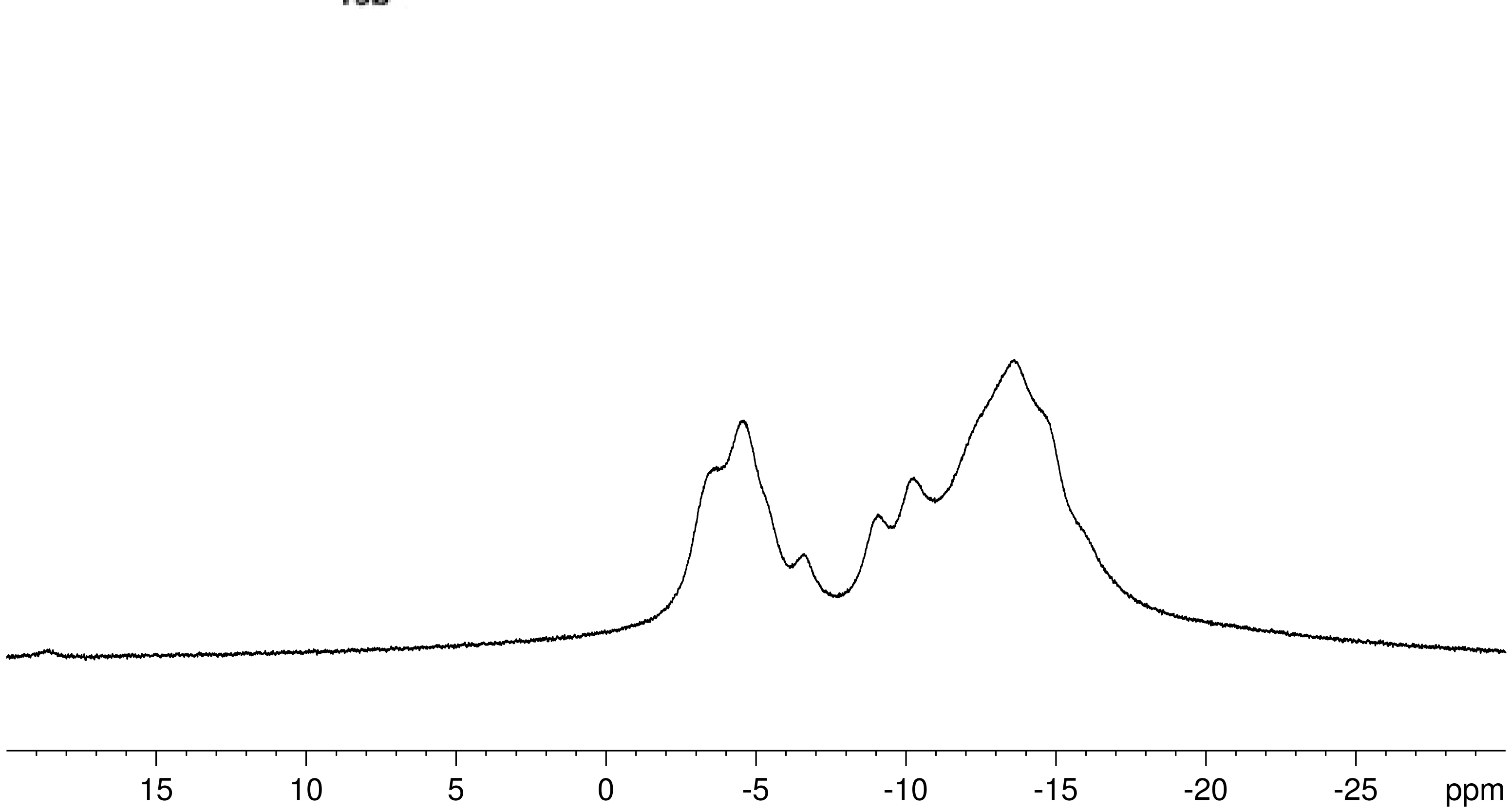

ZD-3-PhCN-CD2Cl2-B(c)

Current Data Parameters
NAME ZD-3-PhCN-CD2Cl2-B(c)
EXPNO 1
PROCNO 1

F2 - Acquisition Parameters
Date_ 20160128
Time 17.39 h
INSTRUM spect
PROBHD Z108618_0257 (
PULPROG zg
TD 65536
SOLVENT D2O
NS 19
DS 2
SWH 24038.461 Hz
FIDRES 0.366798 Hz
AQ 1.3631488 sec
RG 101
DW 20.800 usec
DE 6.50 usec
TE 298.3 K
D1 2.00000000 sec
TD0 1
SFO1 128.4096890 MHz
NUC1 11B
P1 7.50 usec
PLW1 55.09999847 W

F2 - Processing parameters
SI 32768
SF 128.4097221 MHz
WDW EM
SSB 0
LB 1.00 Hz
GB 0
PC 1.40

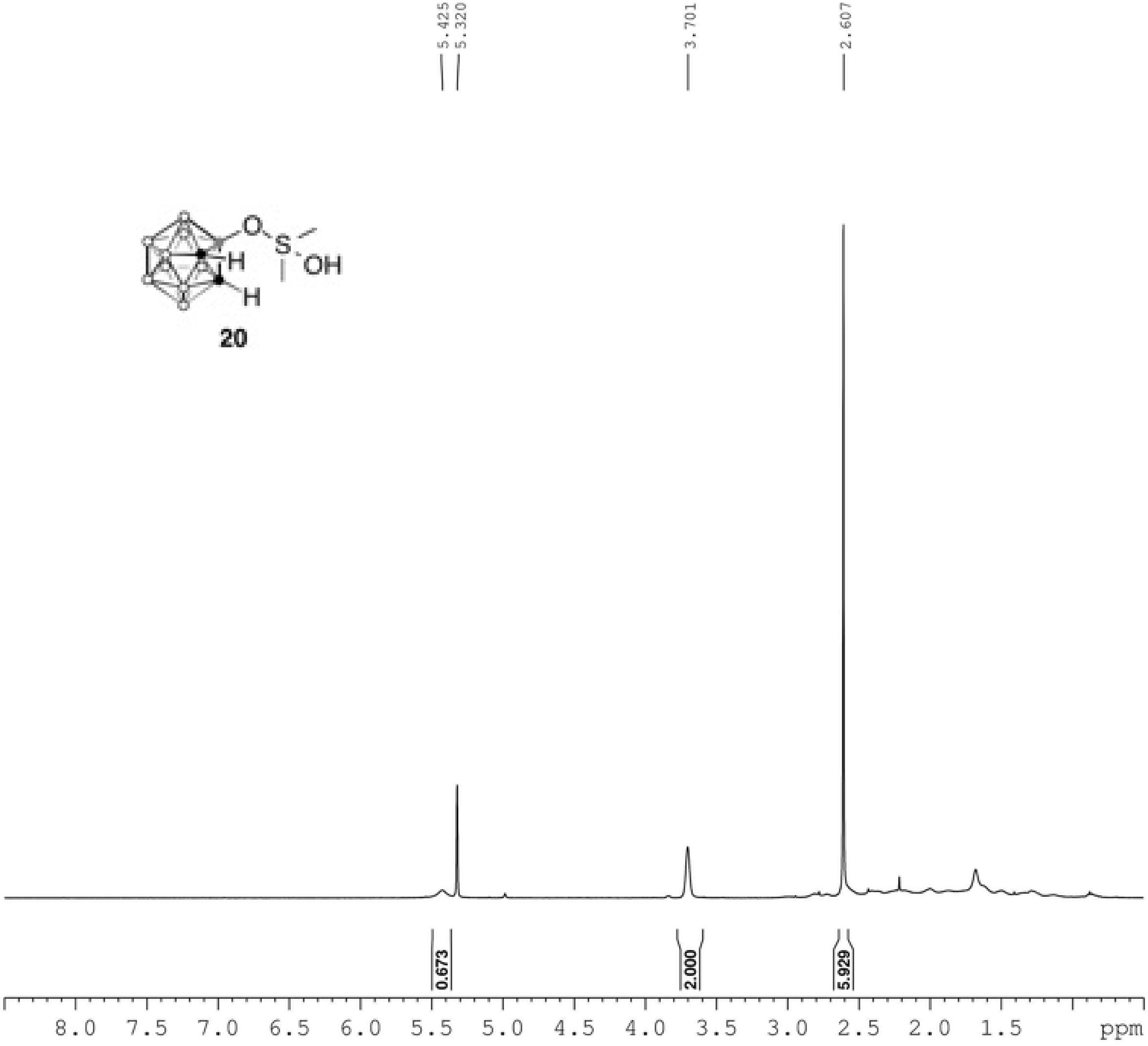

ZD-3-DMSO-CD2Cl2-H

```
Current Data Parameters
NAME       ZD-3-DMSO-CD2Cl2-H
EXPNO                       1
PROCNO                      1

F2 - Acquisition Parameters
Date_              20160128
Time                  16.21 h
INSTRUM               spect
PROBHD    Z824601_0021 (
PULPROG                zg30
TD                    65536
SOLVENT              CD2Cl2
NS                        8
DS                        2
SWH              8012.820 Hz
FIDRES           0.122266 Hz
AQ              4.0894465 sec
RG                      203
DW                   62.400 usec
DE                     6.50 usec
TE                    295.1 K
D1               1.00000000 sec
TD0                       1
SFO1          400.1324708 MHz
NUC1                     1H
P1                    15.00 usec
PLW1             8.31000042 W

F2 - Processing parameters
SI                    65536
SF            400.1300151 MHz
WDW                      EM
SSB       0
LB                     0.30 Hz
GB        0
PC                     1.00
```

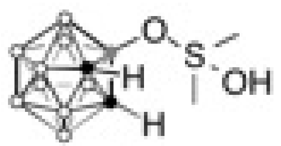

20

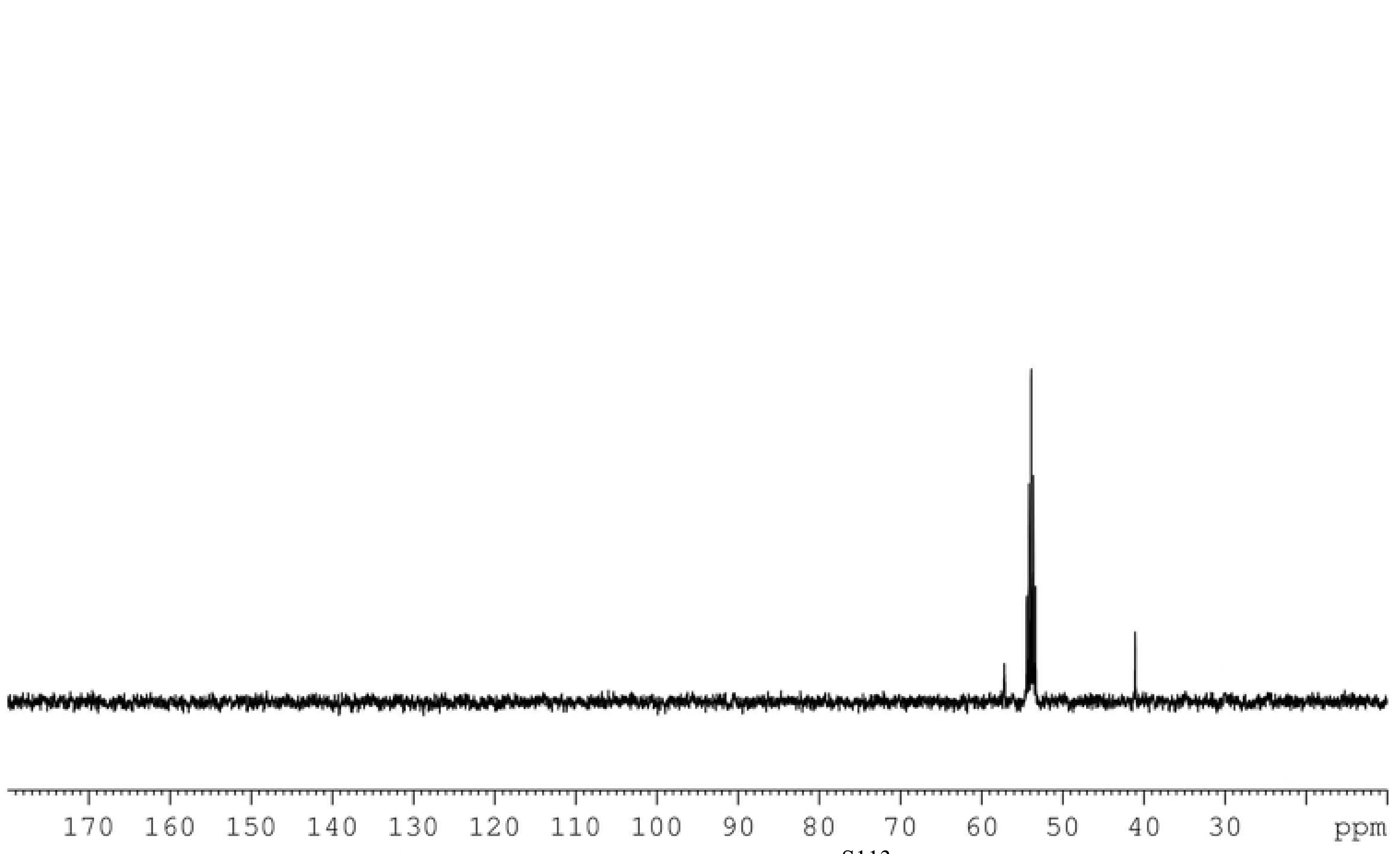

ZD-3-DMSO-CD2Cl2-C

```
Current Data Parameters
NAME      ZD-3-DMSO-CD2Cl2-C
EXPNO                    1
PROCNO                   1

F2 - Acquisition Parameters
Date_             20160128
Time                 16.27 h
INSTRUM              spect
PROBHD    Z824601_0021 (
PULPROG               zgdc
TD                  131072
SOLVENT             CD2Cl2
NS                     128
DS                       0
SWH              25252.525 Hz
FIDRES            0.192661 Hz
AQ               2.5952256 sec
RG                     203
DW                  19.800 usec
DE                    6.50 usec
TE                   295.3 K
D1              1.00000000 sec
D11             0.03000000 sec
TD0                      1
SFO1           100.6227690 MHz
NUC1                   13C
P1                    9.50 usec
PLW1           41.25000000 W
SFO2           400.1320007 MHz
NUC2                    1H
CPDPRG[2           waltz16
PCPD2                90.00 usec
PLW2            8.31000042 W
PLW12           0.23083000 W

F2 - Processing parameters
SI                  131072
SF             100.6127282 MHz
WDW                     EM
SSB      0
LB                    3.00 Hz
GB       0
PC                    1.40
```

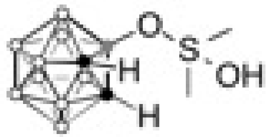

20

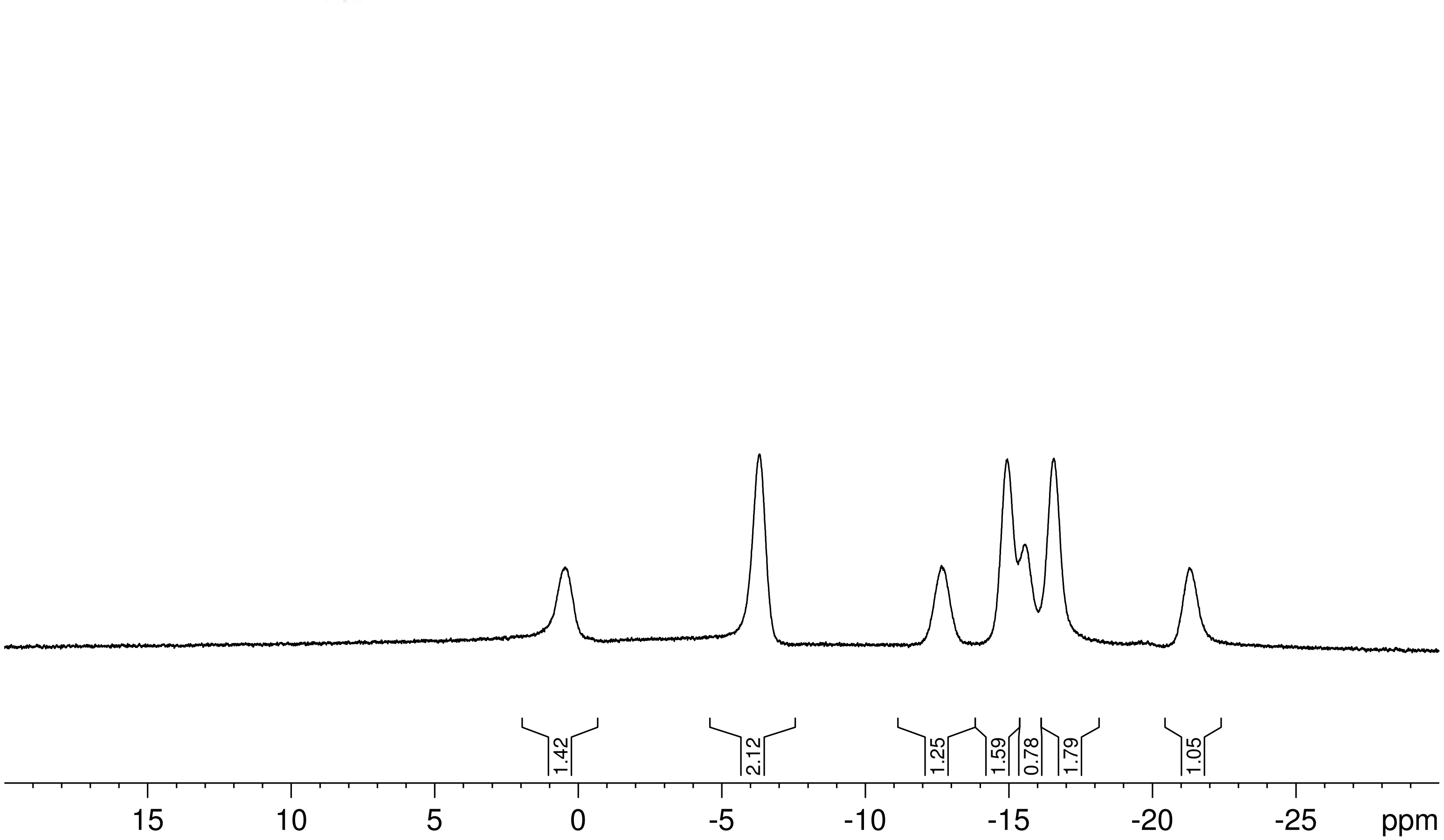

ZD-3-DMSO-CD2Cl2-B(de)

```
Current Data Parameters
NAME      ZD-3-DMSO-CD2Cl2-B(de)
EXPNO                      1
PROCNO                     1

F2 - Acquisition Parameters
Date_             20160128
Time                 17.34 h
INSTRUM              spect
PROBHD    Z108618_0257 (
PULPROG               zgdc
TD                   65536
SOLVENT                D2O
NS                       8
DS                       4
SWH             24038.461 Hz
FIDRES           0.366798 Hz
AQ              1.3631488 sec
RG                     322
DW                  20.800 usec
DE                    6.50 usec
TE                   298.4 K
D1             2.00000000 sec
D11            0.03000000 sec
TD0                      1
SFO1          128.4096890 MHz
NUC1                   11B
P1                    7.50 usec
PLW1           55.09999847 W
SFO2          400.2316009 MHz
NUC2                    1H
CPDPRG[2           waltz16
PCPD2                90.00 usec
PLW2           13.56000042 W
PLW12           0.27428001 W

F2 - Processing parameters
SI                   32768
SF            128.4097615 MHz
WDW                     EM
SSB        0
LB                    1.00 Hz
GB         0
PC                    1.40
```

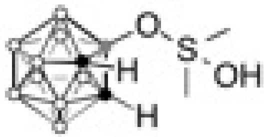
20

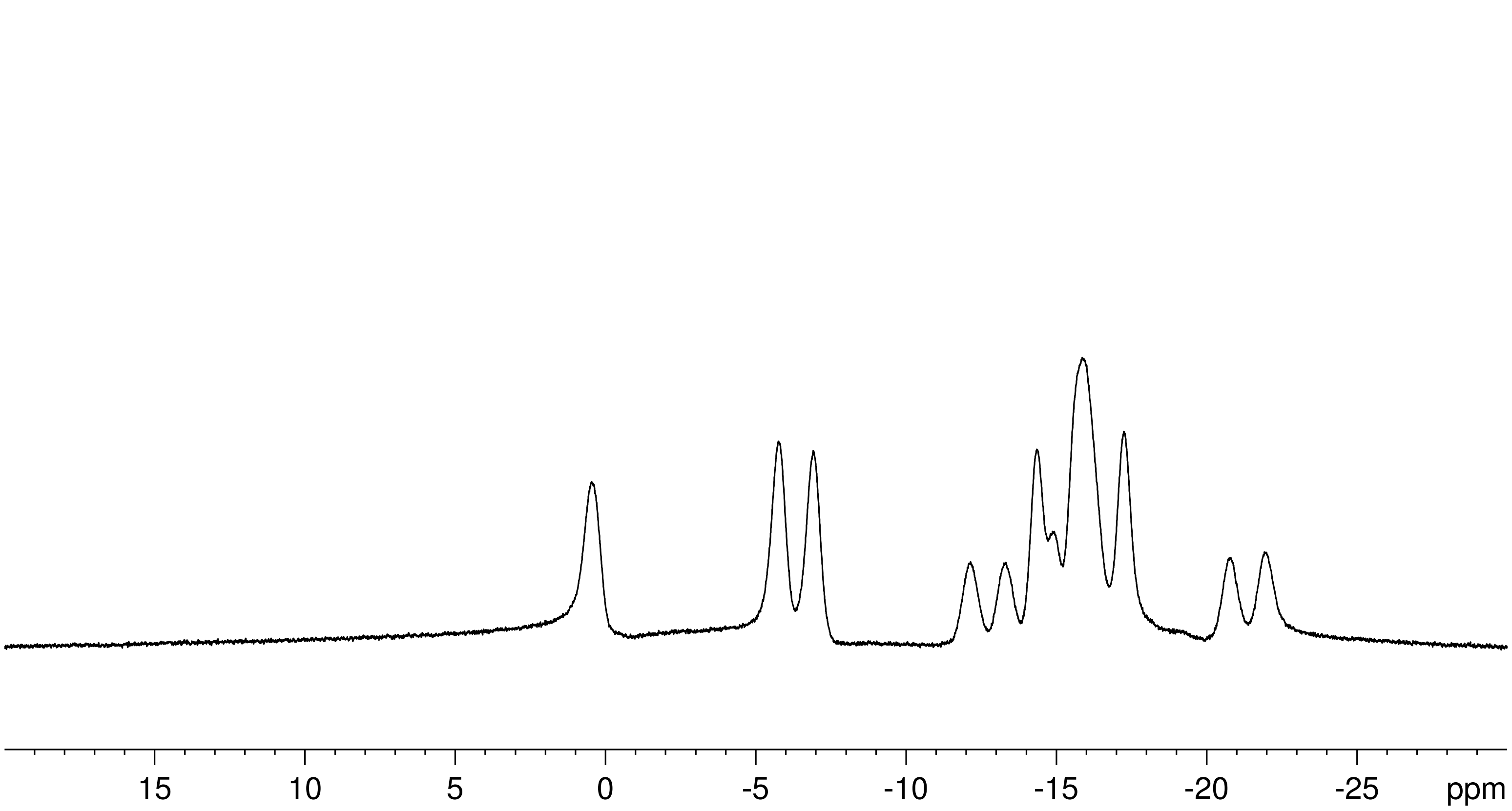

ZD-3-DMSO-CD2Cl2-B(c)

Current Data Parameters
NAME ZD-3-DMSO-CD2Cl2-B(c)
EXPNO 1
PROCNO 1

F2 - Acquisition Parameters
Date_ 20160128
Time 17.36 h
INSTRUM spect
PROBHD Z108618_0257 (
PULPROG zg
TD 65536
SOLVENT D2O
NS 24
DS 2
SWH 24038.461 Hz
FIDRES 0.366798 Hz
AQ 1.3631488 sec
RG 101
DW 20.800 usec
DE 6.50 usec
TE 298.2 K
D1 2.00000000 sec
TD0 1
SFO1 128.4096890 MHz
NUC1 11B
P1 7.50 usec
PLW1 55.09999847 W

F2 - Processing parameters
SI 32768
SF 128.4097682 MHz
WDW EM
SSB 0
LB 1.00 Hz
GB 0
PC 1.40

ZD-787-2-acetone-H

Bruker Advance III 400

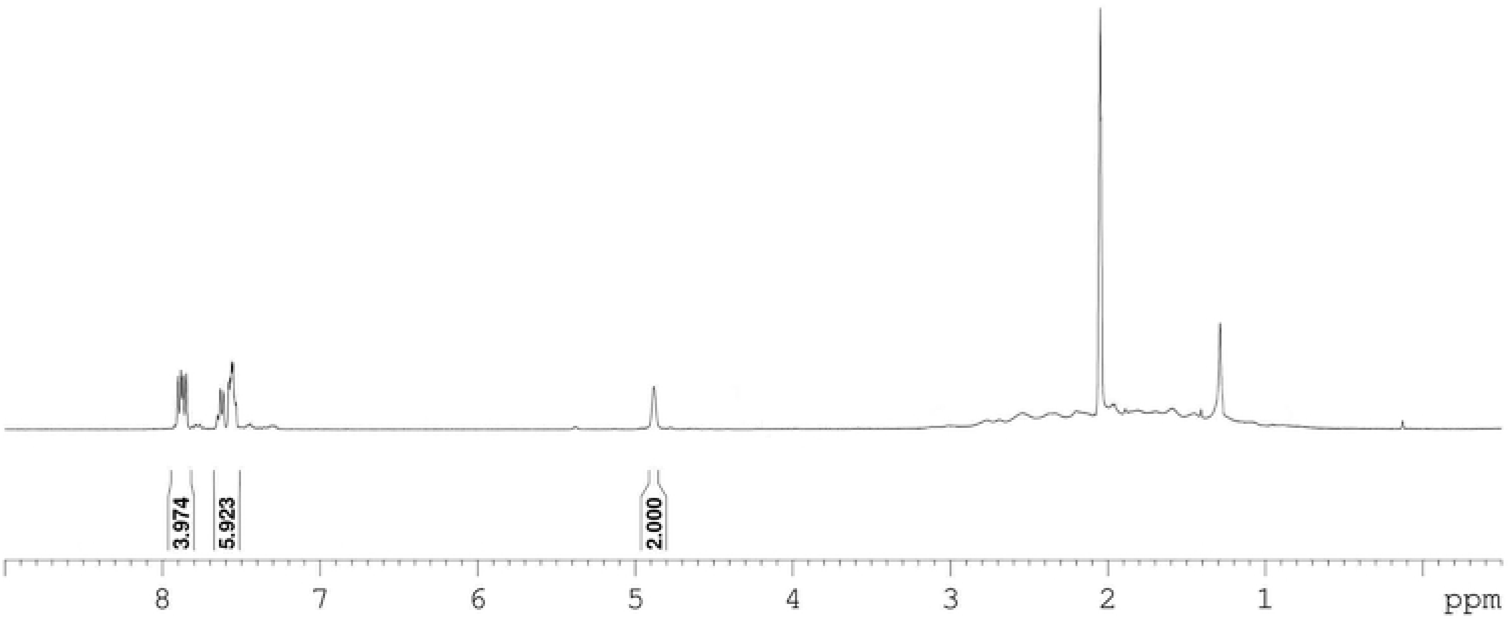

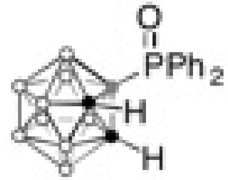

21a

```
NAME       ZD-787-2-acetone-H
EXPNO                       1
PROCNO                      1
Date_                20141016
Time                    14.58
INSTRUM                 spect
PROBHD      5 mm PADUL 13C
PULPROG                    zg
TD                      65536
SOLVENT               Acetone
NS                          8
DS                          0
SWH              10000.000 Hz
FIDRES            0.152588 Hz
AQ               3.2768500 sec
RG                        161
DW                 50.000 usec
DE                   6.50 usec
TE                  673.2 K
D1              1.00000000 sec
TD0                         1

======== CHANNEL f1 ========
NUC1                       1H
P1                  14.83 usec
PL1                  0.00 dB
PL1W           8.31434441 W
SFO1         400.1318000 MHz
SI                      65536
SF           400.1300056 MHz
WDW                        EM
SSB                         0
LB                   0.30 Hz
GB                          0
PC                       1.00
```

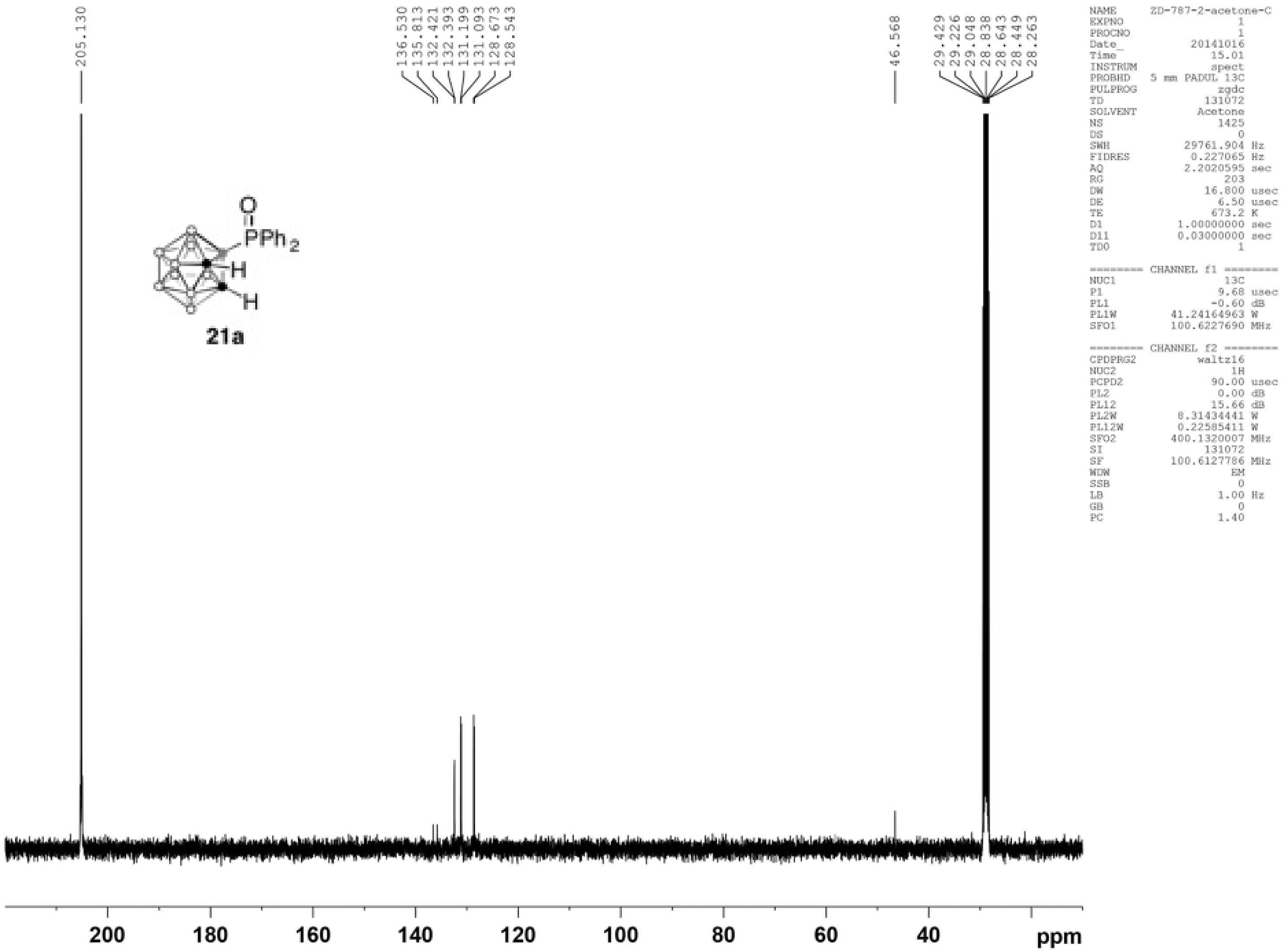
205.130
136.530
135.813
132.421
132.393
131.199
131.093
128.673
128.543
46.568
29.429
29.226
29.048
28.838
28.643
28.449
28.263
O
PPh2
H
H
21a
NAME ZD-787-2-acetone-C
EXPNO 1
PROCNO 1
Date_ 20141016
Time 15.01
INSTRUM spect
PROBHD 5 mm PADUL 13C
PULPROG zgdc
TD 131072
SOLVENT Acetone
NS 1425
DS 0
SWH 29761.904 Hz
FIDRES 0.227065 Hz
AQ 2.2020595 sec
RG 203
DW 16.800 usec
DE 6.50 usec
TE 673.2 K
D1 1.00000000 sec
D11 0.03000000 sec
TD0 1
======== CHANNEL f1 ========
NUC1 13C
P1 9.68 usec
PL1 -0.60 dB
PL1W 41.24164963 W
SFO1 100.6227690 MHz
======== CHANNEL f2 ========
CPDPRG2 waltz16
NUC2 1H
PCPD2 90.00 usec
PL2 0.00 dB
PL12 15.66 dB
PL2W 8.31434441 W
PL12W 0.22585411 W
SFO2 400.1320007 MHz
SI 131072
SF 100.6127786 MHz
WDW EM
SSB 0
LB 1.00 Hz
GB 0
PC 1.40
200
180
160
140
120
100
80
60
40
ppm

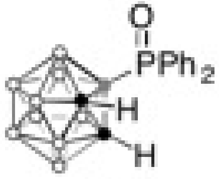

21a

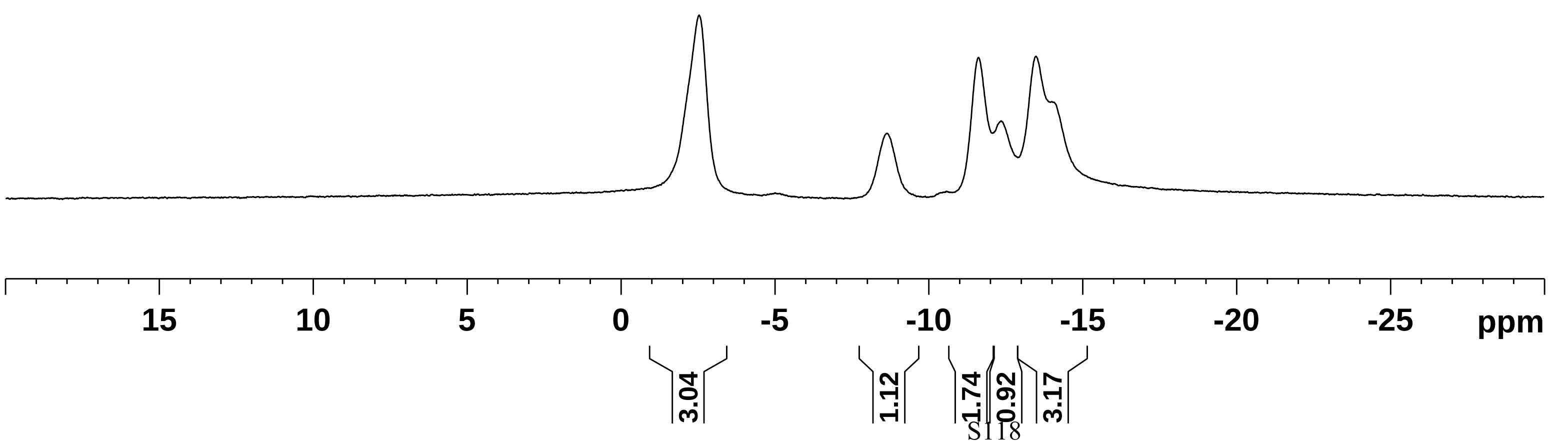


```
NAME     ZD-787-2-acetone-B(de)
EXPNO                     1
PROCNO                    1
Date_              20141016
Time                  14.33
INSTRUM               spect
PROBHD   5 mm PABBO BB-
PULPROG                zgdc
TD                    65536
SOLVENT               CDCl3
NS                        4
DS                        0
SWH               25510.203 Hz
FIDRES             0.389255 Hz
AQ                1.2845556 sec
RG                      287
DW                   19.600 usec
DE                     6.50 usec
TE                    295.8 K
D1               5.00000000 sec
D11              0.03000000 sec
TD0                       1

======== CHANNEL f1 ========
NUC1                    11B
P1                     7.60 usec
PL1                   -3.00 dB
PL1W            55.13059616 W
SFO1           128.3968556 MHz

======== CHANNEL f2 ========
CPDPRG2             waltz16
NUC2                     1H
PCPD2                 90.00 usec
PL2                   -1.00 dB
PL12                  15.16 dB
PL2W            13.56617069 W
PL12W            0.32844096 W
SFO2           400.1916008 MHz
SI                    32768
SF             128.3965728 MHz
WDW                      EM
SSB                       0
LB                     3.00 Hz
GB                        0
PC                     1.40
```

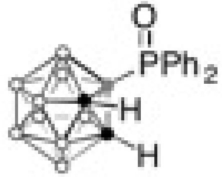

21a

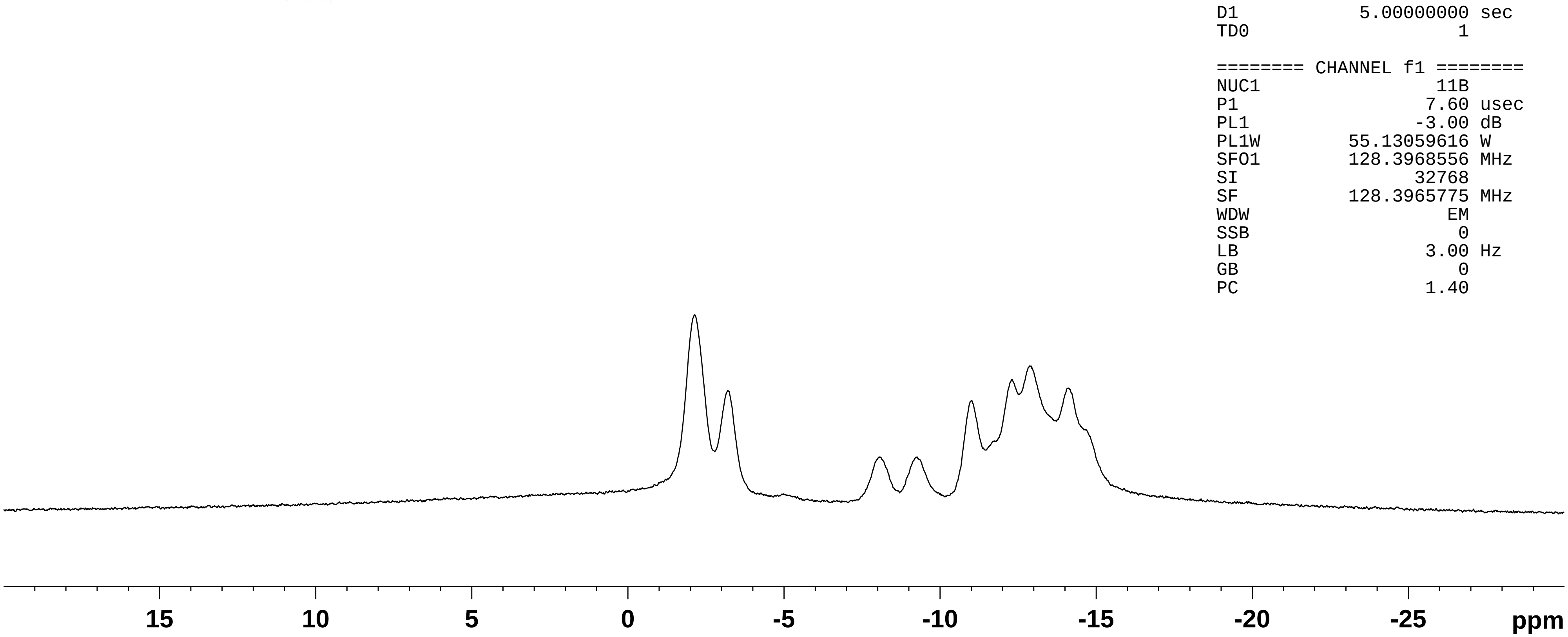
-2.132
-3.205
-8.050
-9.264
-11.017
-12.305
-12.912
-14.103
NAME ZD-787-2-acetone-B(c)
EXPNO 1
PROCNO 1
Date_ 20141016
Time 14.38
INSTRUM spect
PROBHD 5 mm PABBO BB-
PULPROG zg30
TD 65536
SOLVENT CDCl3
NS 8
DS 0
SWH 25510.203 Hz
FIDRES 0.389255 Hz
AQ 1.2845556 sec
RG 161
DW 19.600 usec
DE 6.50 usec
TE 295.5 K
D1 5.00000000 sec
TD0 1
======== CHANNEL f1 ========
NUC1 11B
P1 7.60 usec
PL1 -3.00 dB
PL1W 55.13059616 W
SFO1 128.3968556 MHz
SI 32768
SF 128.3965775 MHz
WDW EM
SSB 0
LB 3.00 Hz
GB 0
PC 1.40
15
10
5
0
-5
-10
-15
-20
-25
ppm

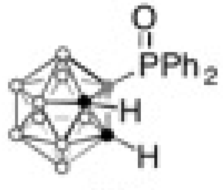

21a

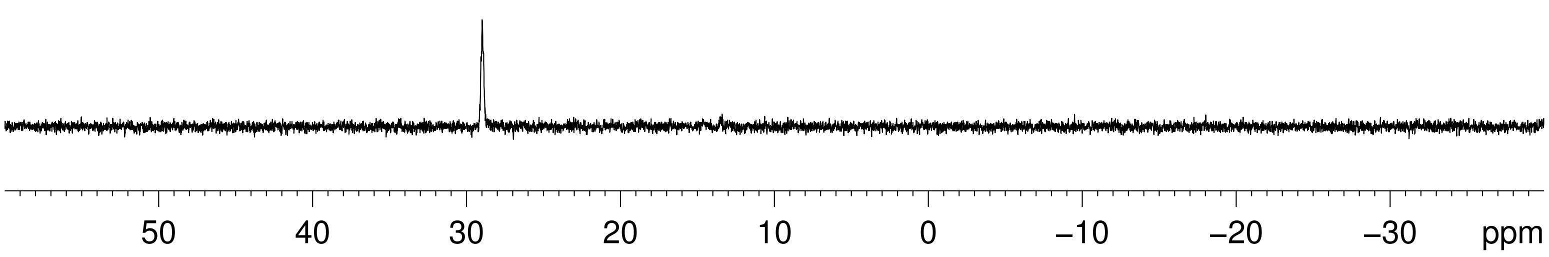

ZD-787-2-Acetone-P

NAME	ZD-787-2-Acetone-P
EXPNO	1
PROCNO	1
Date_	20141016
Time	14.27
INSTRUM	spect
PROBHD	5 mm PABBO BB-
PULPROG	zg30
TD	65536
SOLVENT	Acetone
NS	72
DS	4
SWH	64102.563 Hz
FIDRES	0.978127 Hz
AQ	0.5112308 sec
RG	2050
DW	7.800 usec
DE	6.50 usec
TE	298.0 K
D1	2.00000000 sec
TD0	1

======== CHANNEL f1 ========

NUC1	31P
P1	14.70 usec
PL1	4.00 dB
PL1W	10.30000019 W
SFO1	161.9917814 MHz
SI	32768
SF	161.9998472 MHz
WDW	EM
SSB	0
LB	1.00 Hz
GB	0
PC	1.40

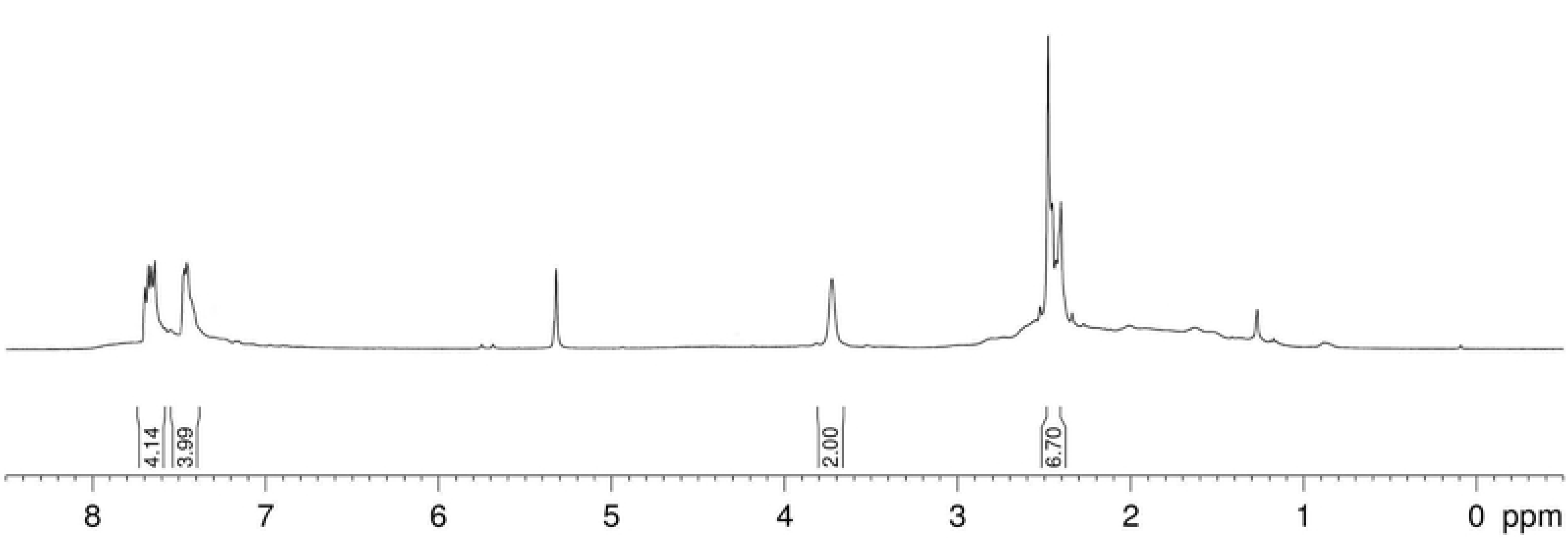

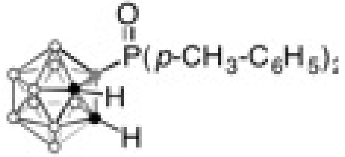

21b

ZD-792-CD2Cl2-H

NAME	ZD-792-CD2Cl2-H
EXPNO	1
PROCNO	1
Date_	20141020
Time	16.51
INSTRUM	spect
PROBHD	5 mm PABBO BB-
PULPROG	zg30
TD	65536
SOLVENT	CD2Cl2
NS	8
DS	2
SWH	8223.685 Hz
FIDRES	0.125483 Hz
AQ	3.9846387 sec
RG	50.8
DW	60.800 usec
DE	6.50 usec
TE	298.0 K
D1	1.00000000 sec
TD0	1

======== CHANNEL f1 =====

NUC1	1H
P1	14.00 usec
PL1	-1.00 dB
PL1W	13.56617069 W
SFO1	400.1924713 MHz
SI	32768
SF	400.1900207 MHz
WDW	EM
SSB	0
LB	0.30 Hz
GB	0
PC	1.00

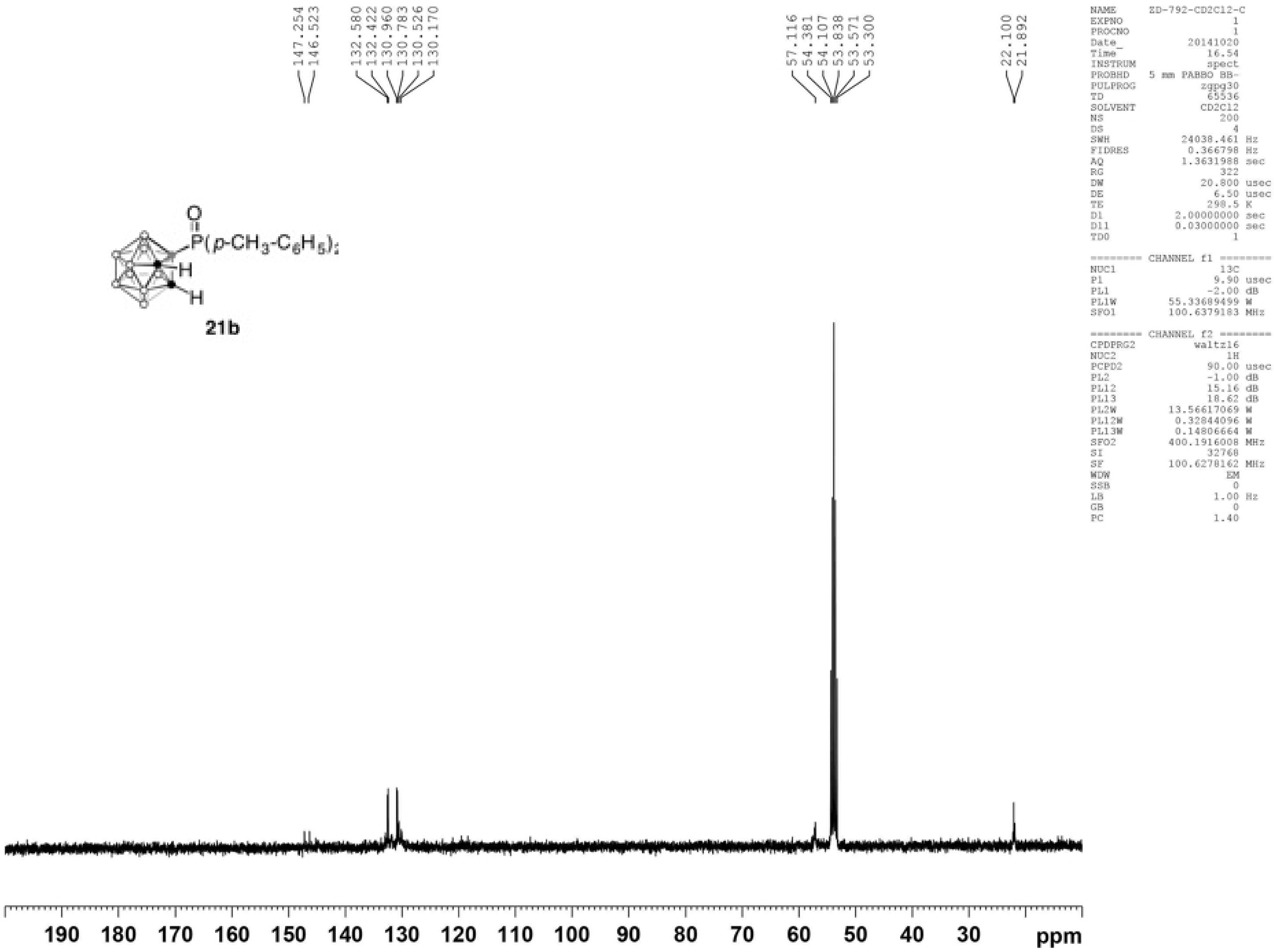
147.254
146.523
132.580
132.422
130.960
130.783
130.526
130.170
57.116
54.381
54.107
53.838
53.571
53.300
22.100
21.892
O
P(p-CH3-C6H5)2
H
H
21b
NAME ZD-792-CD2Cl2-C
EXPNO 1
PROCNO 1
Date_ 20141020
Time 16.54
INSTRUM spect
PROBHD 5 mm PABBO BB-
PULPROG zgpg30
TD 65536
SOLVENT CD2Cl2
NS 200
DS 4
SWH 24038.461 Hz
FIDRES 0.366798 Hz
AQ 1.3631988 sec
RG 322
DW 20.800 usec
DE 6.50 usec
TE 298.5 K
D1 2.00000000 sec
D11 0.03000000 sec
TD0 1
======== CHANNEL f1 ========
NUC1 13C
P1 9.90 usec
PL1 -2.00 dB
PL1W 55.33689499 W
SFO1 100.6379183 MHz
======== CHANNEL f2 ========
CPDPRG2 waltz16
NUC2 1H
PCPD2 90.00 usec
PL2 -1.00 dB
PL12 15.16 dB
PL13 18.62 dB
PL2W 13.56617069 W
PL12W 0.32844096 W
PL13W 0.14806664 W
SFO2 400.1916008 MHz
SI 32768
SF 100.6278162 MHz
WDW EM
SSB 0
LB 1.00 Hz
GB 0
PC 1.40
190
180
170
160
150
140
130
120
110
100
90
80
70
60
50
40
30
ppm

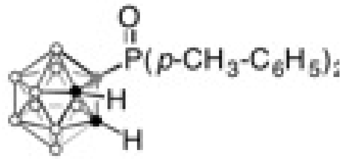

21b

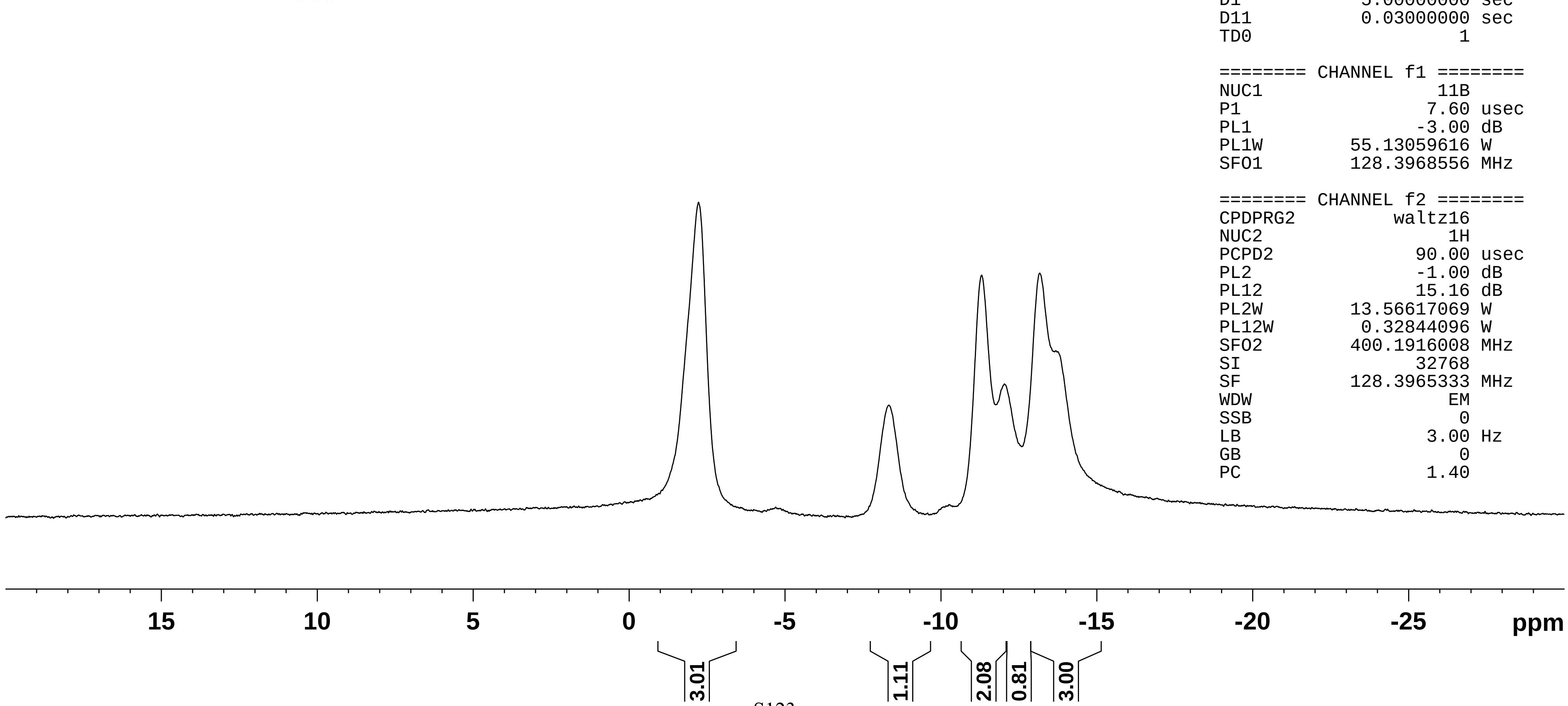
-2.229
-8.334
-11.307
-12.040
-13.170
NAME ZD-792-CD2Cl2-B(de)
EXPNO 1
PROCNO 1
Date_ 20141020
Time 16.17
INSTRUM spect
PROBHD 5 mm PABBO BB-
PULPROG zgdc
TD 65536
SOLVENT CDCl3
NS 4
DS 0
SWH 25510.203 Hz
FIDRES 0.389255 Hz
AQ 1.2845556 sec
RG 287
DW 19.600 usec
DE 6.50 usec
TE 295.8 K
D1 5.00000000 sec
D11 0.03000000 sec
TD0 1
======== CHANNEL f1 ========
NUC1 11B
P1 7.60 usec
PL1 -3.00 dB
PL1W 55.13059616 W
SFO1 128.3968556 MHz
======== CHANNEL f2 ========
CPDPRG2 waltz16
NUC2 1H
PCPD2 90.00 usec
PL2 -1.00 dB
PL12 15.16 dB
PL2W 13.56617069 W
PL12W 0.32844096 W
SFO2 400.1916008 MHz
SI 32768
SF 128.3965333 MHz
WDW EM
SSB 0
LB 3.00 Hz
GB 0
PC 1.40
15 10 5 0 -5 -10 -15 -20 -25 ppm
3.01
1.11
2.08
0.81
3.00

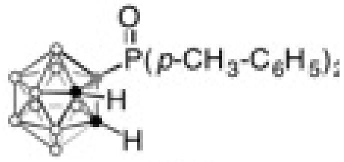

21b

```
NAME       ZD-792-CD2Cl2-B(c)
EXPNO                   1
PROCNO                  1
Date_            20141020
Time                16.21
INSTRUM             spect
PROBHD     5 mm PABBO BB-
PULPROG              zg30
TD                  65536
SOLVENT             CDCl3
NS                      8
DS                      0
SWH             25510.203 Hz
FIDRES           0.389255 Hz
AQ              1.2845556 sec
RG                    161
DW                 19.600 usec
DE                   6.50 usec
TE                  295.5 K
D1             5.00000000 sec
TD0                     1

======== CHANNEL f1 ========
NUC1                  11B
P1                   7.60 usec
PL1                 -3.00 dB
PL1W          55.13059616 W
SFO1         128.3968556 MHz
SI                  32768
SF           128.3965304 MHz
WDW                    EM
SSB                     0
LB                   3.00 Hz
GB                      0
PC                   1.40
```

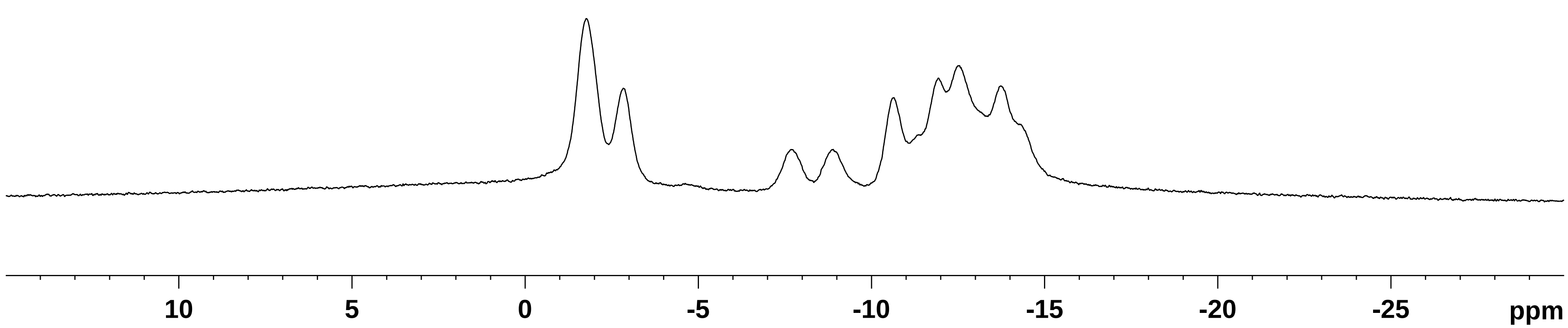

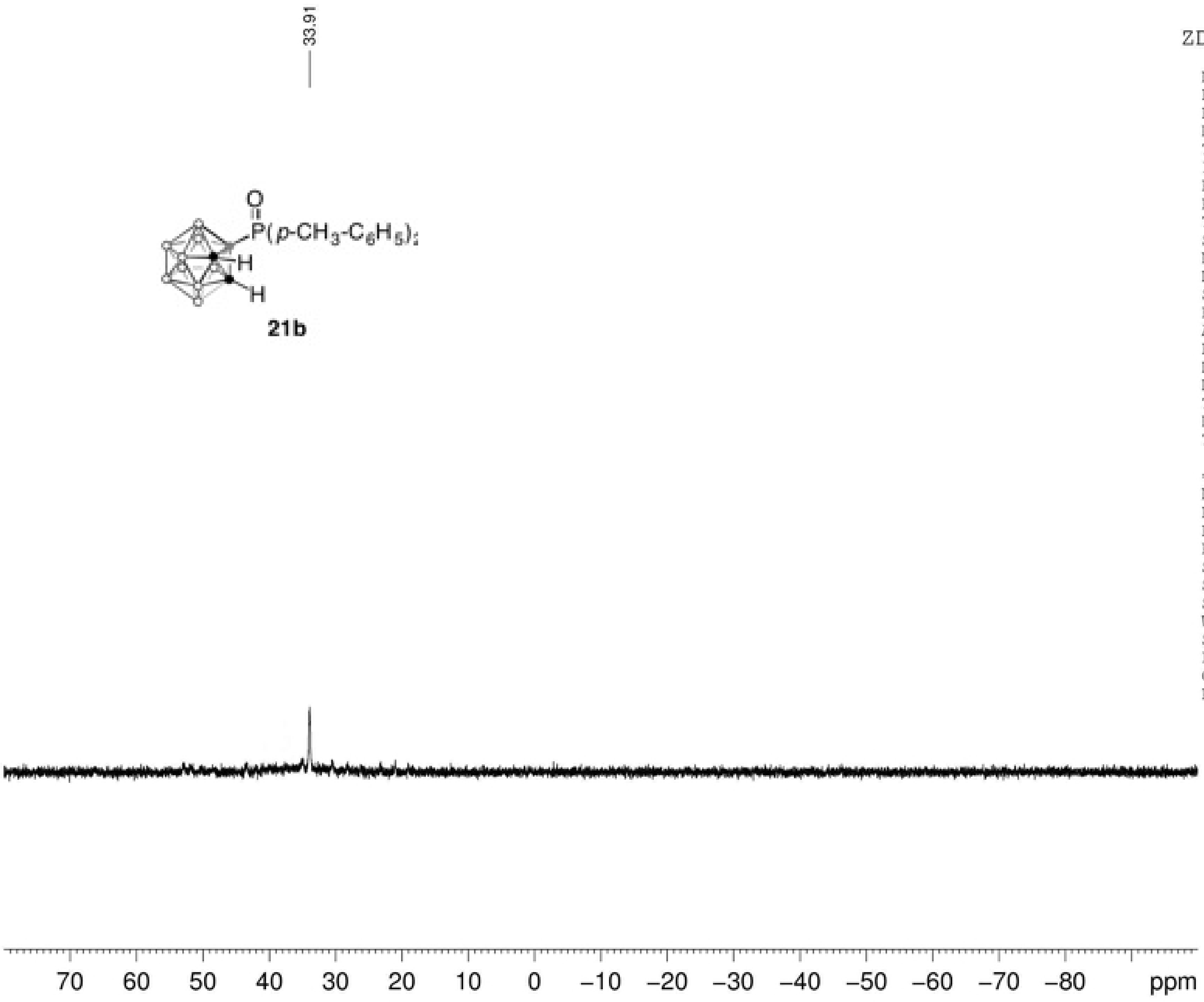

ZD-792-CD2Cl2-P

NAME	ZD-792-CD2Cl2-P	
EXPNO	1	
PROCNO	1	
Date_	20141020	
Time	17.10	
INSTRUM	spect	
PROBHD	5 mm PABBO BB-	
PULPROG	zg30	
TD	65536	
SOLVENT	CD2Cl2	
NS	40	
DS	4	
SWH	64102.563	Hz
FIDRES	0.978127	Hz
AQ	0.5112308	sec
RG	2050	
DW	7.800	usec
DE	6.50	usec
TE	298.5	K
D1	2.00000000	sec
TD0	1	
========	CHANNEL f1	========
NUC1	31P	
P1	14.70	usec
PL1	4.00	dB
PL1W	10.30000019	W
SFO1	161.9917814	MHz
SI	32768	
SF	161.9998472	MHz
WDW	EM	
SSB	0	
LB	1.00	Hz
GB	0	
PC	1.40	
